# Supplementary material for: Novel (+)-Neoisopulegol-Based O-Benzyl Derivatives as Antimicrobial Agents
Source: Int J Mol Sci. 2021 May 26;22(11):5626. doi: 10.3390/ijms22115626 (PMC8198684; doi:10.3390/ijms22115626)
Supplement: Supplementary file 1 [file ijms-22-05626-s001.zip › ijms-1215661-supplementary.pdf]

Supporting informations
for
Novel (+)-neoisopulegol based- *O*-benzyl derivatives as antibacterial agents

Tam Minh Le [1,2], Thu Huynh [3,4,5], Fatima Zahra Bamou[1], András Szekeres [3,6], Ferenc Fülöp [1,2] and Zsolt Szakonyi [1,6*]

Institute of Pharmaceutical Chemistry, University of Szeged, Interdisciplinary Excellent Center, H-6720 Szeged, Eötvös utca 6, Hungary

[2] Stereochemistry Research Group of the Hungarian Academy of Sciences, H-6720 Szeged, Eötvös utca 6, Hungary

[3] Department of Microbiology, University of Szeged, 6726 Szeged, Közép fasor 52, Hungary

[4] Department of Biotecnology, Faculty of Chemical Engineering, Ho Chi Minh University of Technology (HCMUT), 72607, Ho Chi Minh City, District 10, 268 Ly Thuong Kiet Street, Vietnam

[5] Vietnam National University Ho Chi Minh City, 71351, Ho Chi Minh City, Thu Duc District, Linh Trung Ward, Vietnam

[6] Interdisciplinary Centre of Natural Products, University of Szeged, H-6720 Szeged, Eötvös utca 6, Hungary

* Correspondence: szakonyi.zsolt@szte.hu; Tel.: +36-62-546809; Fax: +36-62-545705

Contents

1. **Complete results of antimicrobial activities** **S3-S6**
2. **^{1}H–, ^{13}C–NMR spectra of new compounds** **S7-S140**
3. **Chiral GC chromatograms of (-)- and (+)-isopulegol** **S141-S142**

Table S1: Complete results of antimicrobial activities

RSD Inhibitory effect (%) ± (%)							
		Gram positive		Gram negative		Yeast	
**Analogue**	**Conc. (µg/mL)**	***B. subtilis* SZMC0209**	***S. aureus* SZMC14611**	***E. coli* SZMC6271**	***P. aeruginosa* SZMC23290**	***C. albicans* SZMC1533**	***C. krusei* SZMC1352**
Nystatin	100	-	-	-	-	93.38 ± 2.13	92.01 ± 3.64
	10	-	-	-	-	92.88 ± 10.18	58.00 ± 9.21
Ampicillin	100	95.22 ± 8.40	81.88 ± 8.99	94.07 ± 3.61	29.03 ± 2.06	-	-
	10	93.00 ± 3.20	70.37 ± 6.15	89.37 ± 0.39	5.36 ± 8.32	-	-
**3**	100	97.60 ± 6.42	57.57 ± 9.93	26.50 ± 2.28	49.10 ± 7.52	9.38 ± 7.95	-
	10	59.58 ± 8.06	10.75 ± 5.41	24.19 ± 3.99	33.91 ± 10.35	5.22 ± 6.73	-
**5a**	100	92.82 ± 4.69	80.07 ± 2.21	38.70 ± 5.87	54.06 ± 9.08	91.56 ± 1.27	94.88 ± 2.18
	10	48.25 ± 6.16	-	24.67 ± 1.84	26.68 ± 5.56	8.67 ± 3.99	-
**5b**	100	86.35 ± 1.88	71.48 ± 1.28	29.58 ± 0.18	46.66 ± 1.37	92.88 ± 2.63	93.62 ± 0.80
	10	23.23 ± 3.15	-	16.80 ± 2.82	6.19 ± 1.77	-	-
**6a**	100	-	-	13.63 ± 0.90	4.07 ± 5.69	-	32.04 ± 1.45
	10	-	-	14.28 ± 3.11	-	-	0.16 ± 28.77
**6b**	100	-	6.78 ± 3.84	23.07 ± 3.03	-	7.32 ± 1.18	-
	10	-	-	18.66 ± 2.08	0.52 ± 8.77	6.35 ± 1.81	-
**7a**	100	81.51 ± 4.73	70.66 ± 0.91	9.10 ± 1.86	-	87.90 ± 10.46	-
	10	-	-	9.82 ± 8.12	-	2.24 ± 5.95	-
**7b**	100	95.34 ± 4.81	92.34 ± 1.32	23.19 ± 0.21	41.59 ± 3.53	22.68 ± 8.67	29.02 ± 4.69
	10	50.00 ± 7.21	2.69 ± 2.48	21.21 ± 6.20	29.09 ± 1.97	6.60 ± 1.65	-
**8a**	100	49.17 ± 2.41	3.62 ± 1.47	11.73 ± 2.01	47.26 ± 2.69	17.96 ± 1.27	-
	10	35.67 ± 4.38	2.45 ± 1.08	16.13 ± 3.04	38.18 ± 2.16	7.64 ± 0.46	7.56 ± 38.36
**8b**	100	29.88 ± 4.78	-	-	40.81 ± 2.35	14.63 ± 0.25	-
	10	18.40 ± 2.09	-	9.06 ± 0.85	35.05 ± 3.94	-	-
**9a**	100	52.37 ± 5.19	-	16.94 ± 4.24	29.26 ± 1.31	7.58 ± 1.85	-
	10	-	-	12.39 ± 4.49	8.37 ± 6.25	4.81 ± 2.74	-
**9b**	100	51.60 ± 10.30	-	13.92 ± 0.87	4.13 ± 1.12	2.33 ± 1.21	4.90 ± 49.13
	10	38.16 ± 3.93	-	12.08 ± 1.48	-	2.76 ± 1.08	-
**10**	100	95.16 ± 2.81	90.71 ± 3.27	25.74 ± 4.75	50.87 ± 9.72	95.91 ± 16.31	-
	10	55.43 ± 15.48	-	16.90 ± 2.19	44.05 ± 7.57	6.57 ± 3.05	-
**12a**	100	95.16 ± 6.46	2.81 ± 6.51	24.95 ± 4.61	70.85 ± 6.49	95.83 ± 11.18	-
	10	73.41 ± 5.45	-	17.14 ± 4.41	47.81 ± 7.92	9.34 ± 5.24	-
**12b**	100	91.84 ± 6.01	83.11 ± 2.61	50.07 ± 10.97	75.84 ± 7.14	94.50 ± 0.97	67.59 ± 16.45
	10	32.17 ± 11.19	-	14.39 ± 4.75	58.24 ± 4.20	2.44 ± 2.28	-
**13a**	100	6.59 ± 2.79	-	-	21.30 ± 6.68	-	-
	10	-	-	10.01 ± 7.48	-	1.83 ± 4.80	-
**13b**	100	51.10 ± 12.82	-	12.30 ± 3.93	48.58 ± 1.33	5.66 ± 2.32	-
	10	45.85 ± 4.38	-	11.82 ± 9.78	47.75 ± 2.01	5.68 ± 3.59	-
**14a**	100	92.67 ± 3.90	82.35 ± 3.19	6.48 ± 0.00	52.97 ± 7.47	2.69 ± 2.37	-

RSD Inhibitory effect (%) ± (%)							
		**Gram positive**		**Gram negative**		**Yeast**	
**Analogue**	**Conc. (μg/mL)**	***B. subtilis*** **SZMC0209**	***S. aureus*** **SZMC14611**	***E. coli*** **SZMC6271**	***P. aeruginosa*** **SZMC23290**	***C. albicans*** **SZMC1533**	***C. krusei*** **SZMC1352**
	10	-	1.87 ± 6.30	12.92 ± 5.21	44.00 ± 1.32	0.09 ± 5.55	-
**15a**	100	38.49 ± 6.18	-	3.57 ± 2.04	56.86 ± 11.05	6.66 ± 5.02	-
	10	7.77 ± 34.76	-	17.28 ± 0.91	44.31 ± 4.75	6.53 ± 3.65	-
**16a**	100	-	-	2.34 ± 5.51	28.43 ± 2.27	-	-
	10	-	-	8.51 ± 8.10	24.45 ± 1.41	-	4.53 ± 20.51
**16b**	100	3.09 ± 24.95	5.55 ± 5.25	24.29 ± 0.82	44.60 ± 6.69	1.14 ± 2.76	-
	10	-	7.25 ± 5.79	15.09 ± 0.78	36.77 ± 11.02	3.42 ± 1.50	4.64 ± 54.48
**18a**	100	-	-	16.80 ± 0.79	16.54 ± 1.30	-	-
	10	-	-	11.94 ± 0.25	17.34 ± 3.26	-	0.74 ± 48.92
**20a**	100	84.57 ± 3.18	70.13 ± 0.90	22.02 ± 2.97	-	91.35 ± 1.07	-
	10	89.70 ± 1.32	65.81 ± 0.51	2.36 ± 2.13	-	6.76 ± 8.04	14.72 ± 10.68
**20b**	100	78.34 ± 2.51	69.49 ± 0.57	22.78 ± 3.27	-	90.74 ± 2.90	79.88 ± 3.39
	10	78.43 ± 5.39	61.84 ± 0.27	17.87 ± 3.01	-	80.54 ± 17.23	-
**21a**	100	53.71 ± 2.83	8.71 ± 2.58	15.80 ± 1.19	35.11 ± 6.17	5.54 ± 4.07	-
	10	-	-	14.35 ± 3.99	25.39 ± 9.90	1.92 ± 4.53	-
**21b**	100	15.01 ± 3.04	10.23 ± 2.37	7.27 ± 2.34	33.25 ± 1.72	7.85 ± 4.93	-
	10	-	-	4.58 ± 8.16	24.48 ± 10.32	-	-
**22a**	100	83.44 ± 20.97	76.39 ± 1.13	10.96 ± 0.90	32.16 ± 8.18	37.72 ± 8.39	27.69 ± 8.00
	10	81.63 ± 1.22	70.02 ± 1.01	4.10 ± 2.99	-	5.78 ± 4.38	-
**22b**	100	78.43 ± 10.14	60.32 ± 1.11	2.91 ± 1.54	20.72 ± 4.93	81.97 ± 4.00	-
	10	81.01 ± 1.08	62.77 ± 0.27	4.19 ± 3.55	-	61.02 ± 6.51	-
**23a**	100	73.83 ± 4.14	73.99 ± 5.15	16.44 ± 0.57	47.92 ± 1.67	9.17 ± 1.39	-
	10	83.29 ± 5.94	2.51 ± 3.47	18.28 ± 13.03	47.78 ± 3.40	8.54 ± 3.44	-
**23b**	100	75.64 ± 0.21	71.95 ± 4.38	3.41 ± 2.52	46.03 ± 2.10	6.30 ± 0.86	-
	10	77.54 ± 5.94	-	-	42.22 ± 1.49	4.17 ± 2.49	-
**18b**	100	57.36 ± 4.28	-	8.22 ± 1.06	-	22.57 ± 6.29	-
	10	-	-	2.74 ± 0.40	0.34 ± 28.88	-	-
**25a**	100	78.96 ± 0.88	-	10.51 ± 1.91	-	-	-
	10	-	-	6.32 ± 2.1	-	-	-
**25b**	100	42.11 ± 10.10	3.04 ± 2.13	11.20 ± 2.31	35.40 ± 10.64	5.25 ± 0.90	-
	10	-	3.51 ± 5.19	10.75 ± 5.28	37.23 ±7.38	2.01 ± 3.69	-
**26a**	100	-	5.14 ± 4.72	1.62 ± 4.90	36.60 ± 4.48	2.42 ± 1.77	-
	10	-	-	-	33.48 ±4.53	-	-
**26b**	100	-	-	-	33.56 ± 4.72	-	-
	10	-	-	4.65 ± 0.86	33.94 ± 9.15	-	9.56 ± 14.00
**27a**	100	71.13 ± 4.78	-	13.32 ± 2.05	43.48 ± 3.42	27.31 ± 7.74	-
	10	-	-	9.39 ± 5.34	38.95 ± 9.32	2.69 ± 6.48	-
**27b**	100	-	-	0.86 ± 8.18	34.19 ± 6.00	80.58 ± 12.34	-
	10	-	-	2.29 ± 4.70	33.16 ± 8.01	24.80 ± 4.83	-
**28a**	100	58.49 ± 1.06	-	3.77 ± 6.14	26.28 ± 2.93	64.44 ± 8.94	-

RSD Inhibitory effect (%) ± (%)							
		Gram positive		Gram negative		Yeast	
Analogue	Conc. (µg/mL)	*B. subtilis* SZMC0209	*S. aureus* SZMC14611	*E. coli* SZMC6271	*P. aeruginosa* SZMC23290	*C. albicans* SZMC1533	*C. krusei* SZMC1352
	10	-	-	9.75 ± 0.64	26.51 ± 0.64	-	6.49 ± 21.22
**28b**	100	67.24 ± 9.14	-	12.63 ± 9.33	27.89 ± 0.56	41.19 ± 9.83	14.84 ± 6.96
	10	0.30 ± 11.11	2.51 ± 4.71	10.13 ± 6.13	26.11 ± 3.08	17.80 ± 4.06	-
**30b**	100	17.74 ± 6.94	-	-	-	0.96 ± 1.11	27.46 ± 3.59
	10	-	-	-	-	-	-
**31a**	100	95.13 ± 9.21	82.58 ± 10.08	30.12 ± 0.97	48.38 ± 1.94	17.28 ± 7.99	-
	10	12.76 ± 9.95	13.68 ± 5.80	24.59 ± 3.59	32.10 ± 3.98	1.69 ± 0.64	28.20 ± 1.39
**31b**	100	93.89 ± 5.51	86.85 ± 4.00	23.33 ± 2.01	53.31 ± 4.84	95.21 ± 3.59	38.27 ± 2.37
	10	47.83 ± 9.92	-	13.89 ± 2.43	47.81 ± 6.60	6.76 ± 0.58	-
**32a**	100	39.97 ± 6.05	-	9.06 ± 0.93	33.08 ± 5.20	14.04 ± 4.77	-
	10	8.61 ± 8.01	-	12.18 ± 4.13	22.81 ± 3.94	-	5.10 ± 40.80
**32b**	100	41.93 ± 6.93	-	7.84 ± 5.50	37.43 ± 2.96	14.38 ± 3.07	-
	10	1.19 ± 15.68	-	9.10 ± 1.81	22.01 ± 4.78	-	-
**34a**	100	-	-	1.05 ±1.01	23.82 ± 8.60	-	-
	10	-	-	8.56 ± 0.59	27.34 ± 2.69	-	-
**35a**	100	69.20 ± 0.76	12.92 ± 7.01	13.23 ± 3.67	24.51 ± 9.67	-	-
	10	-	-	10.41 ± 0.37	22.04 ± 3.36	-	11.48 ± 9.50
**35b**	100	-	7.07 ± 2.14	21.90 ± 0.11	32.59 ± 4.02	3.95 ± 4.36	2.26 ± 48.23
	10	-	-	11.01 ± 4.49	31.38 ± 7.59	14.47 ± 11.24	8.57 ± 38.32
**36a**	100	51.90 ± 14.00	-	14.16 ± 1.44	36.89 ± 4.71	3.61 ± 0.15	-
	10	0.83 ± 5.73	2.69 ± 2.36	13.04 ± 1.52	38.44 ± 5.81	-	15.49 ± 9.99
**36b**	100	-	-	6.91 ± 2.33	29.23 ± 4.53	48.67 ± 1.97	-
	10	-	-	5.53 ± 6.56	5.04 ± 6.13	14.42 ± 6.02	-
**38a**	100	-	2.05 ± 1.05	1.86 ± 2.39	-	4.00 ± 4.96	-
	10	-	-	7.34 ± 3.49	-	1.69 ± 0.89	1.85 ± 50.46
**38b**	100	40.89 ± 6.41	5.08 ± 1.72	6.53 ± 1.79	-	-	1.76 ± 48.50
	10	-	-	6.24 ± 3.32	1.15 ± 10.83	-	3.57 ± 46.50
**39a**	100	79.38 ± 4.19	63.47 ± 4.90	15.94 ± 1.38	-	88.22 ± 3.96	20.44 ± 22.00
	10	82.73 ± 0.52	69.84 ± 0.00	10.37 ± 1.56	-	-	-
**39b**	100	87.80 ± 7.04	79.66 ± 2.59	12.08 ± 4.28	48.09 ± 1.38	90.89 ± 13.31	91.08 ± 4.90
	10	92.94 ± 1.46	83.69 ± 38.18	16.37 ± 2.25	33.59 ± 6.43	85.10 ± 9.56	-
**40a**	100	72.88 ± 1.68	68.26 ± 1.66	12.75 ± 0.66	37.66 ± 2.39	1.07 ± 2.07	13.12 ± 35.38
	10	55.43 ± 5.07	-	1.14 ± 14.60	38.89 ± 1.13	7.90 ± 3.78	-
**40b**	100	71.39 ± 3.84	69.37 ± 1.44	3.81 ± 7.29	42.13 ± 2.25	9.22 ± 1.99	-
	10	65.82 ± 4.56	12.39 ± 1.50	5.08 ± 0.44	39.58 ± 0.73	16.79 ± 6.53	-
**41a**	100	20.92 ± 4.79	-	7.53 ± 1.10	-	-	-
	10	-	-	5.55 ± 2.23	-	-	9.76 ± 13.24
**41b**	100	-	-	22.59 ± 1.73	29.69 ± 7.98	8.01 ± 0.15	12.17 ± 13.52
	10	6.94 ± 12.97	-	21.97 ± 1.20	24.05 ± 3.11	7.78 ± 0.59	13.86 ± 12.14
**42a**	100	69.05 ± 10.02	5.79 ± 6.44	11.70 ± 2.09	26.71 ± 7.59	3.20 ± 0.25	-

RSD Inhibitory effect (%) ± (%)							
		**Gram positive**		**Gram negative**		**Yeast**	
**Analogue**	**Conc. (μg/mL)**	***B. subtilis*** **SZMC0209**	***S. aureus*** **SZMC14611**	***E. coli*** **SZMC6271**	***P. aeruginosa*** **SZMC23290**	***C. albicans*** **SZMC1533**	***C. krusei*** **SZMC1352**
	10	51.75 ± 11.13	-	10.30 ± 10.48	25.65 ± 4.83	1.12 ± 2.51	-
**42b**	100	86.62 ± 8.48	66.22 ± 4.03	11.13 ± 1.43	26.31 ± 3.55	30.69 ± 3.51	32.51 ± 8.51
	10	43.95 ± 5.65	-	8.70 ± 5.49	12.78 ± 2.95	-	16.64 ± 7.37
**43a**	100	41.19 ± 1.97	-	2.57 ± 10.50	-	5.02 ± 2.65	-
	10	-	-	8.44 ± 0.73	9.86 ± 4.63	-	-
**43b**	100	4.63 ± 24.25	-	25.00 ± 4.39	20.61 ± 4.03	80.90 ± 4.76	18.34 ± 20.72
	10	-	-	15.68 ± 1.68	8.48 ± 3.77	20.45 ± 13.47	-

^{1}H- and ^{13}C-NMR spectra of new compounds

Figure S 1: ^{1}H-NMR of compound **3**

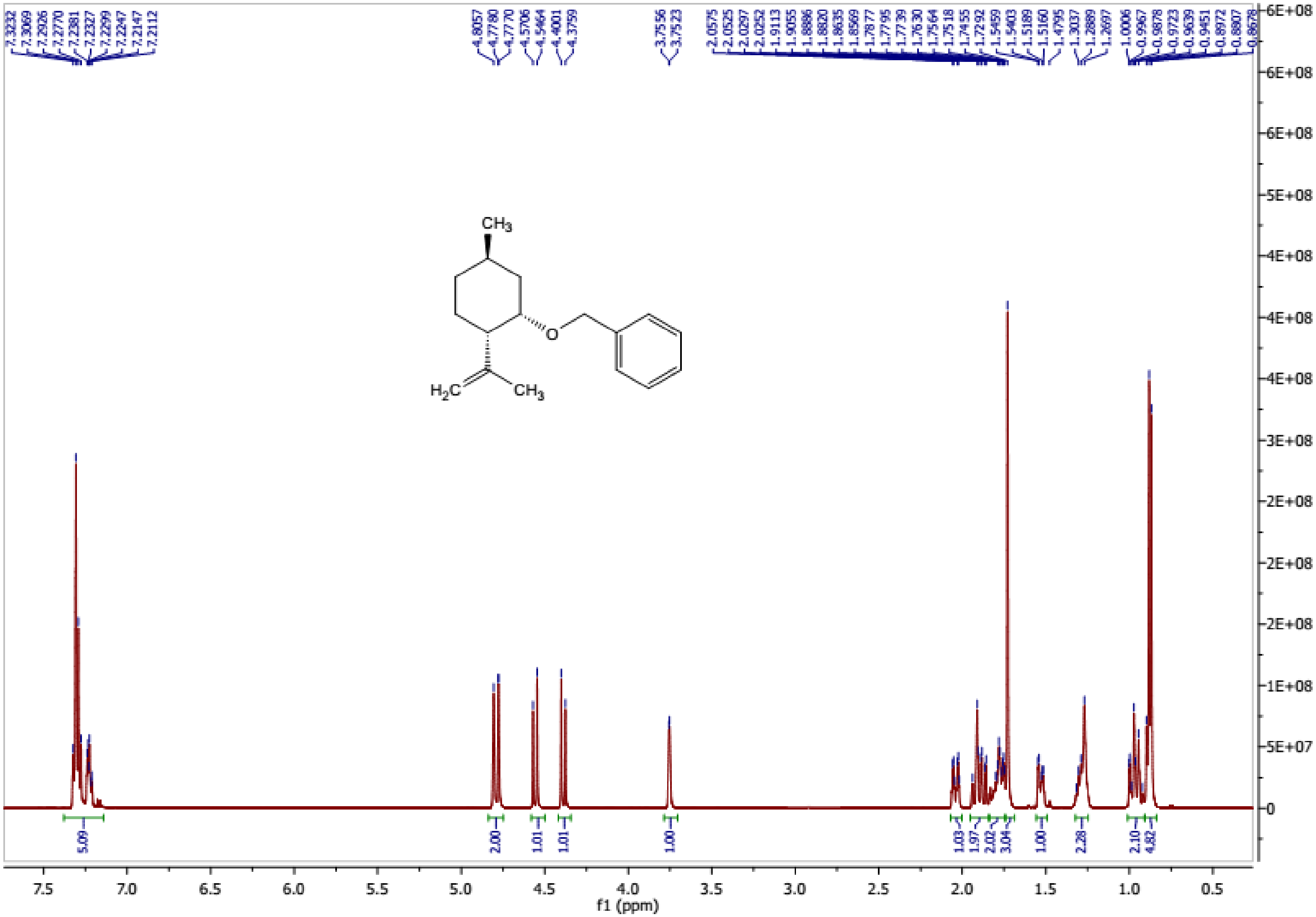

Figure S 2: ^{13}C-NMR of compound **3**

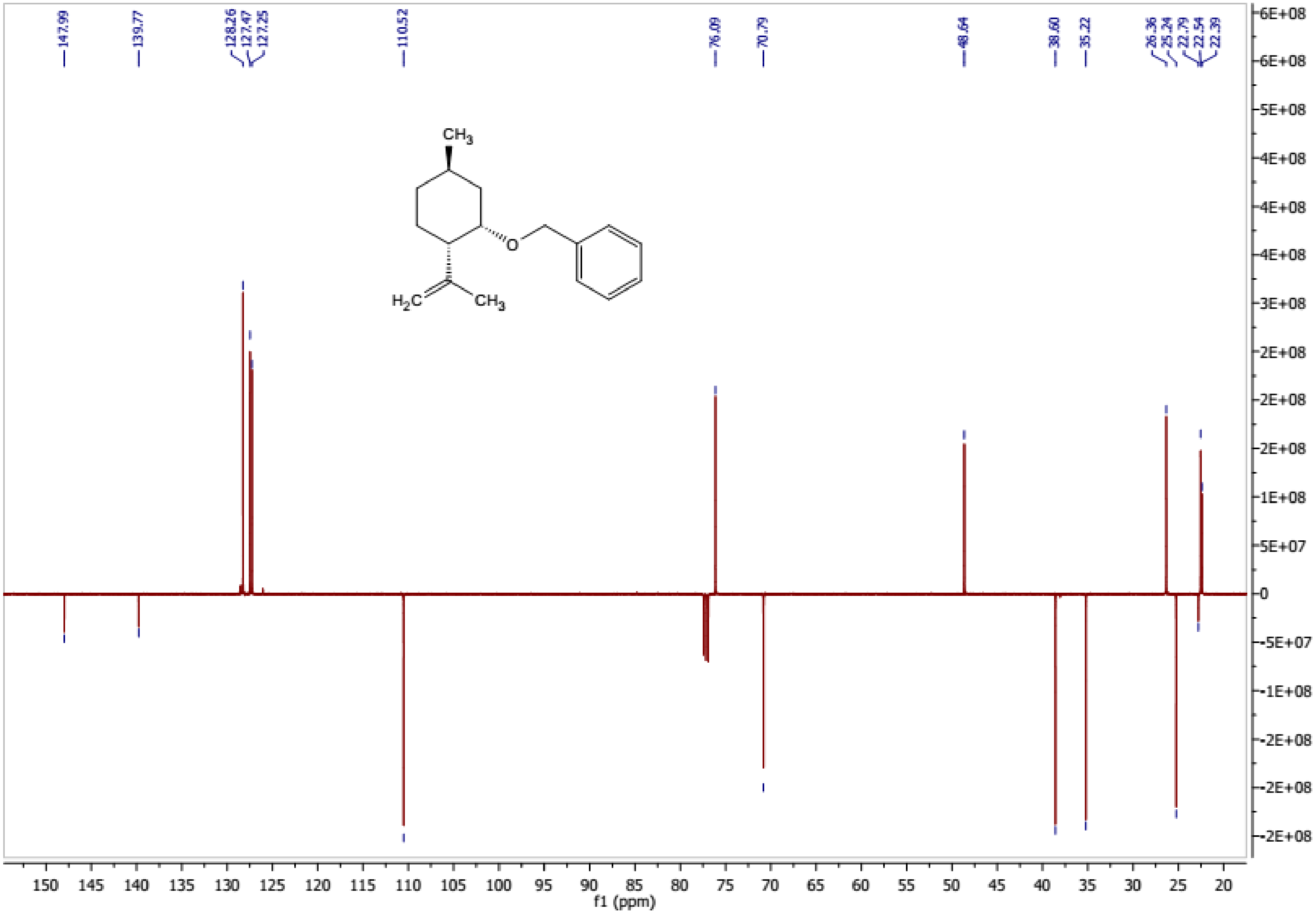

Figure S 3: ^{1}H-NMR of compound **4a**

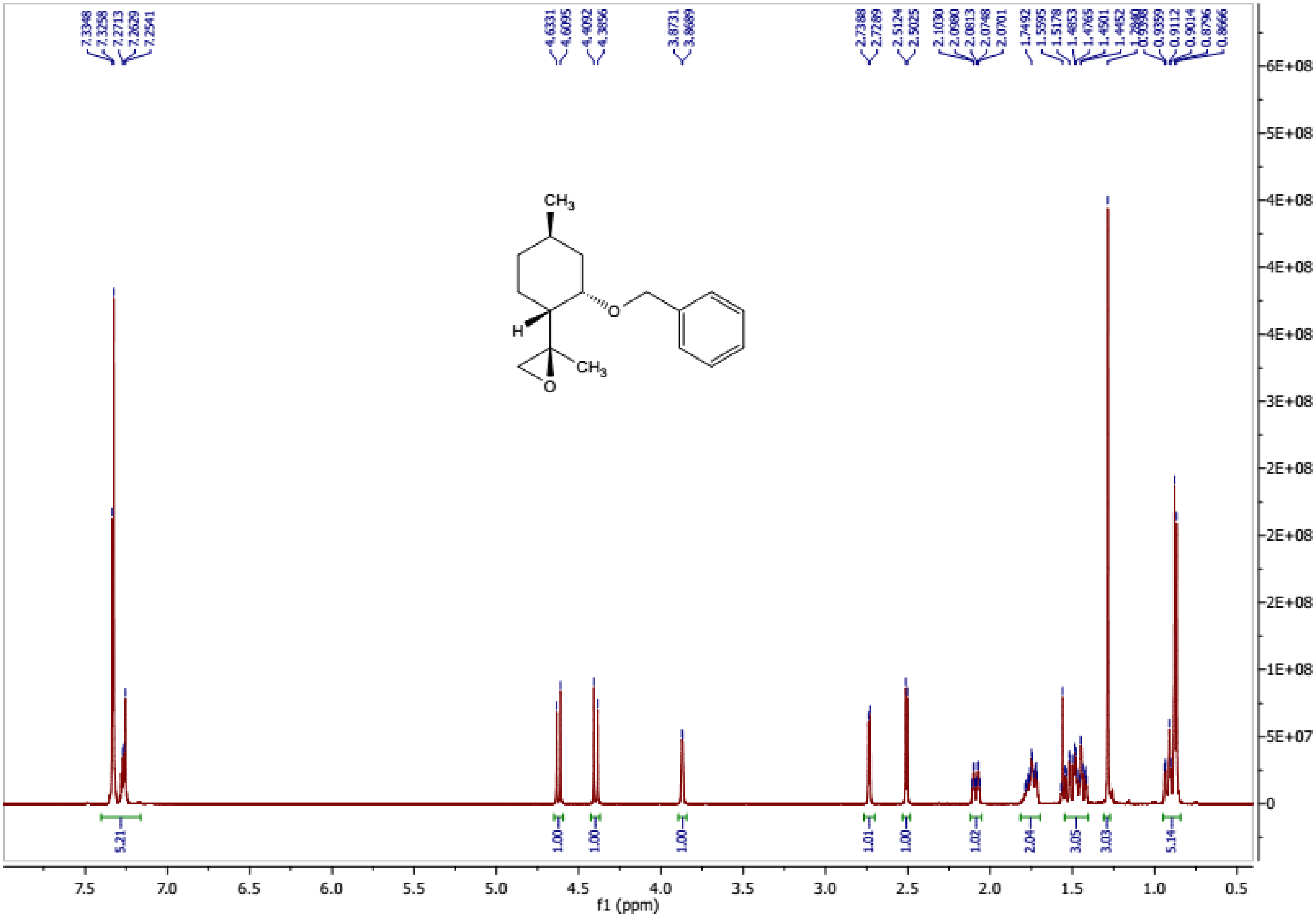

Figure S 4: ^{13}C-NMR of compound **4a**

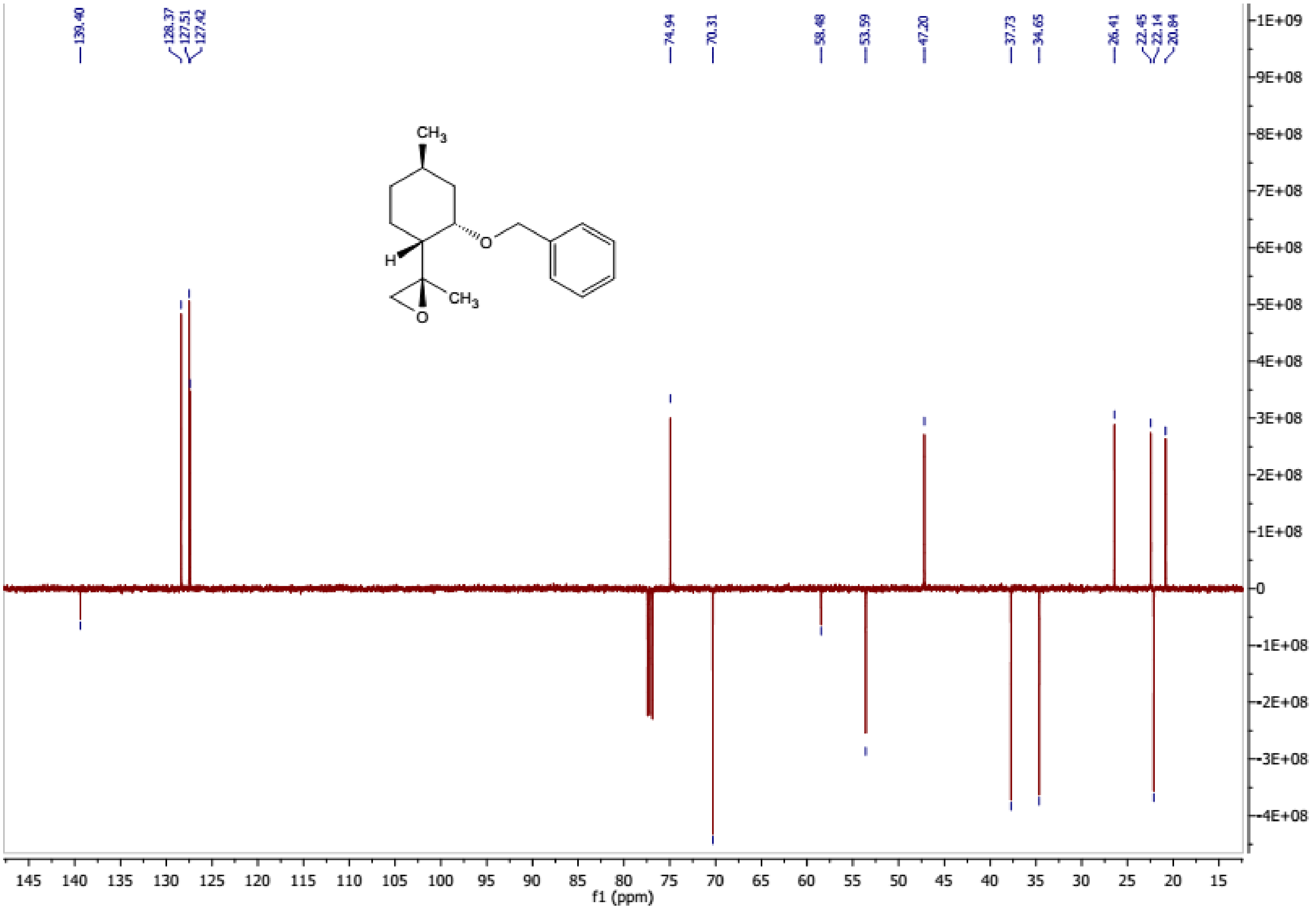

Figure S 5: ¹H-NMR of compound **4b**

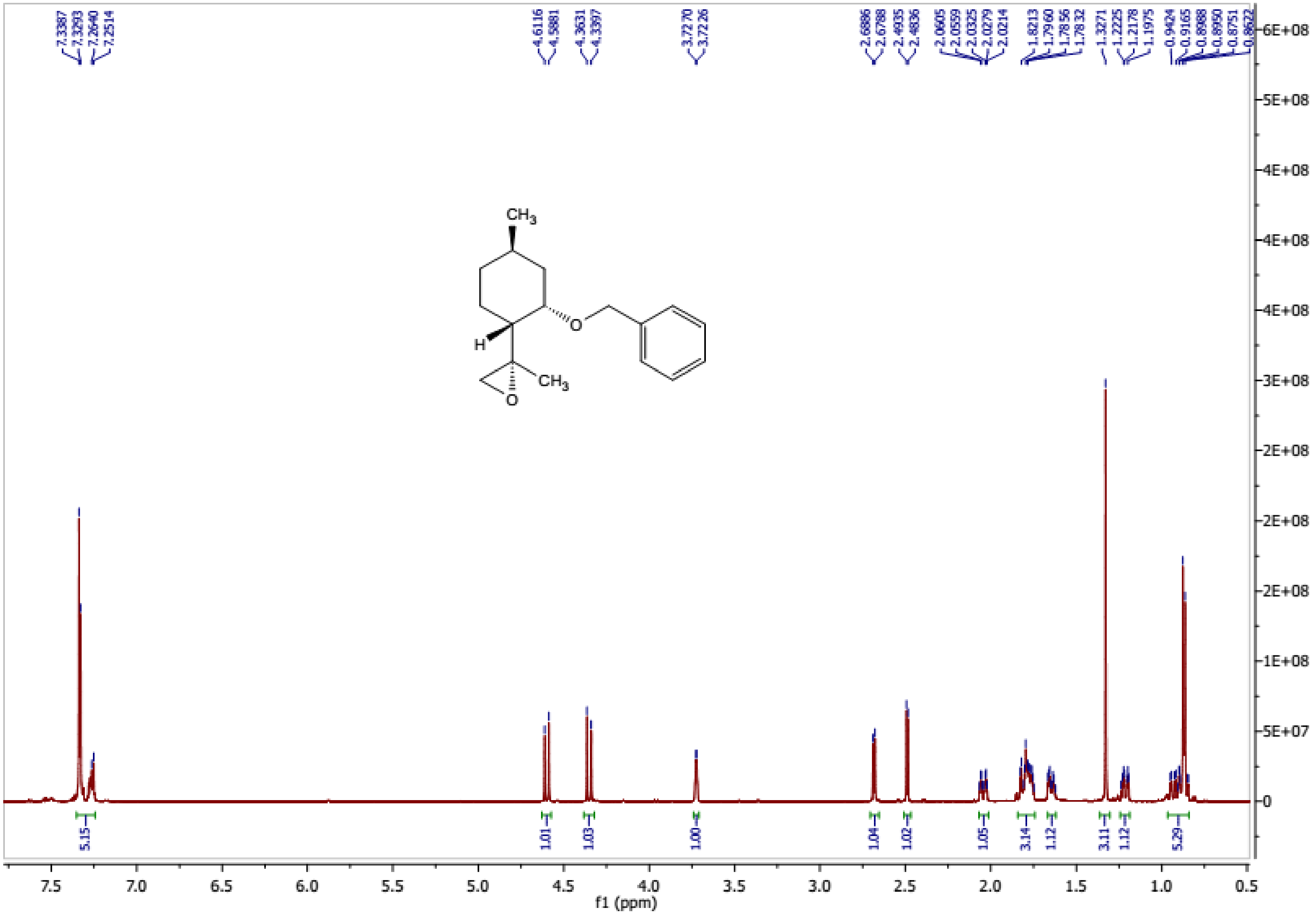

Figure S 6: ^{13}C-NMR of compound **4b**

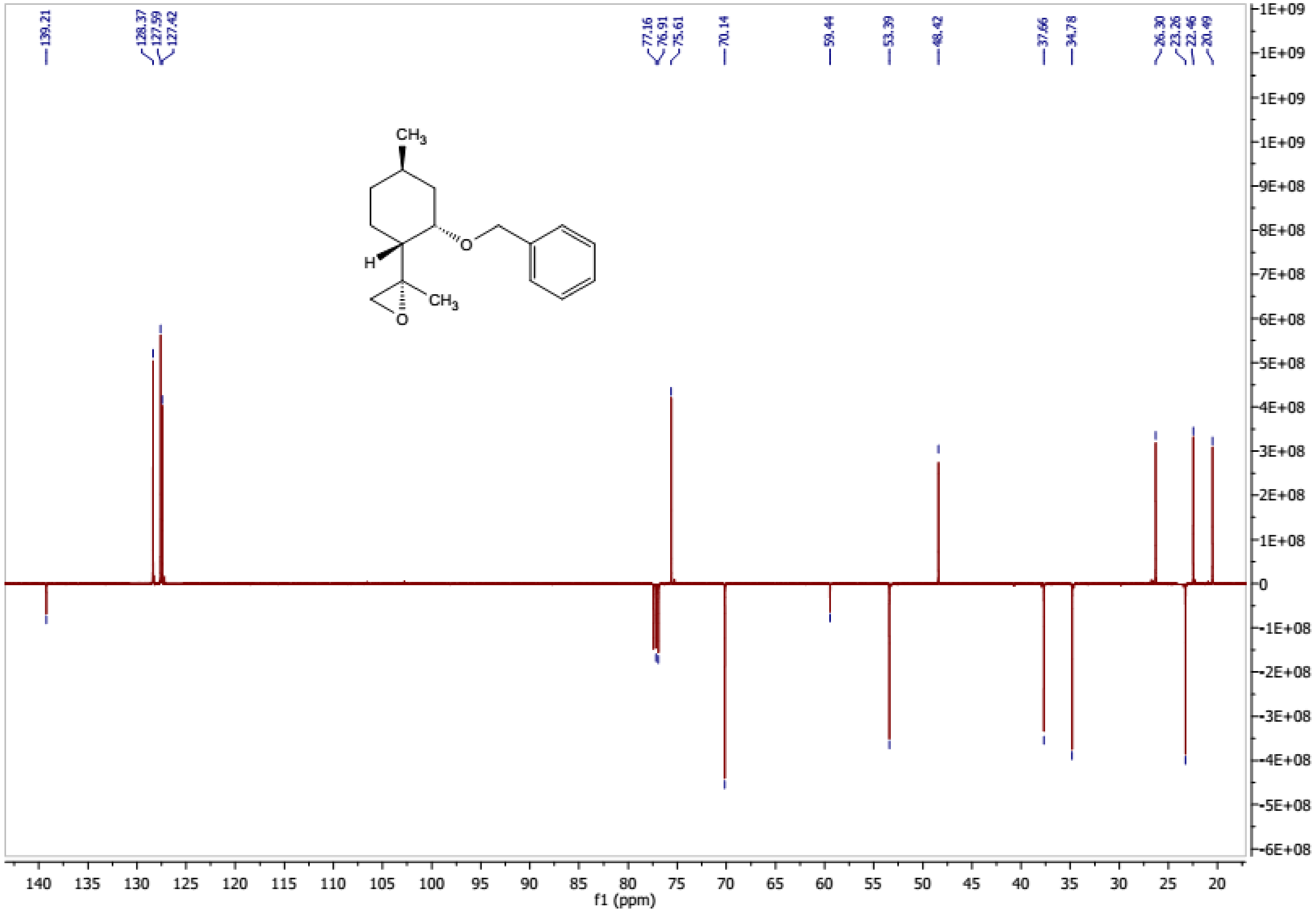

Figure S 7: ^1H-NMR of compound **5a**

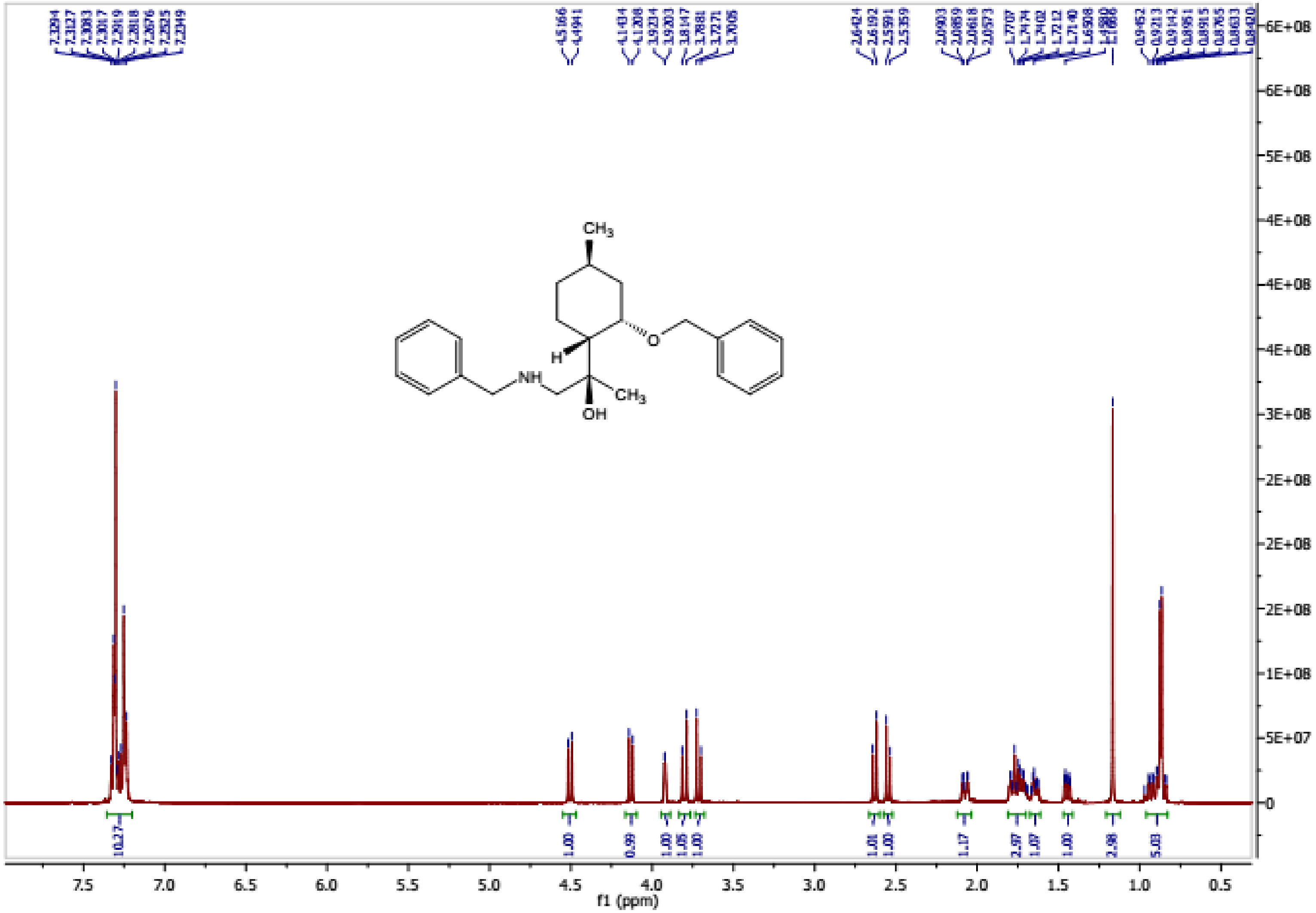

Figure S 8: ^{13}C-NMR of compound **5a**

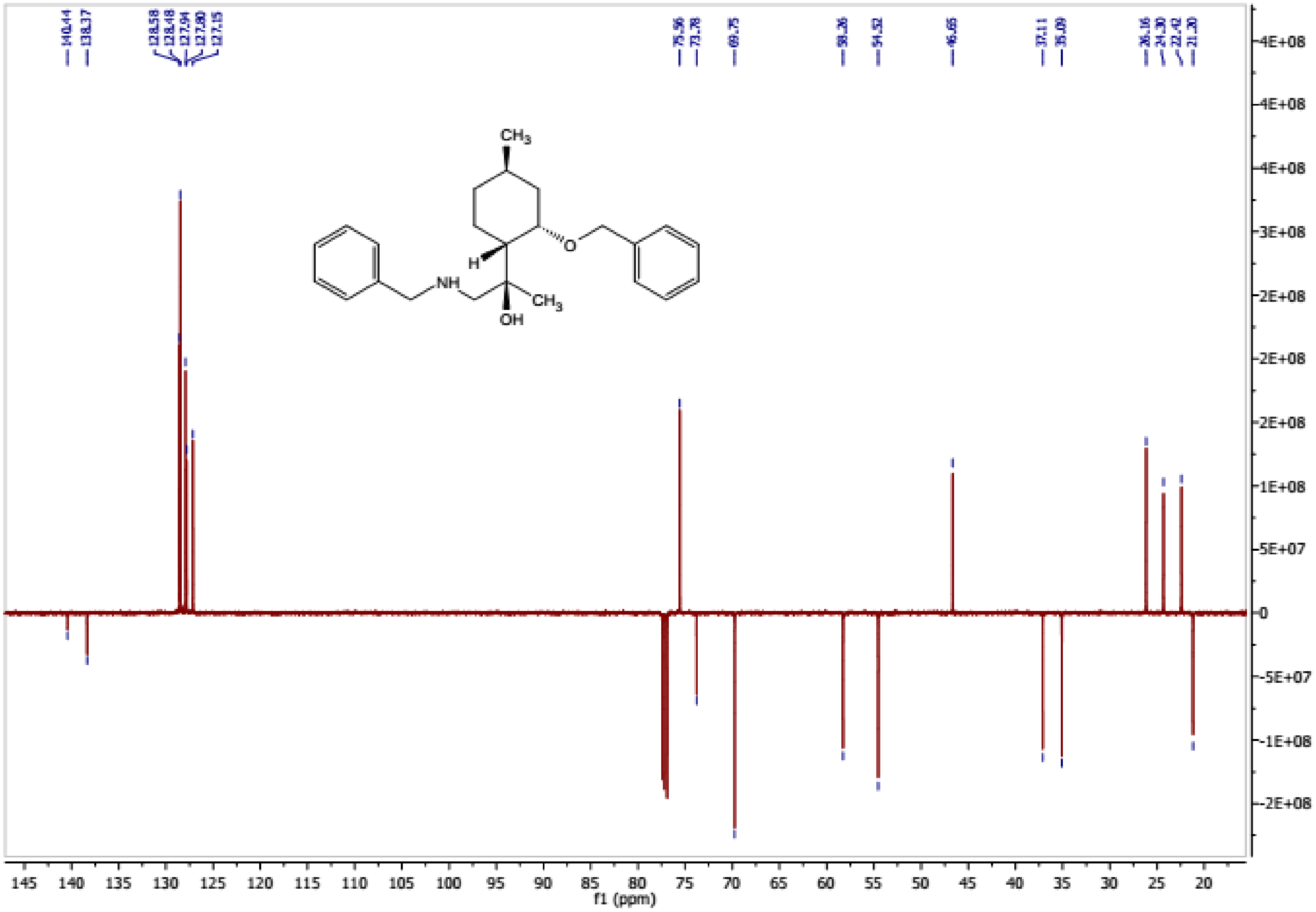

Figure S 9: ^{1}H-NMR of compound **5b**

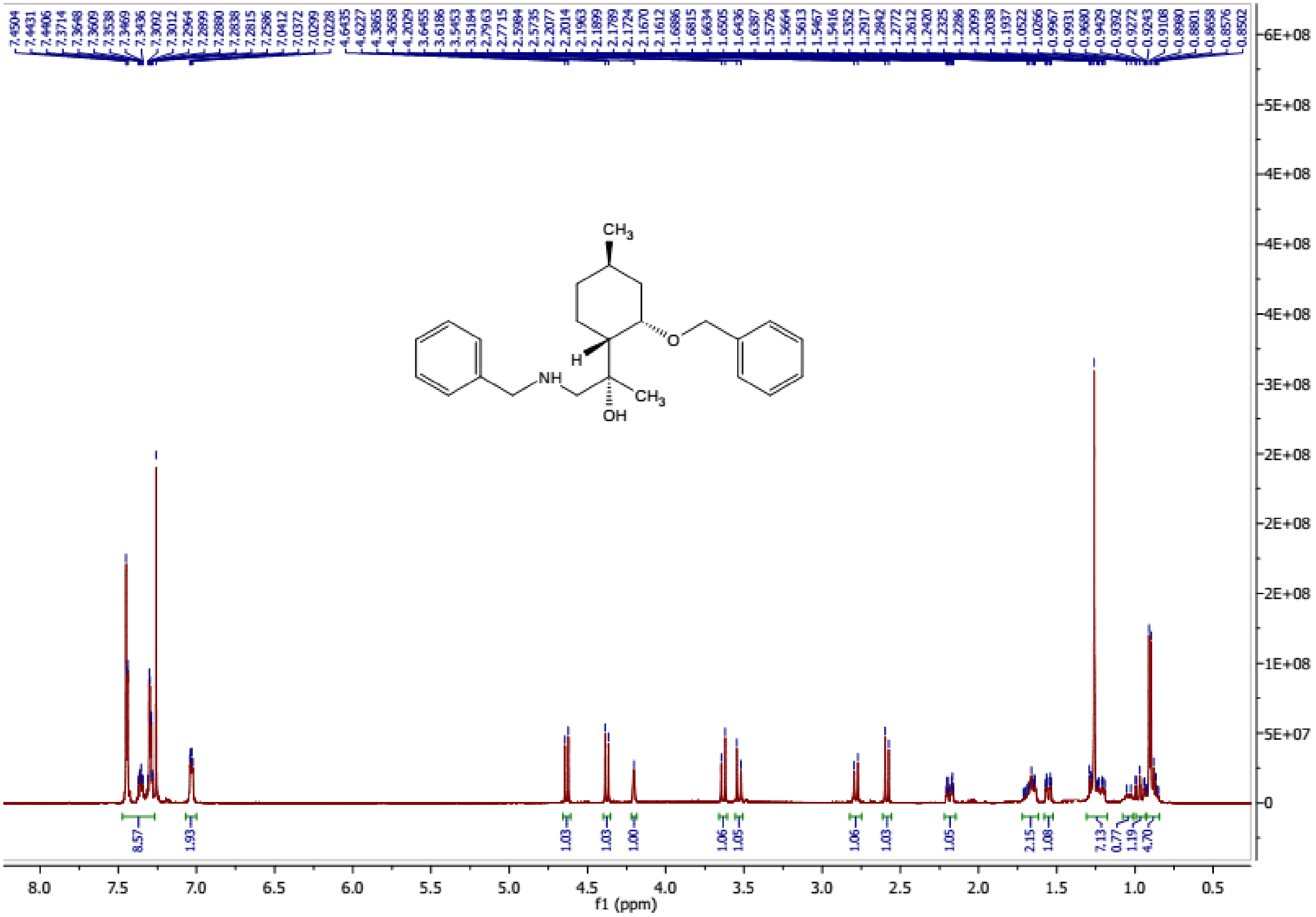

Figure S 10: ^{13}C-NMR of compound **5b**

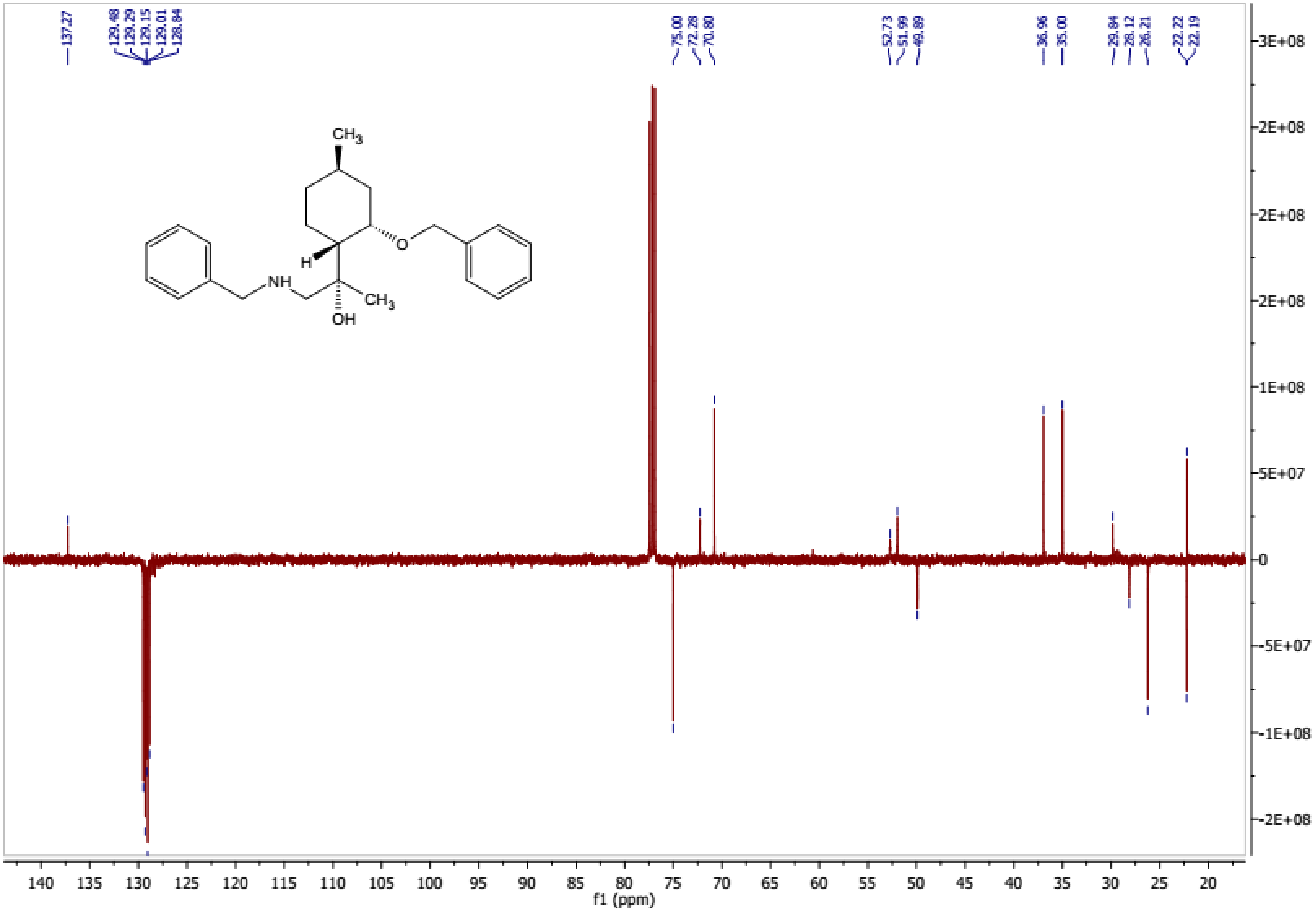

Figure S 11: ^{1}H-NMR of compound **6a**

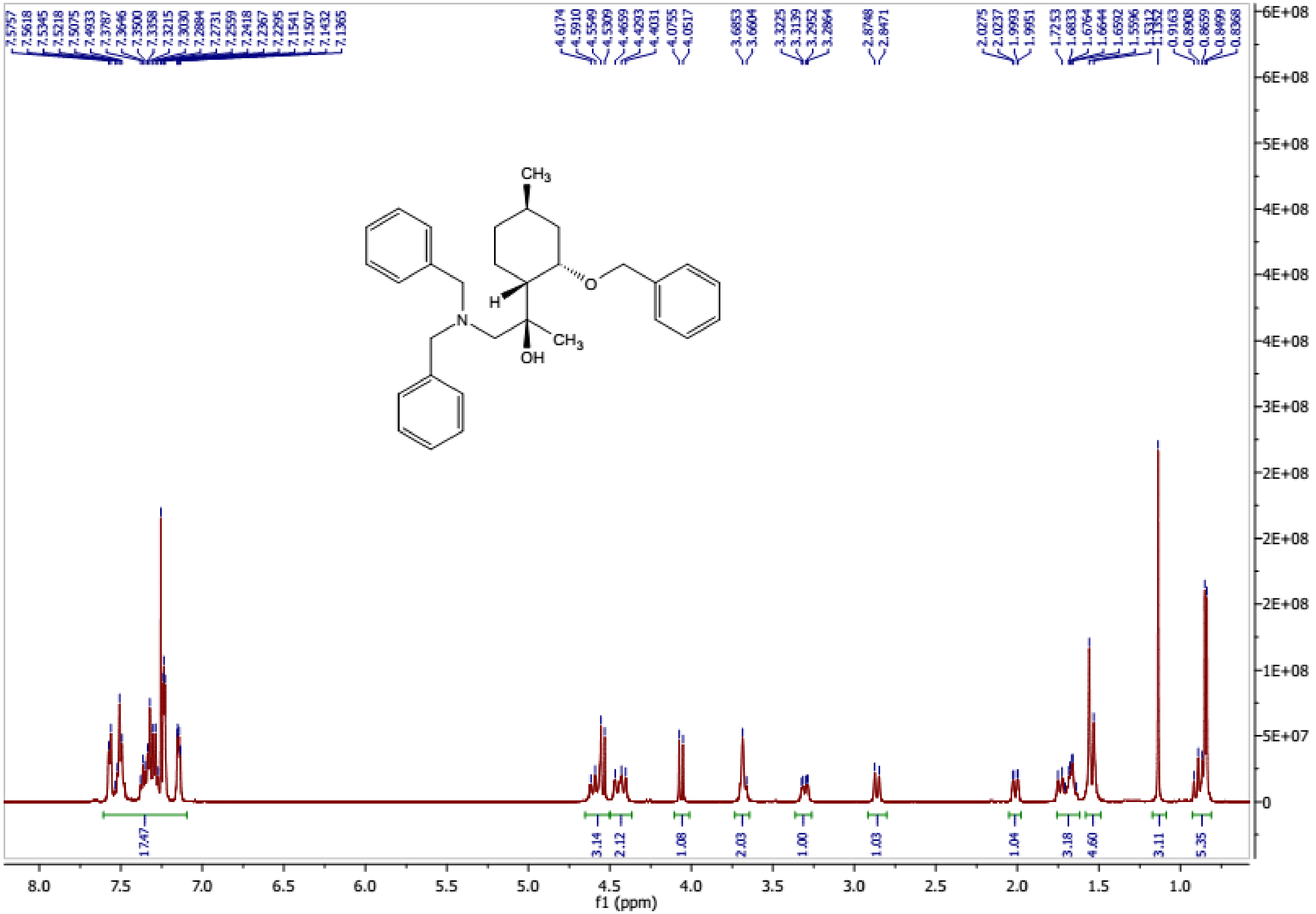

Figure S 12: ^{13}C-NMR of compound **6a**

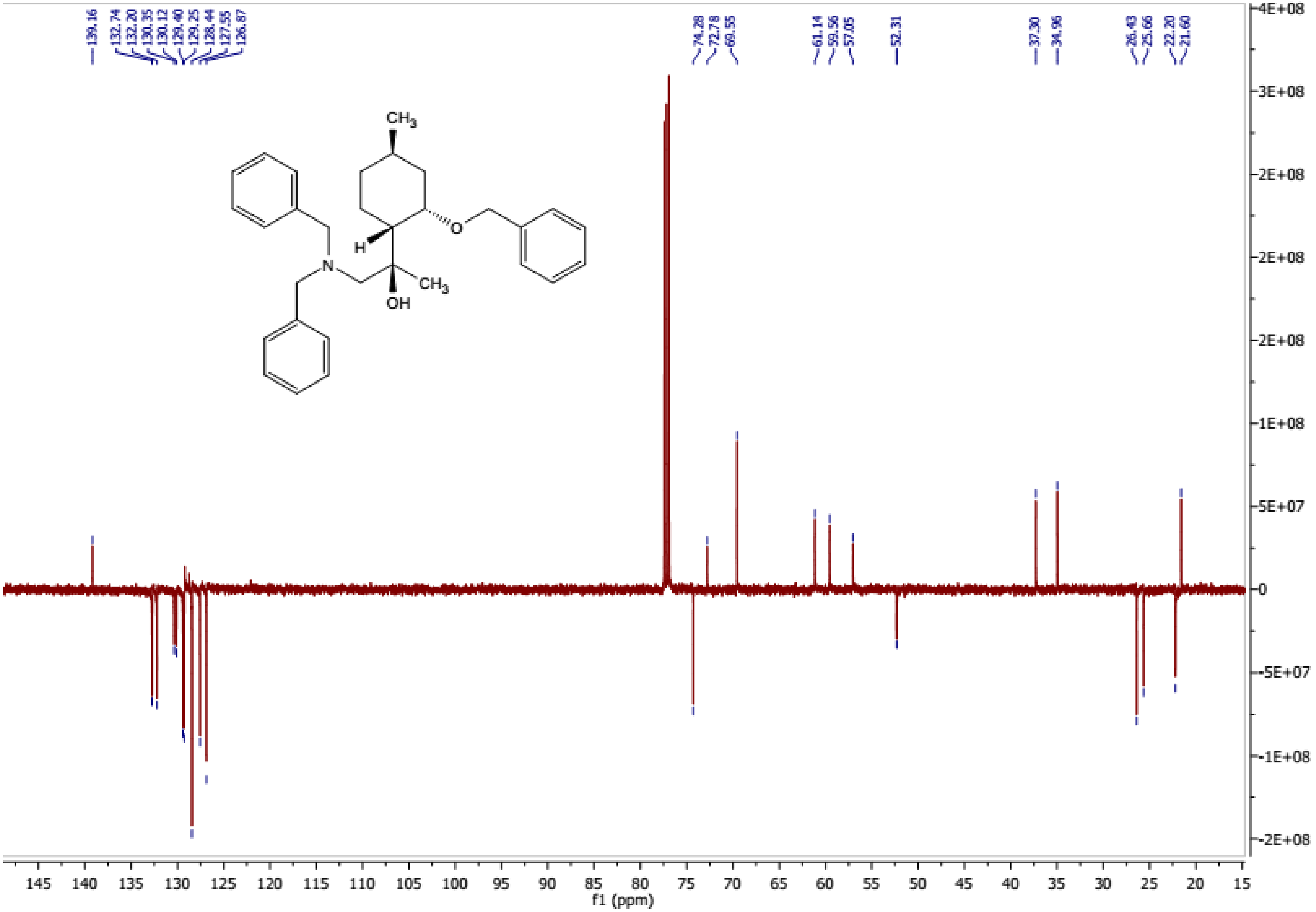

Figure S 13: ¹H-NMR of compound **6b**

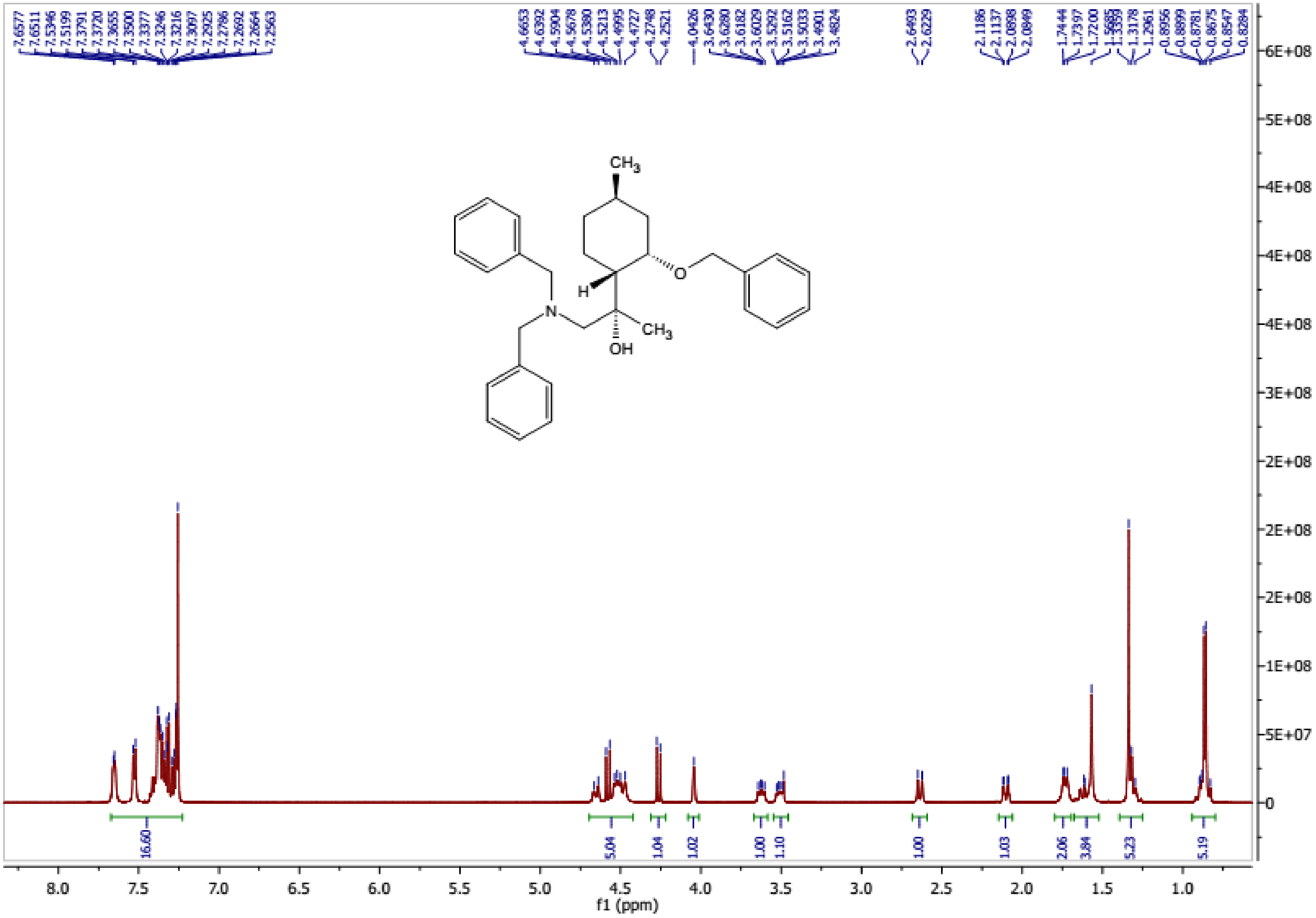

^{13}C-NMR of compound **6a**

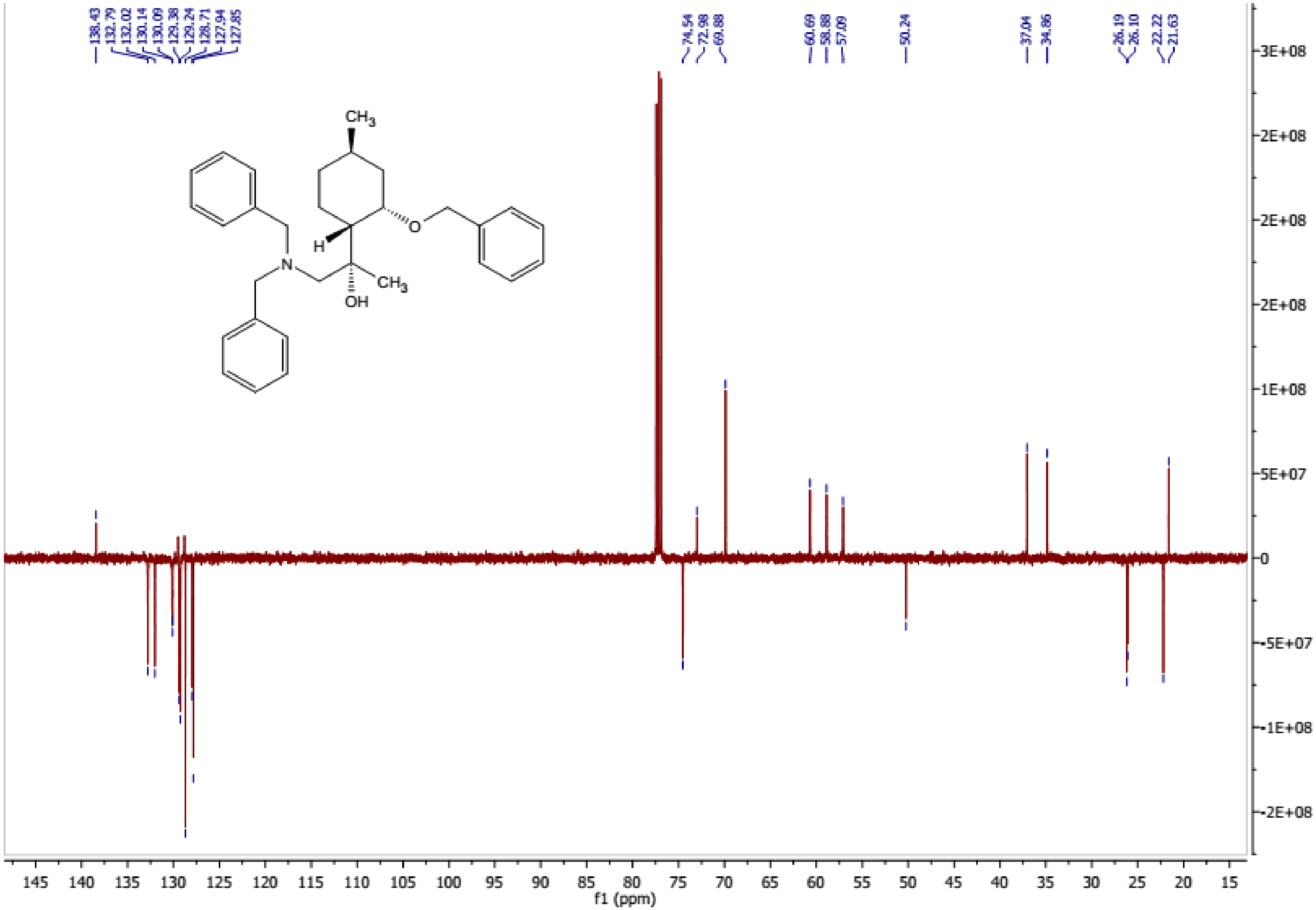

Figure S 14: ^{1}H-NMR of compound **7a**

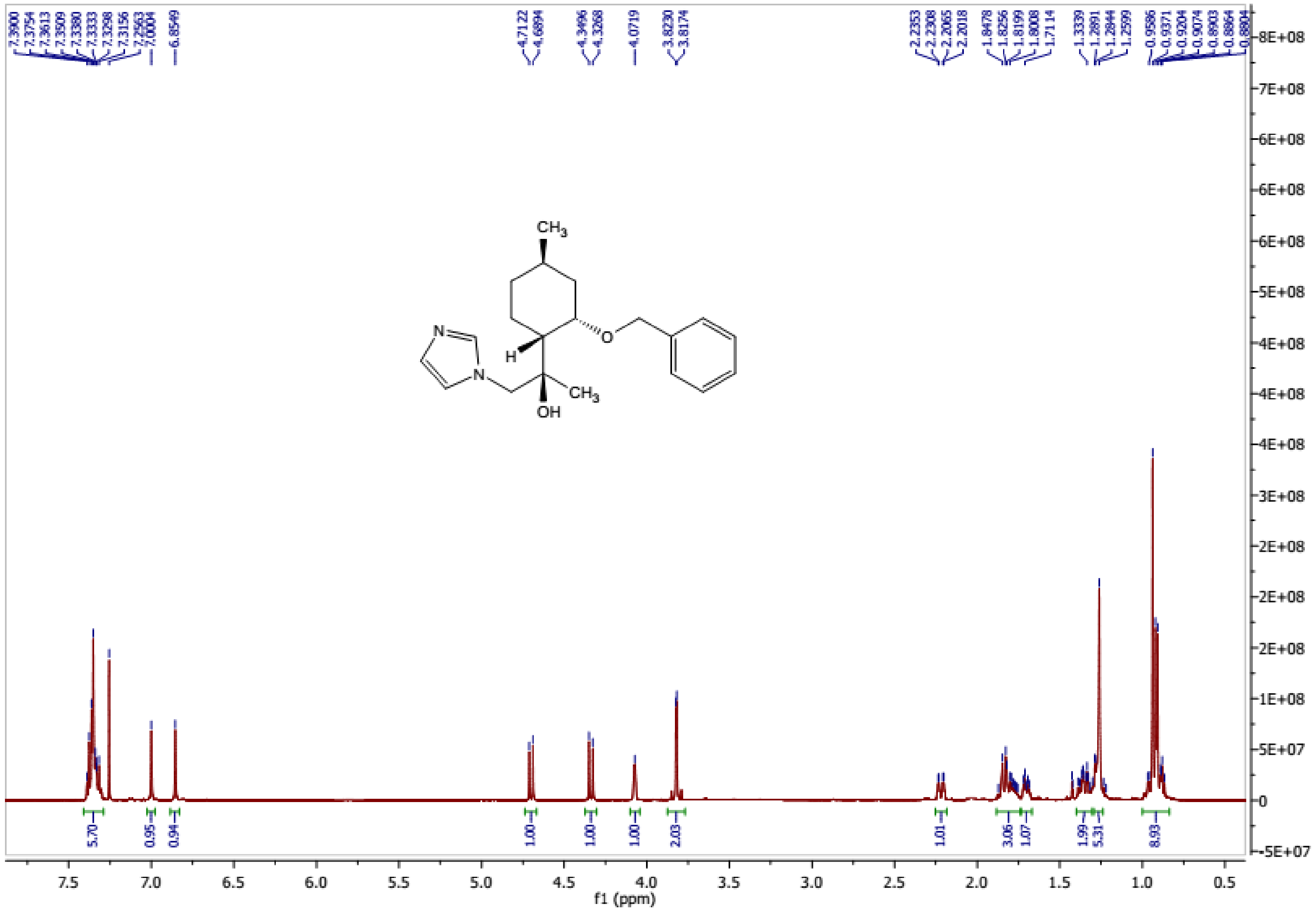

Figure S 15: ^{13}C-NMR of compound **7a**

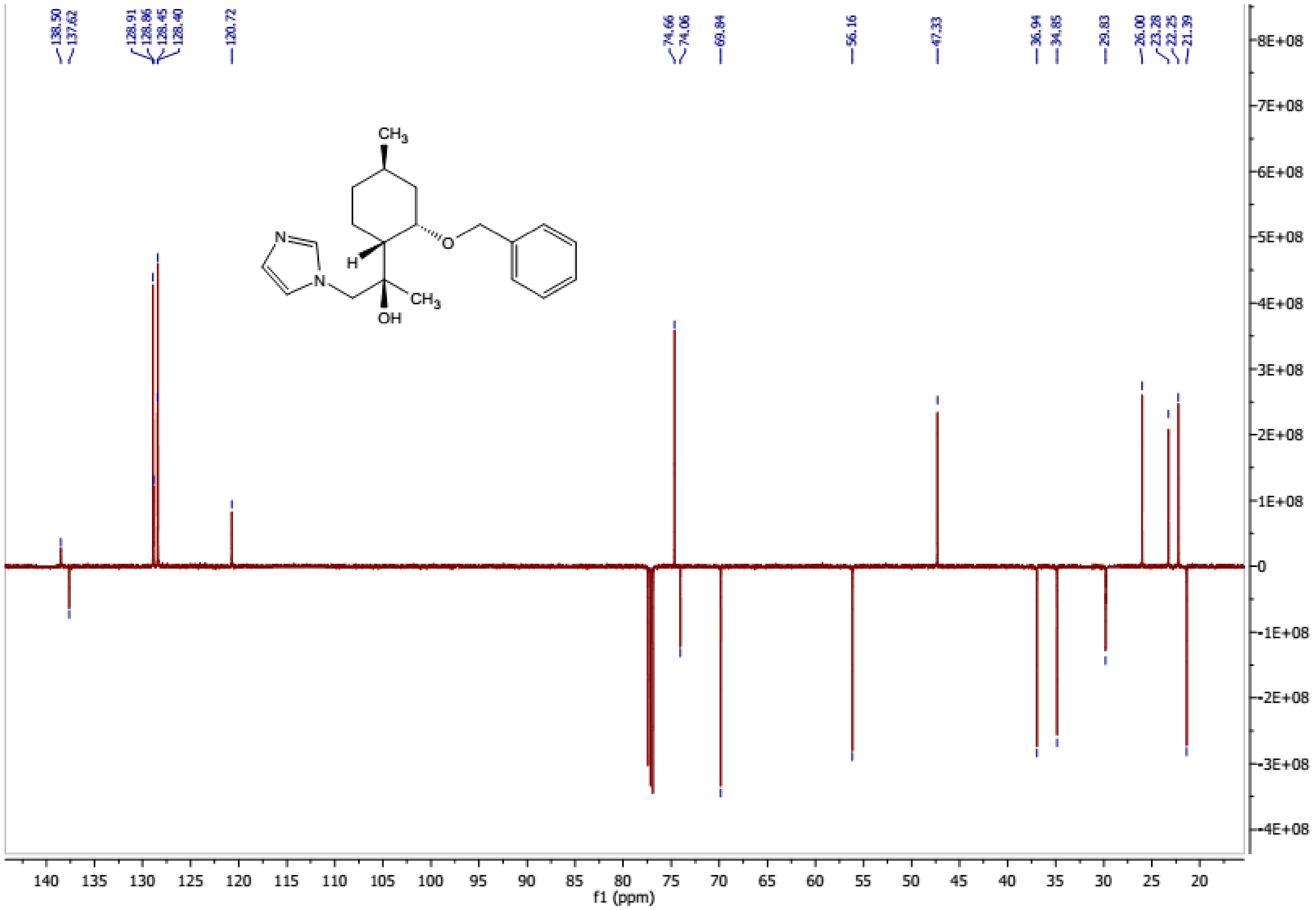

Figure S 16: ¹H-NMR of compound **7b**

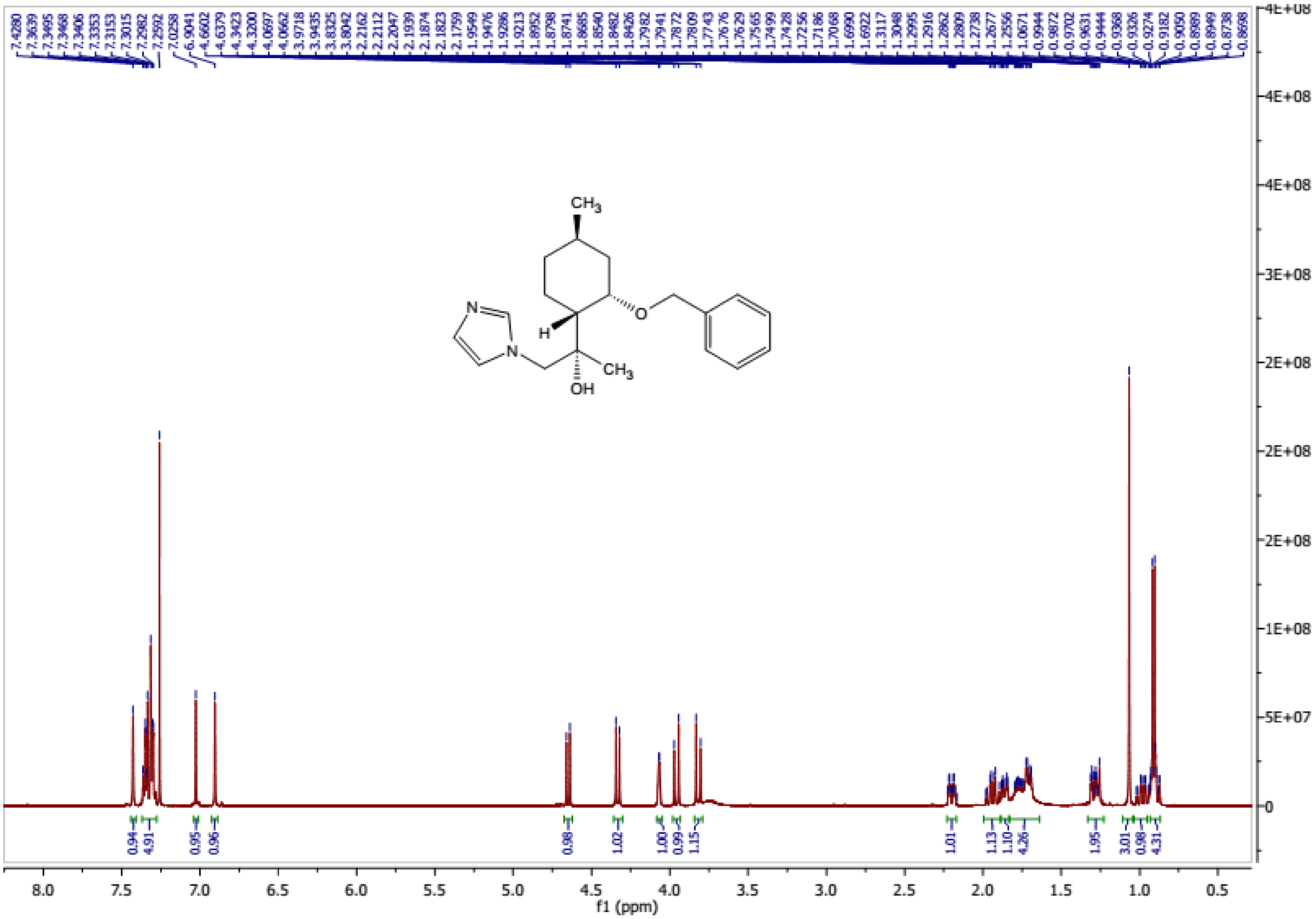

Figure S 17: ^{13}C-NMR of compound **7b**

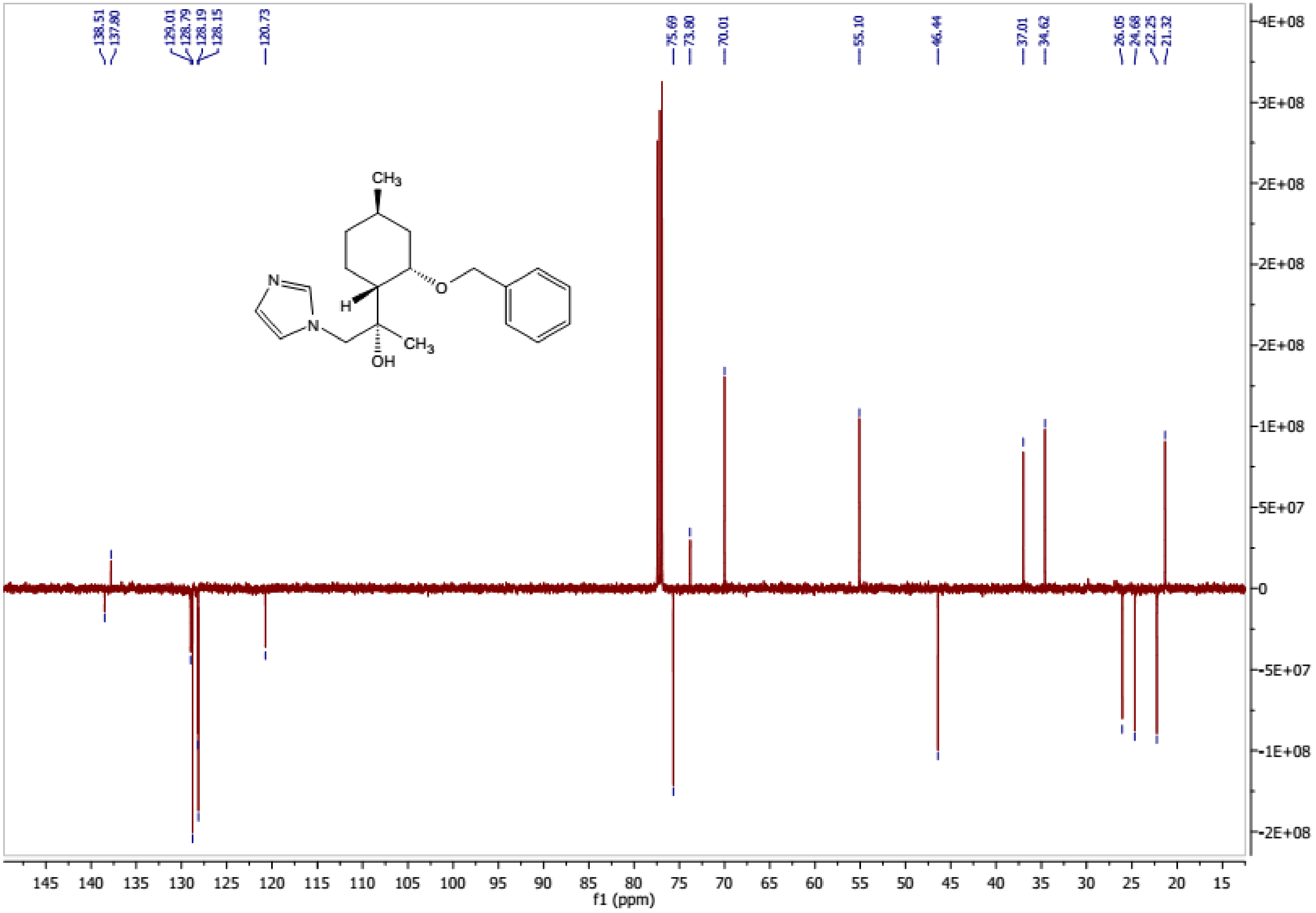

Figure S 18: ^{1}H-NMR of compound **8a**

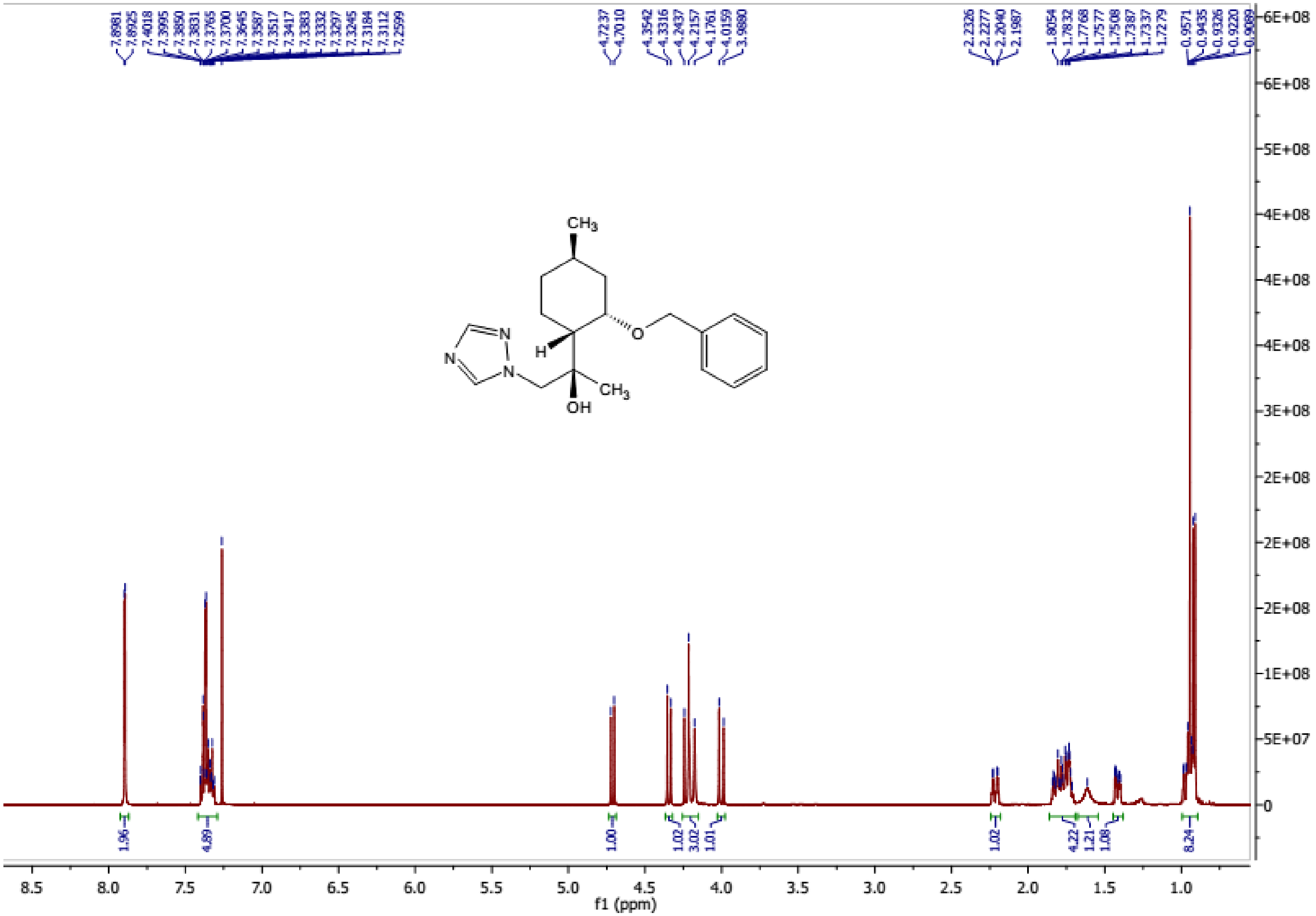

Figure S 19: ¹³C-NMR of compound **8a**

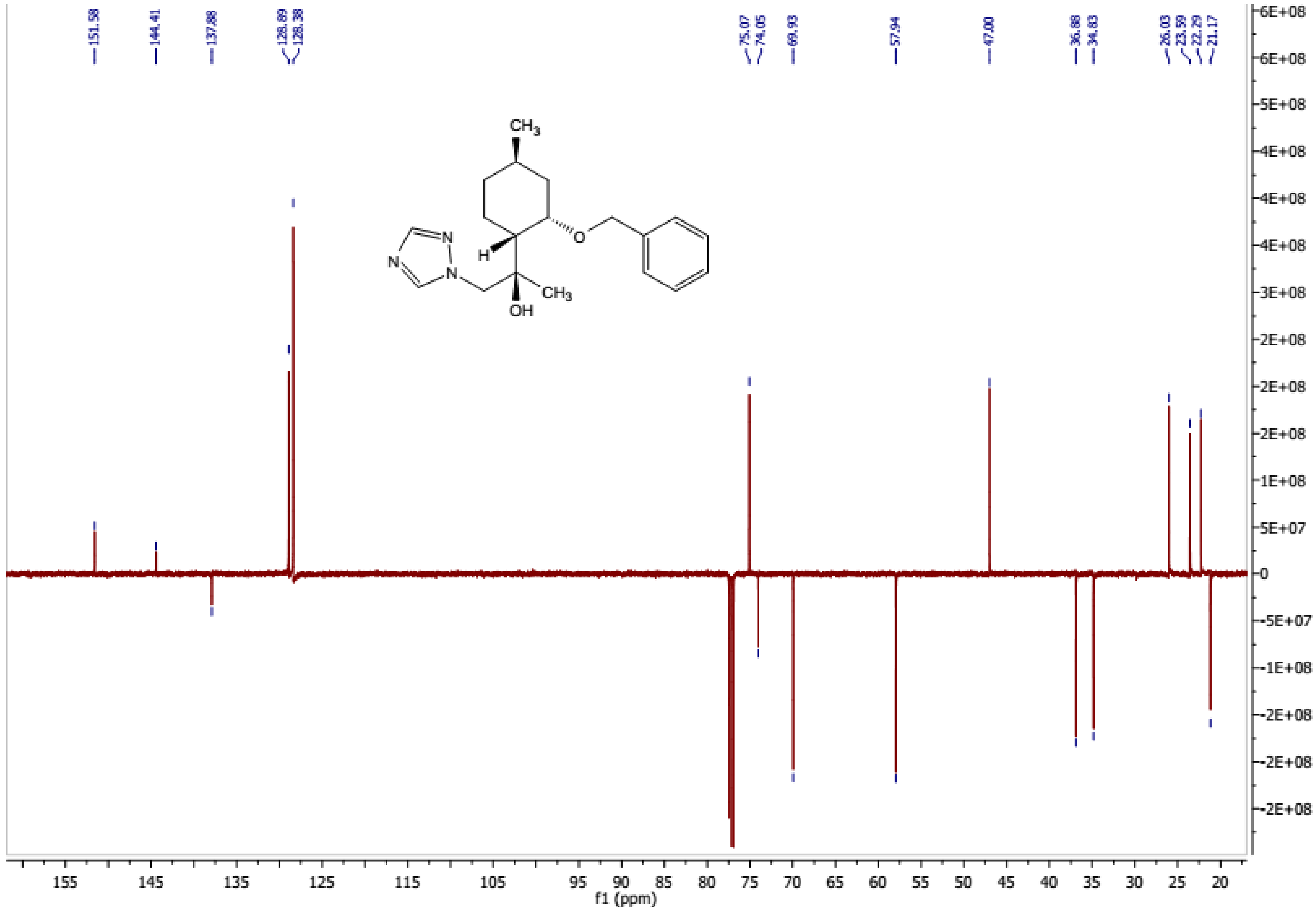

Figure S 20: ^{1}H-NMR of compound **8b**

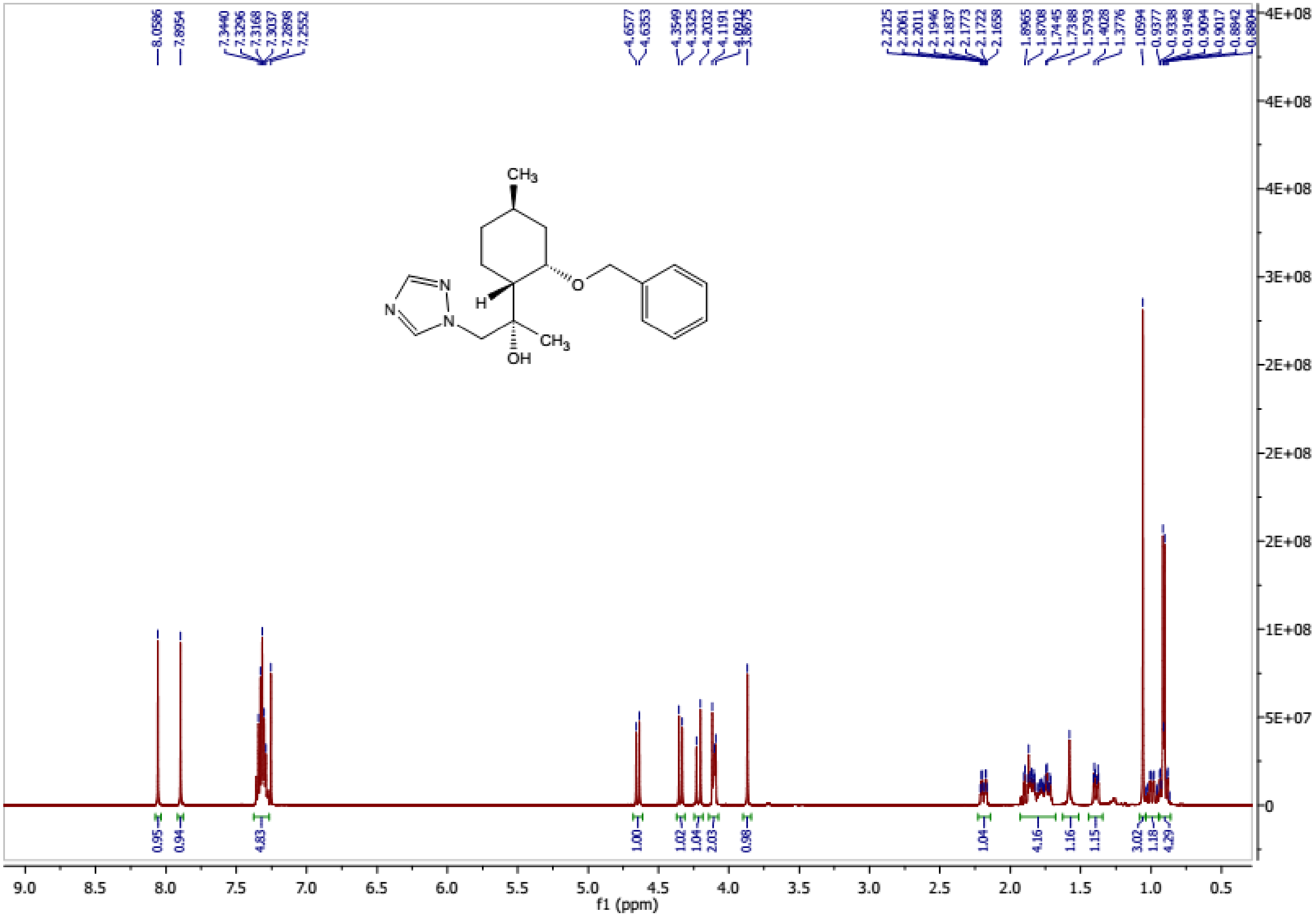

Figure S 21: ^{13}C-NMR of compound **8b**

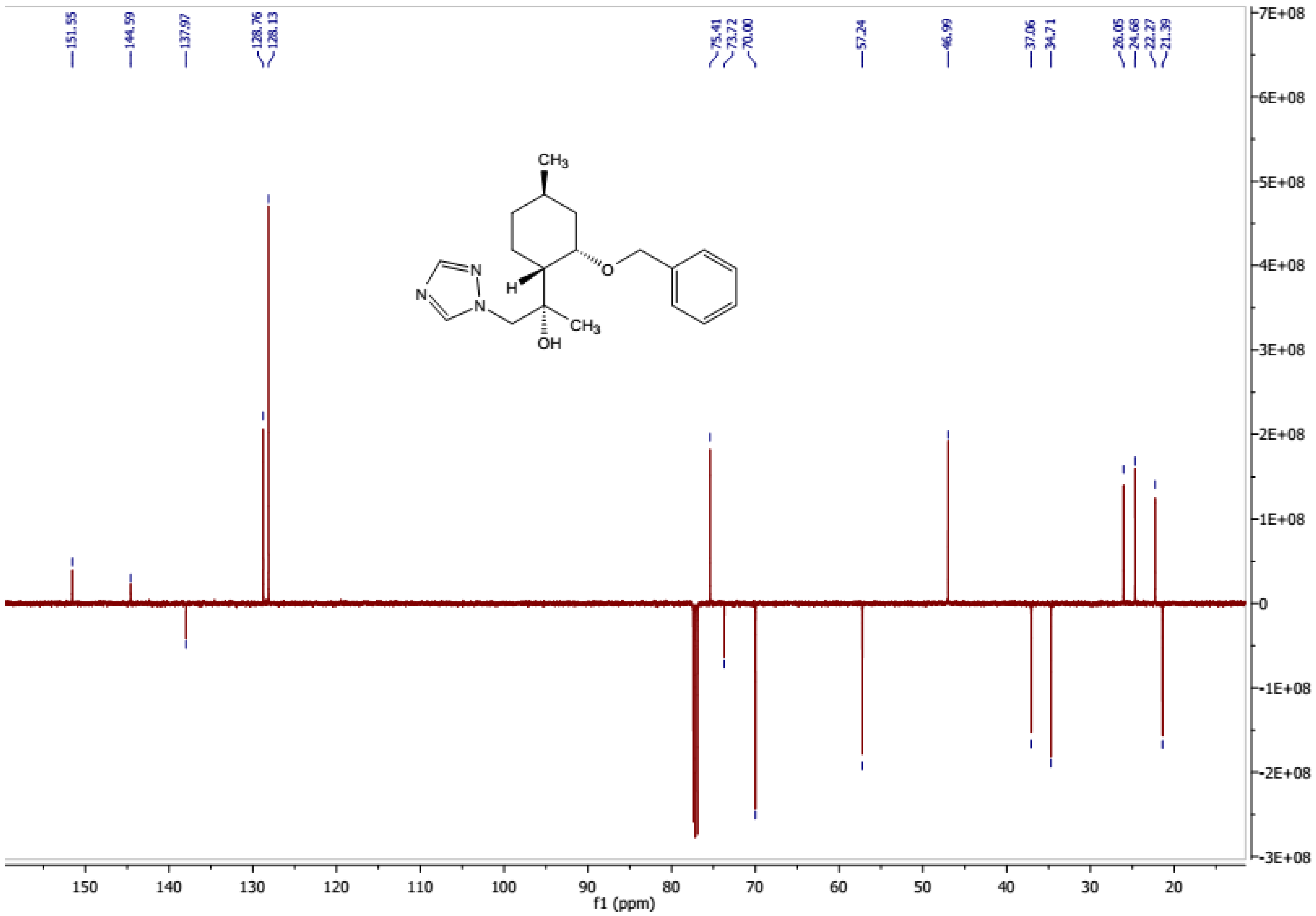

Figure S 22: ¹H-NMR of compound **9a**

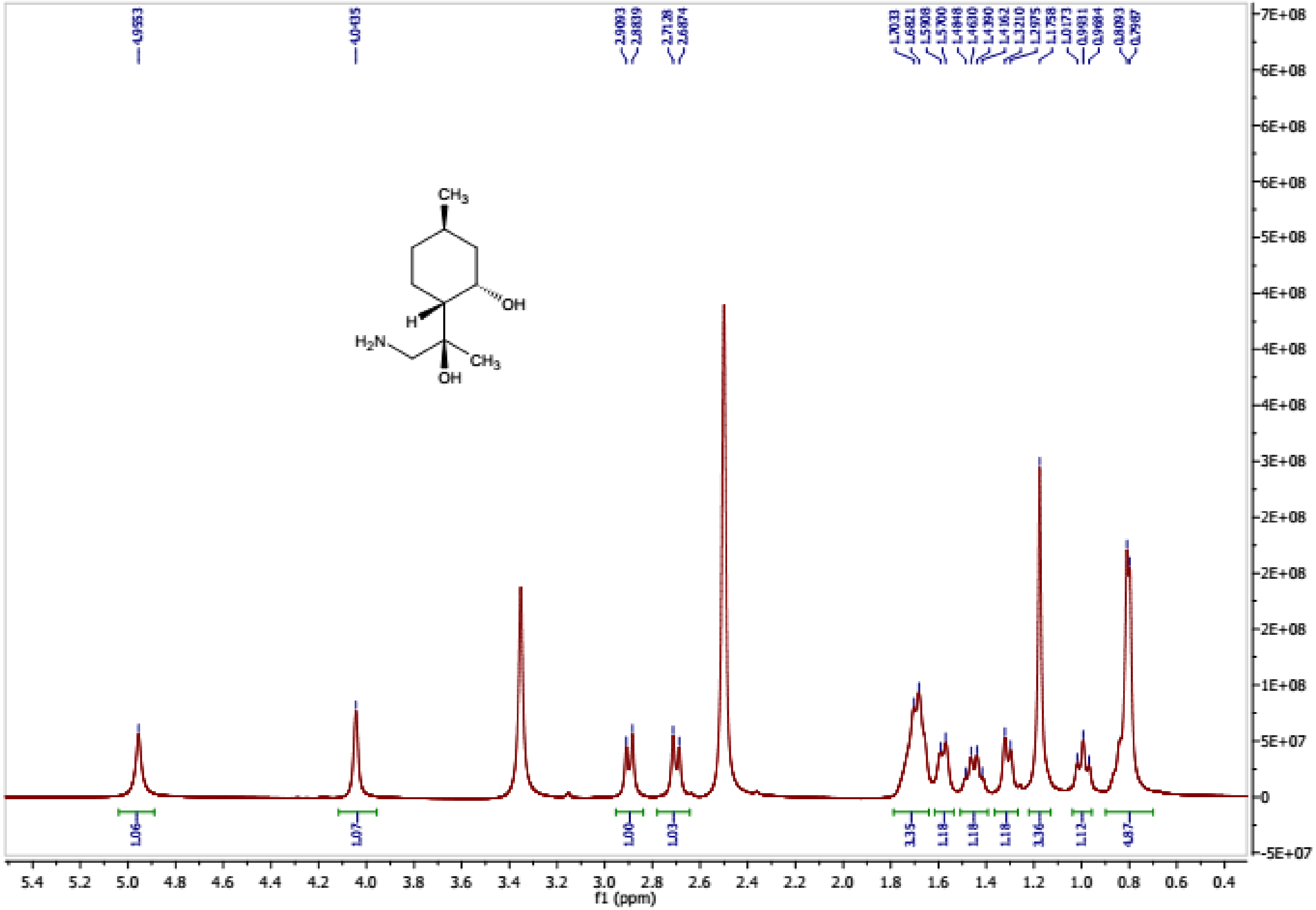

Figure S 23: ^{13}C-NMR of compound **9a**

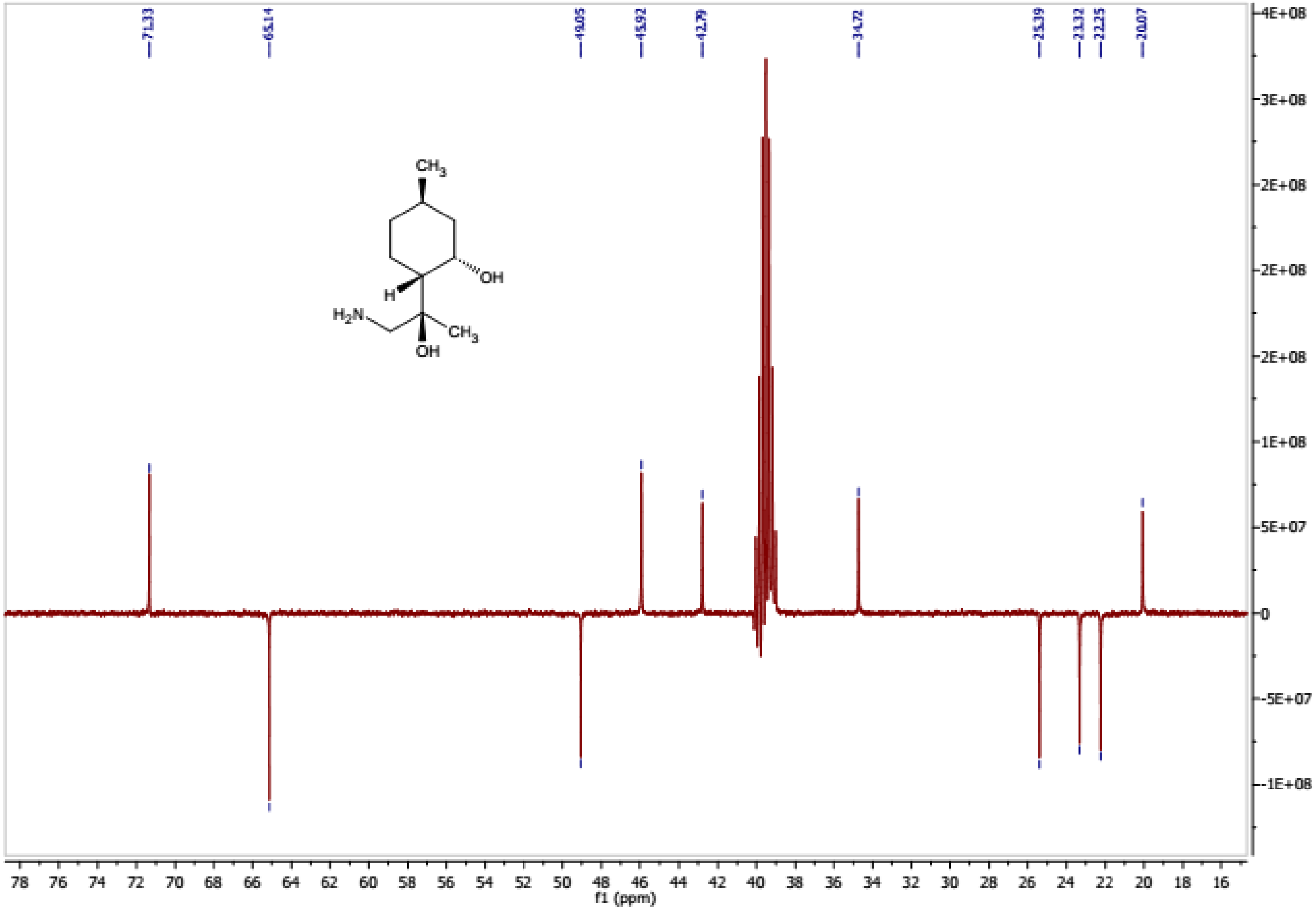

Figure S 24: ^{1}H-NMR of compound **9b**

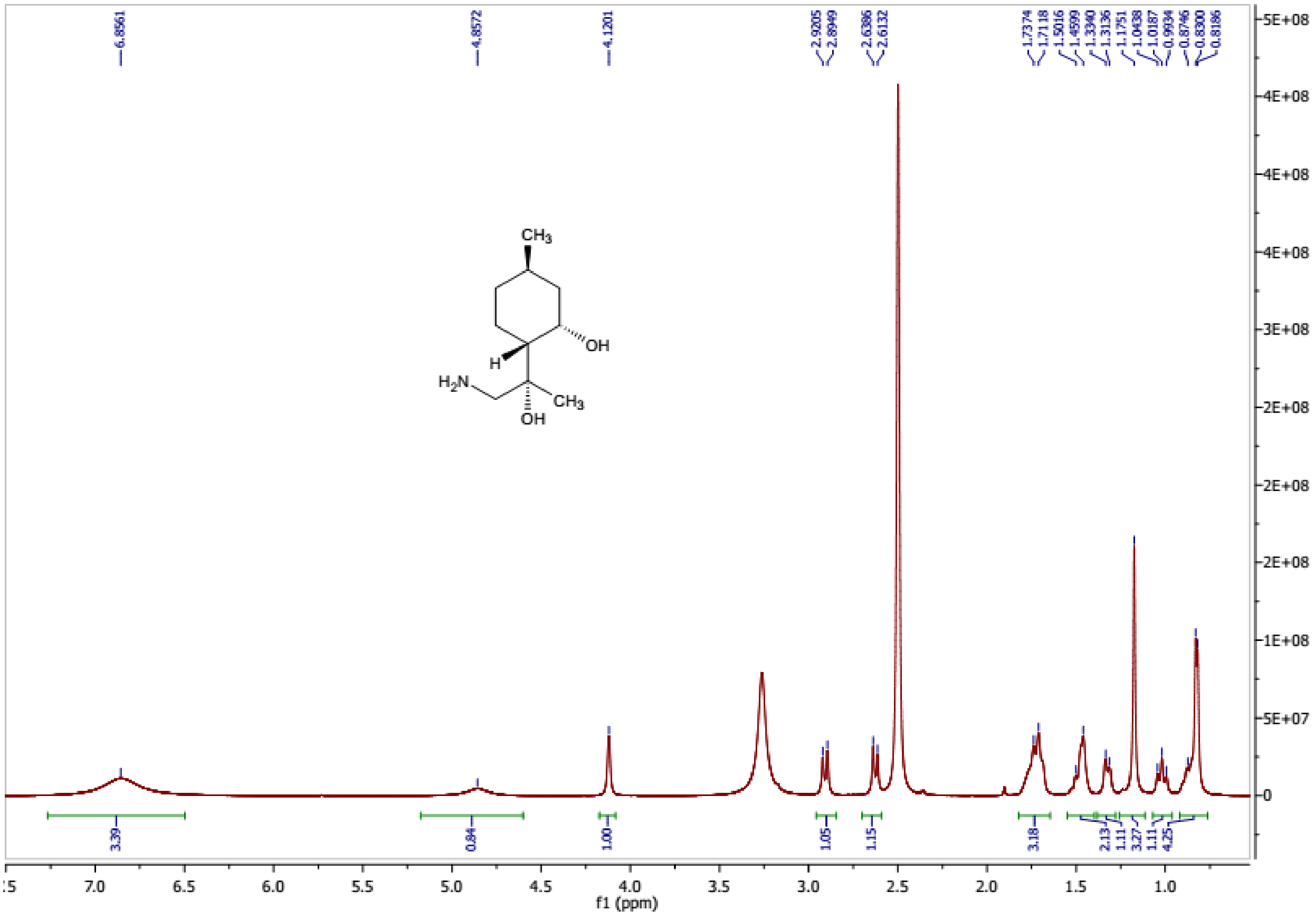

Figure S 25: ^{13}C-NMR of compound **9b**

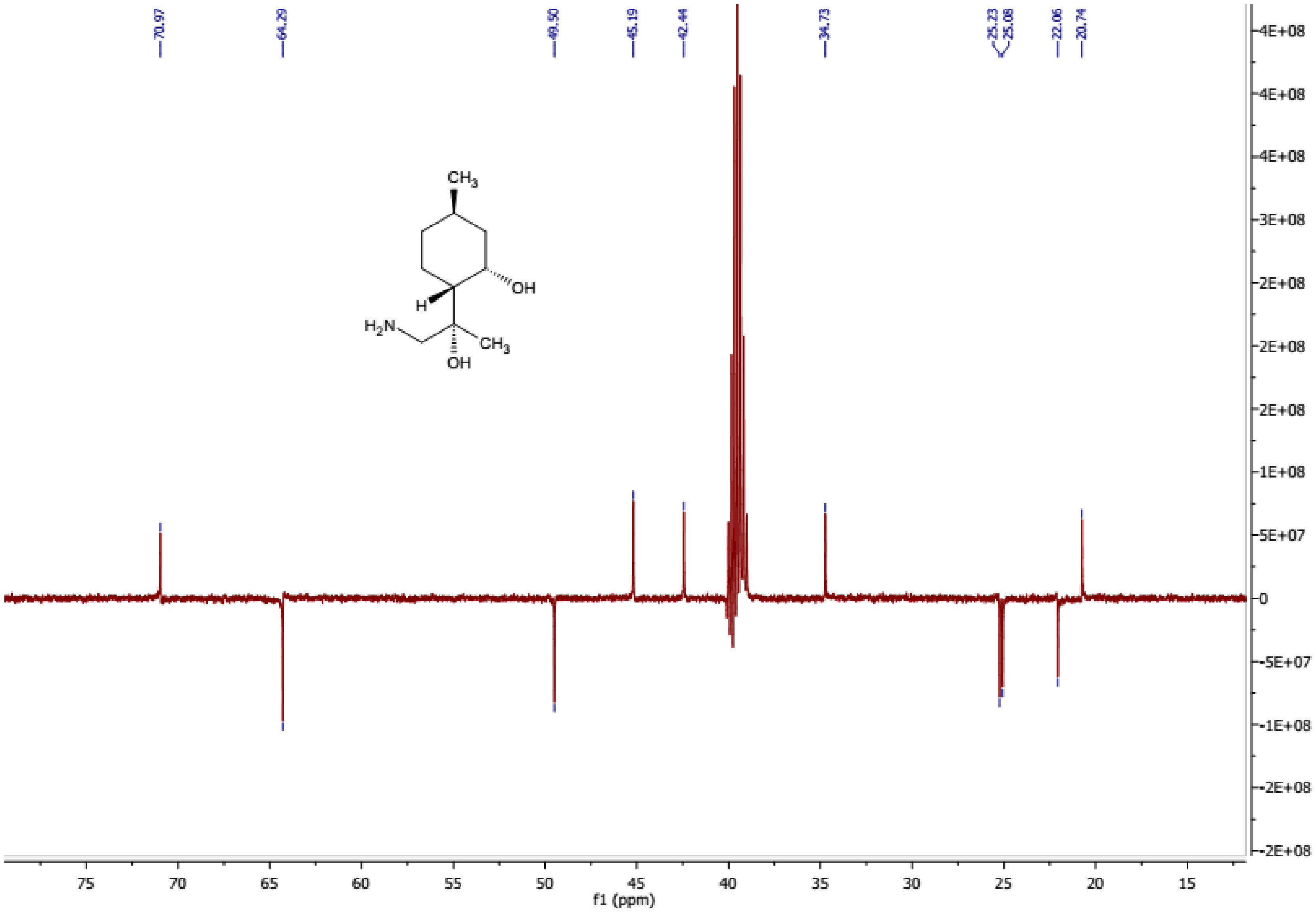

Figure S 26: ^{1}H-NMR of compound **10**

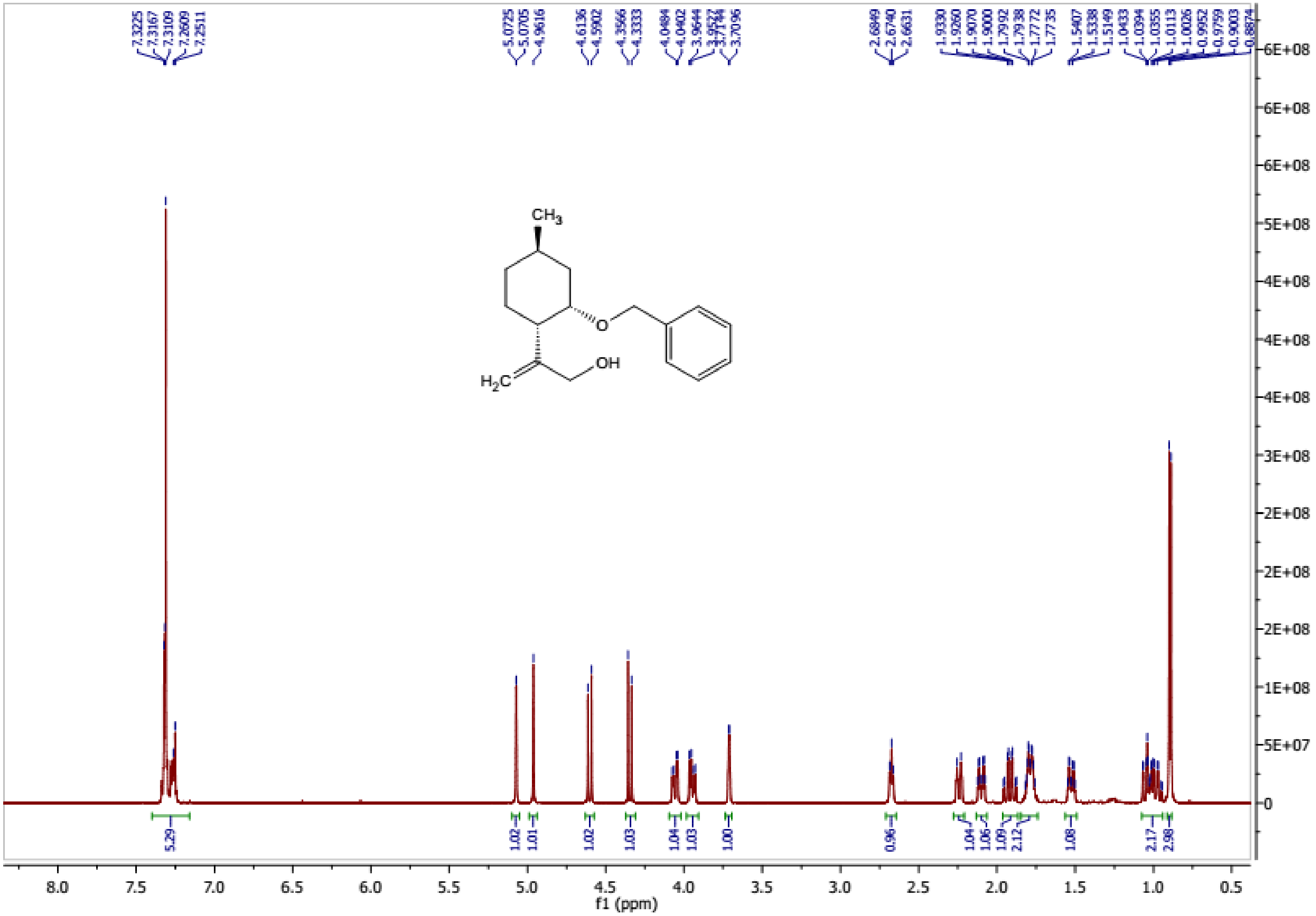

Figure S 27: ^{13}C-NMR of compound **10**

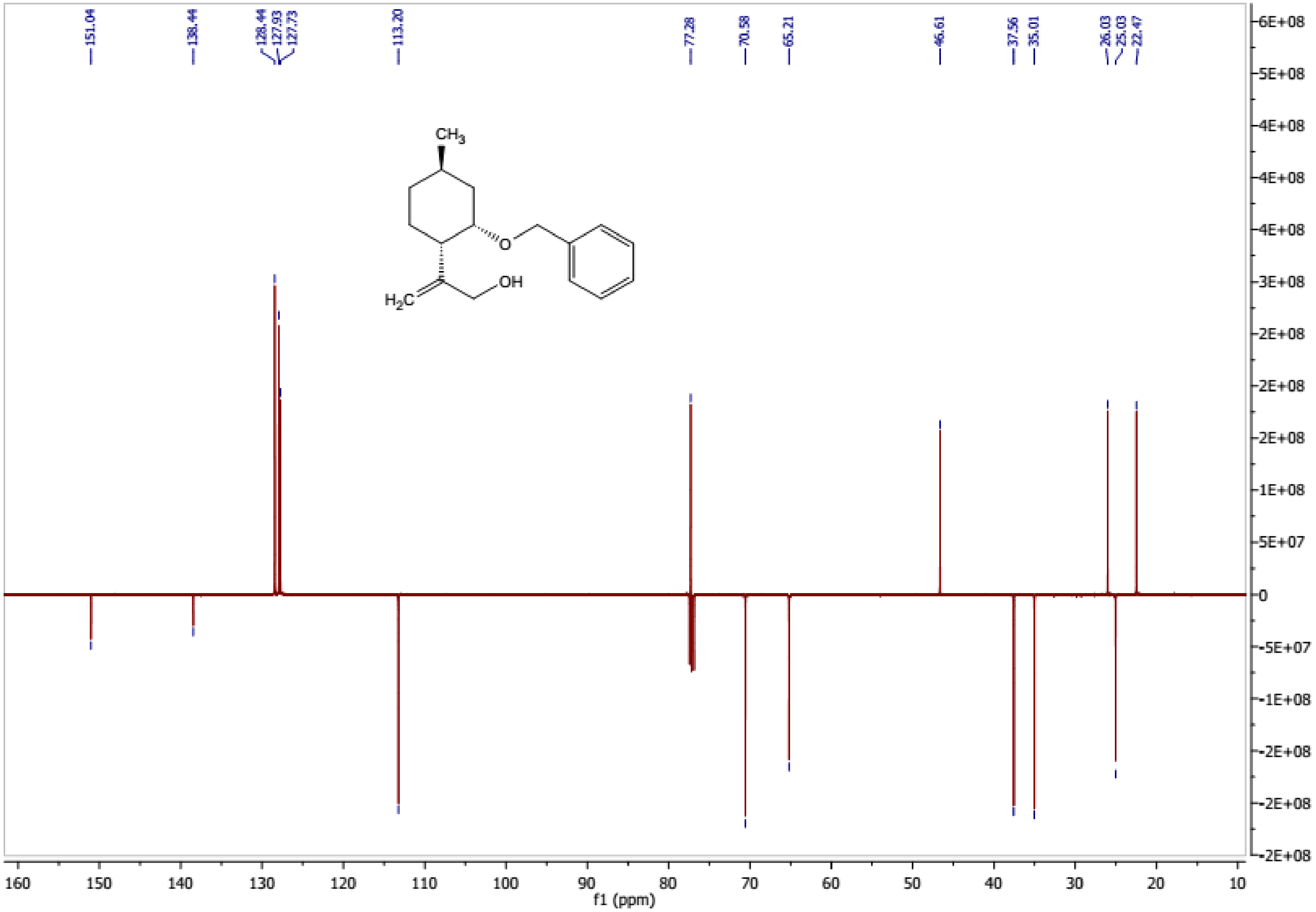

Figure S 28: 1H-NMR of compound **12a**

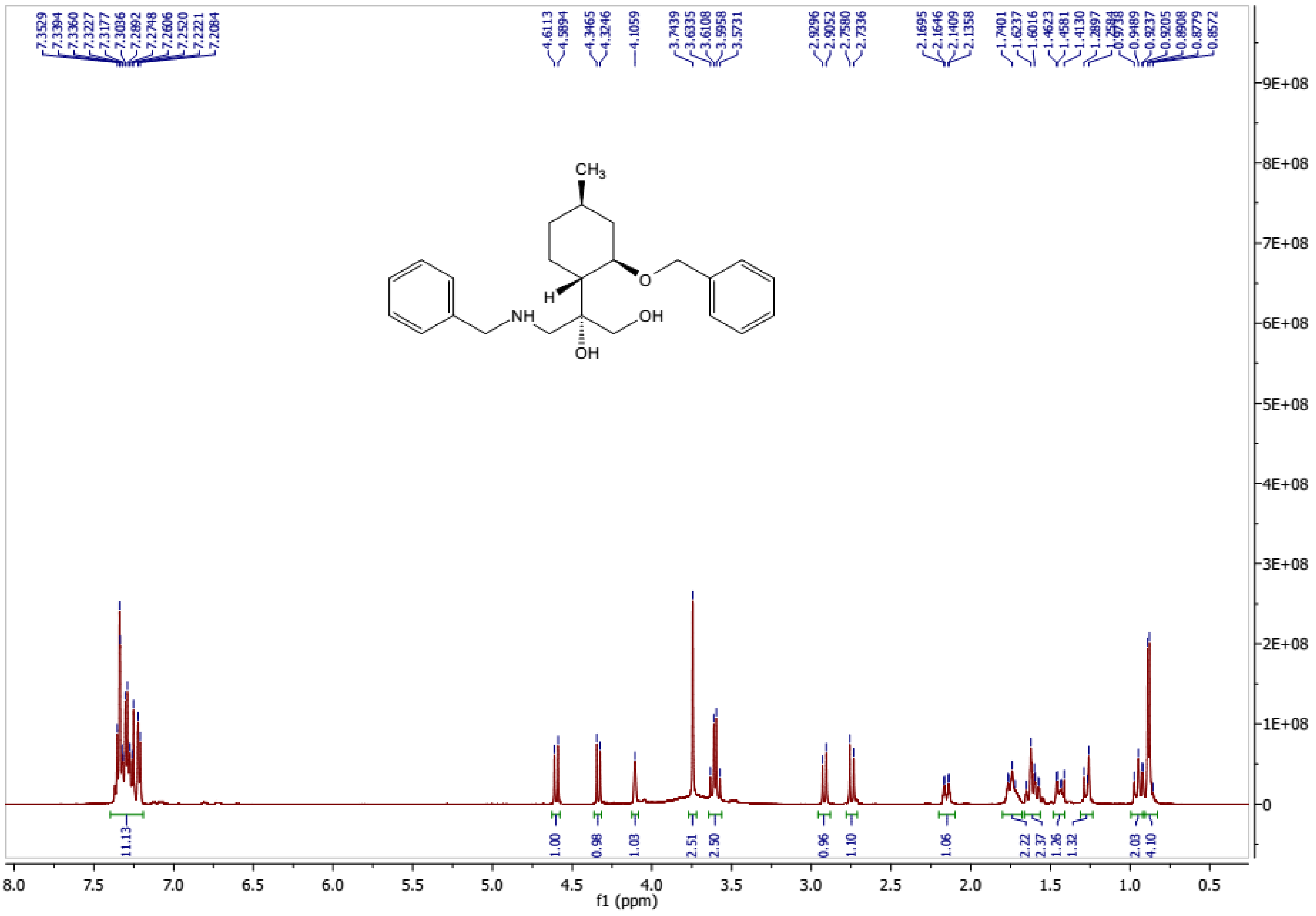

Figure S 29: ^{13}C-NMR of compound **12a**

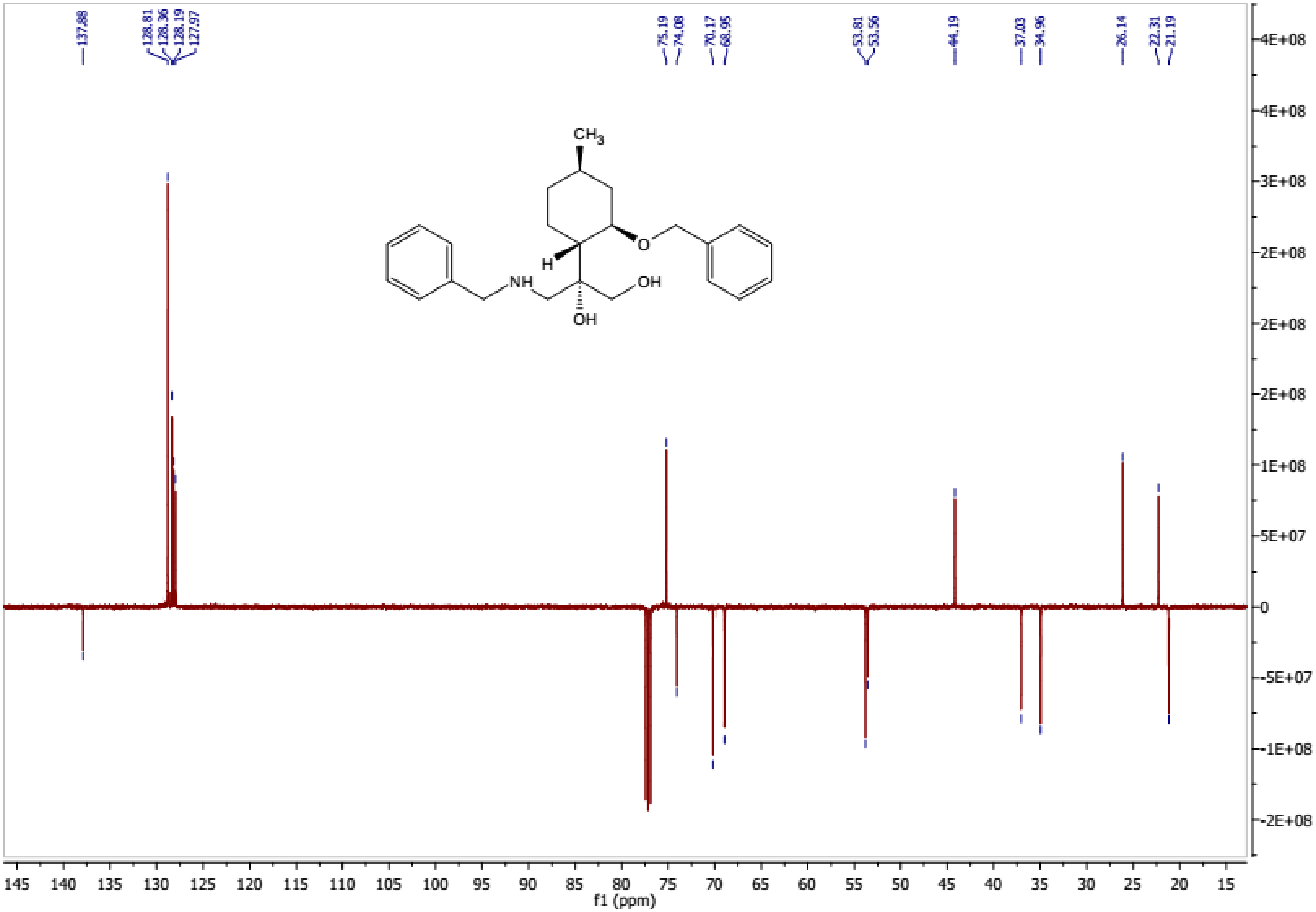

Figure S 30: ^{1}H-NMR of compound **12b**

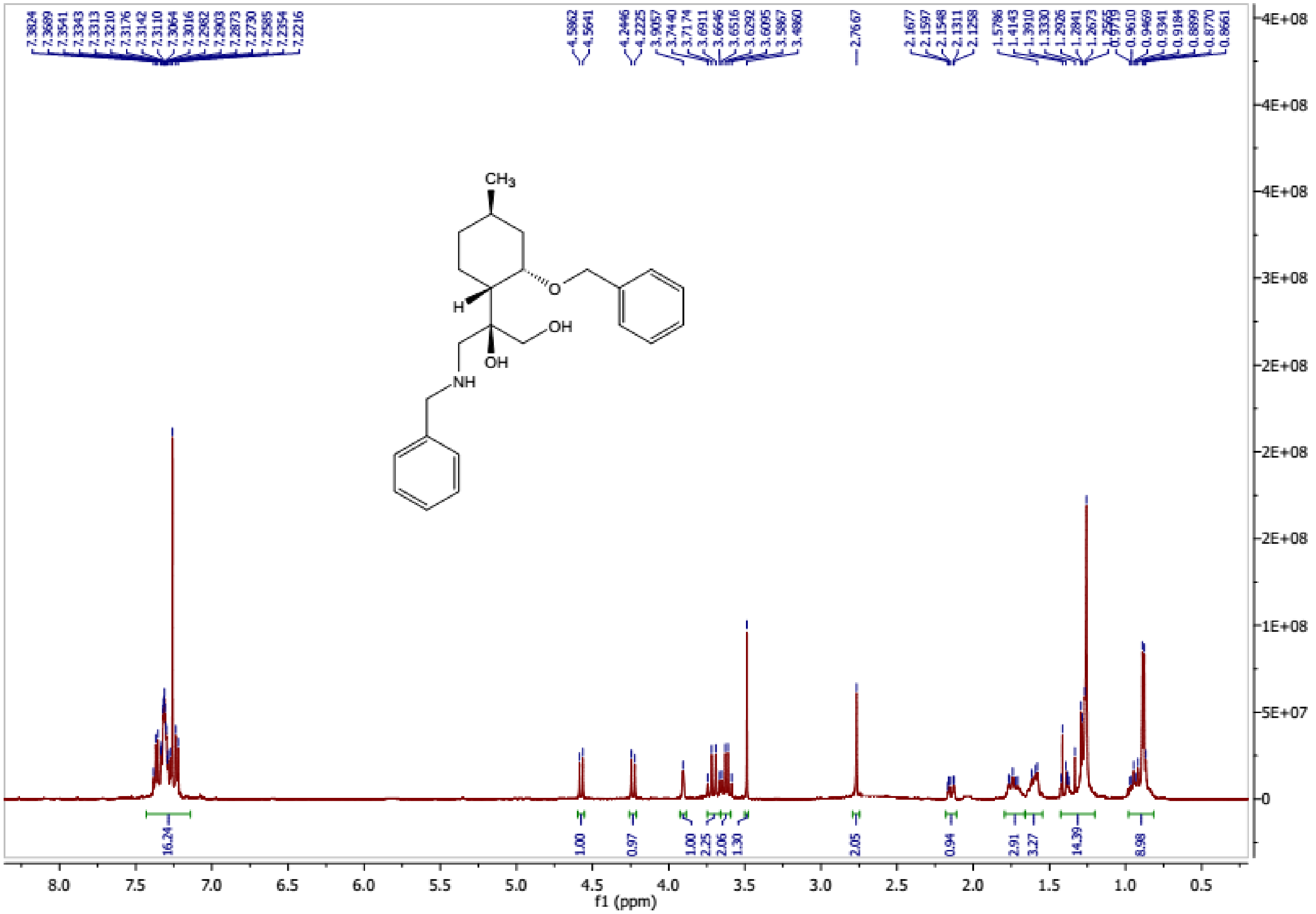

Figure S 31: ^{13}C-NMR of compound **12b**

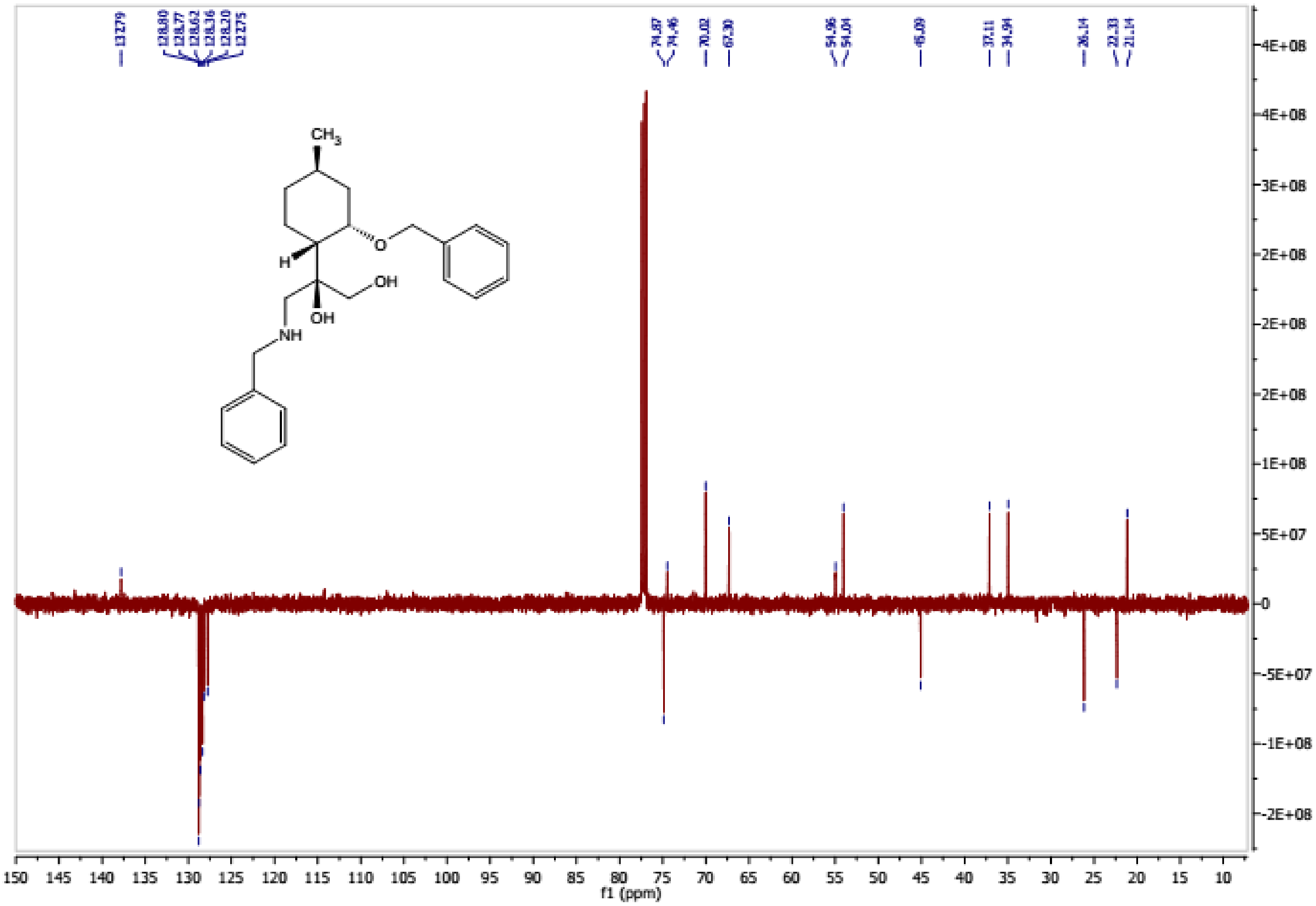

Figure S 32: ^{1}H-NMR of compound **13a**

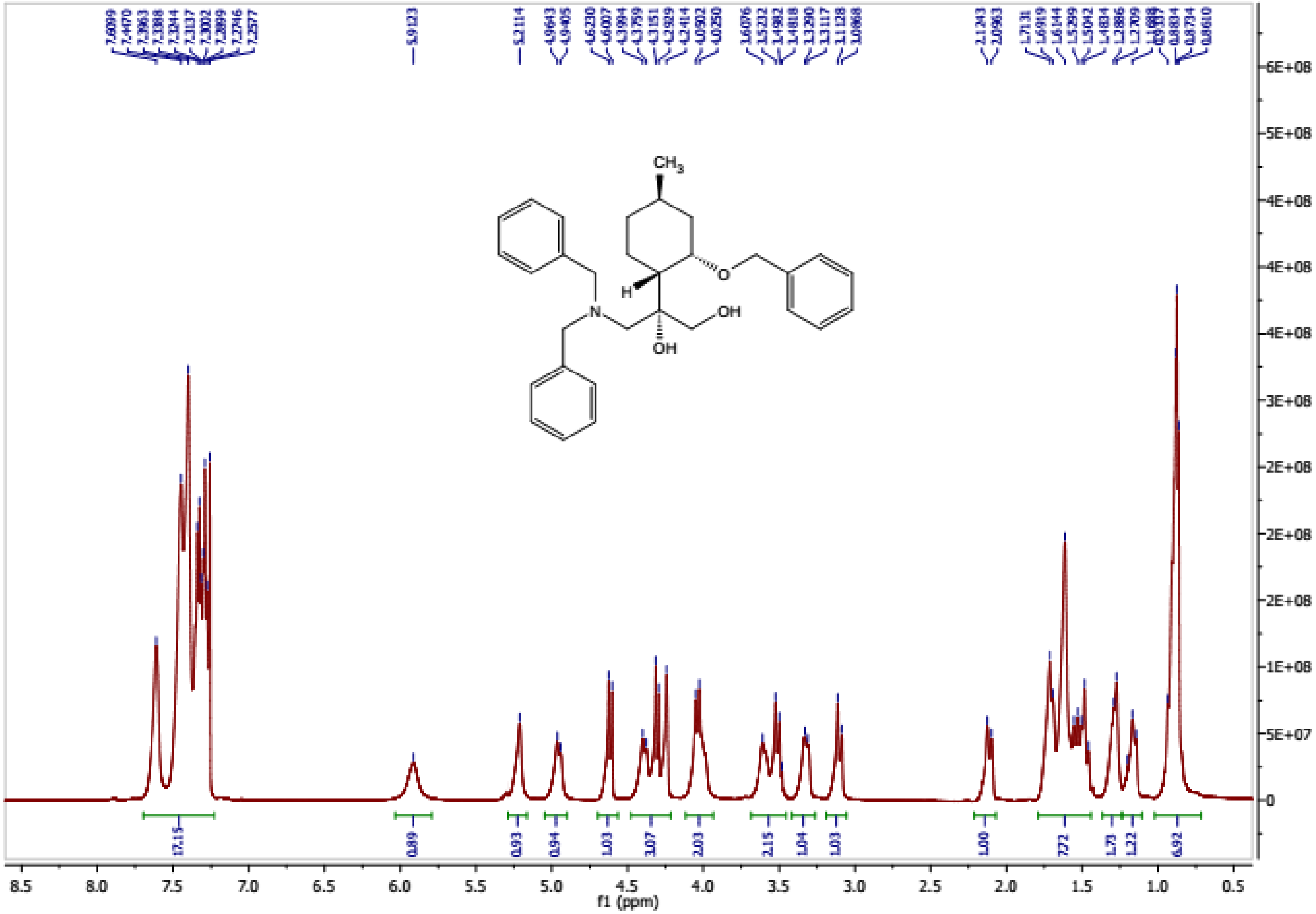

Figure S 33: ^{13}C-NMR of compound **13a**

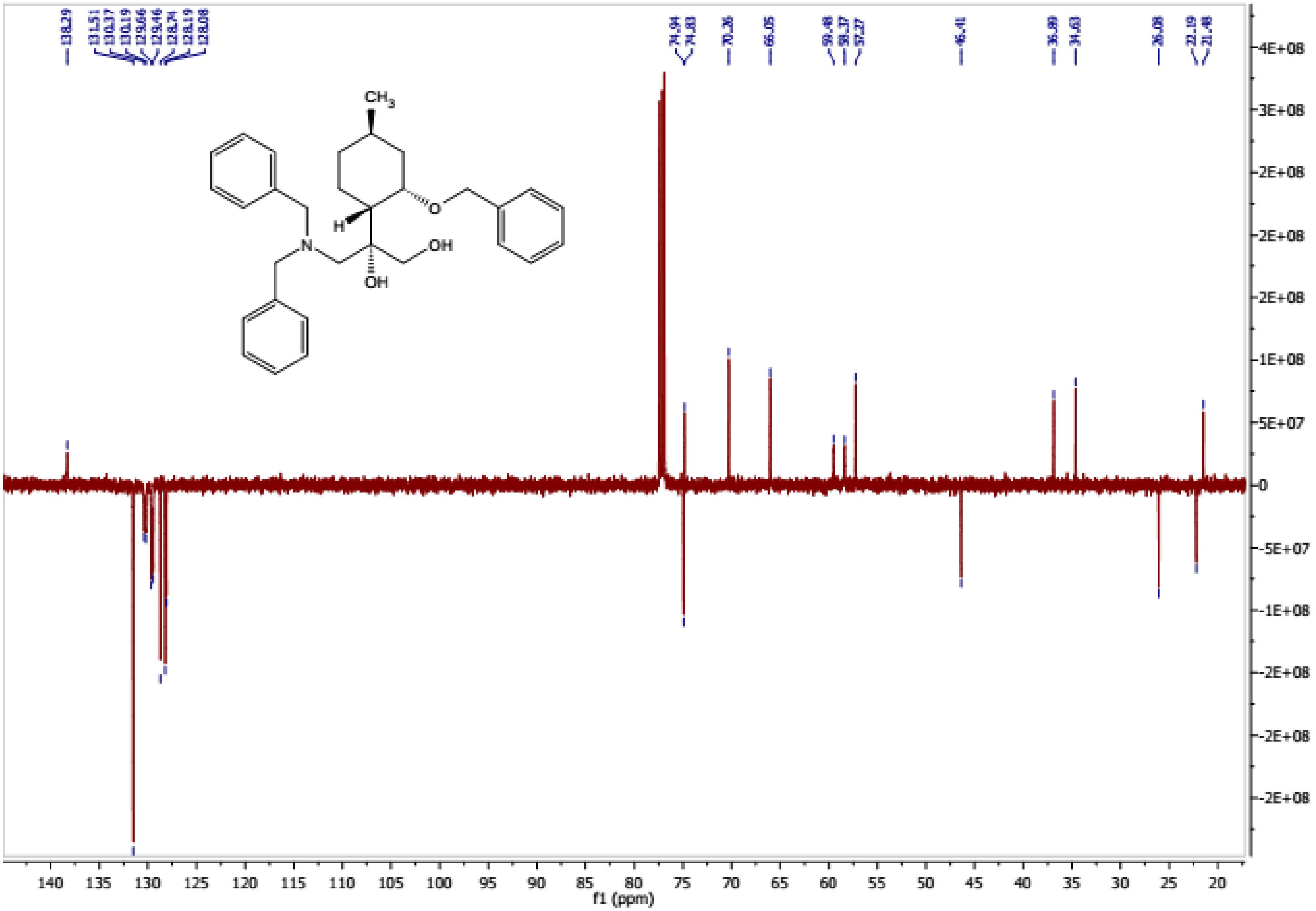

Figure S 34: ^{1}H-NMR of compound **13b**

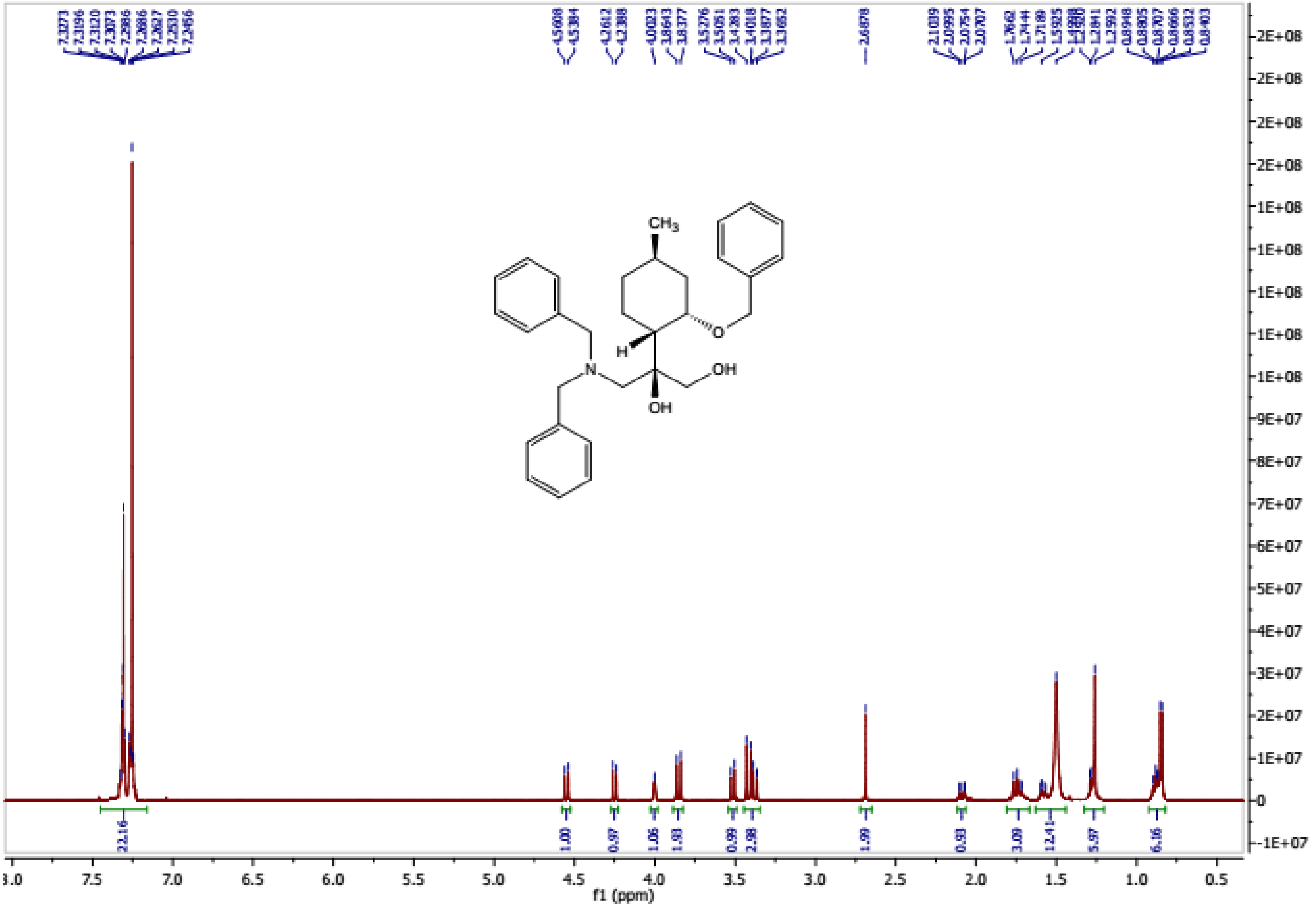

Figure S 35: ^{13}C-NMR of compound **13b**

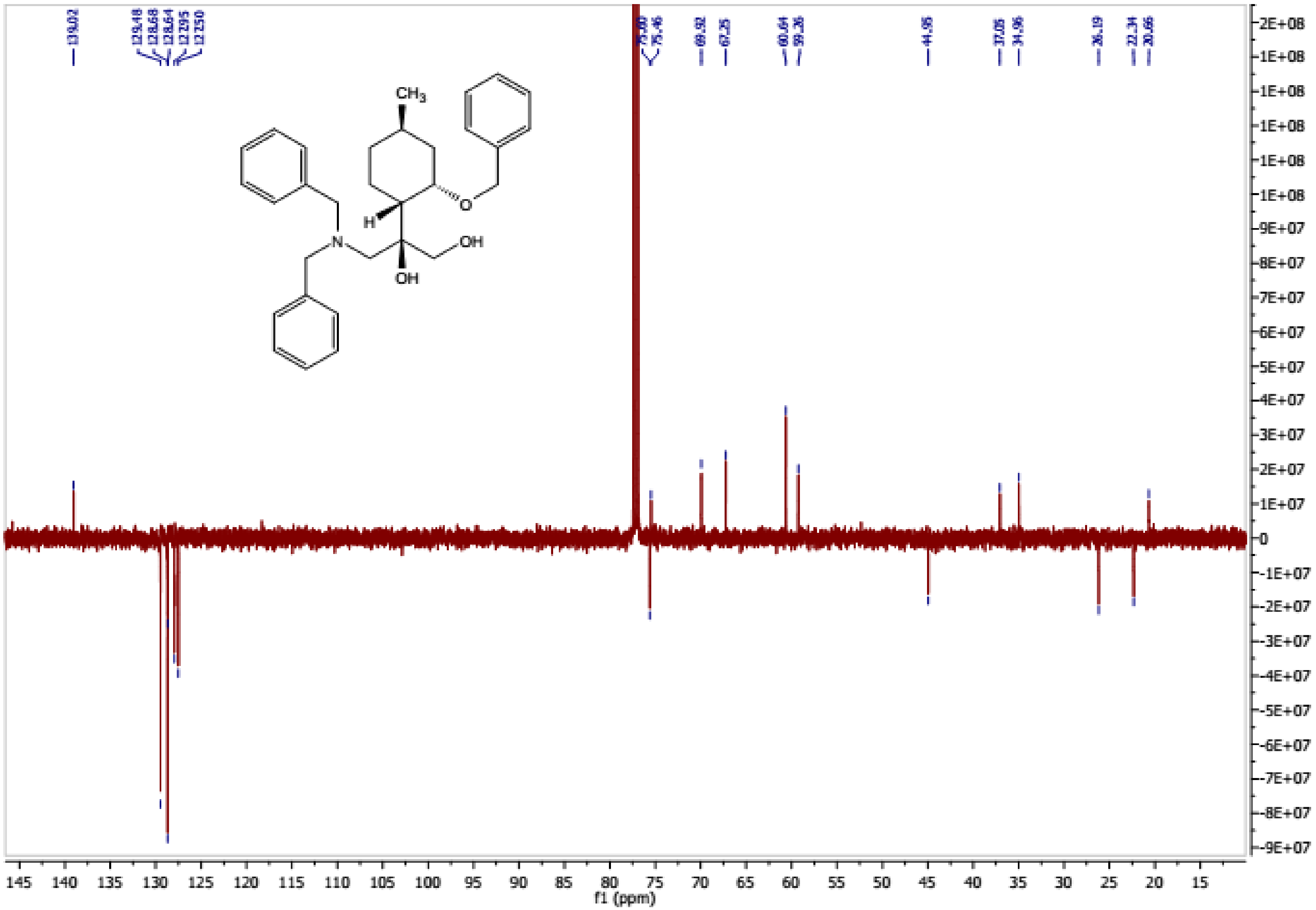

Figure S 36: ^{1}H-NMR of compound **14a**

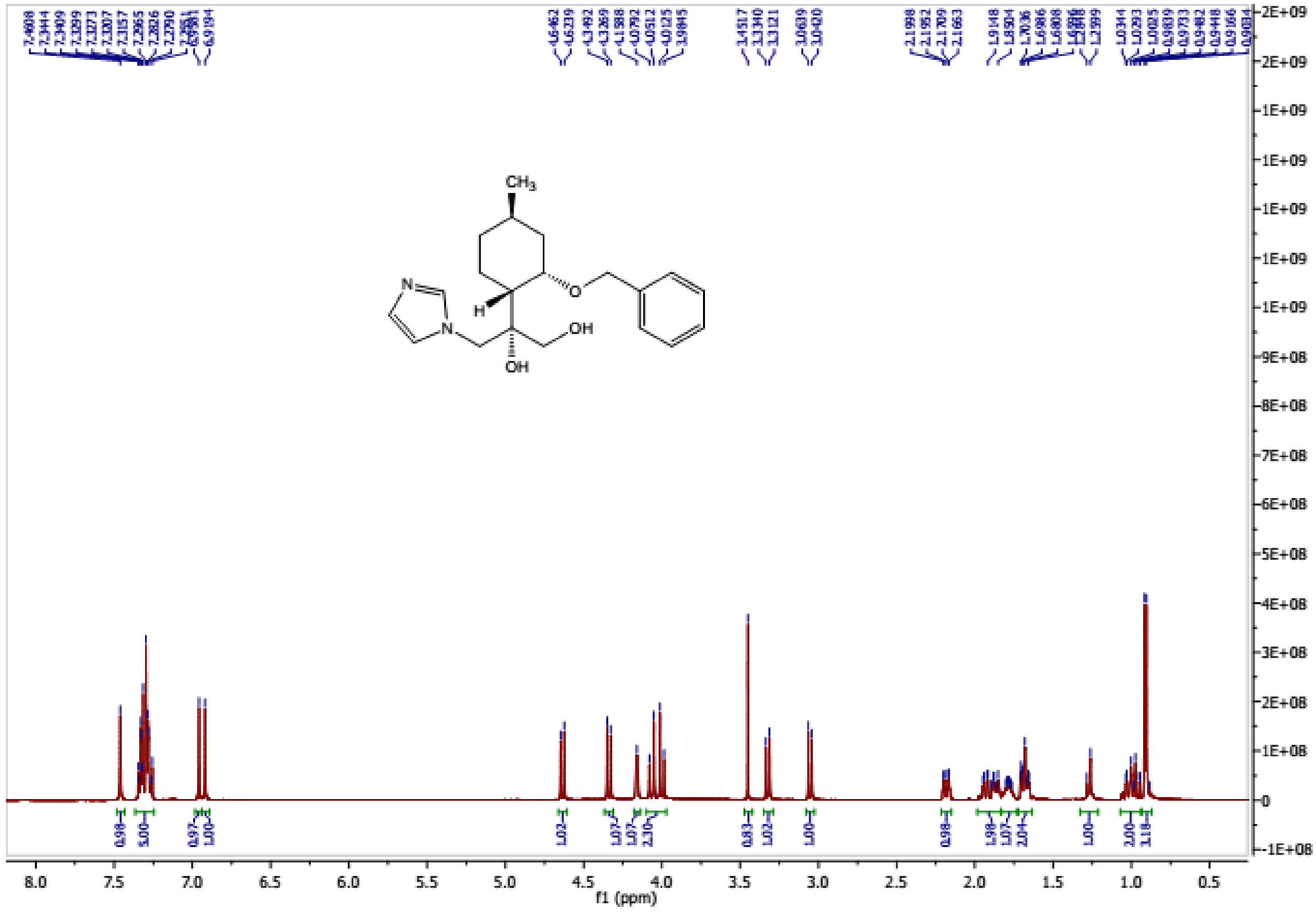

Figure S 37: ^{13}C-NMR of compound **14a**

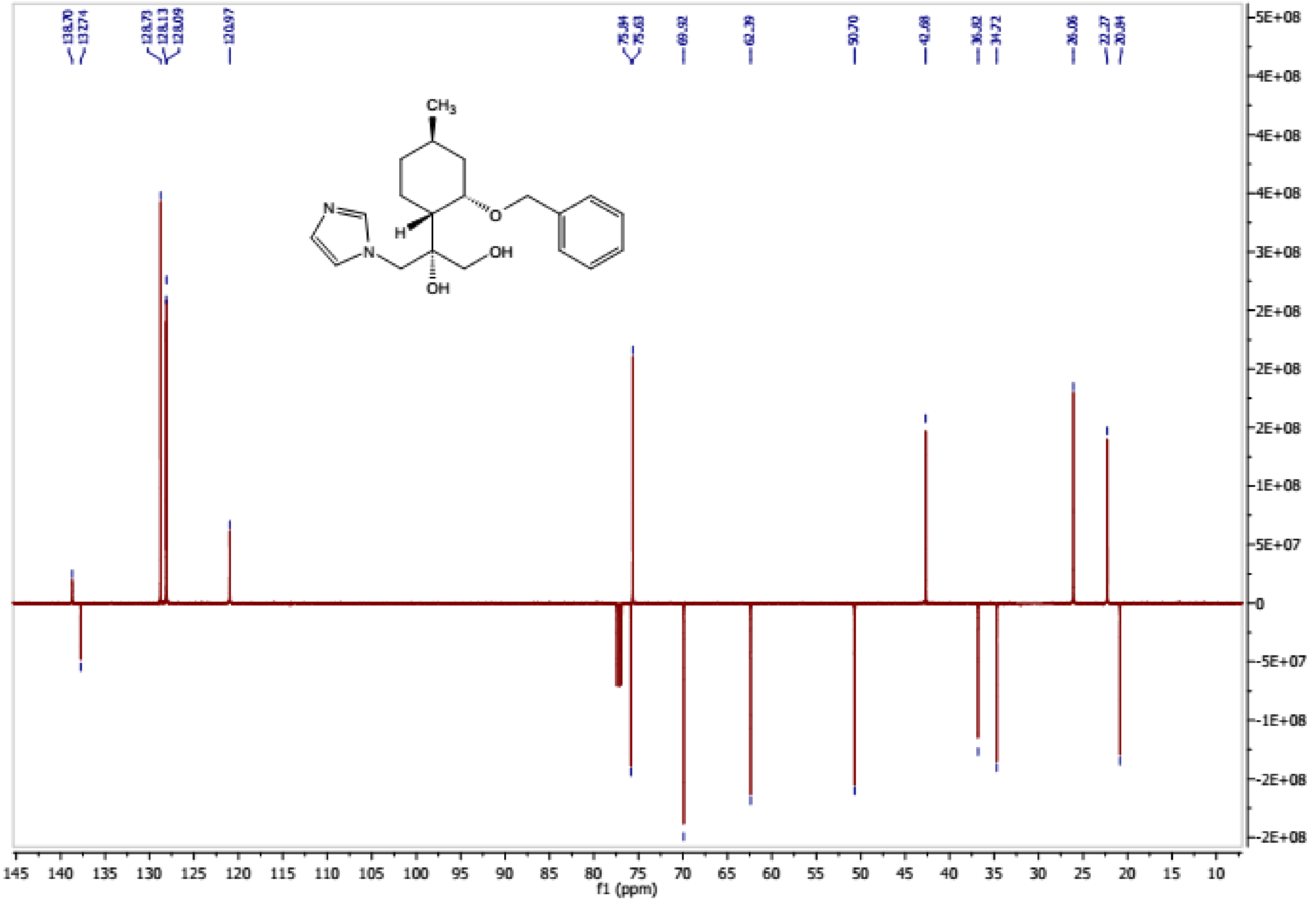

Figure S 38: ^{1}H-NMR of compound **15a**

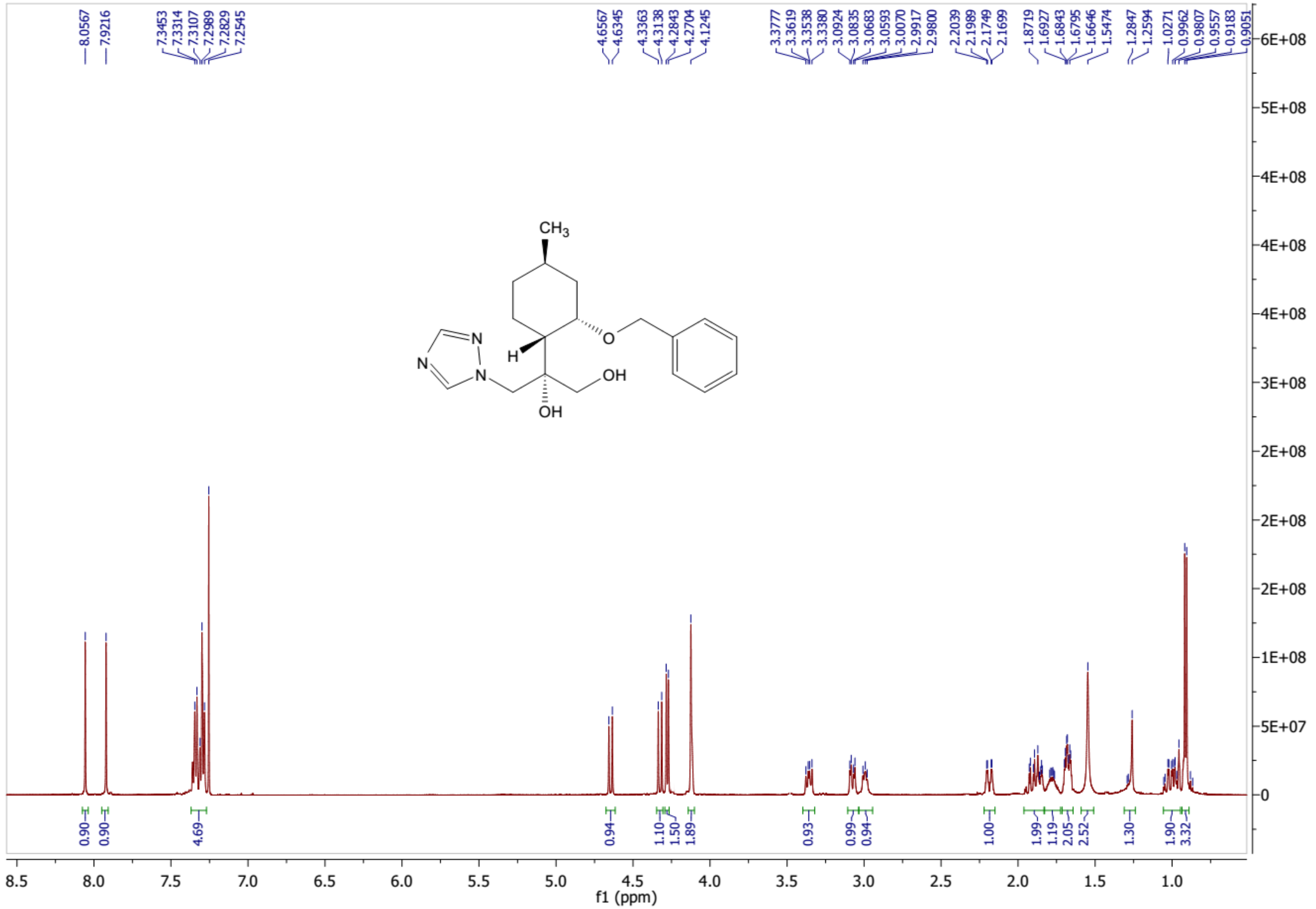

Figure S 39: ^{13}C-NMR of compound **15a**

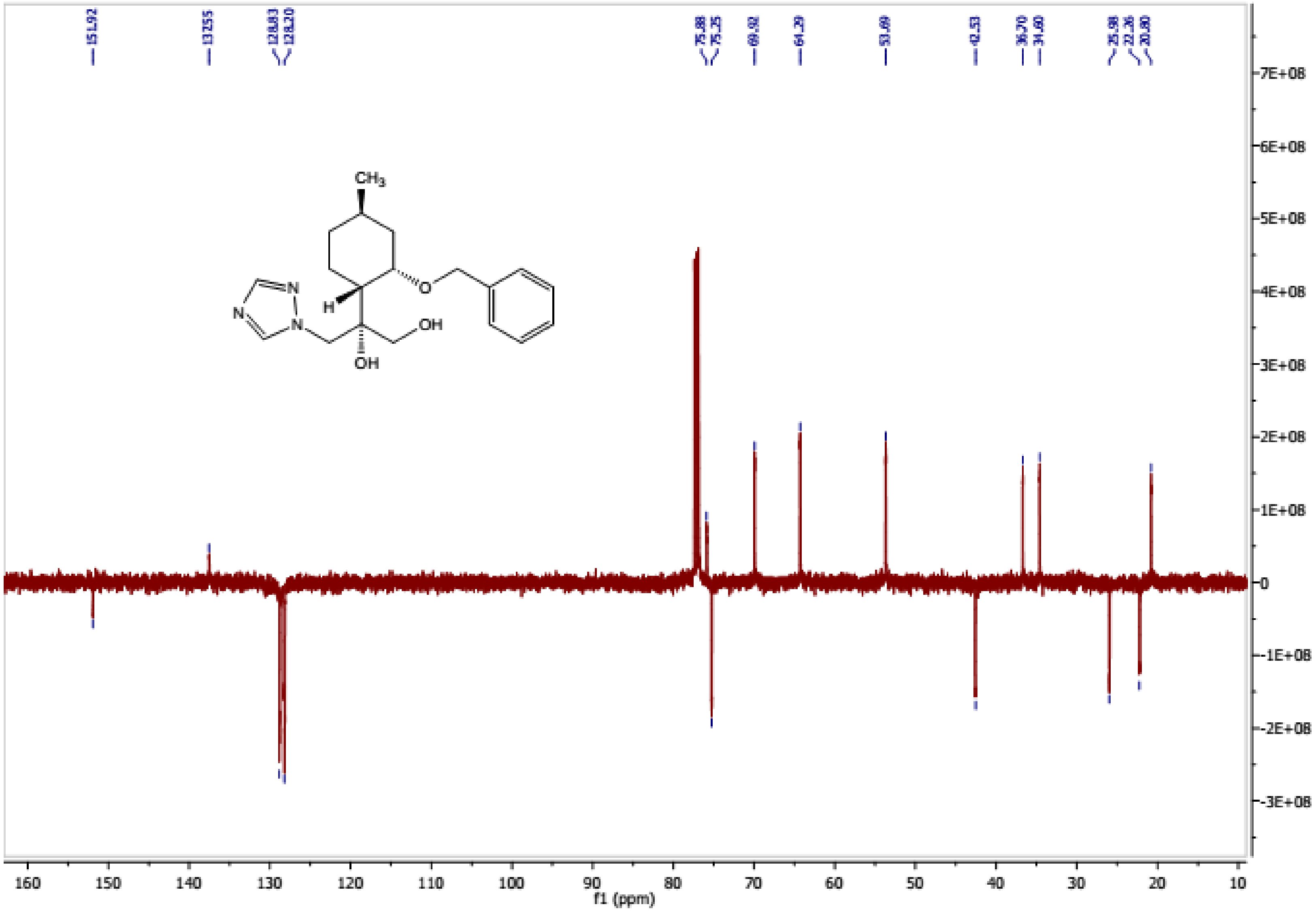

Figure S 40: ^{1}H-NMR of compound **16a**

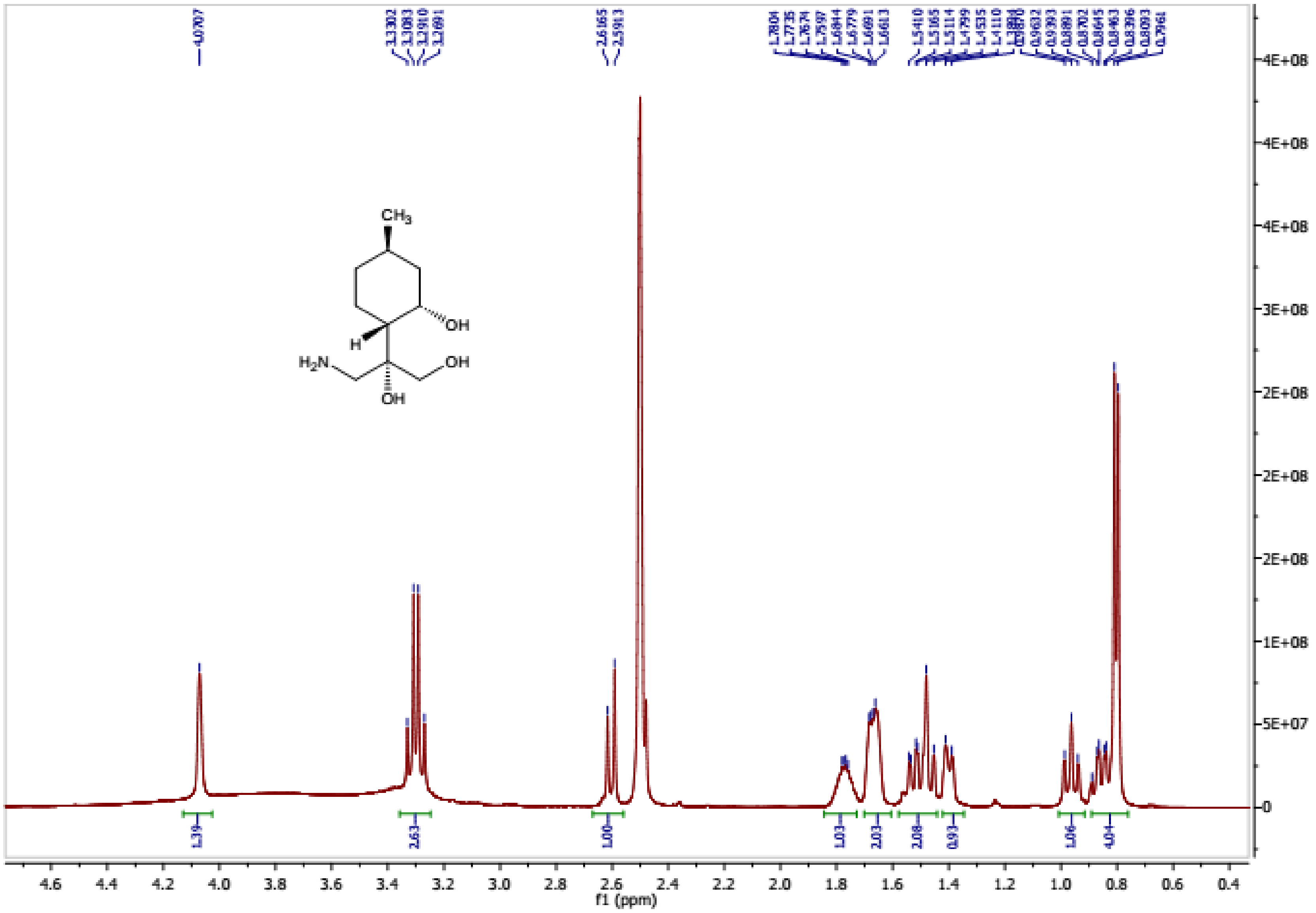

Figure S 41: ^{13}C-NMR of compound **16a**

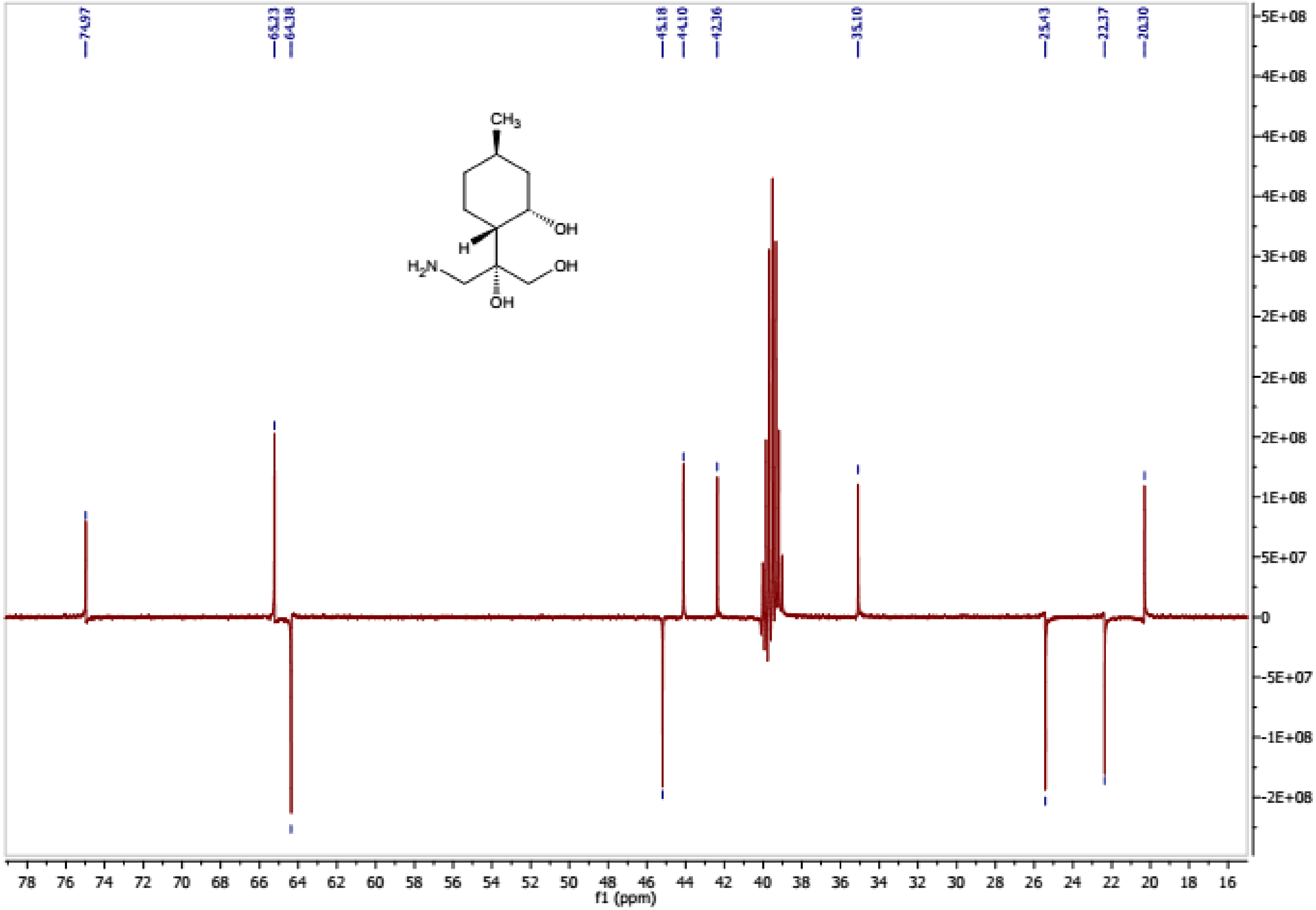

Figure S 42: ^{1}H-NMR of compound **16b**

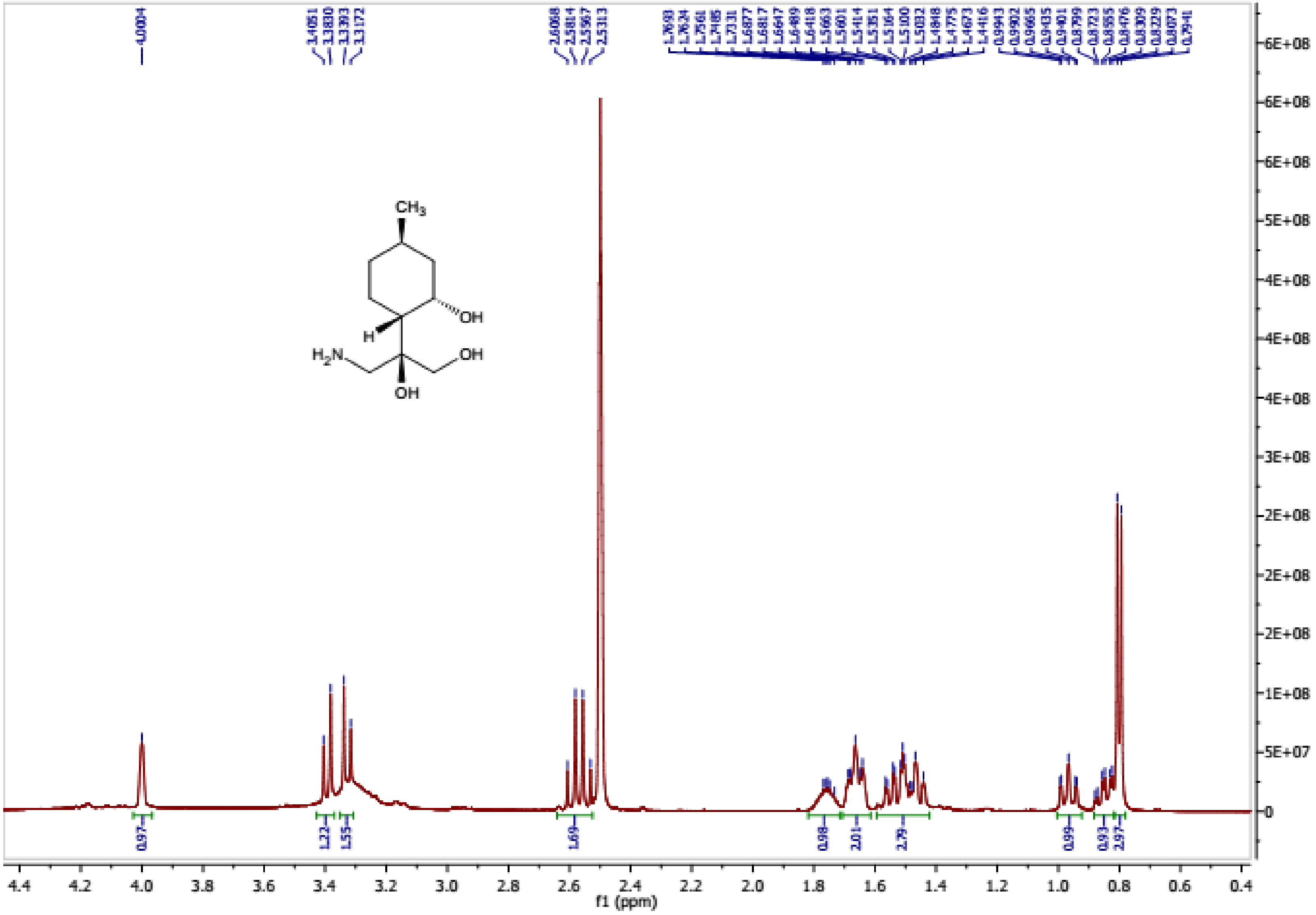

Figure S 43: ^{13}C-NMR of compound **16b**

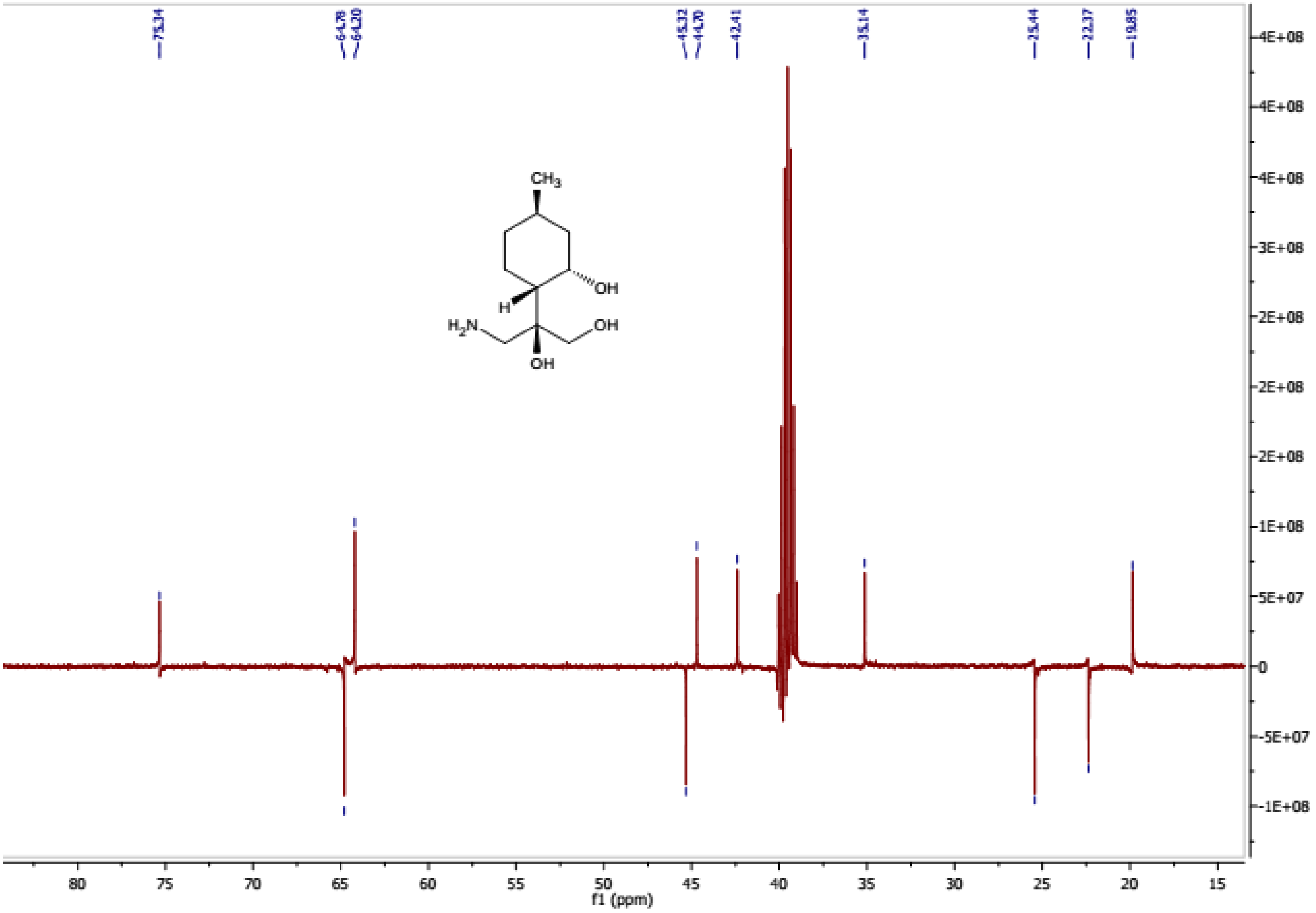

Figure S 44: ^{1}H-NMR of compound **18a**

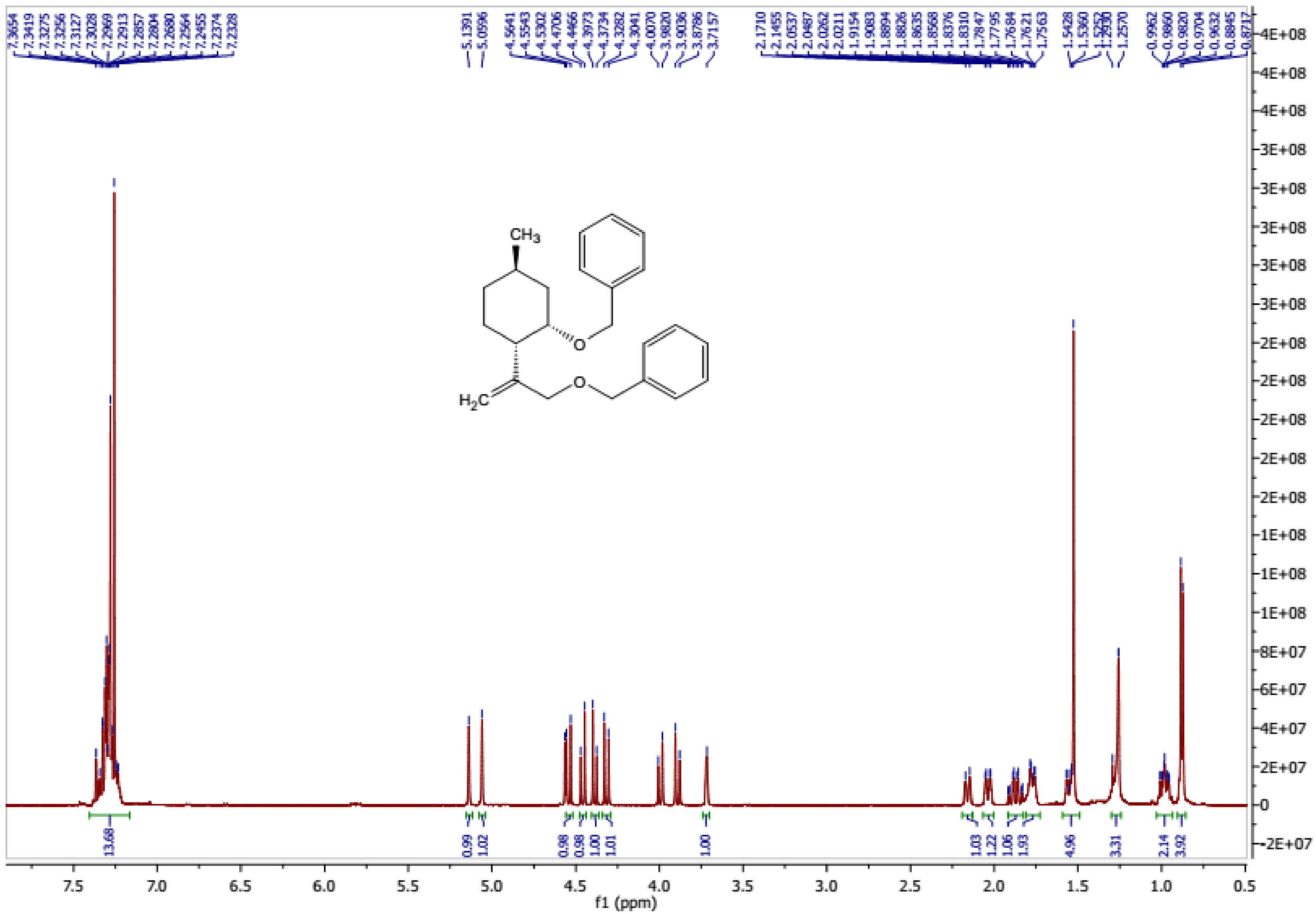

Figure S 45: ^{13}C-NMR of compound **18a**

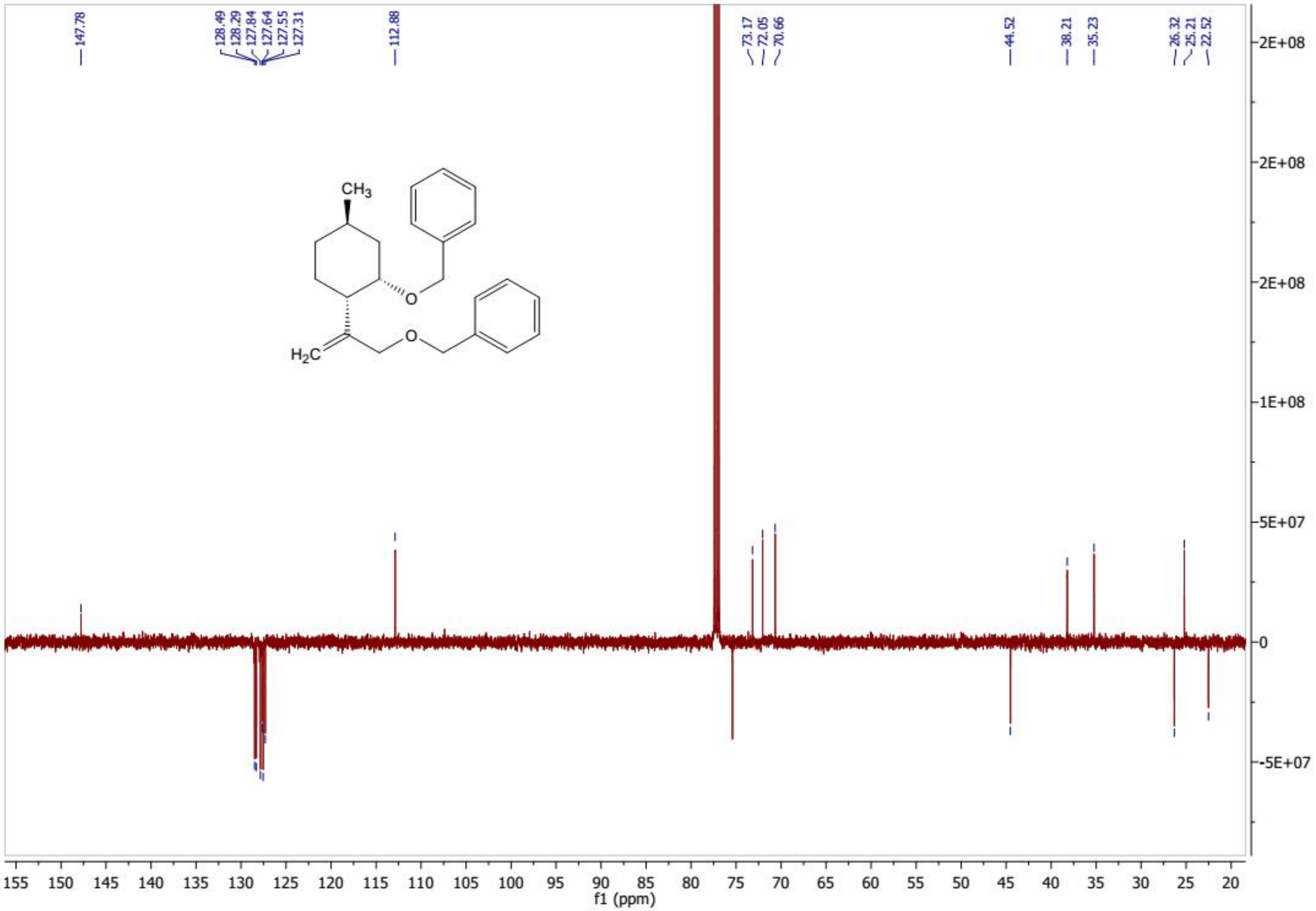

Figure S 46: ^{1}H-NMR of compound **19a**

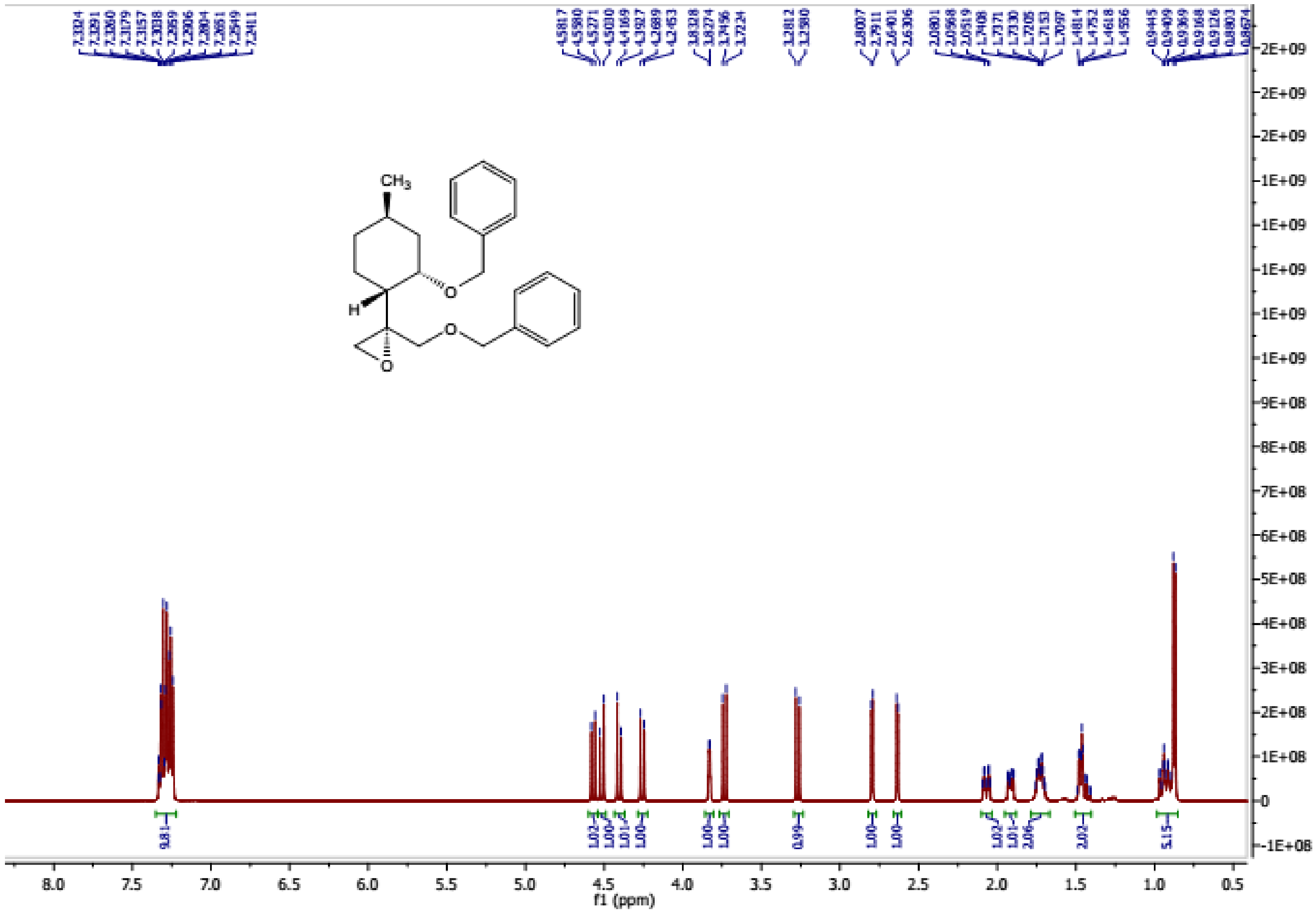

Figure S 47: ^{13}C-NMR of compound **19**

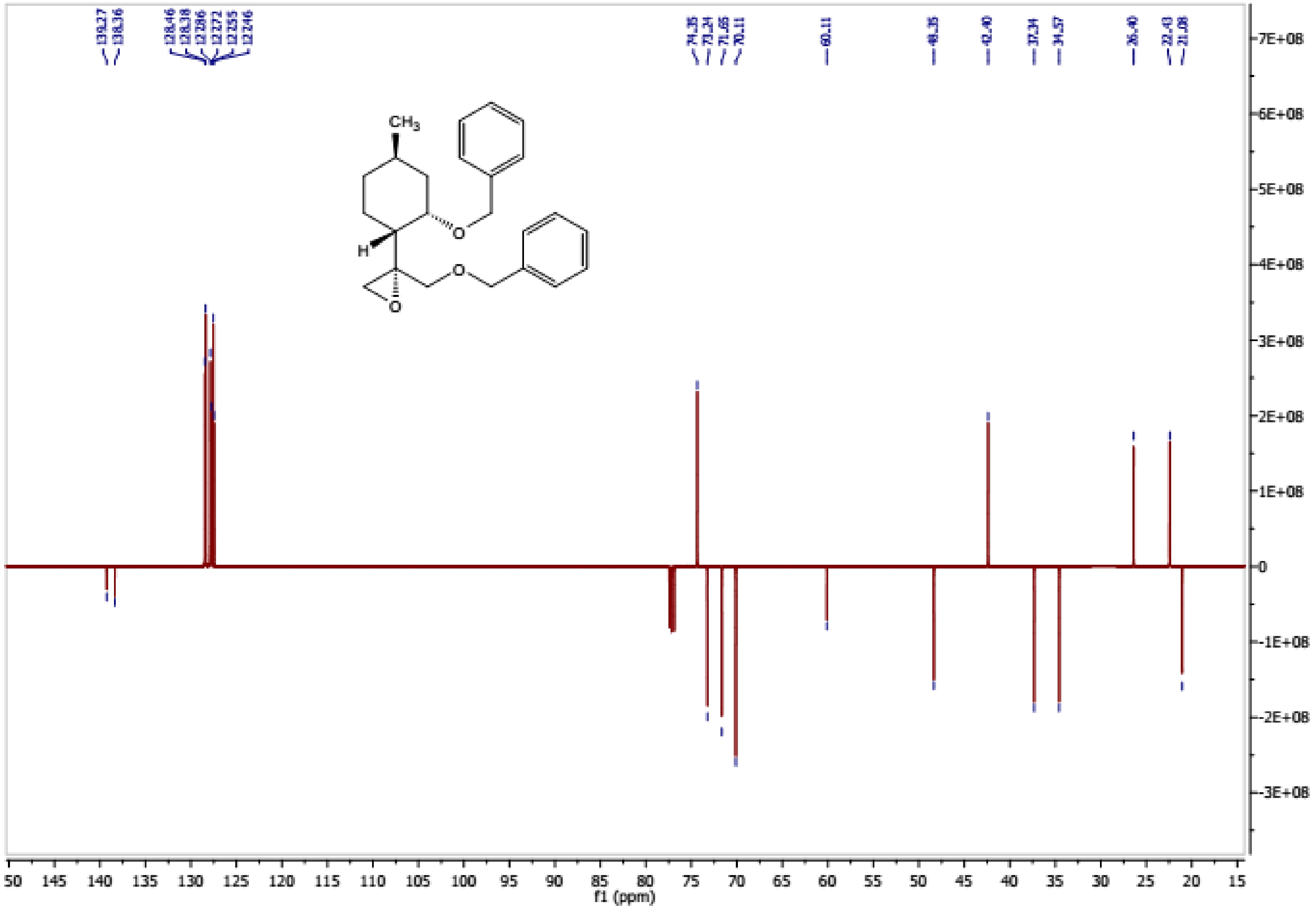

Figure S 48: ^{1}H-NMR of compound **19b**

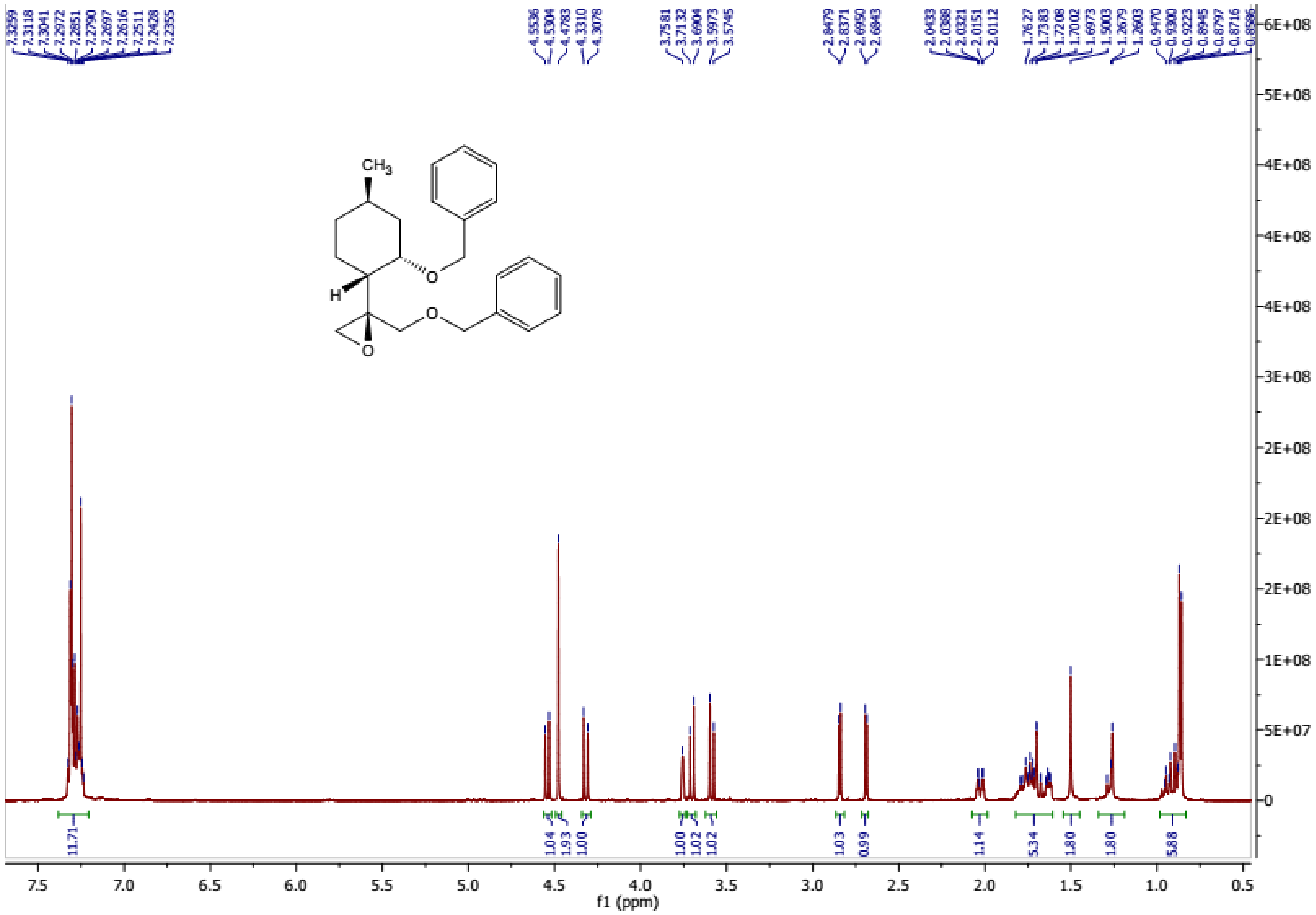

Figure S 49: ^{13}C-NMR of compound **19b**

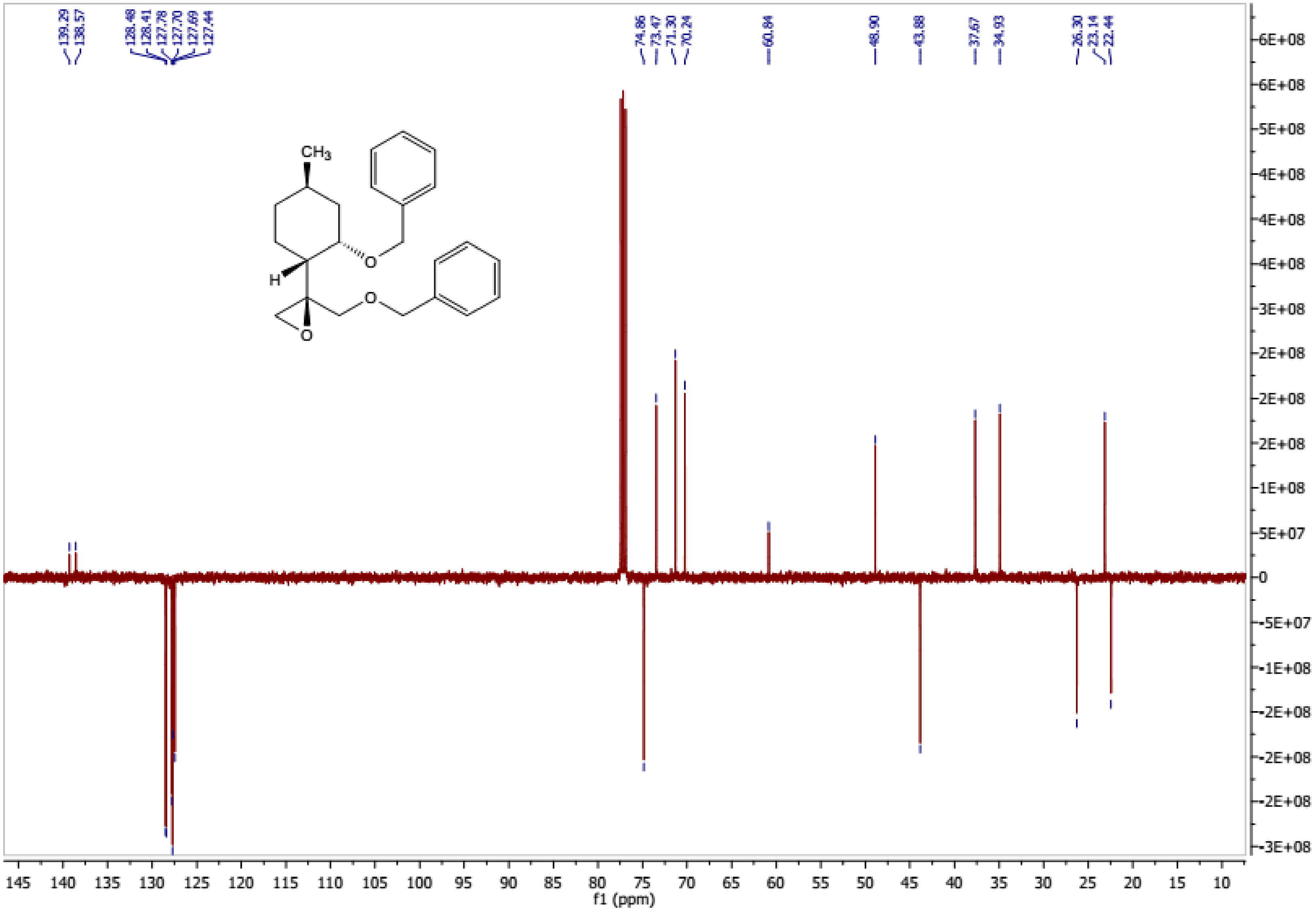

Figure S 50: ^{1}H-NMR of compound **20a**

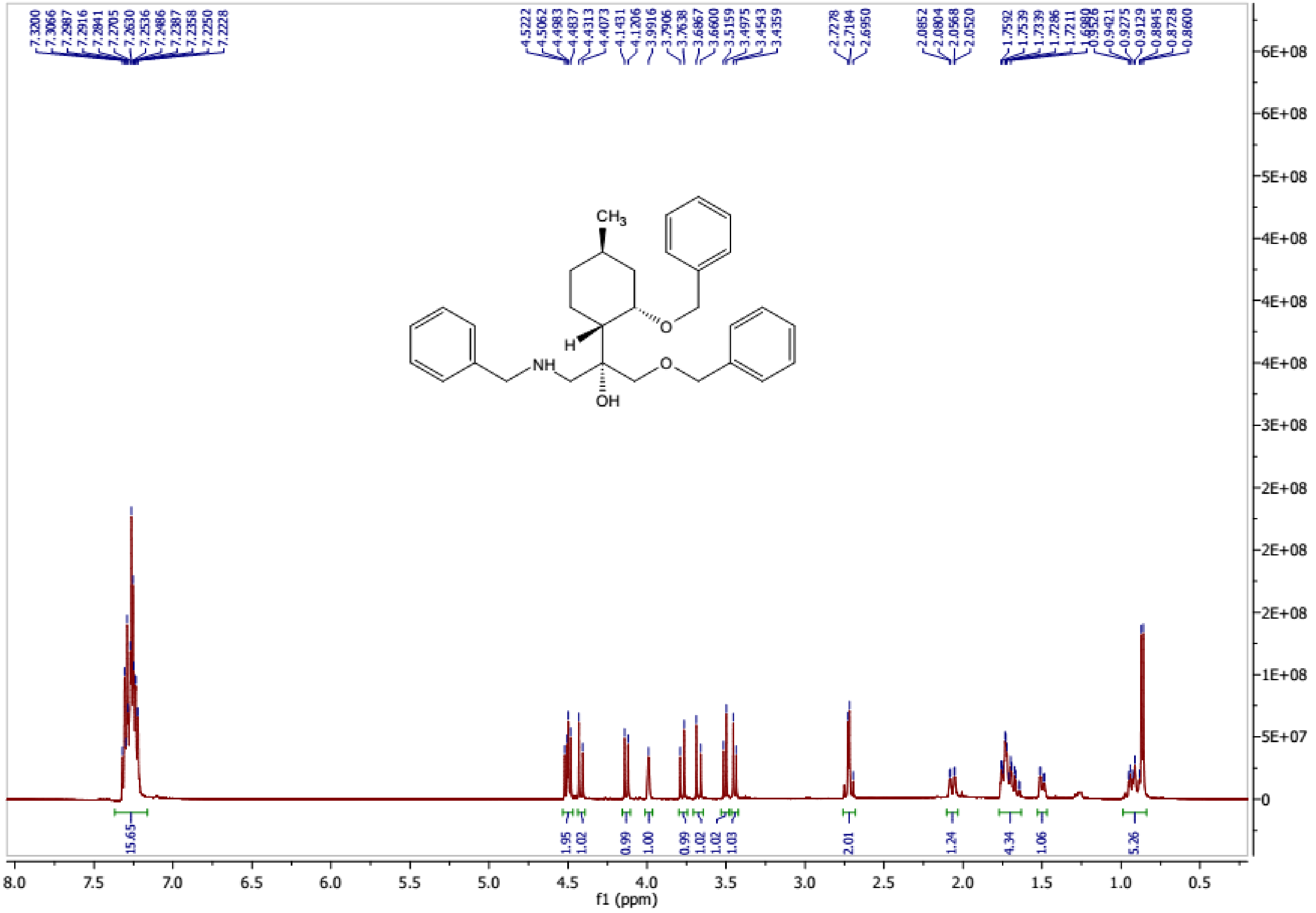

Figure S 51: ^{13}C-NMR of compound **20a**

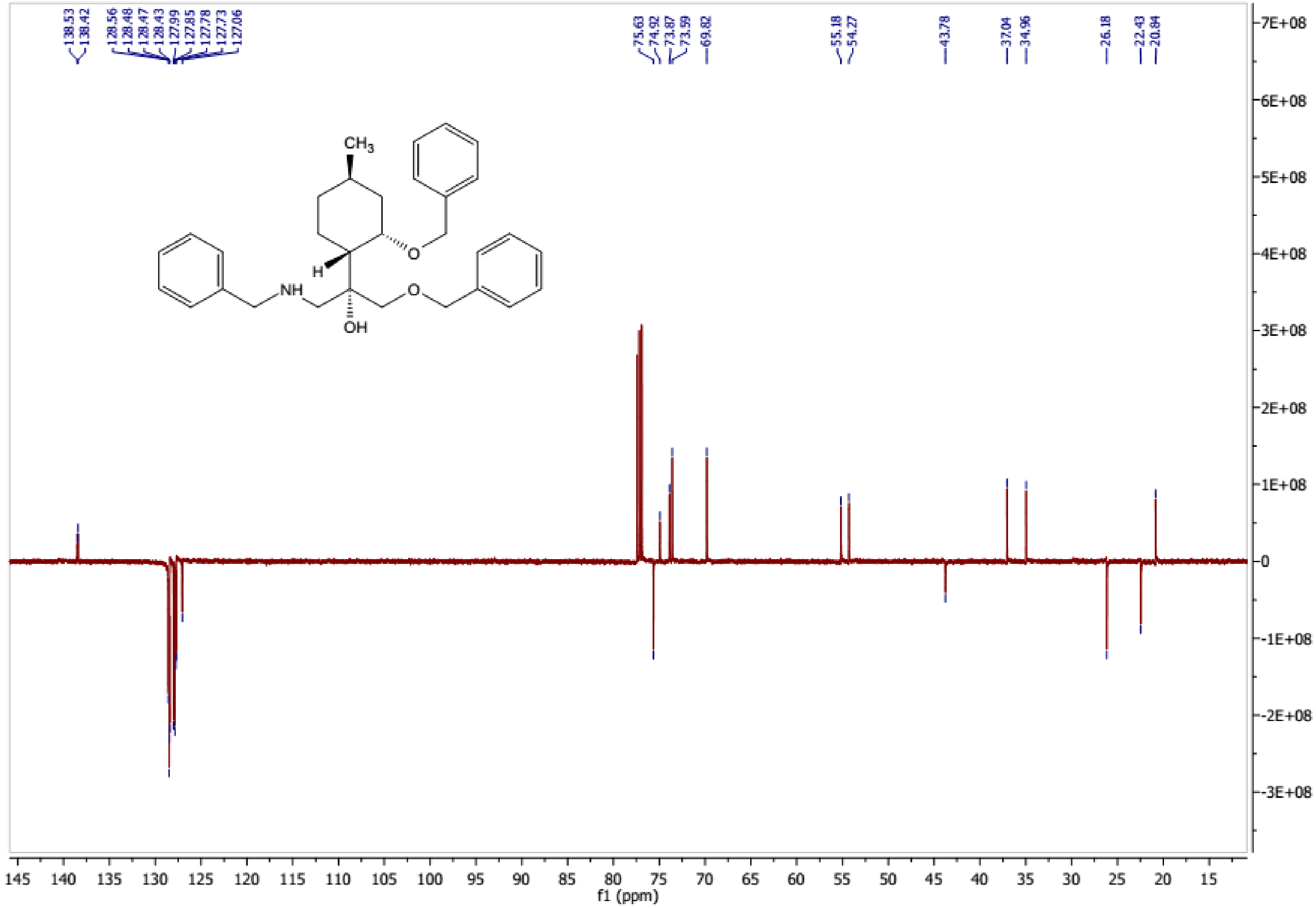

Figure S 52: ¹H-NMR of compound **20b**

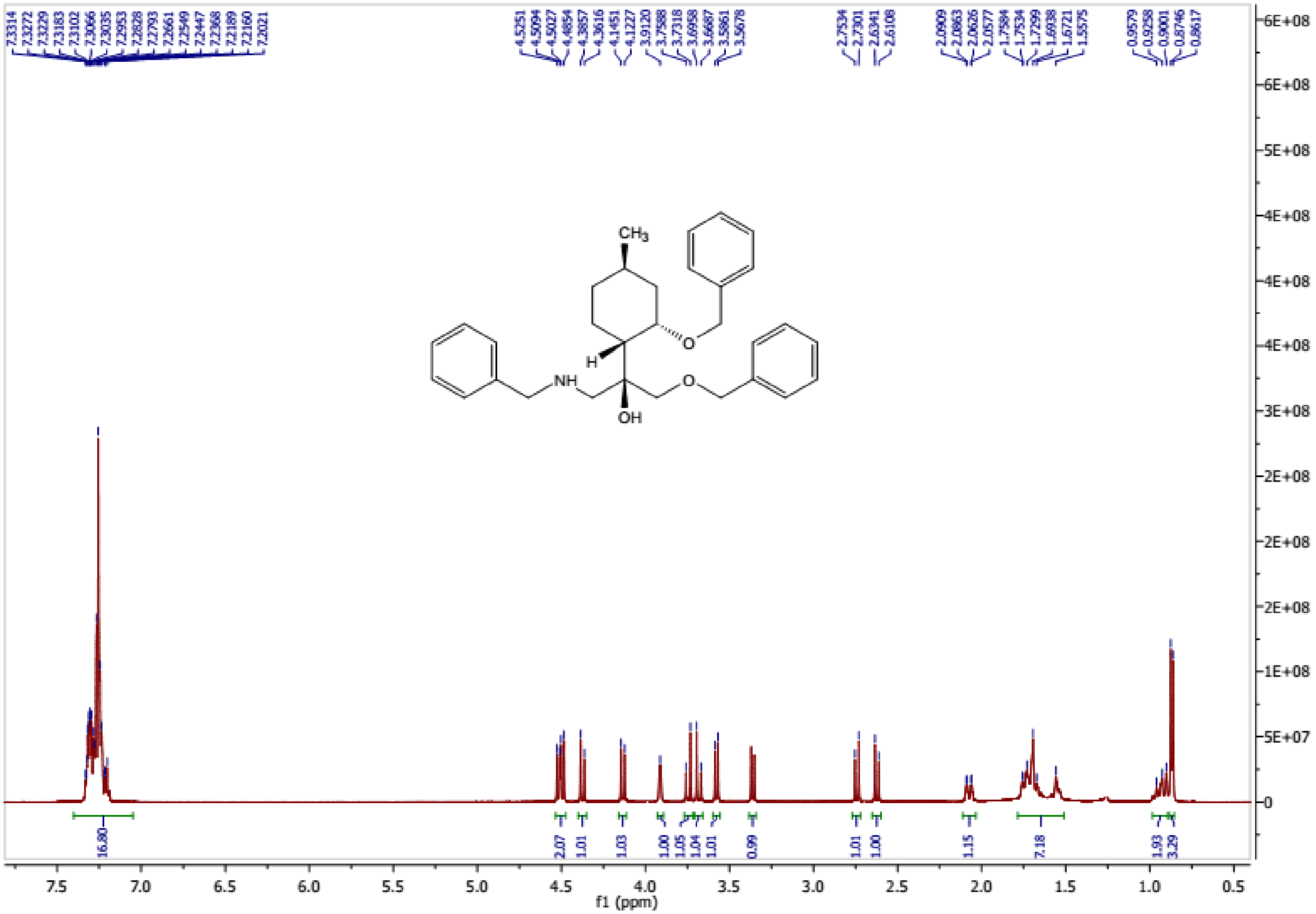

Figure S 53: ^{13}C-NMR of compound **20b**

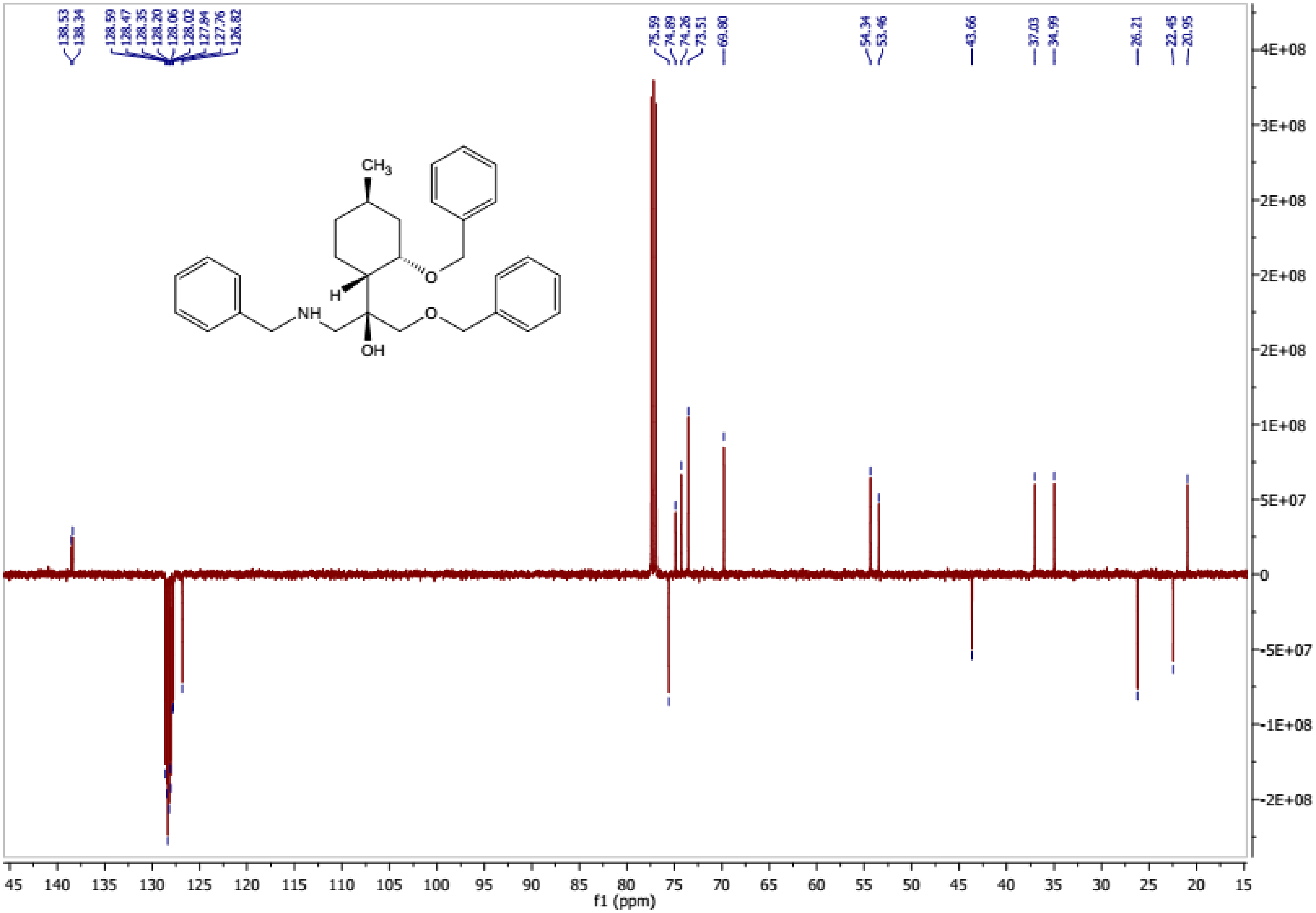

Figure S 54: ^{1}H-NMR of compound **21a**

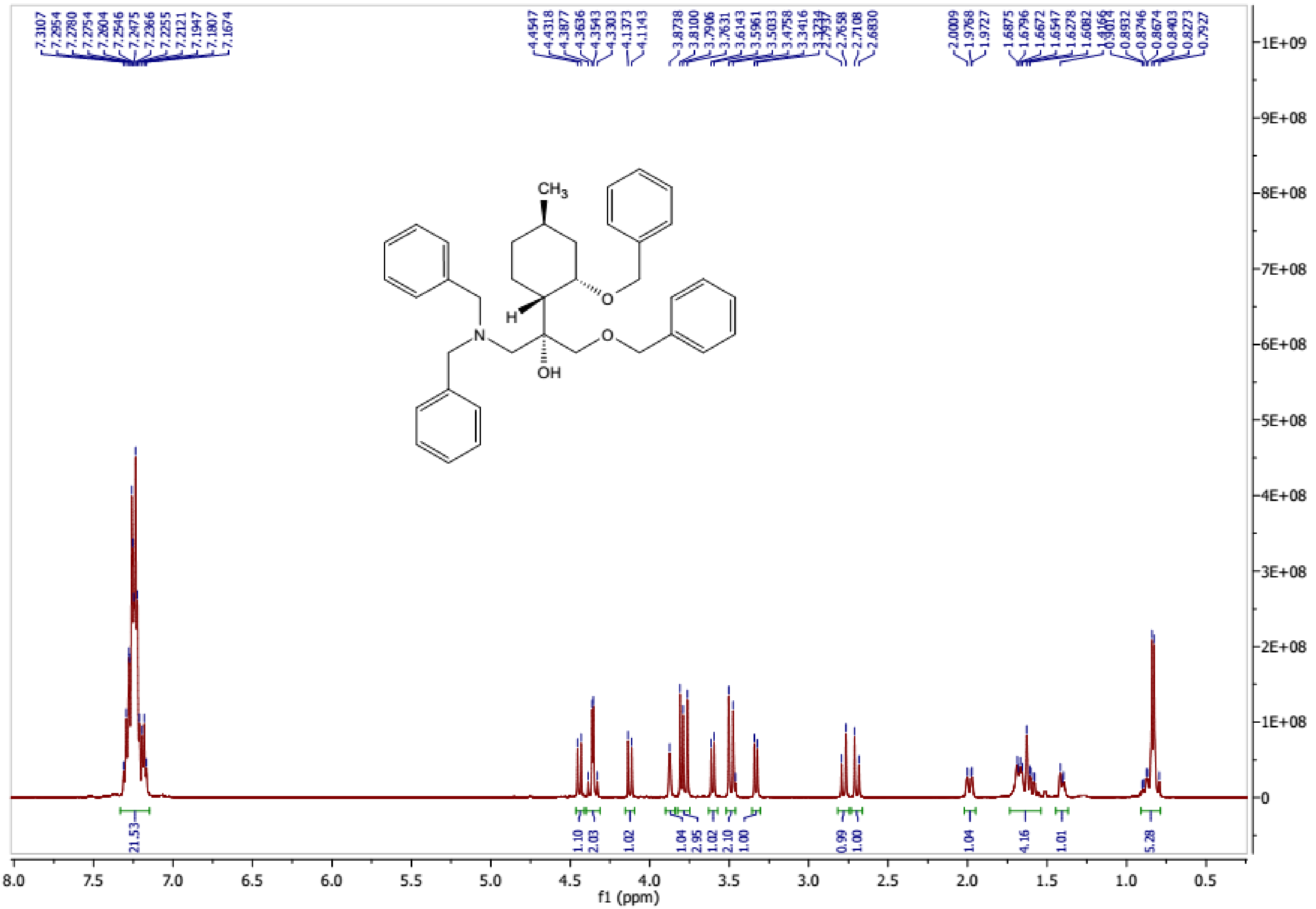

Figure S 55: ^{13}C-NMR of compound **21a**

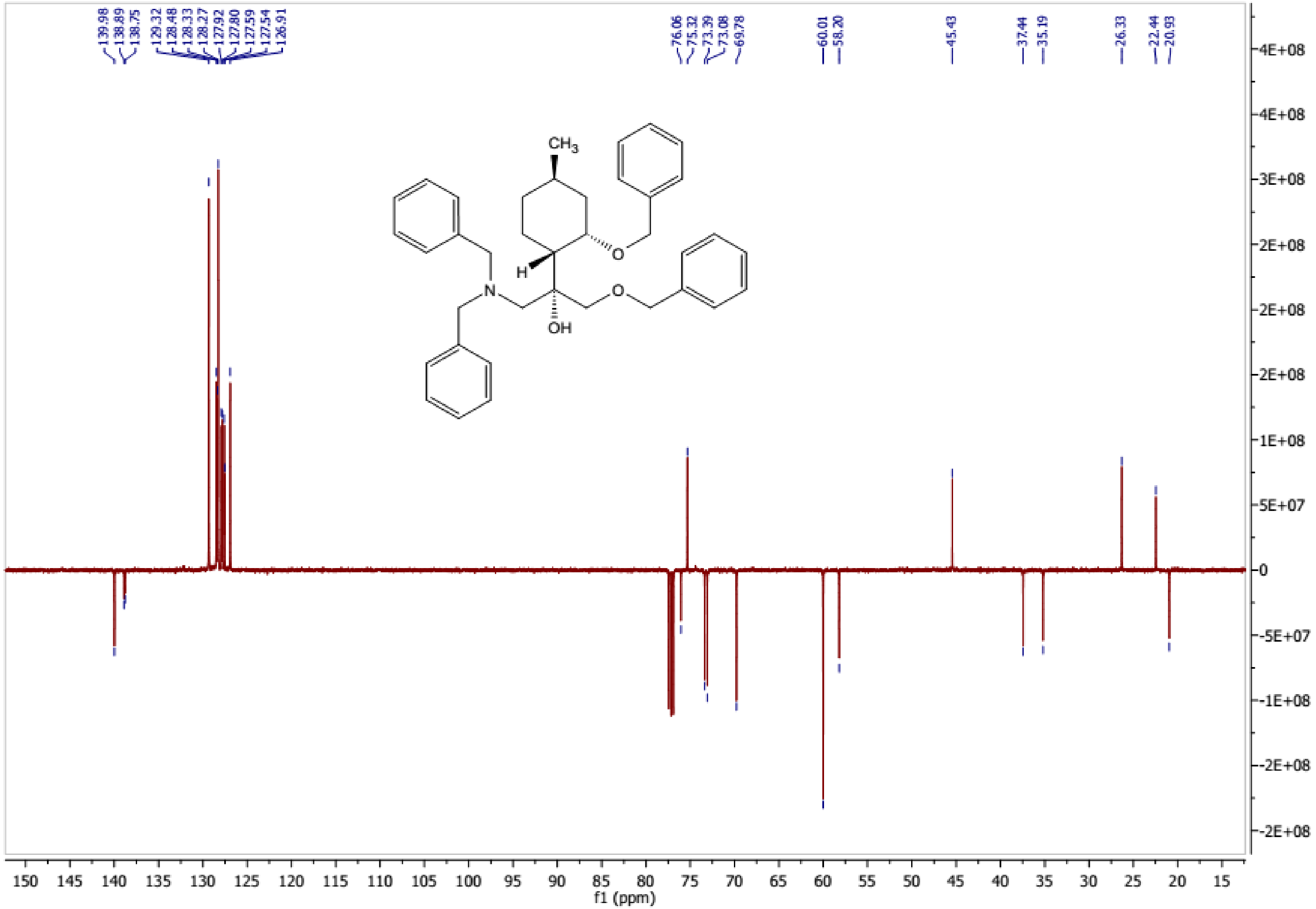

Figure S 56: ¹H-NMR of compound **21b**

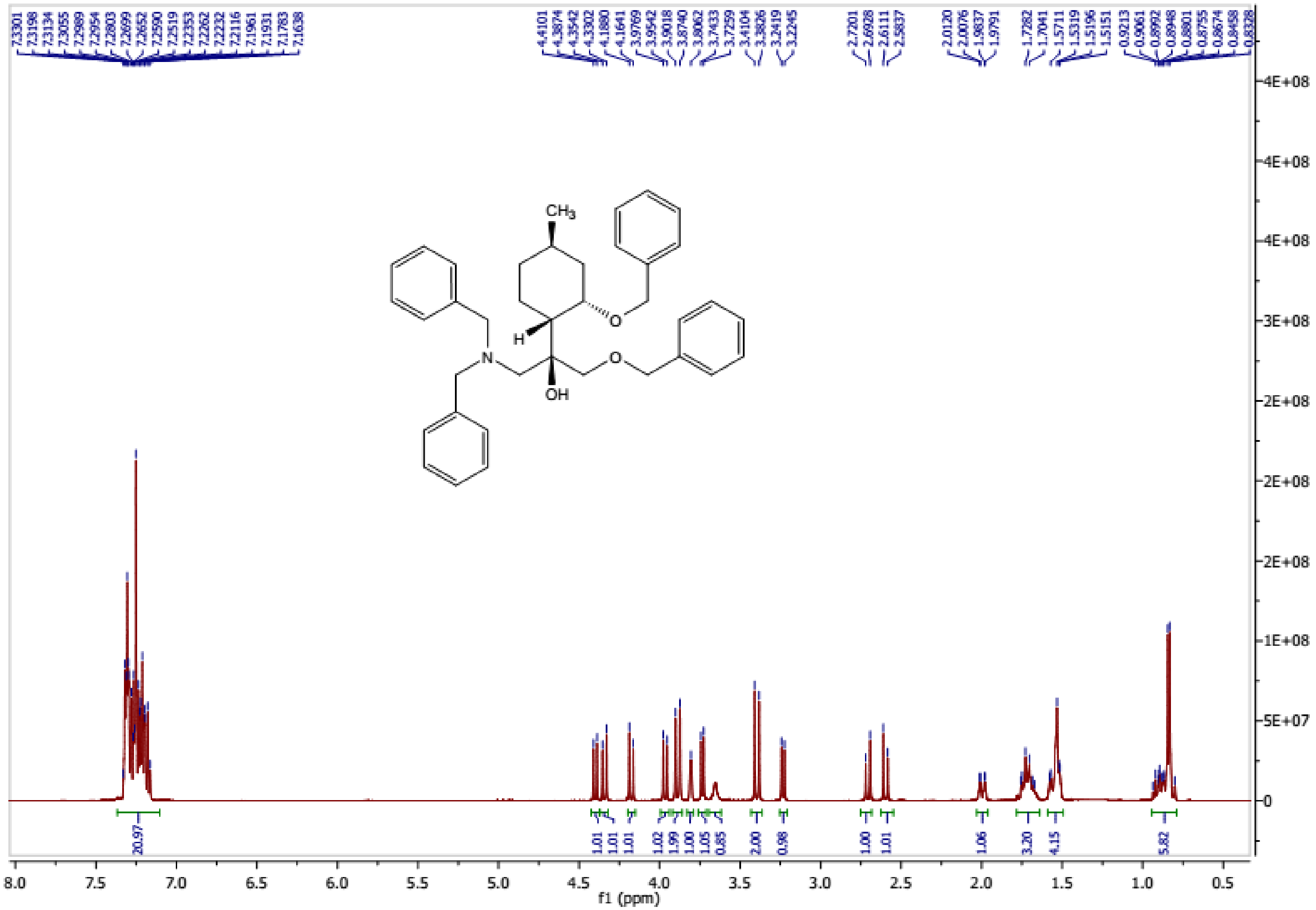

Figure S 57: ^{13}C-NMR of compound **21b**

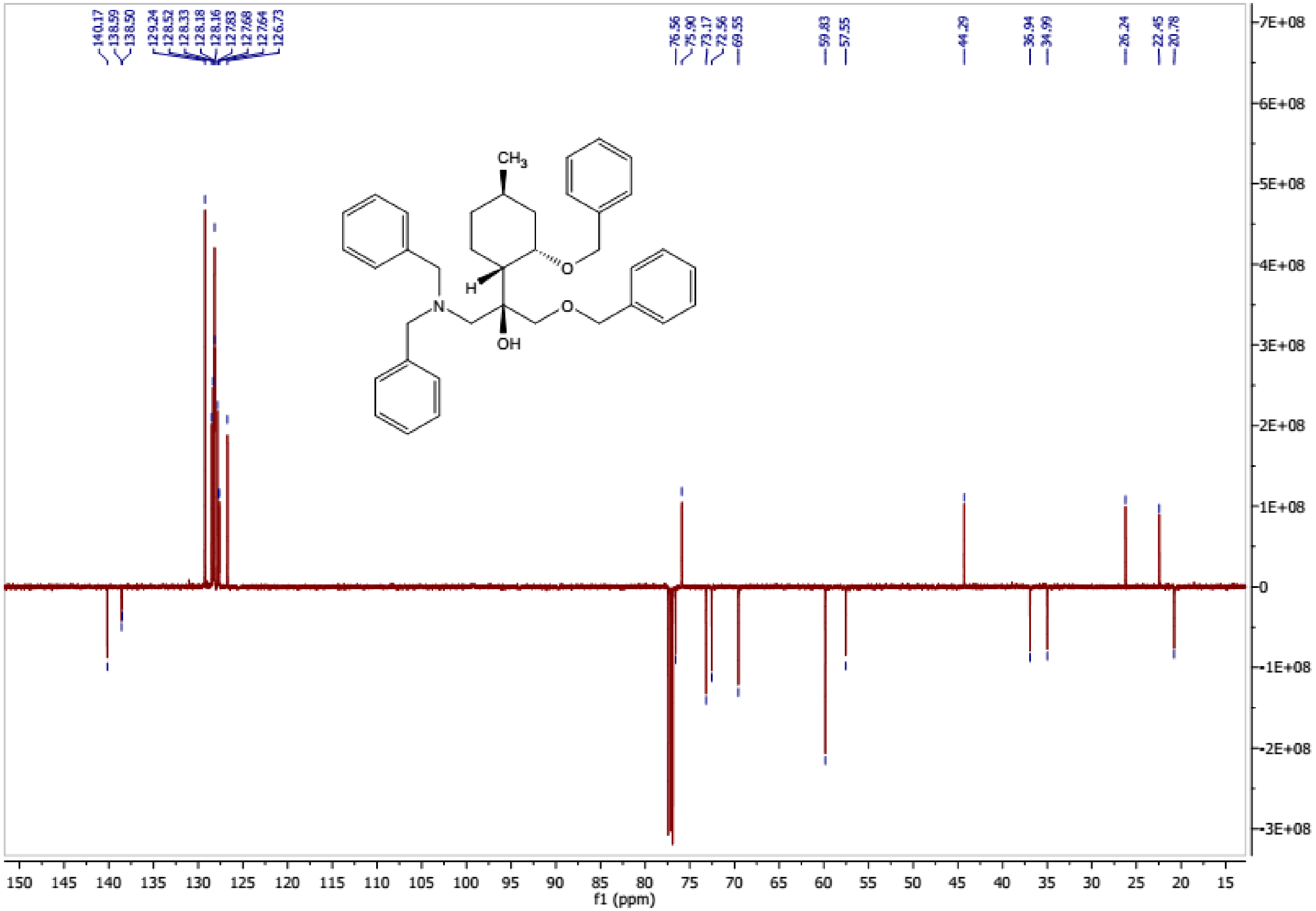

Figure S 58: ^{1}H-NMR of compound **22a**

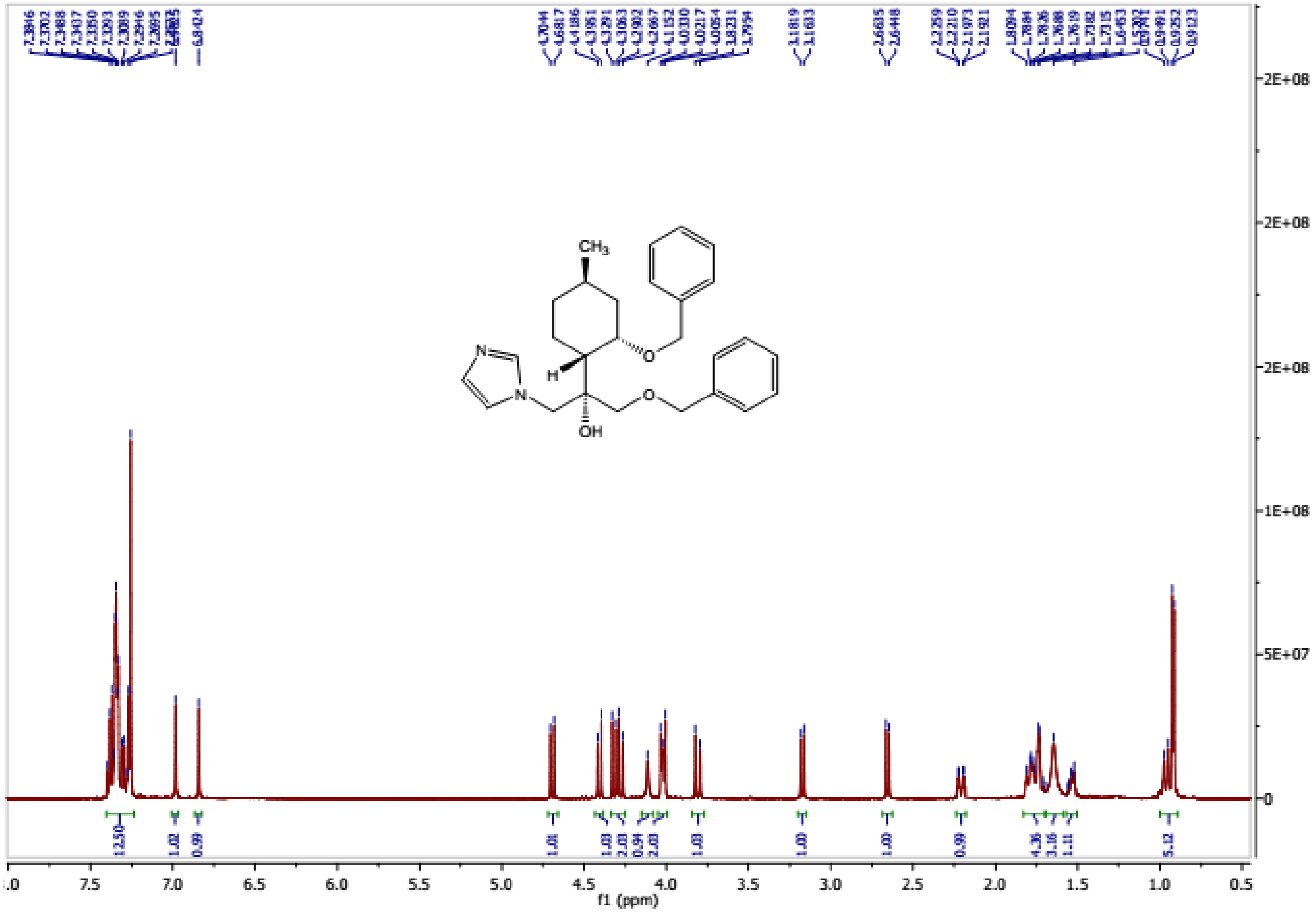

Figure S 59: ^{13}C-NMR of compound **22a**

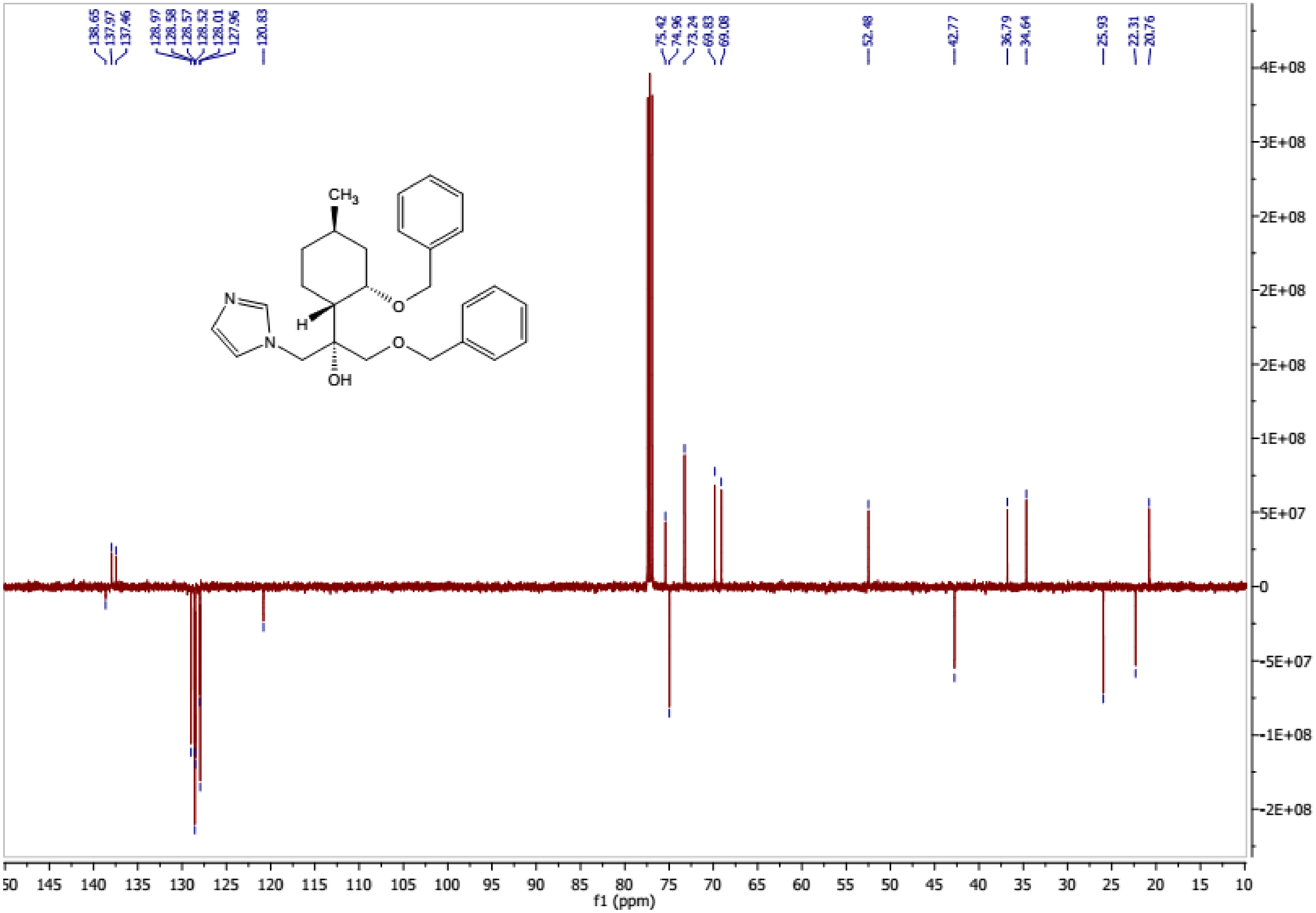

Figure S 60: ^{1}H-NMR of compound **22b**

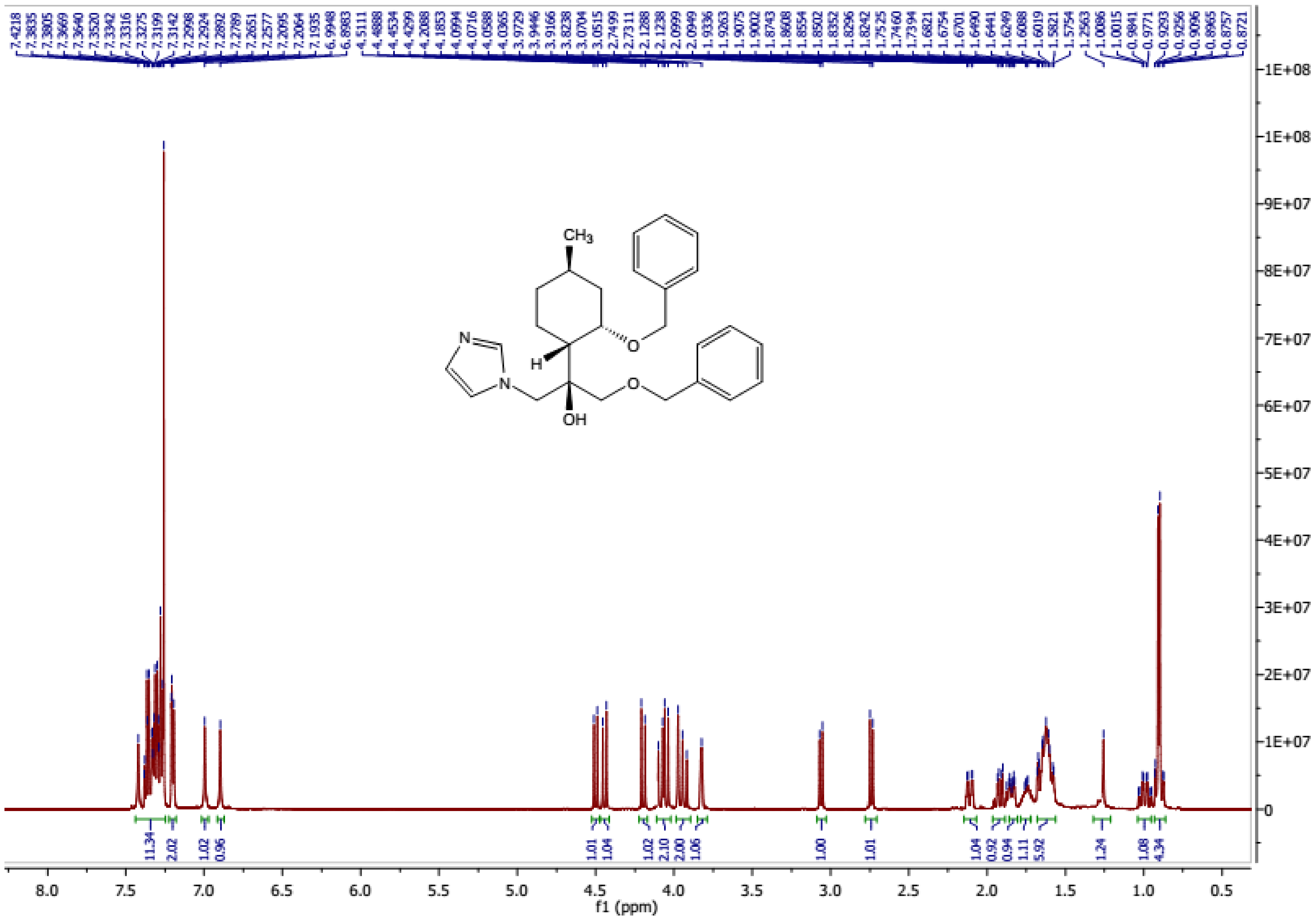

Figure S 61: ^{13}C-NMR of compound **22b**

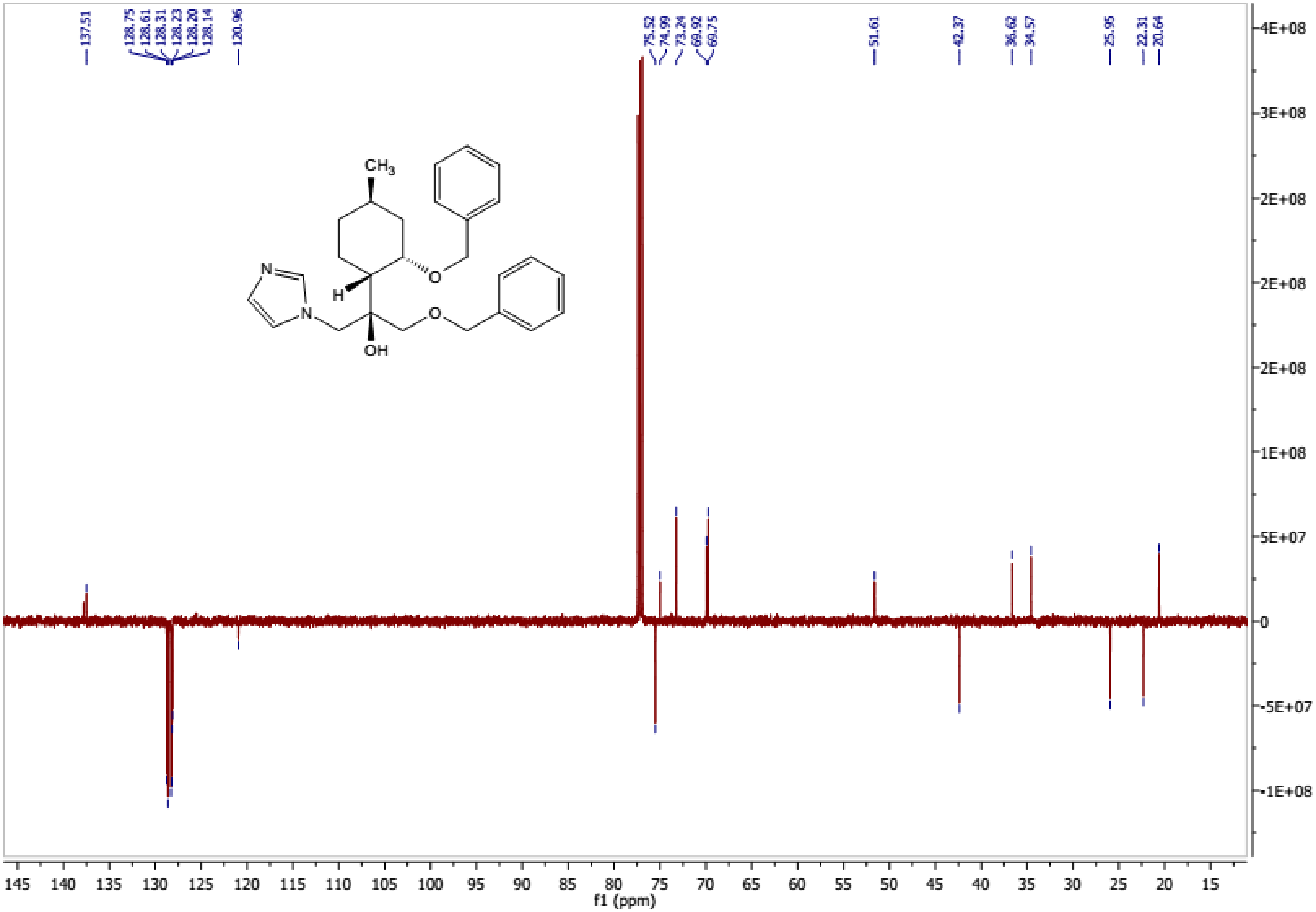

Figure S 62: ¹H-NMR of compound **23a**

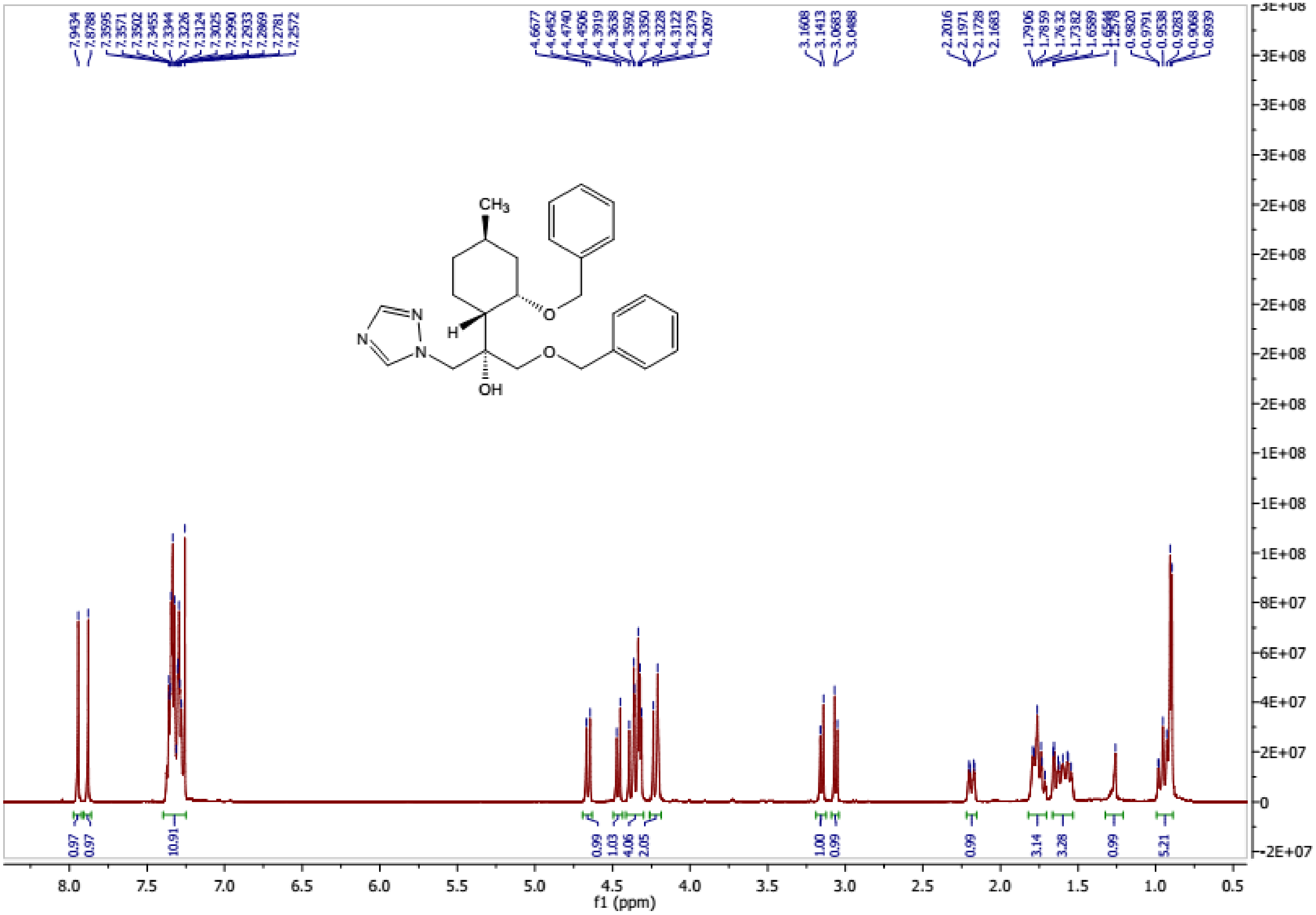

Figure S 63: ^{13}C-NMR of compound **23a**

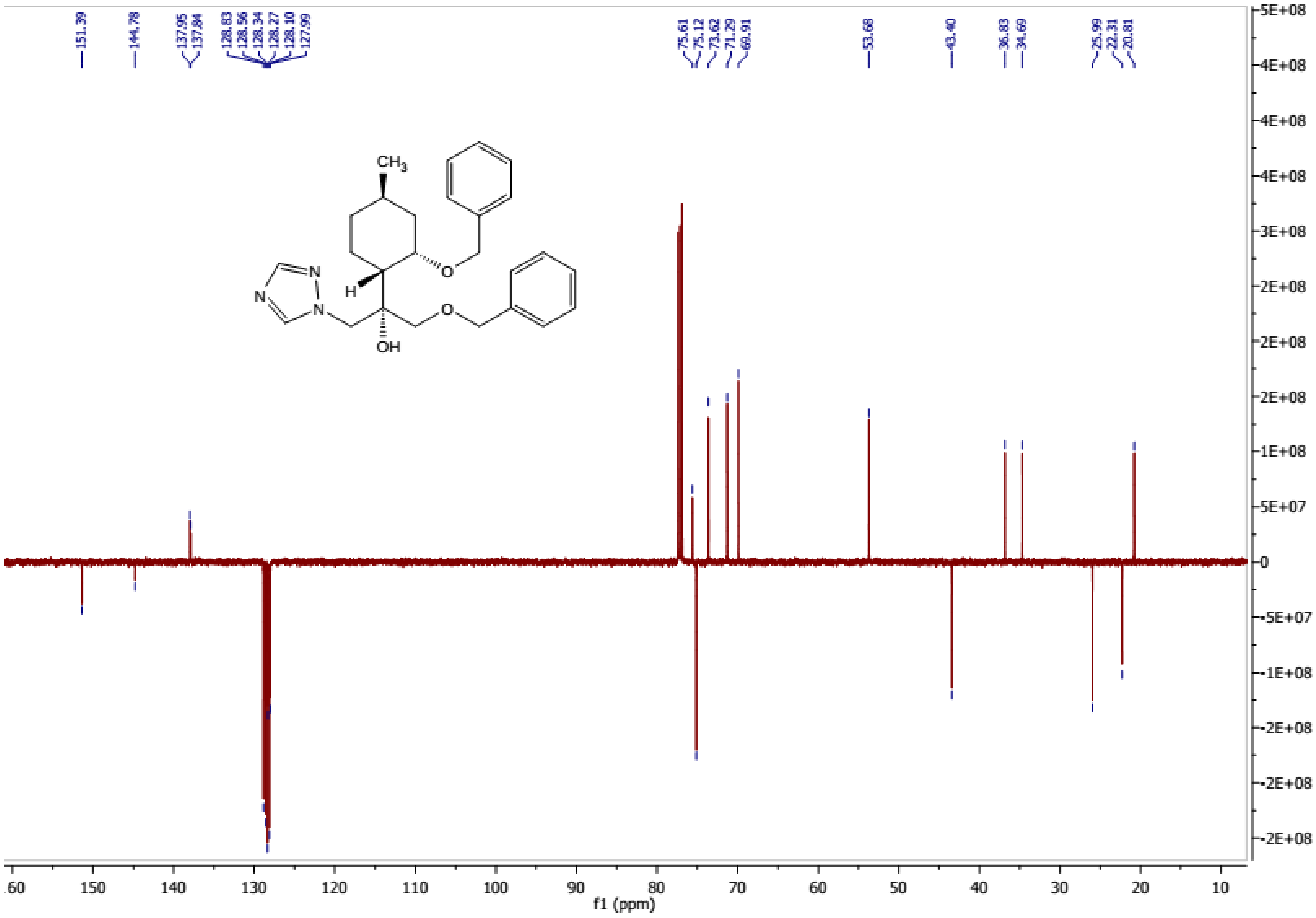

Figure S 64: ^{1}H-NMR of compound **23b**

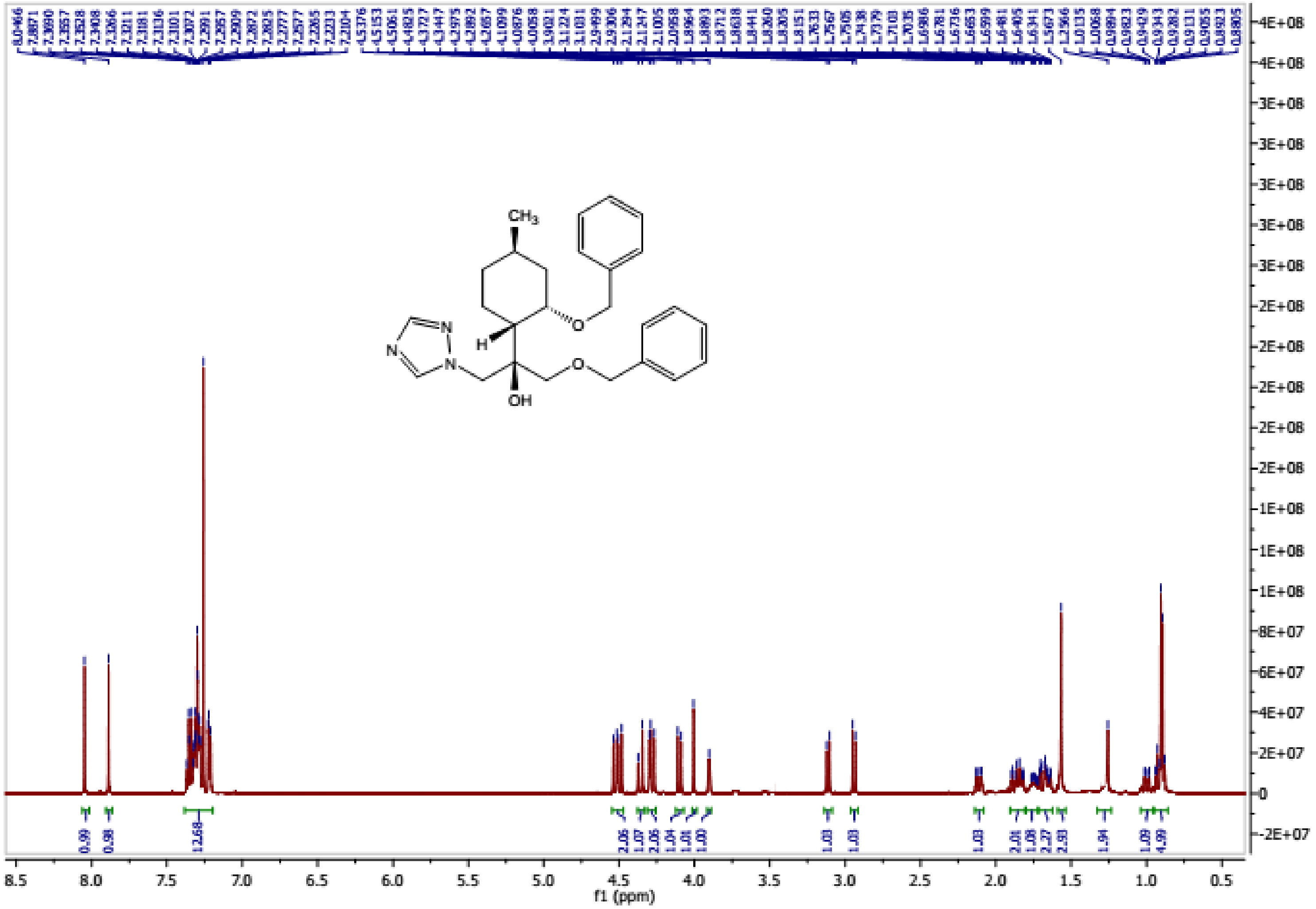

Figure S 65: ^{13}C-NMR of compound **23b**

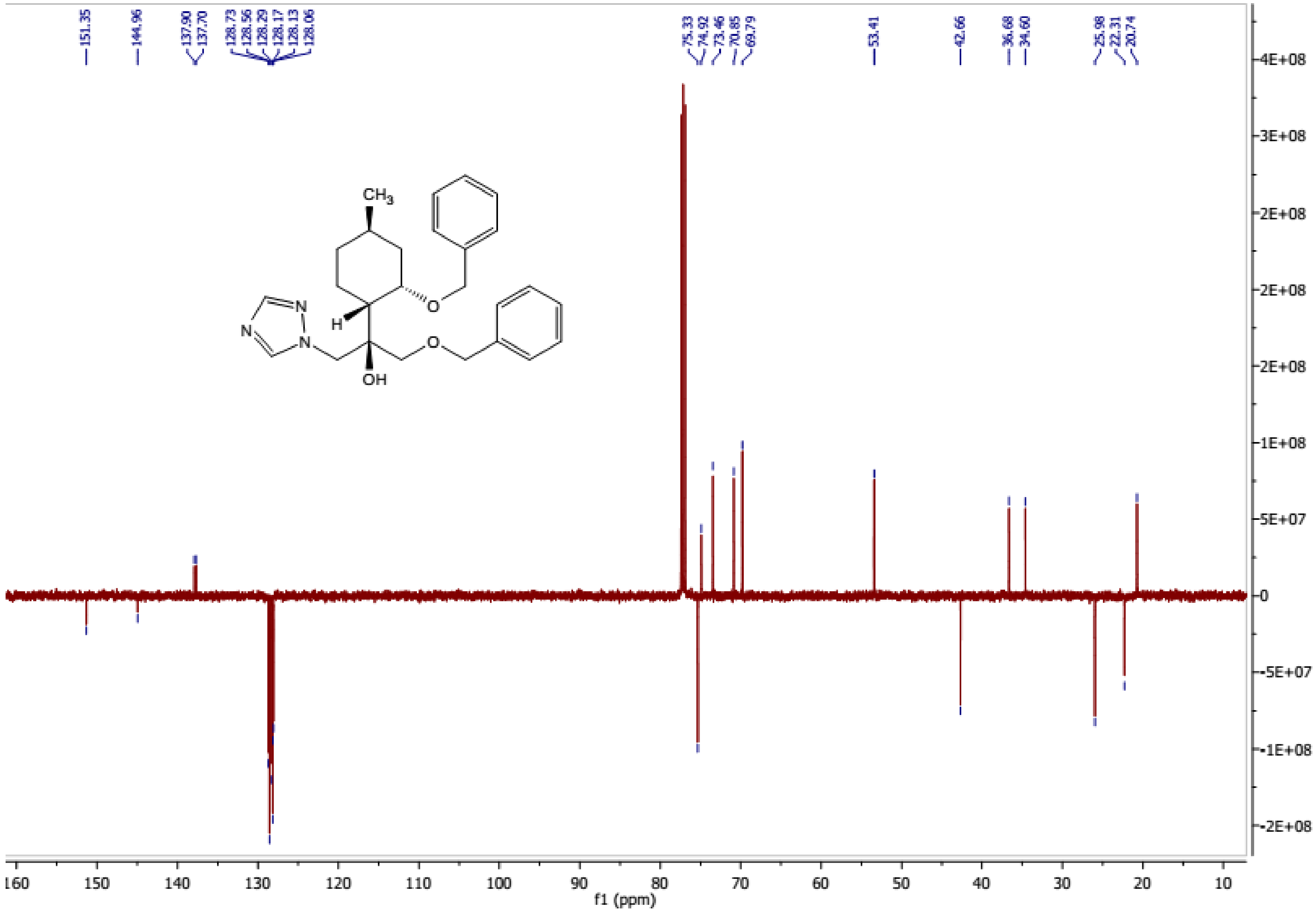

Figure S 66: ^{1}H-NMR of compound **24a**

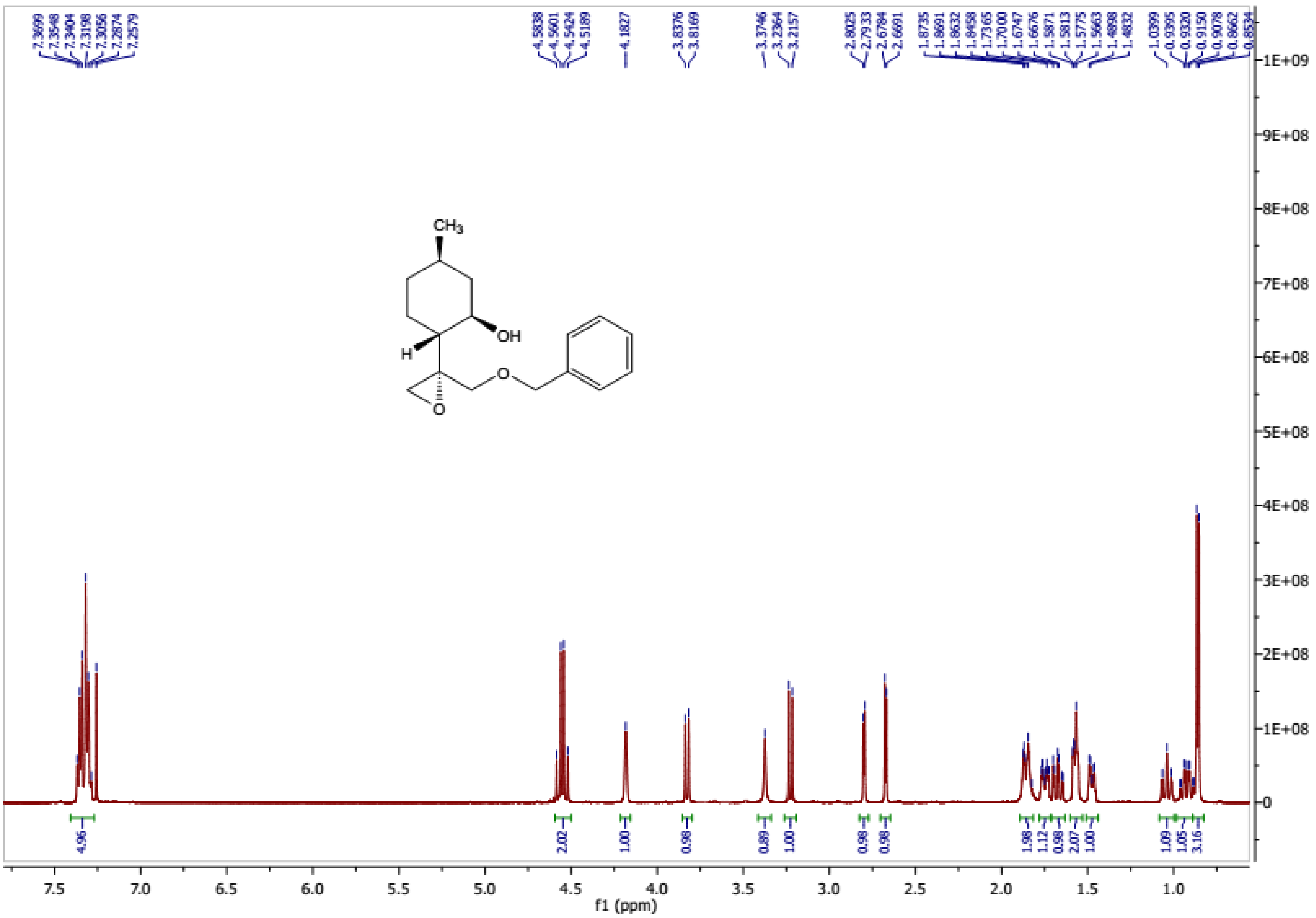

Figure S 67: ^{13}C-NMR of compound **24a**

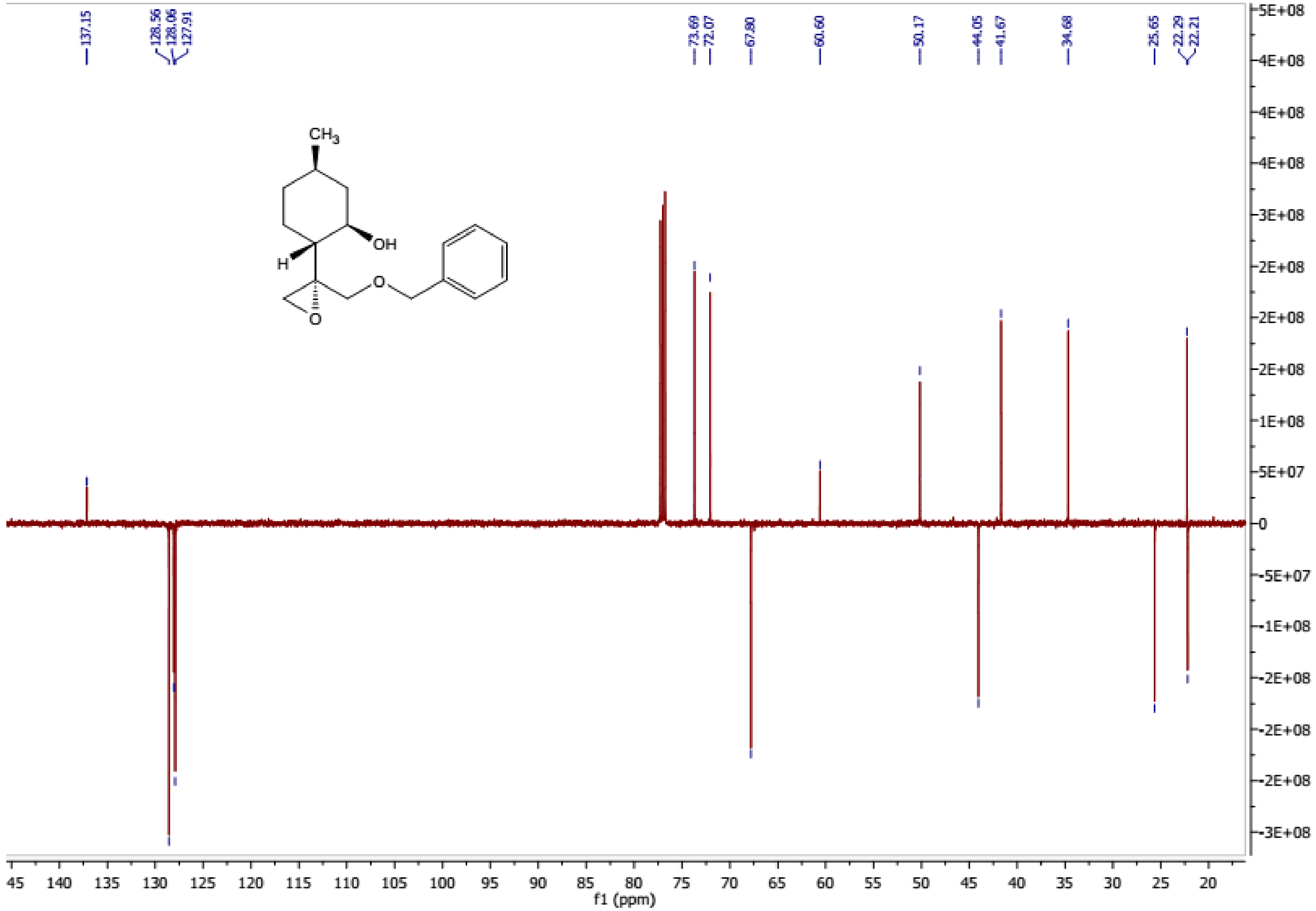

Figure S 68: ^{1}H-NMR of compound **24b**

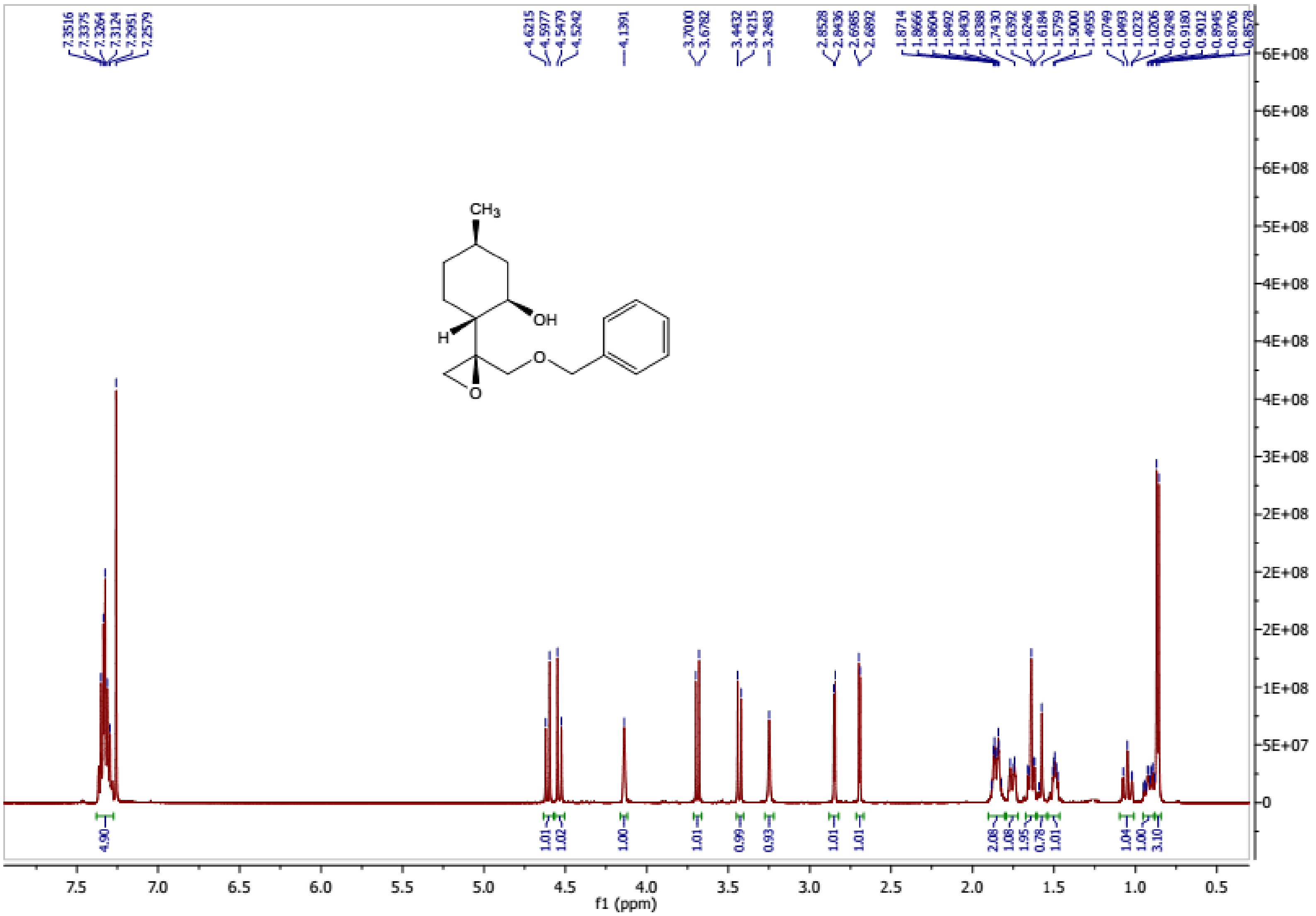

Figure S 69: ^{13}C-NMR of compound **24b**

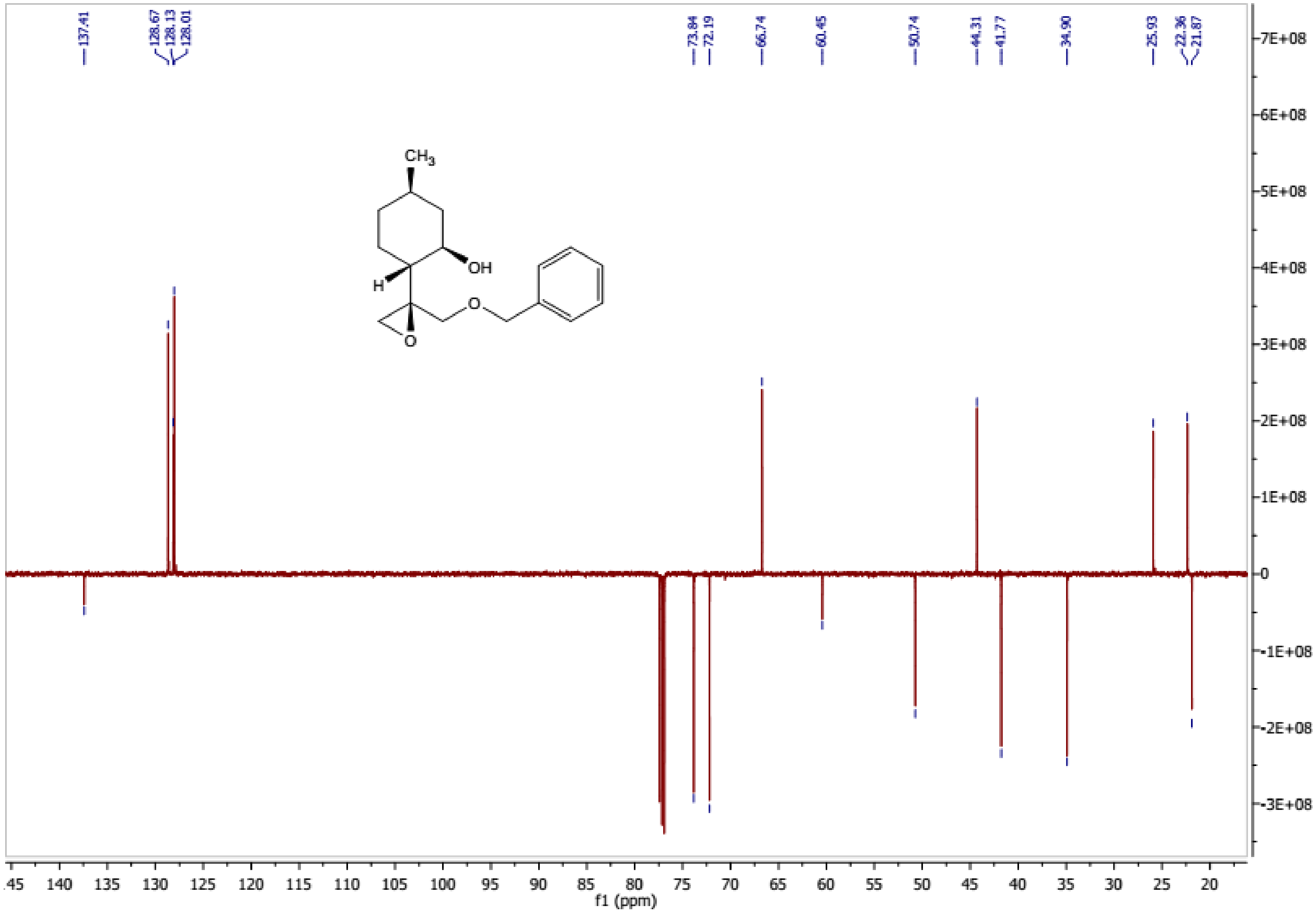

Figure S 70: ^{1}H-NMR of compound **25a**

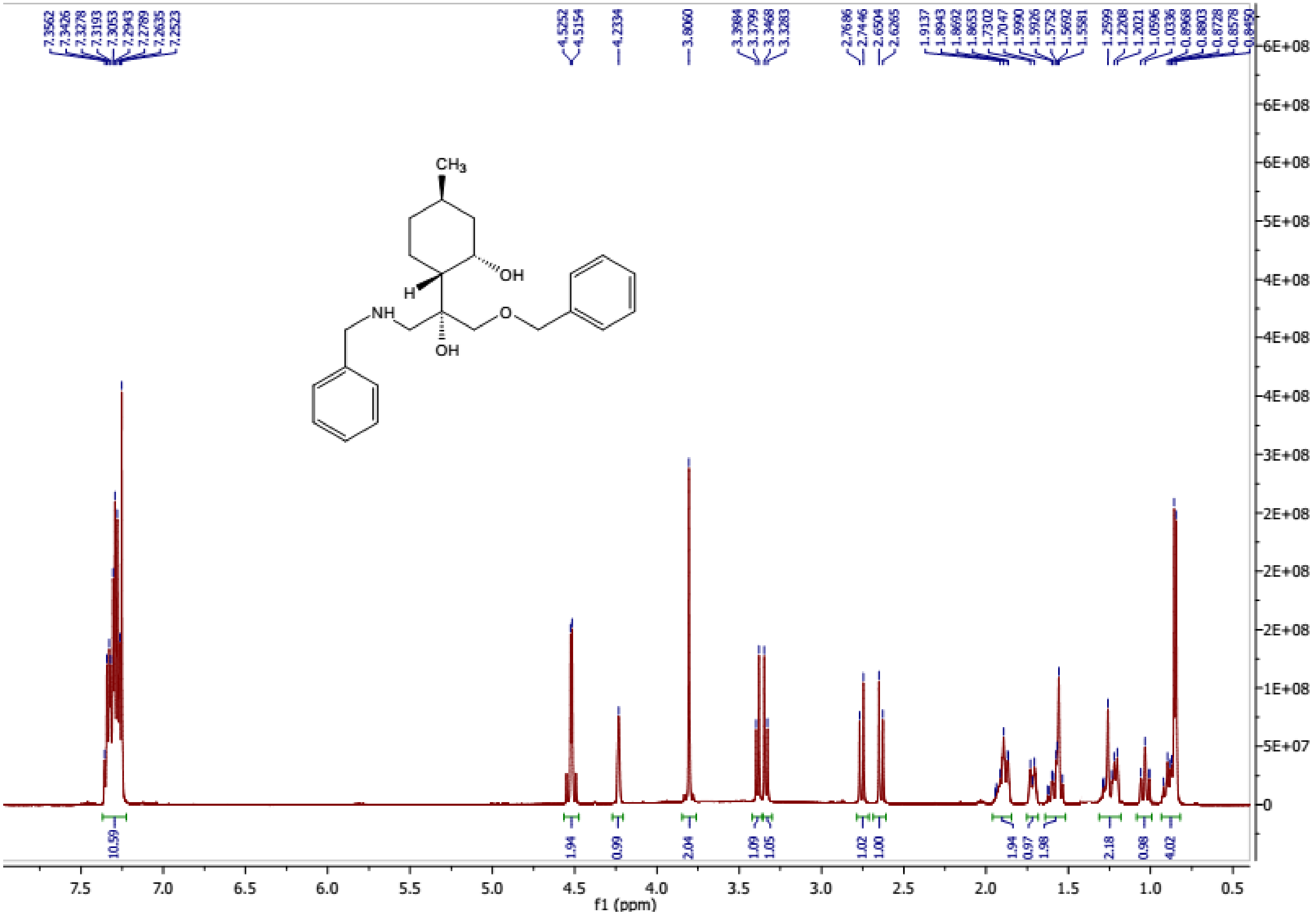

Figure S 71: ^{13}C-NMR of compound **25a**

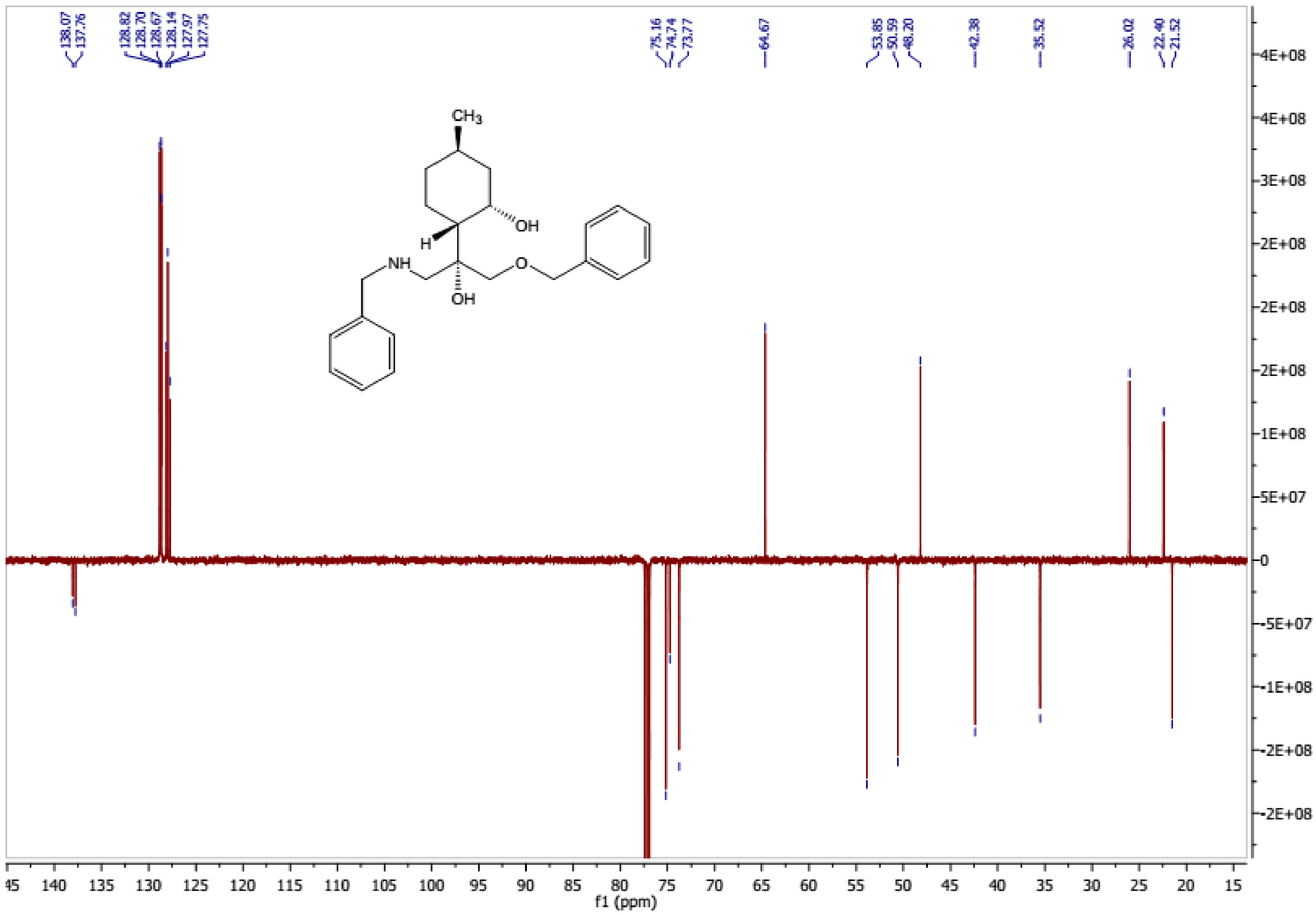

Figure S 72: ^{1}H-NMR of compound **25b**

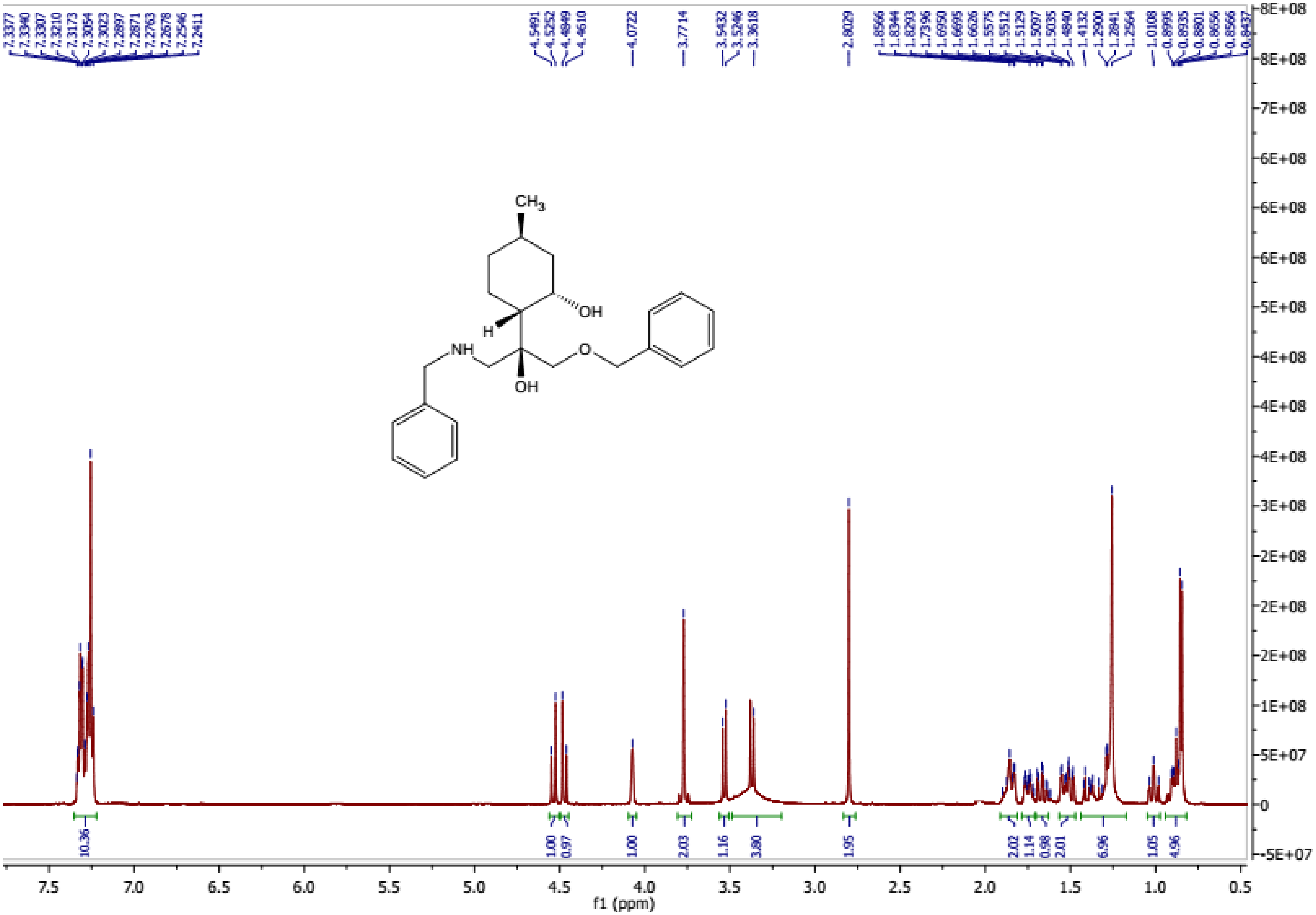

Figure S 73: ^{13}C-NMR of compound **25b**

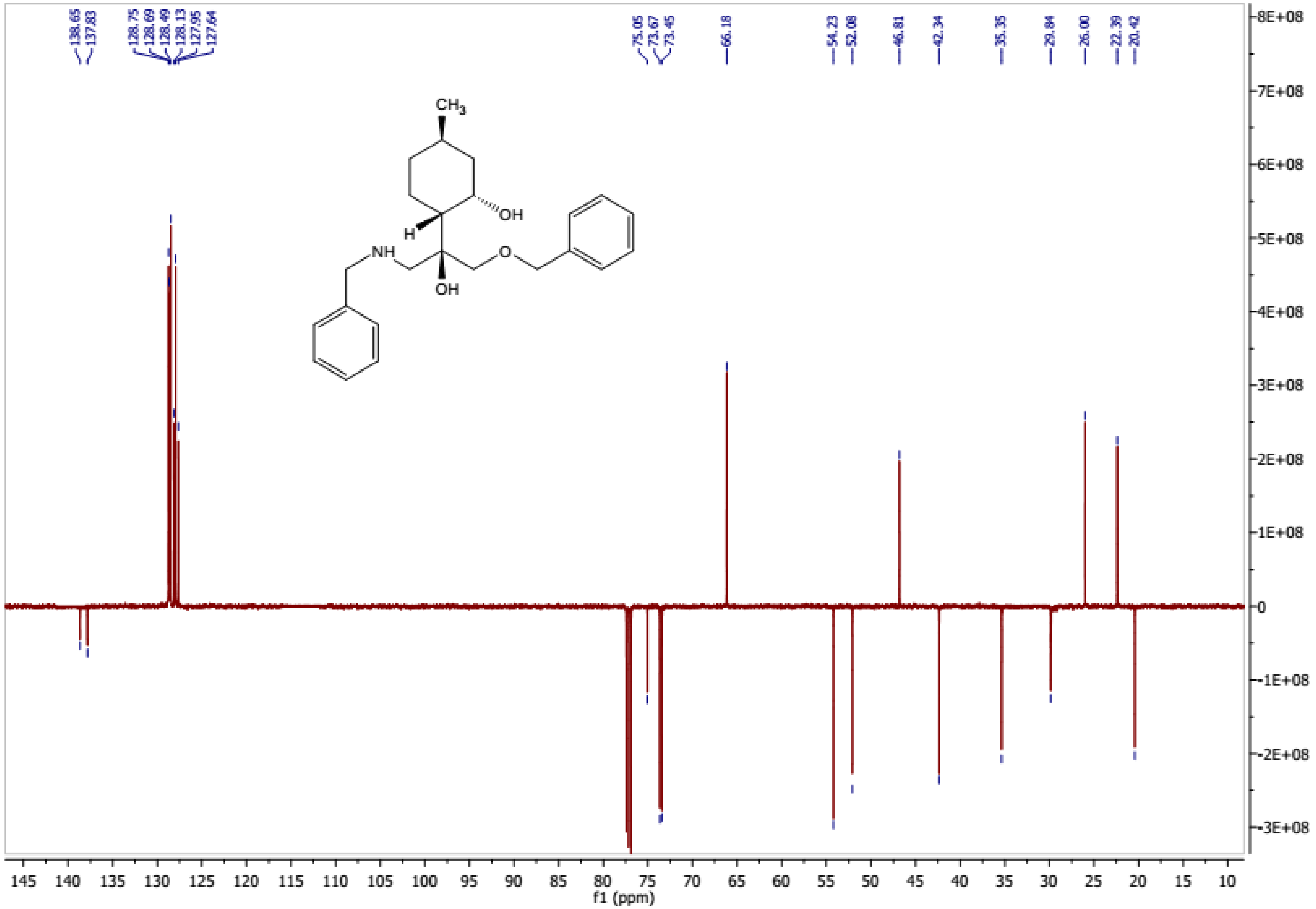

Figure S 74: ^{1}H-NMR of compound **26a**

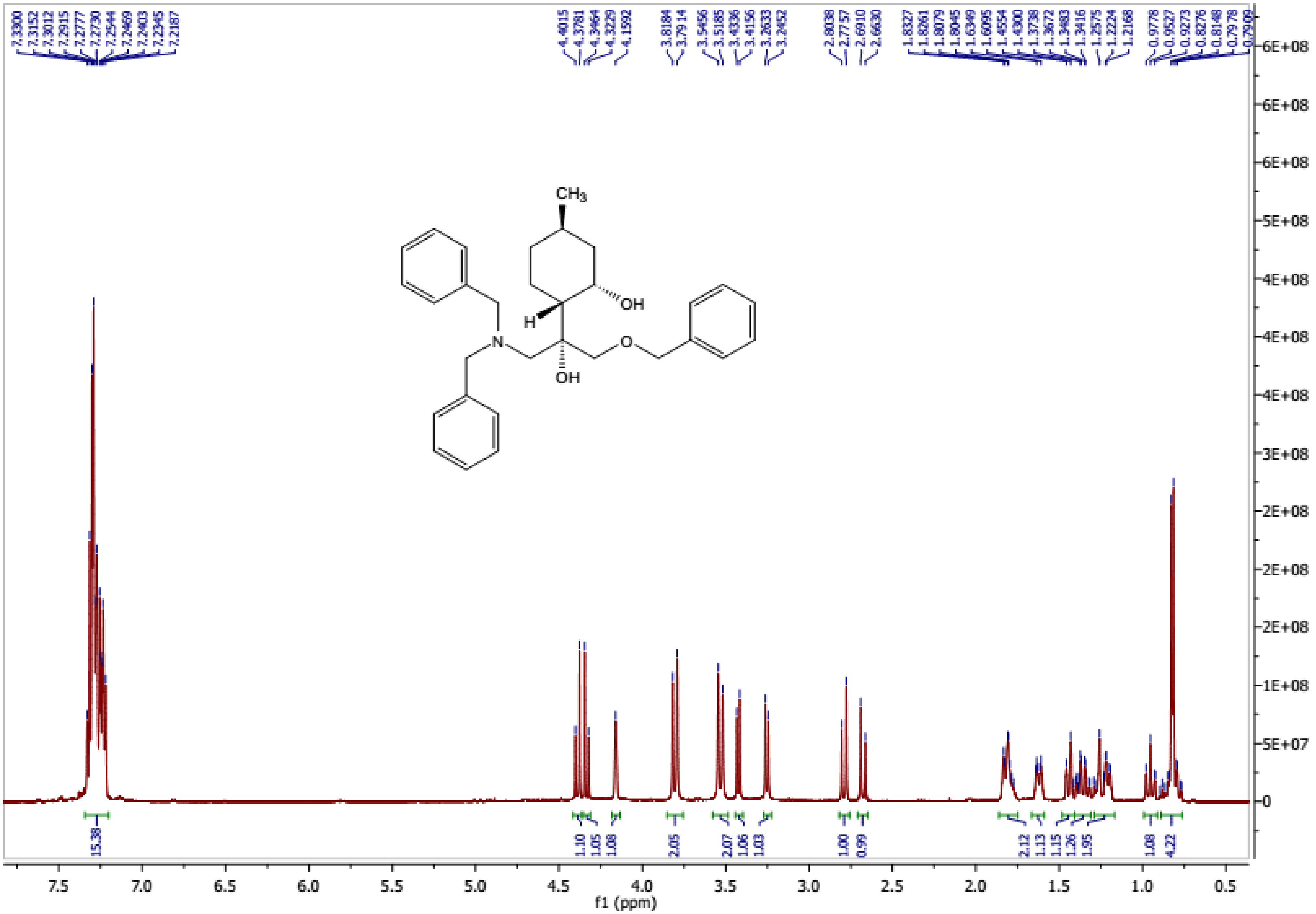

Figure S 75: ^{13}C-NMR of compound **26a**

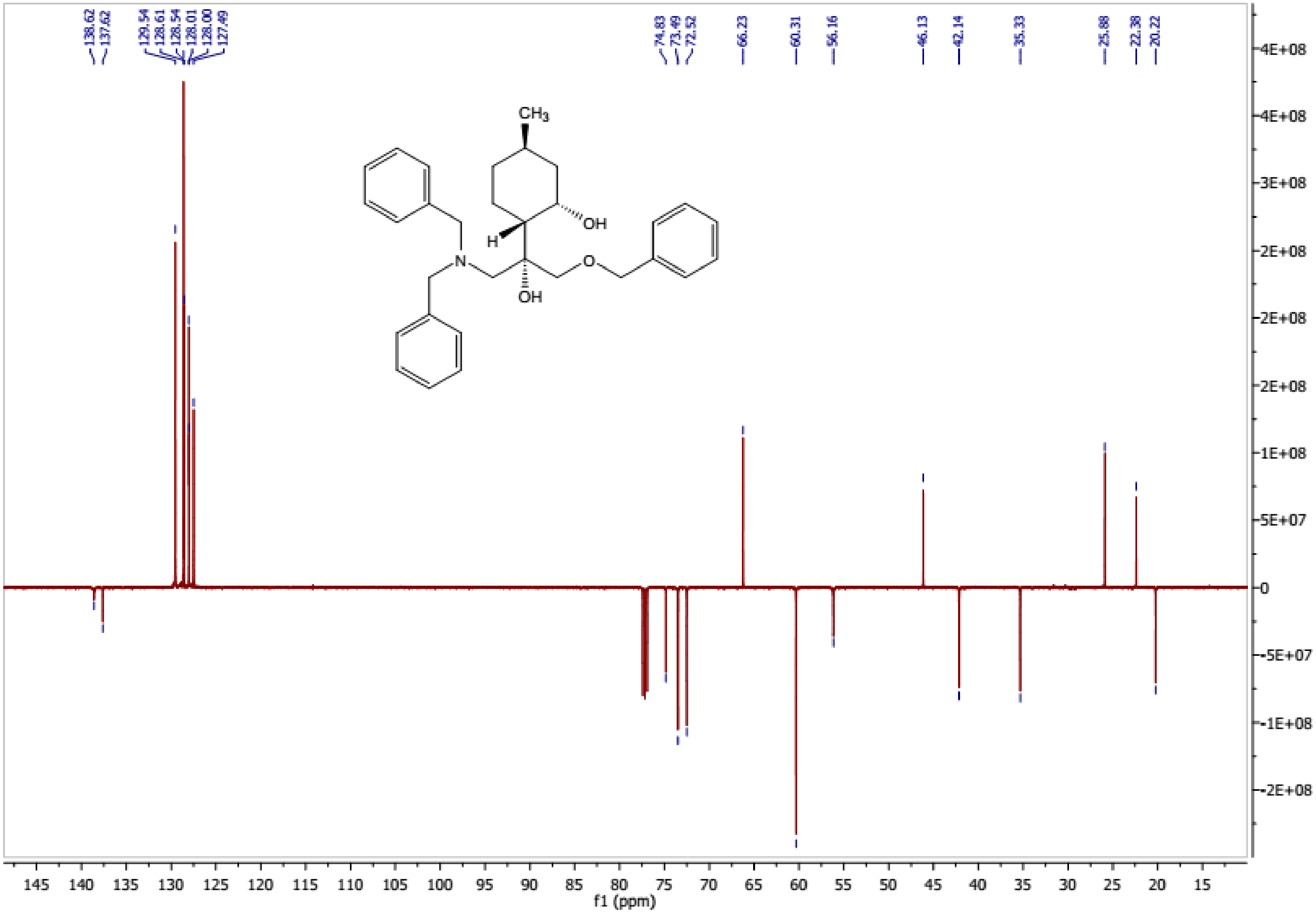

Figure S 76: ^{1}H-NMR of compound **26b**

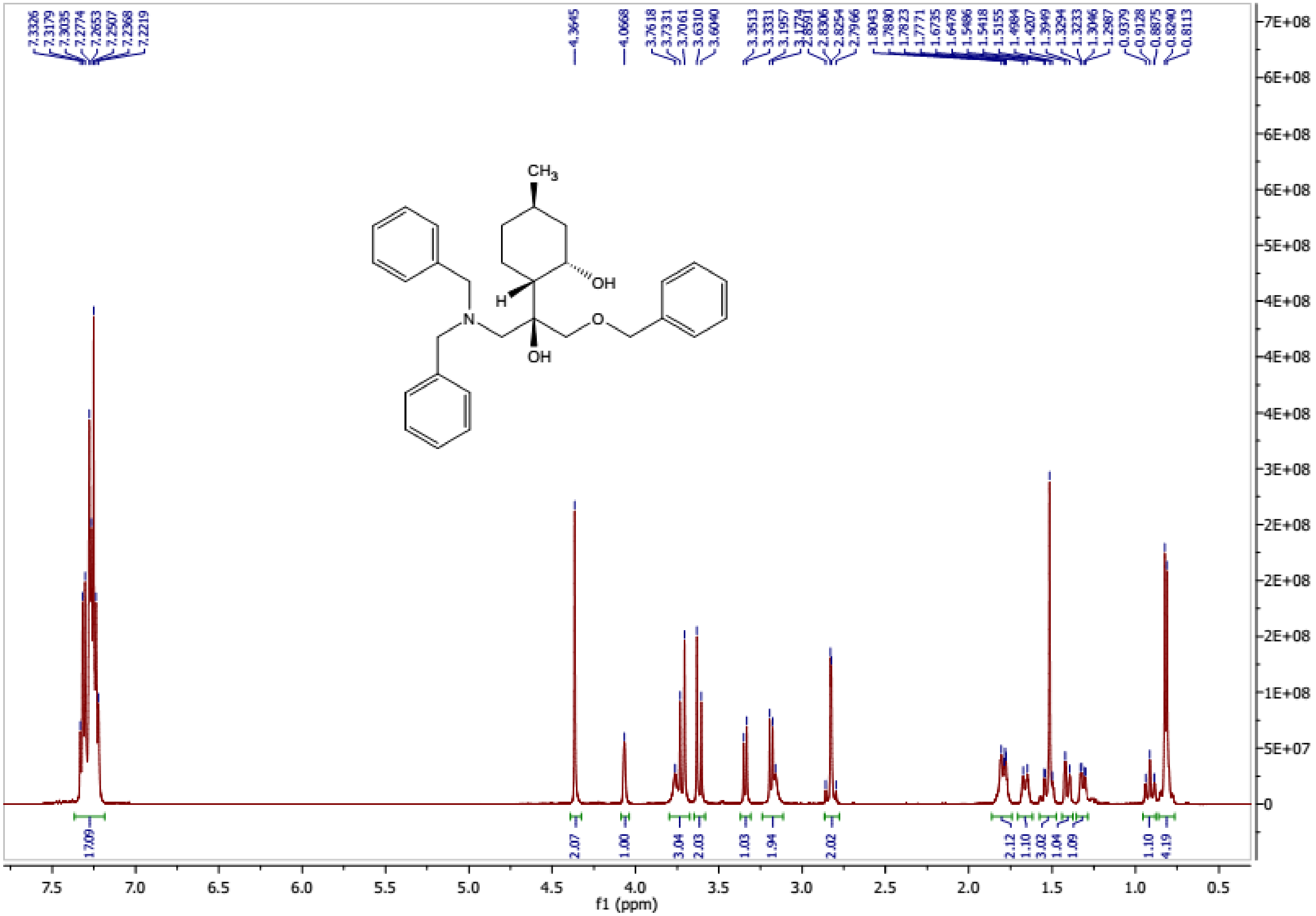

Figure S 77: ^{13}C-NMR of compound **26b**

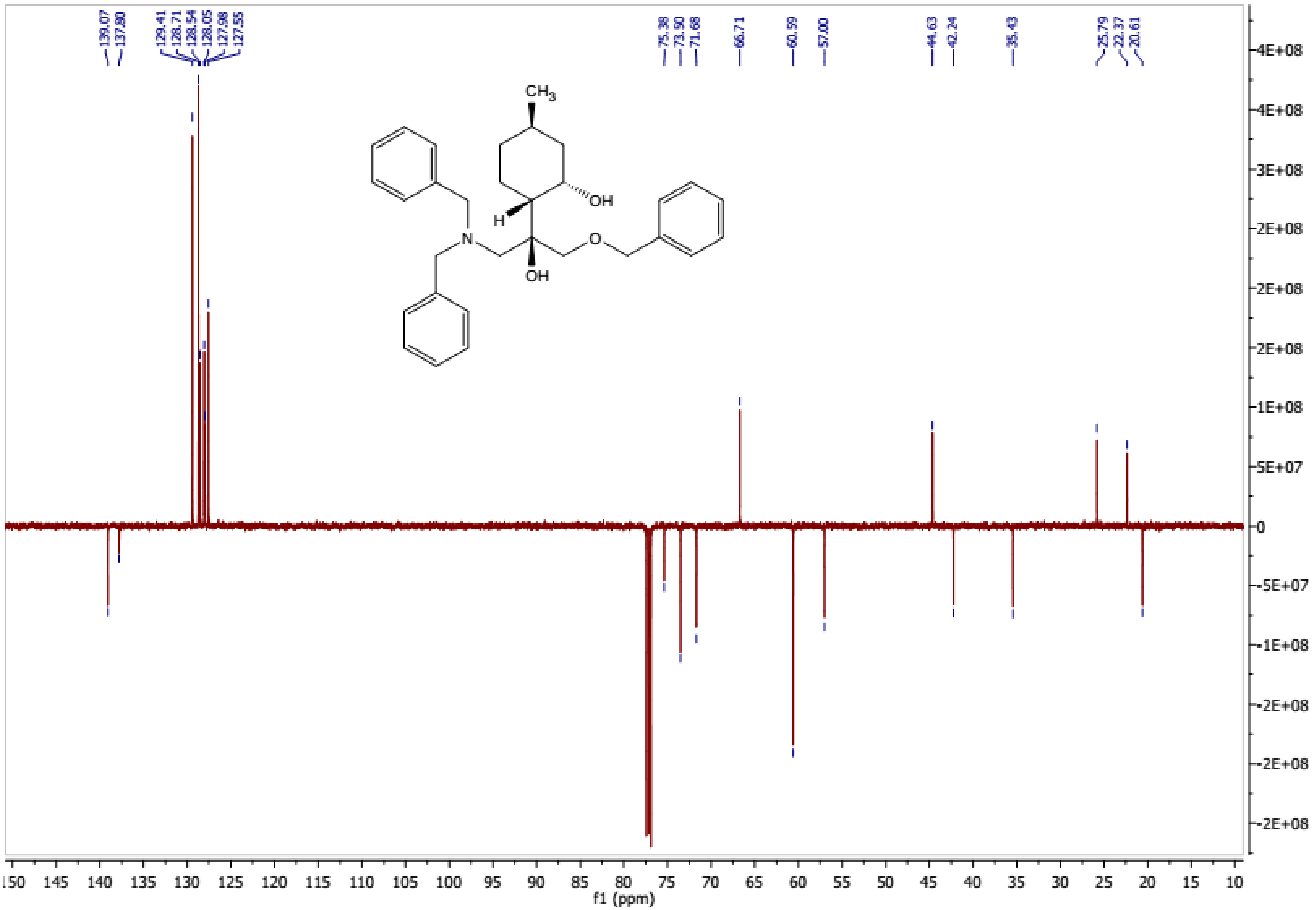

Figure S 78: ^{1}H-NMR of compound **27a**

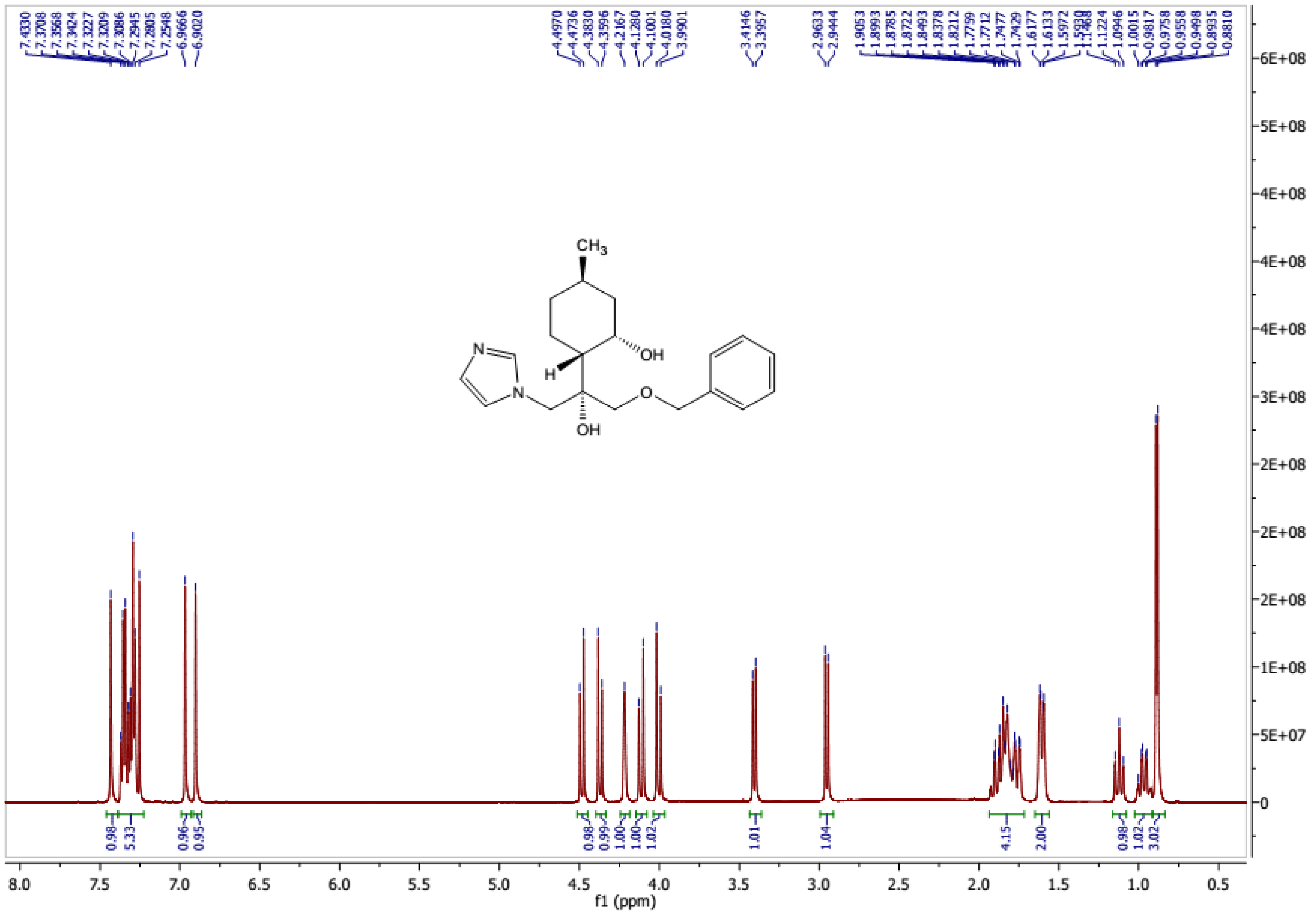

Figure S 79: ^{13}C-NMR of compound **27a**

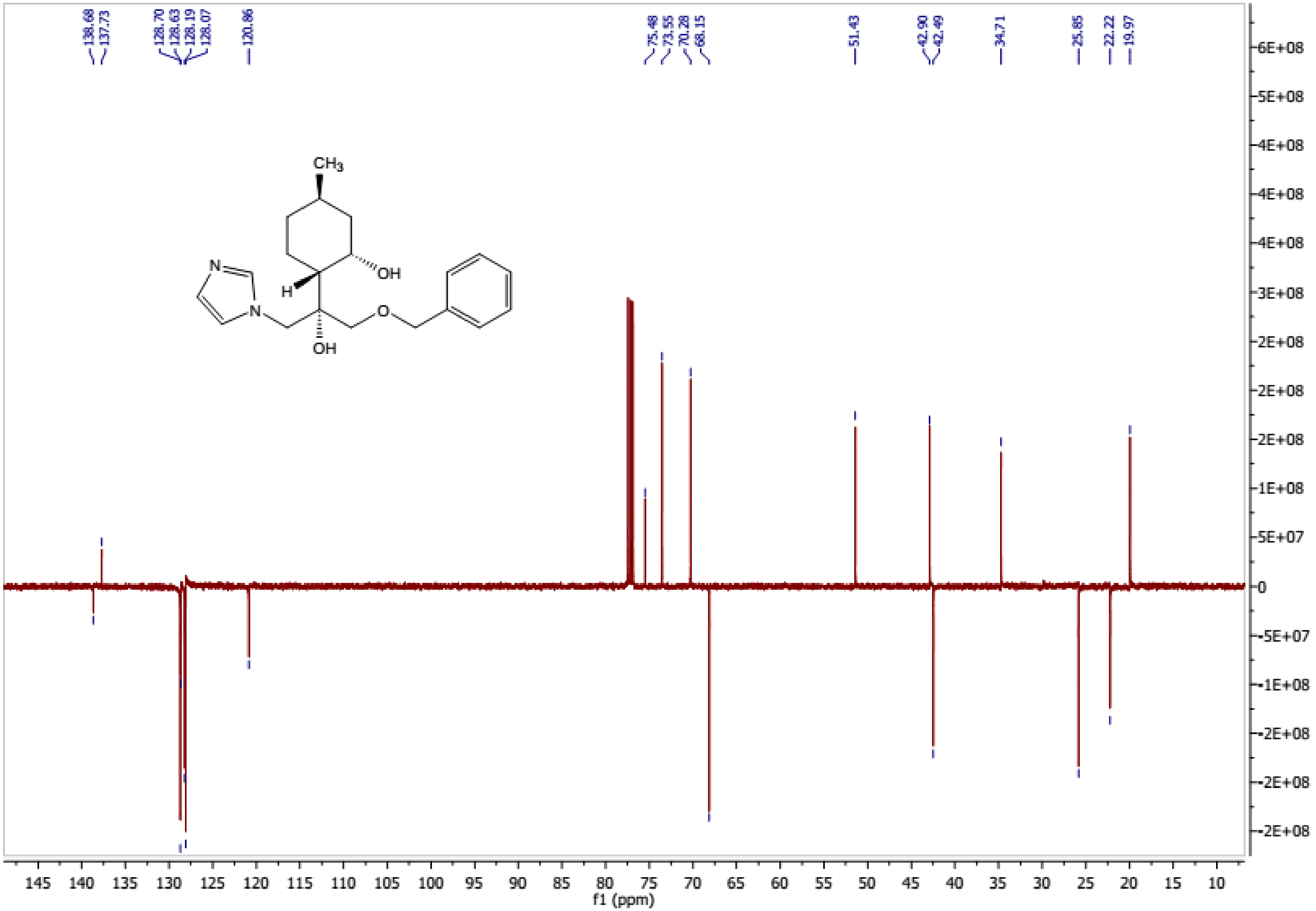

Figure S 80: ^{1}H-NMR of compound **27b**

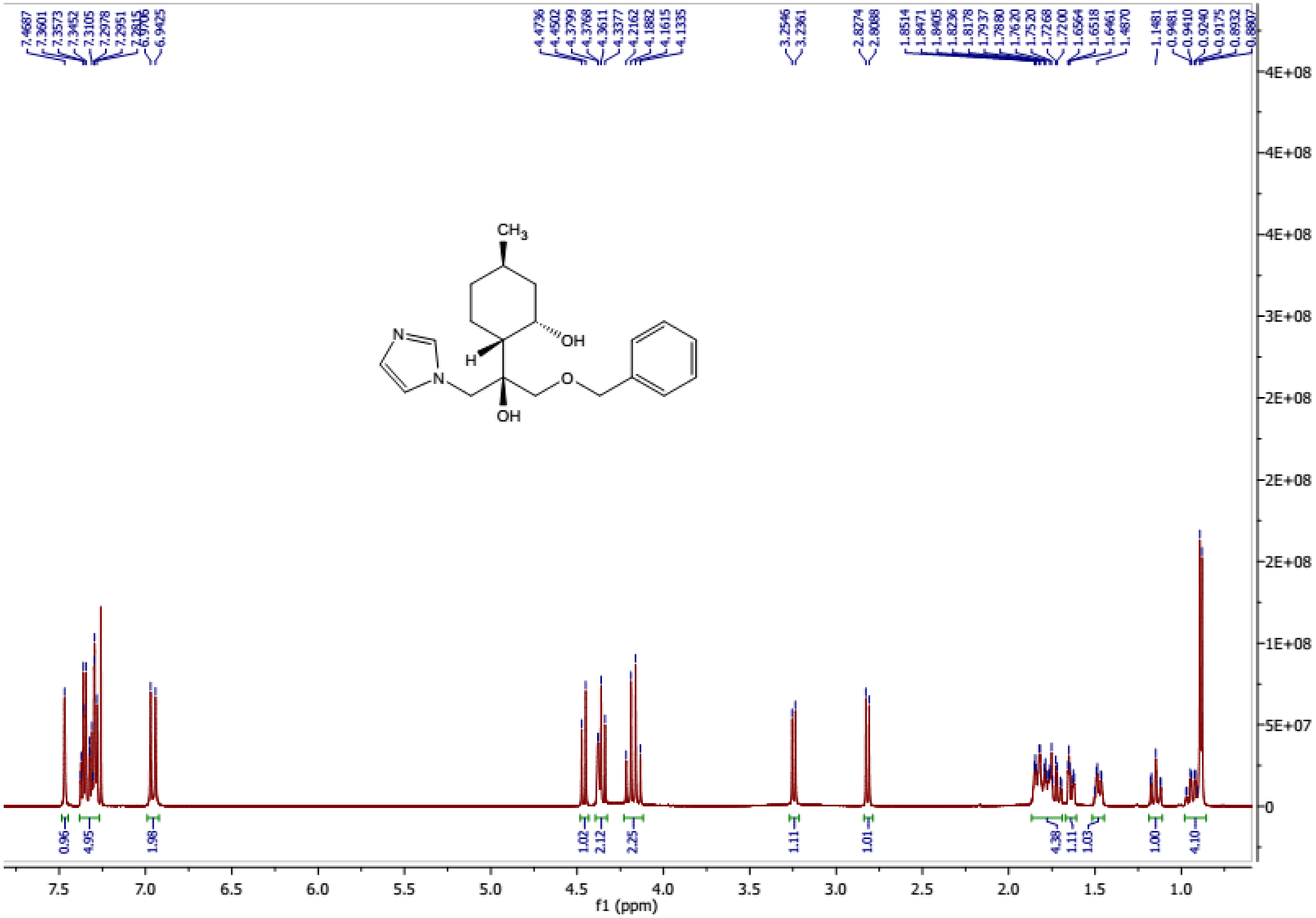

Figure S 81: ^{13}C-NMR of compound **27b**

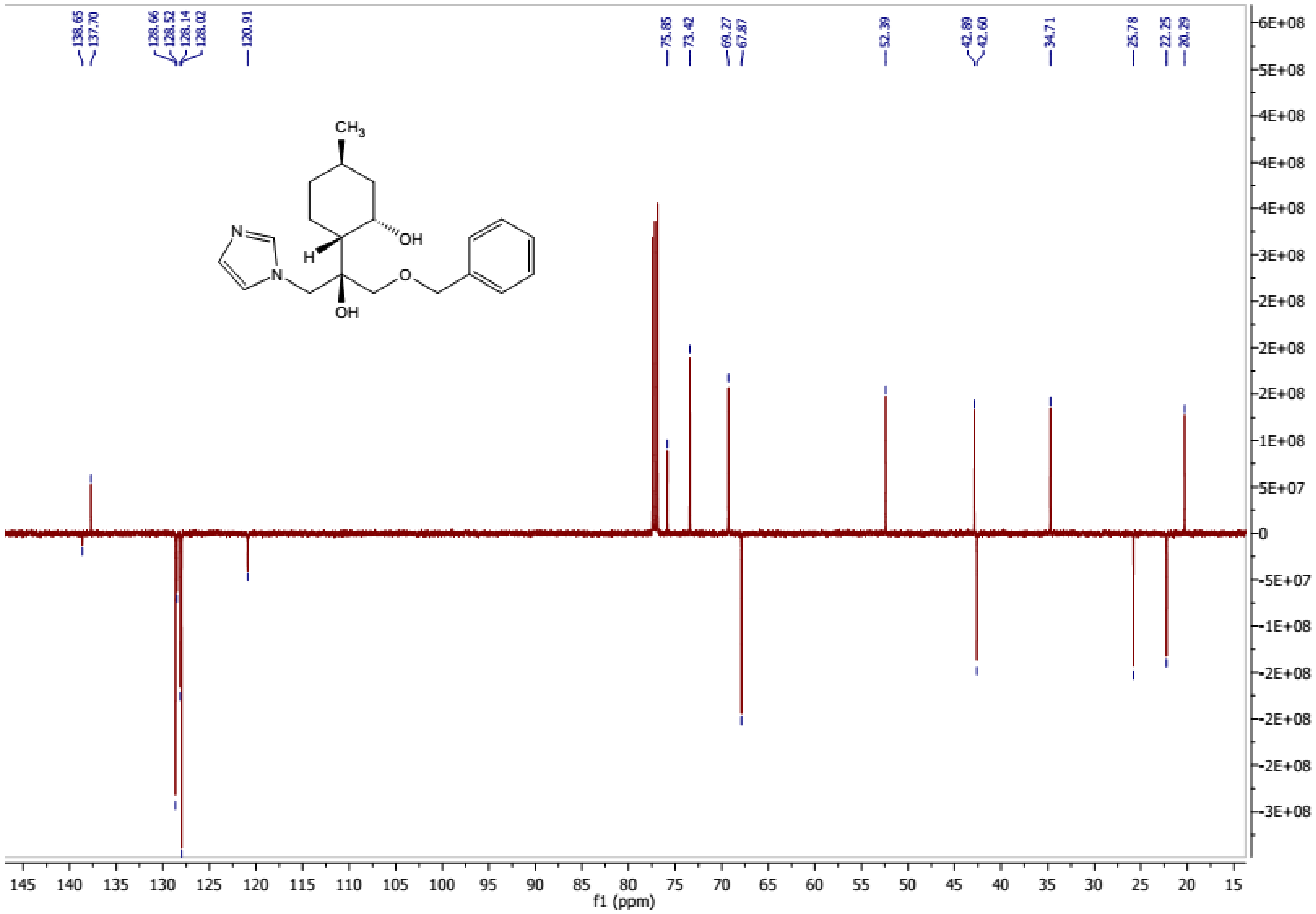

Figure S 82: ^{1}H-NMR of compound **28a**

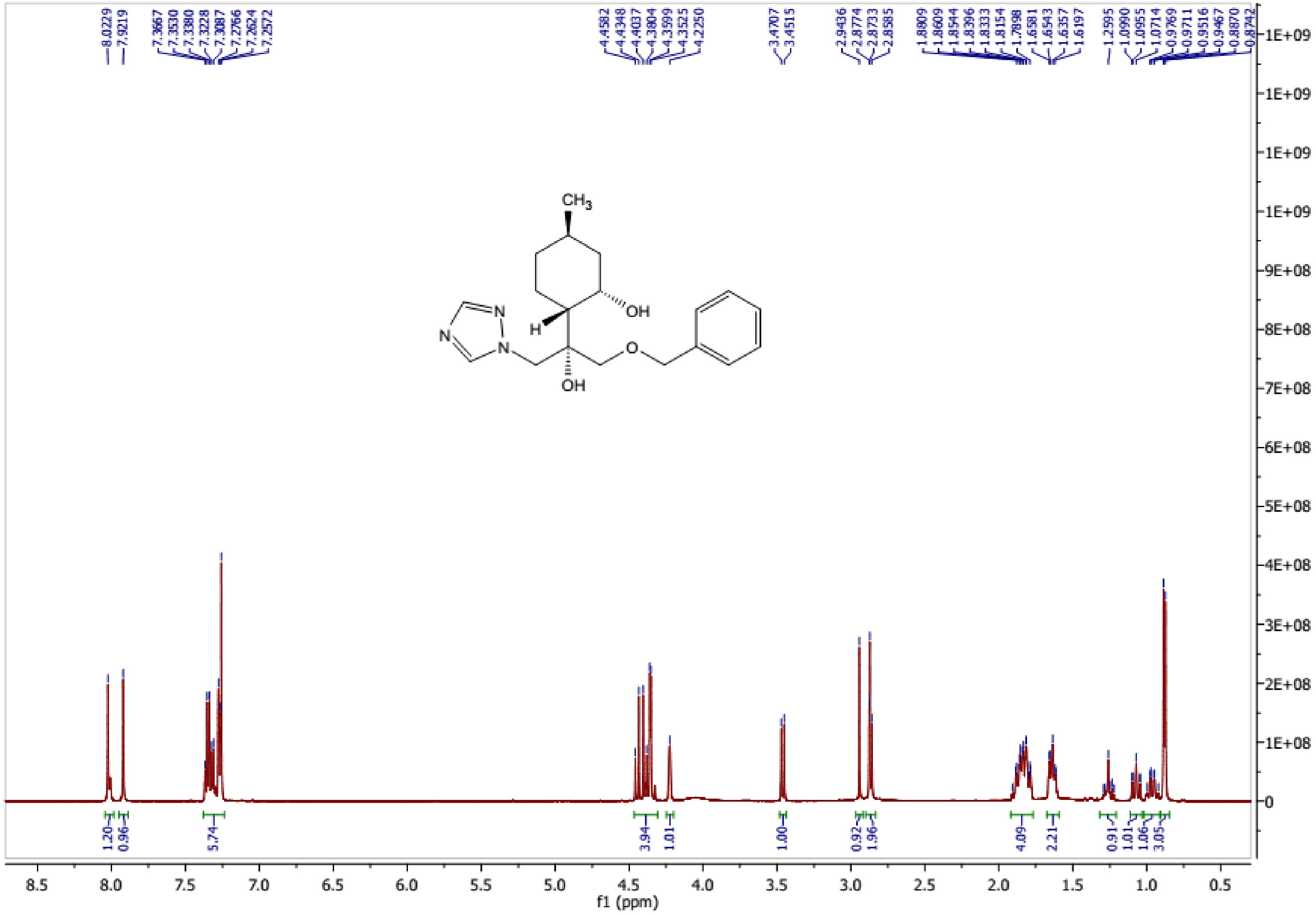

Figure S 83: ^{13}C-NMR of compound **28a**

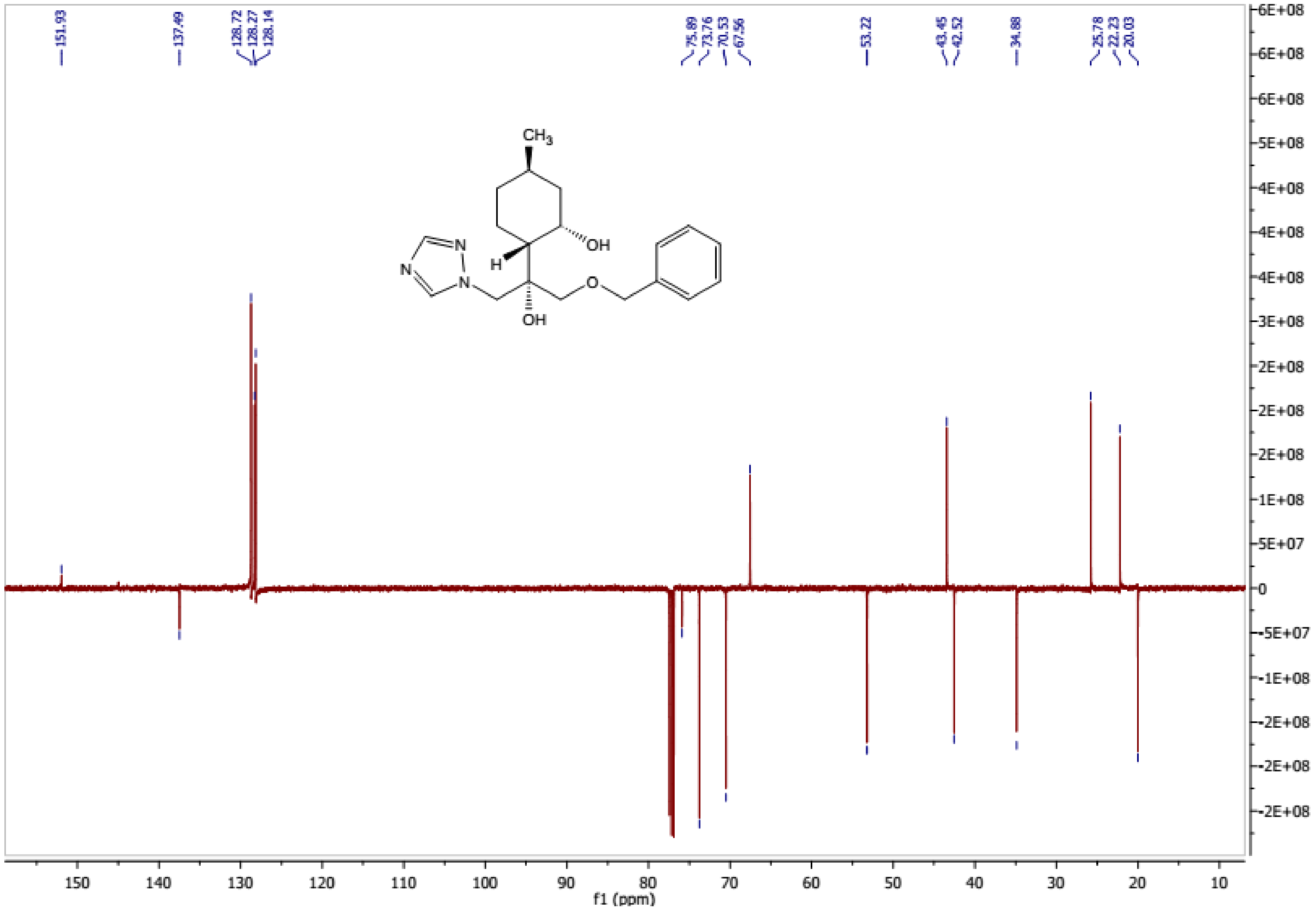

Figure S 84: ^{1}H-NMR of compound **28b**

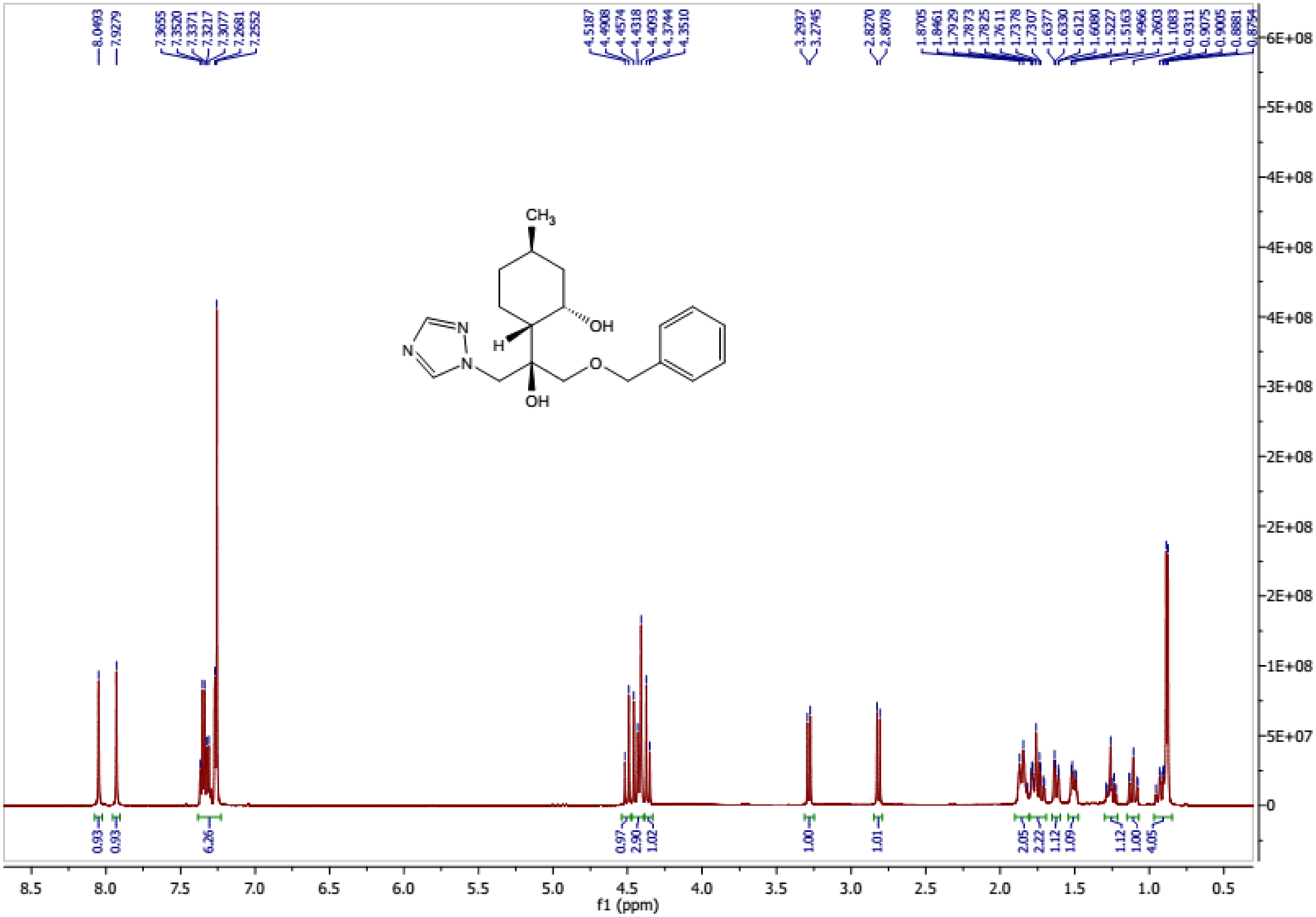

Figure S 85: ^{13}C-NMR of compound **28b**

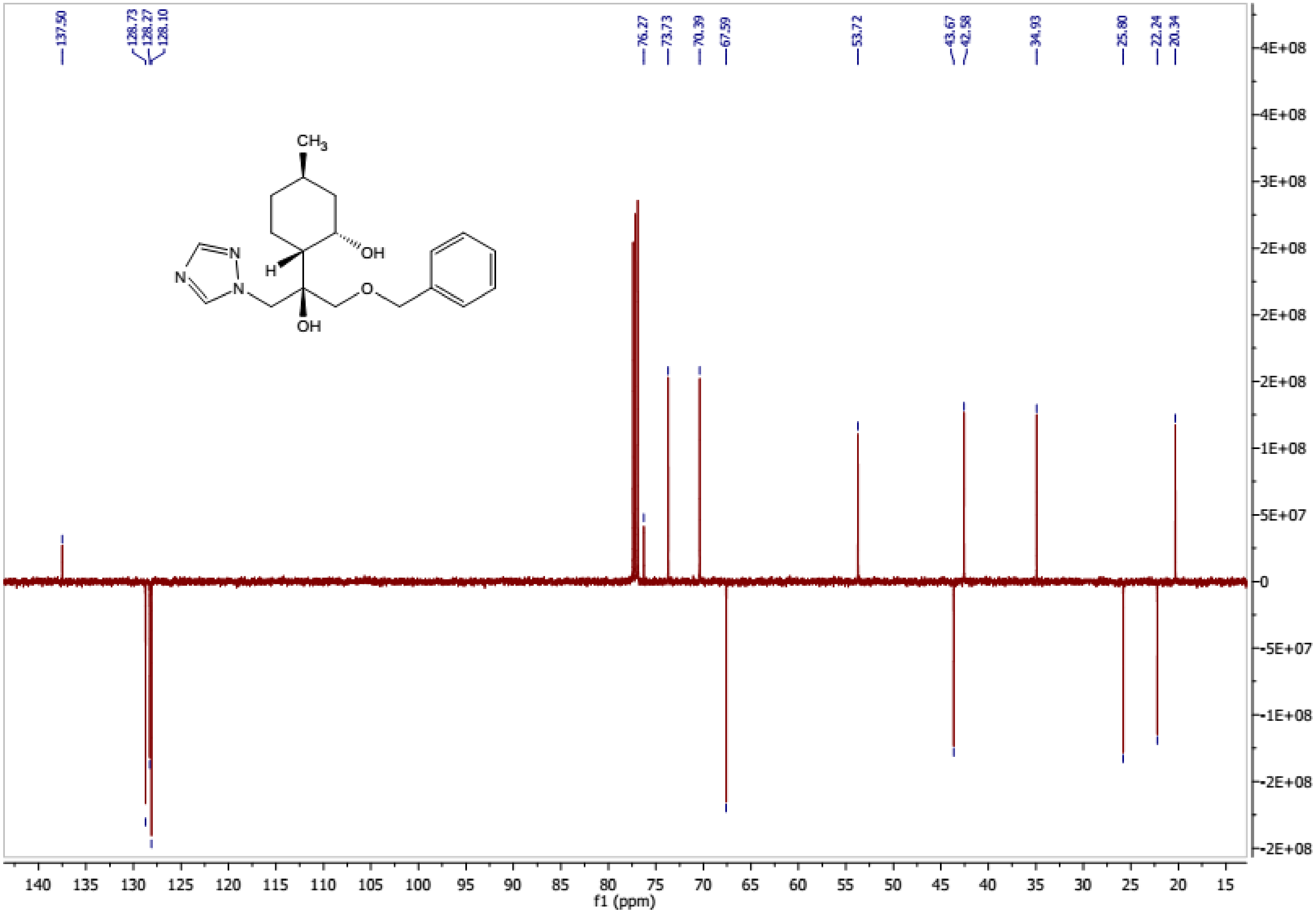

Figure S 86: ^{1}H-NMR of compound **30b**

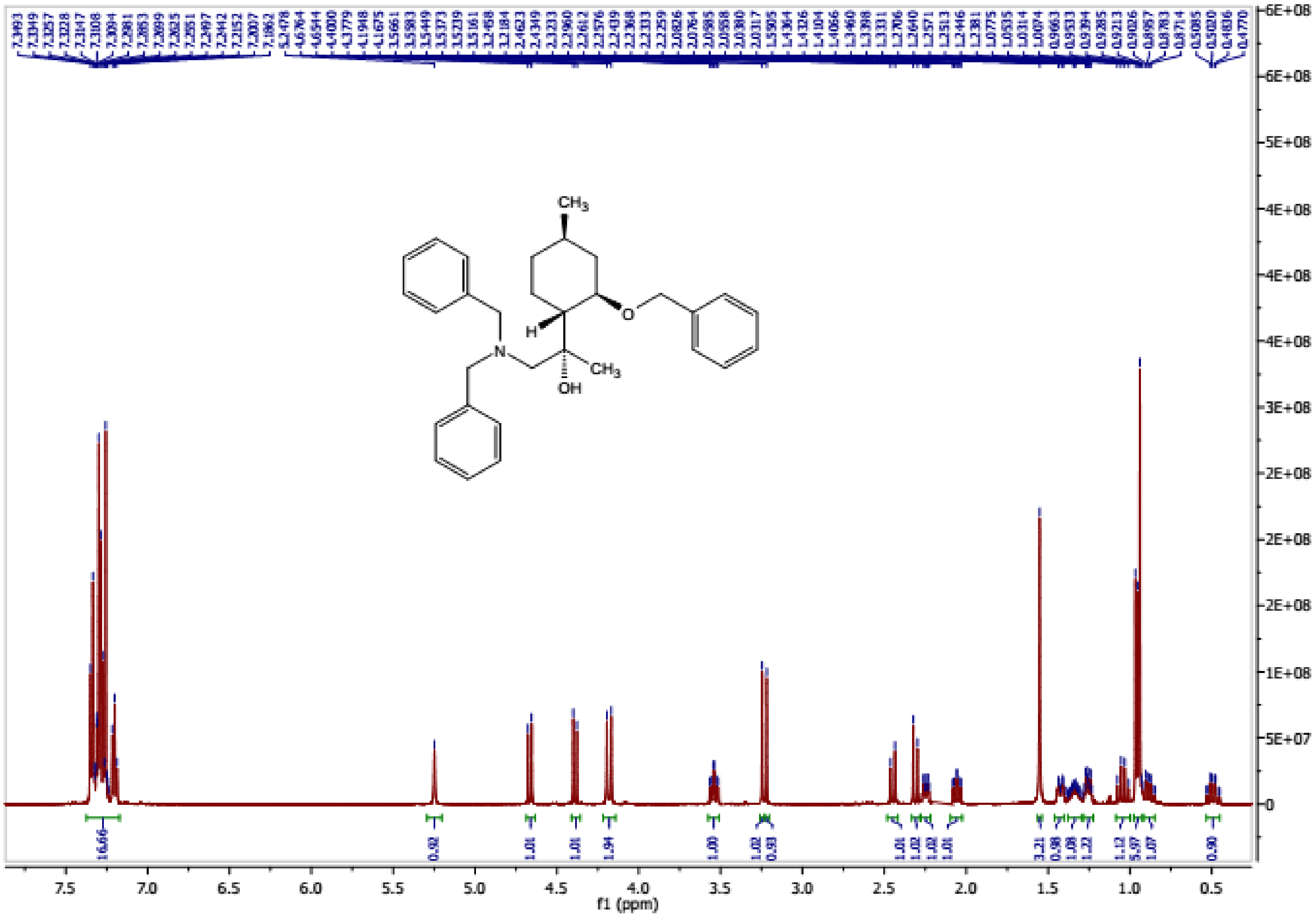

Figure S 87: ^{13}C-NMR of compound **30b**

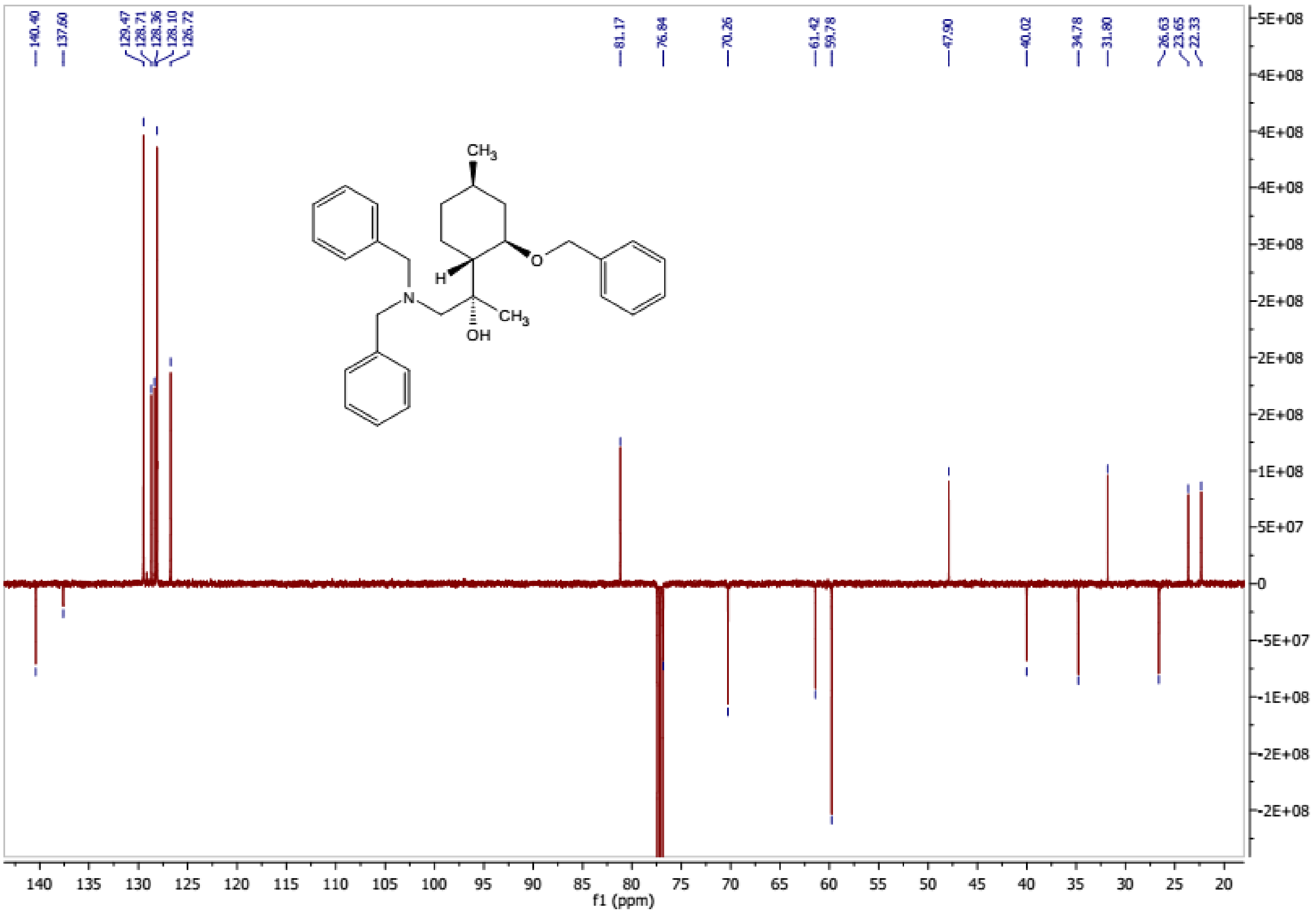

Figure S 88: ^{1}H-NMR of compound **31a**

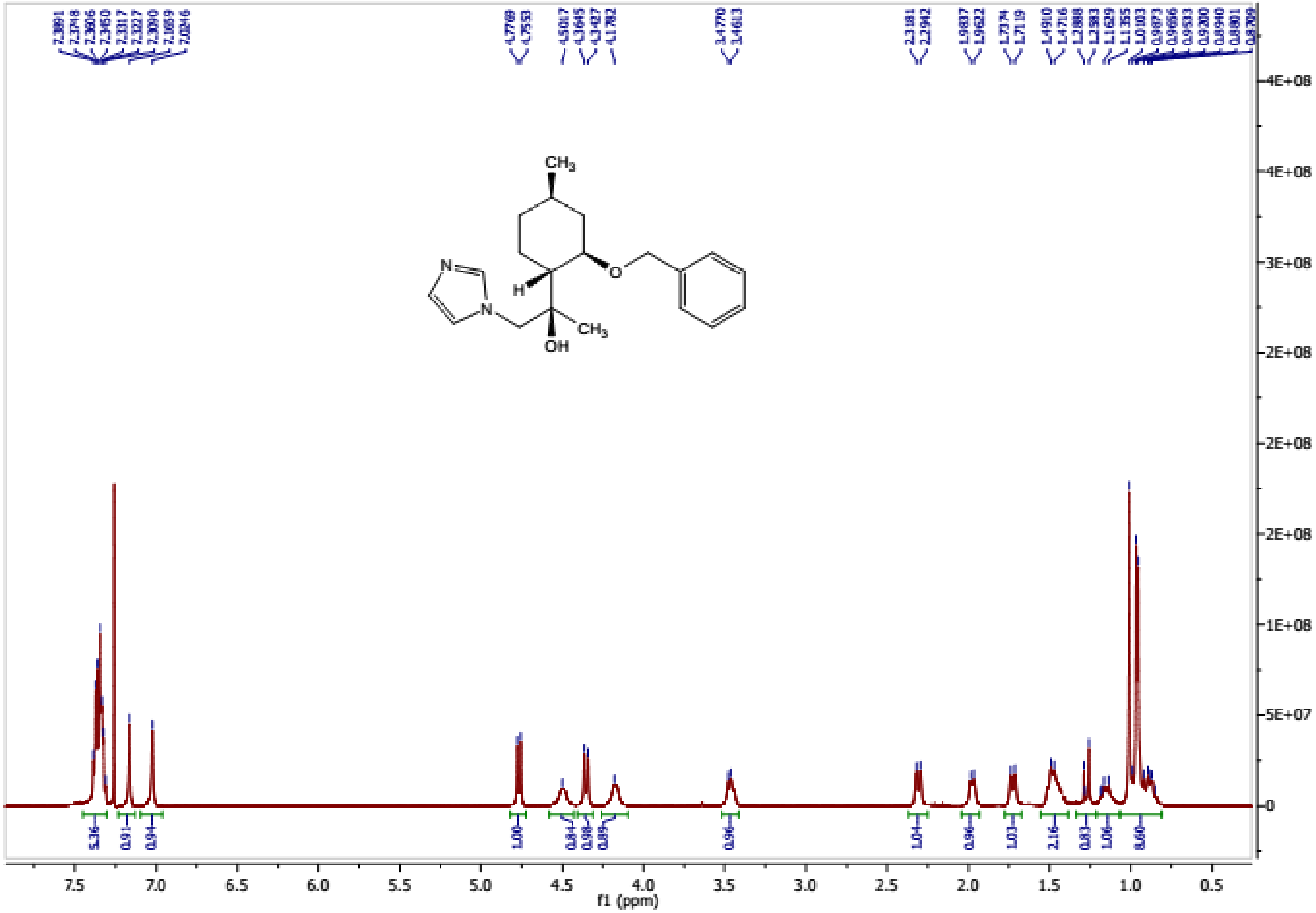

Figure S 89: ^{13}C-NMR of compound **31a**

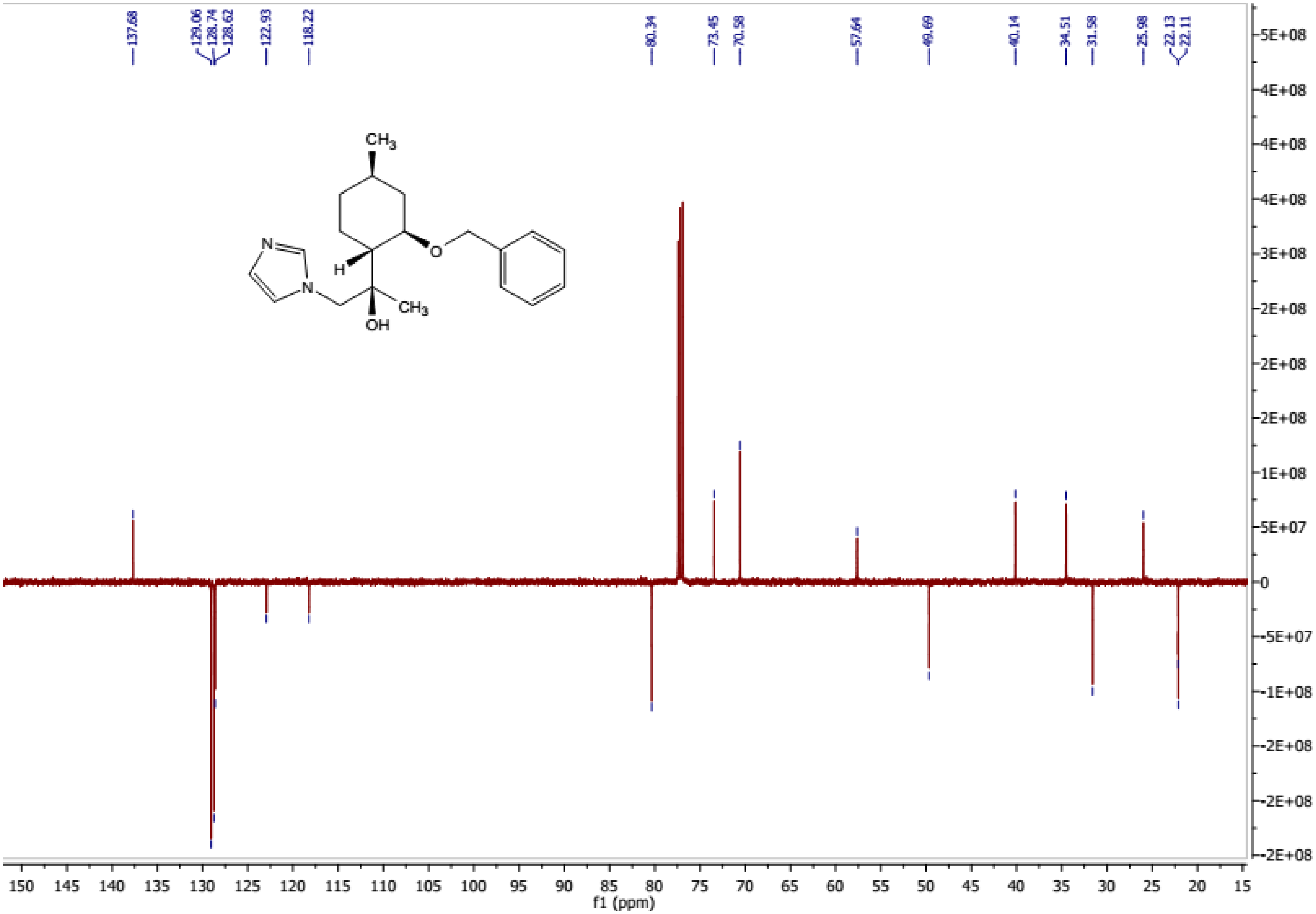

Figure S 90: ^{1}H-NMR of compound **31b**

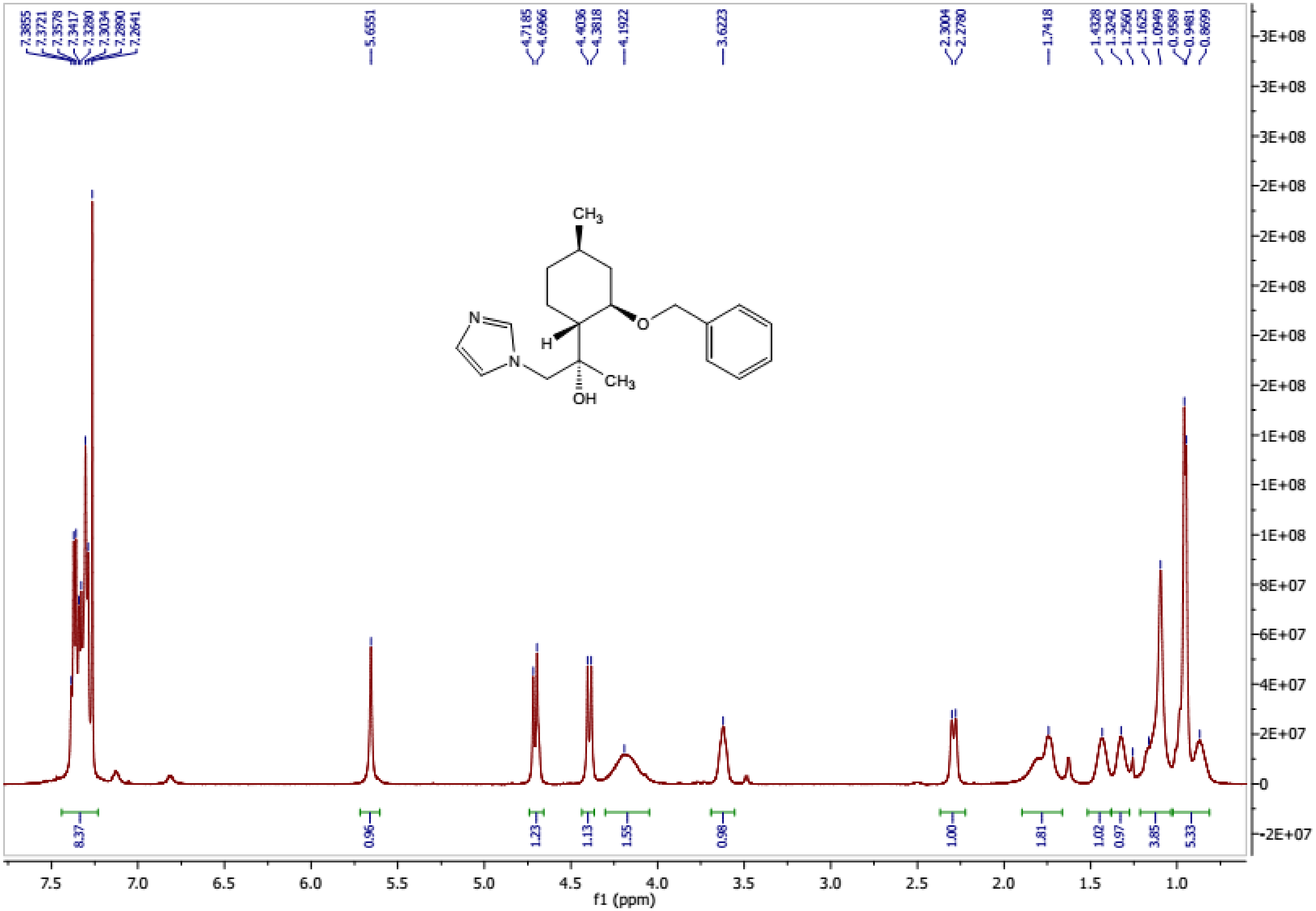

Figure S 91: ^{13}C-NMR of compound **31b**

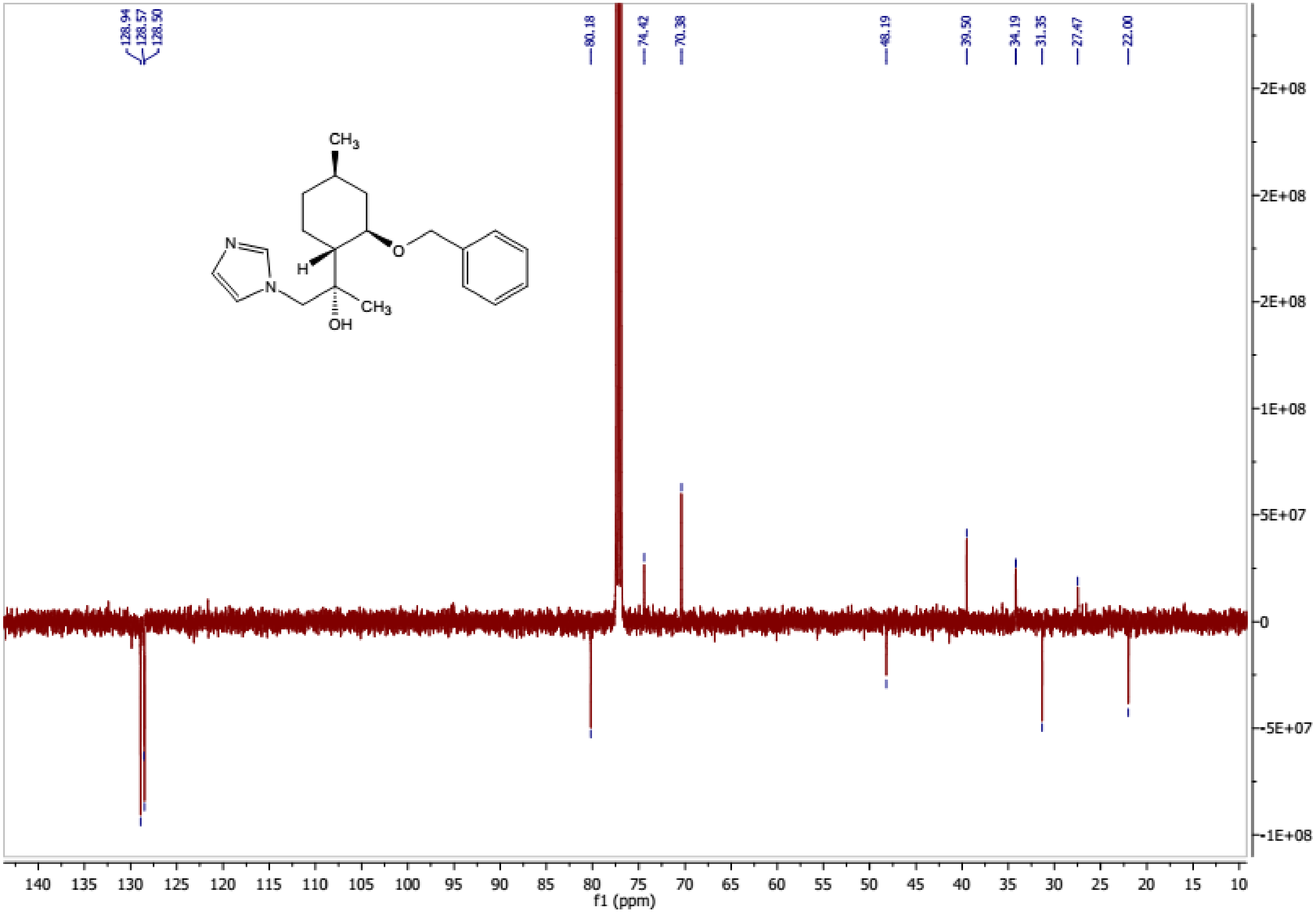

Figure S 92: ^{1}H-NMR of compound **32a**

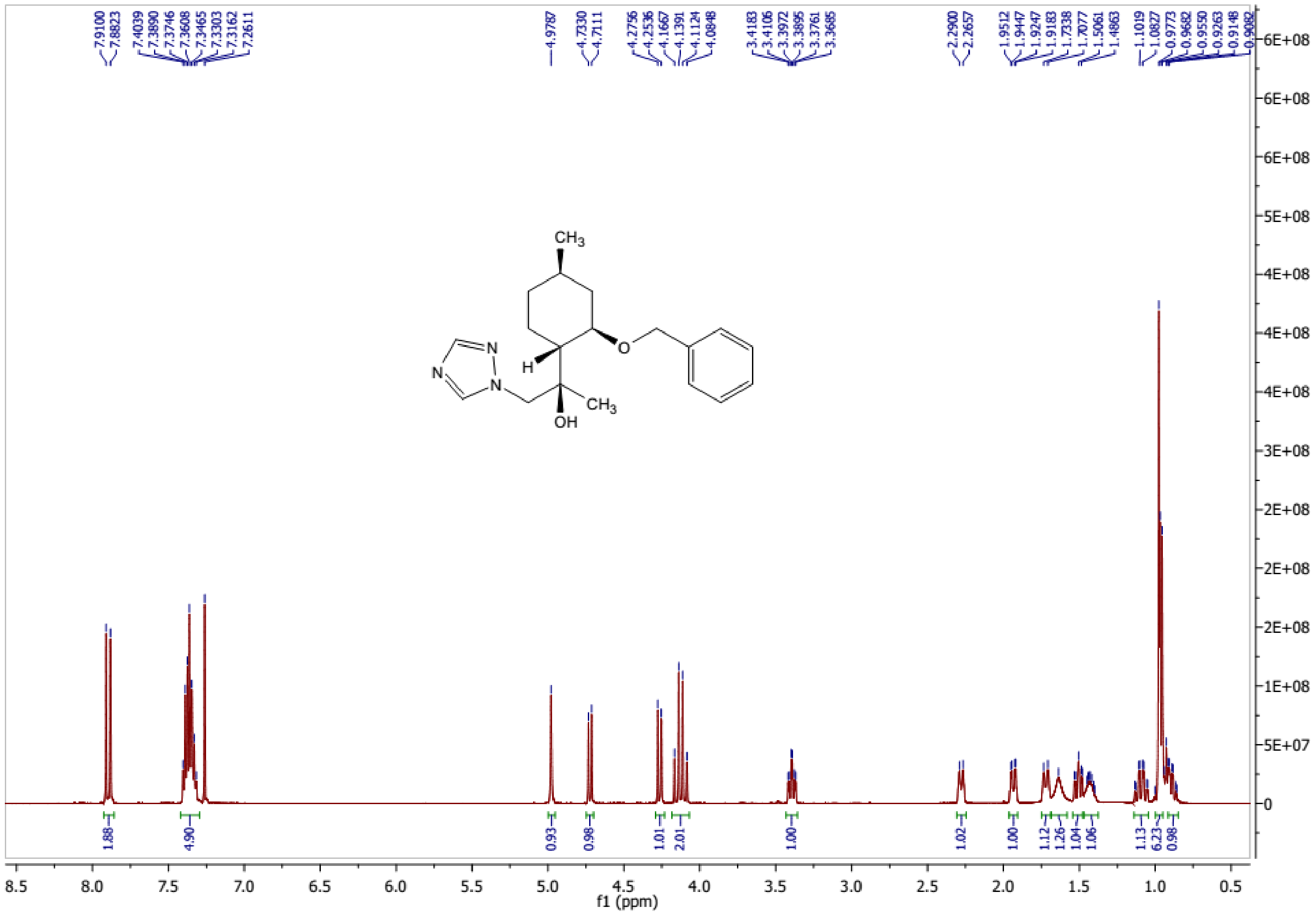

Figure S 93: ^{13}C-NMR of compound **32a**

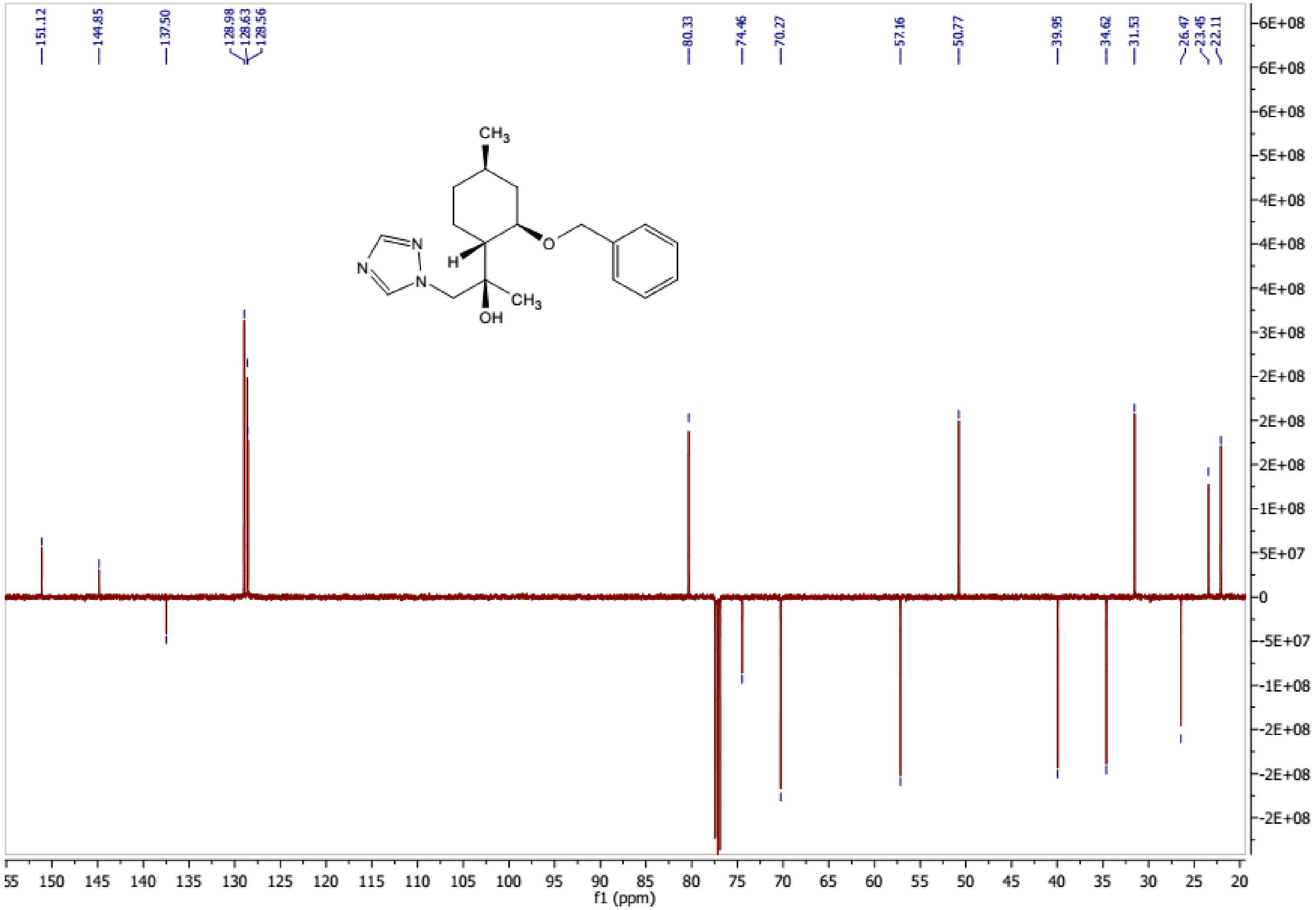

Figure S 94: ^{1}H-NMR of compound **32b**

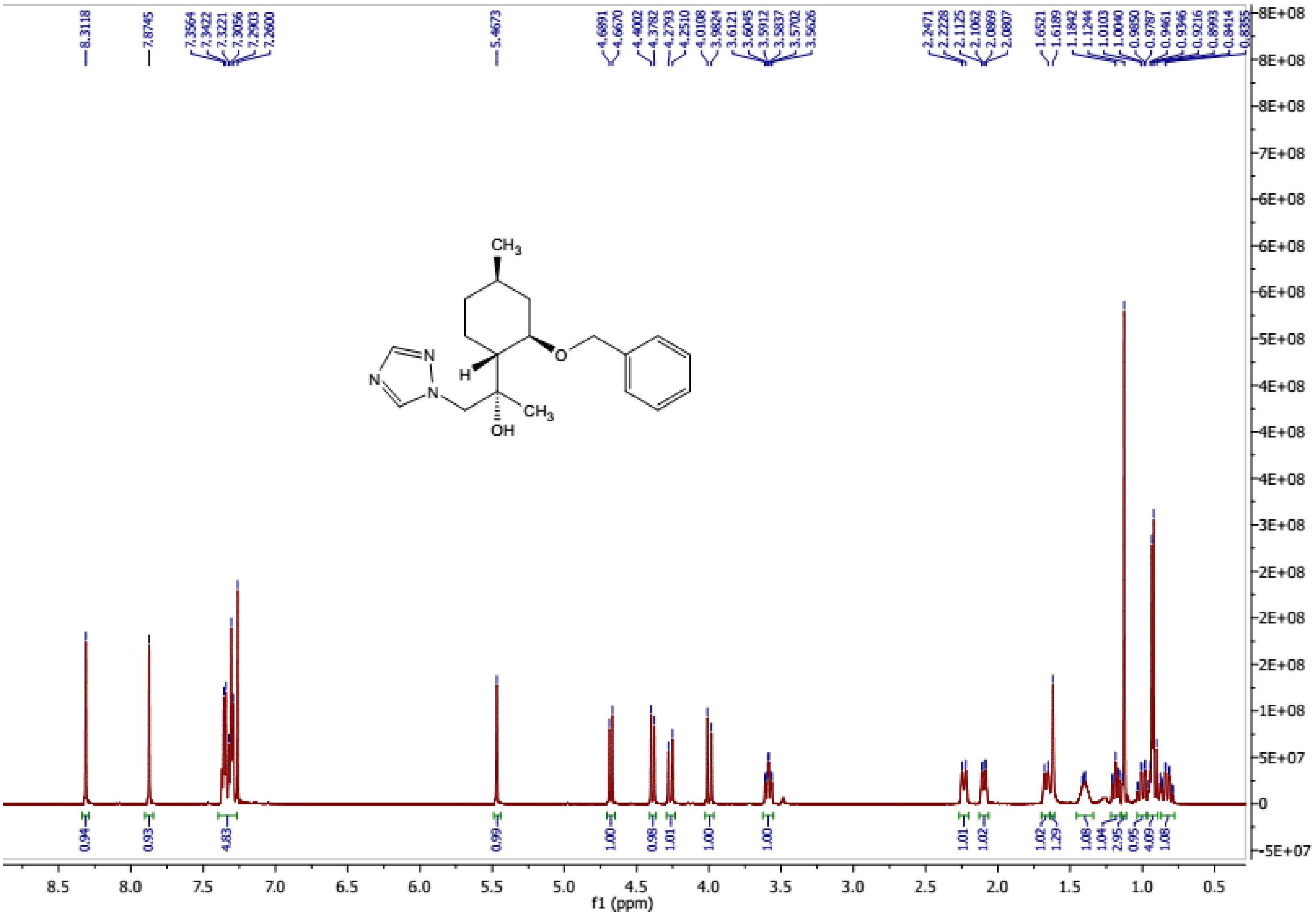

Figure S 95: ^{13}C-NMR of compound **32b**

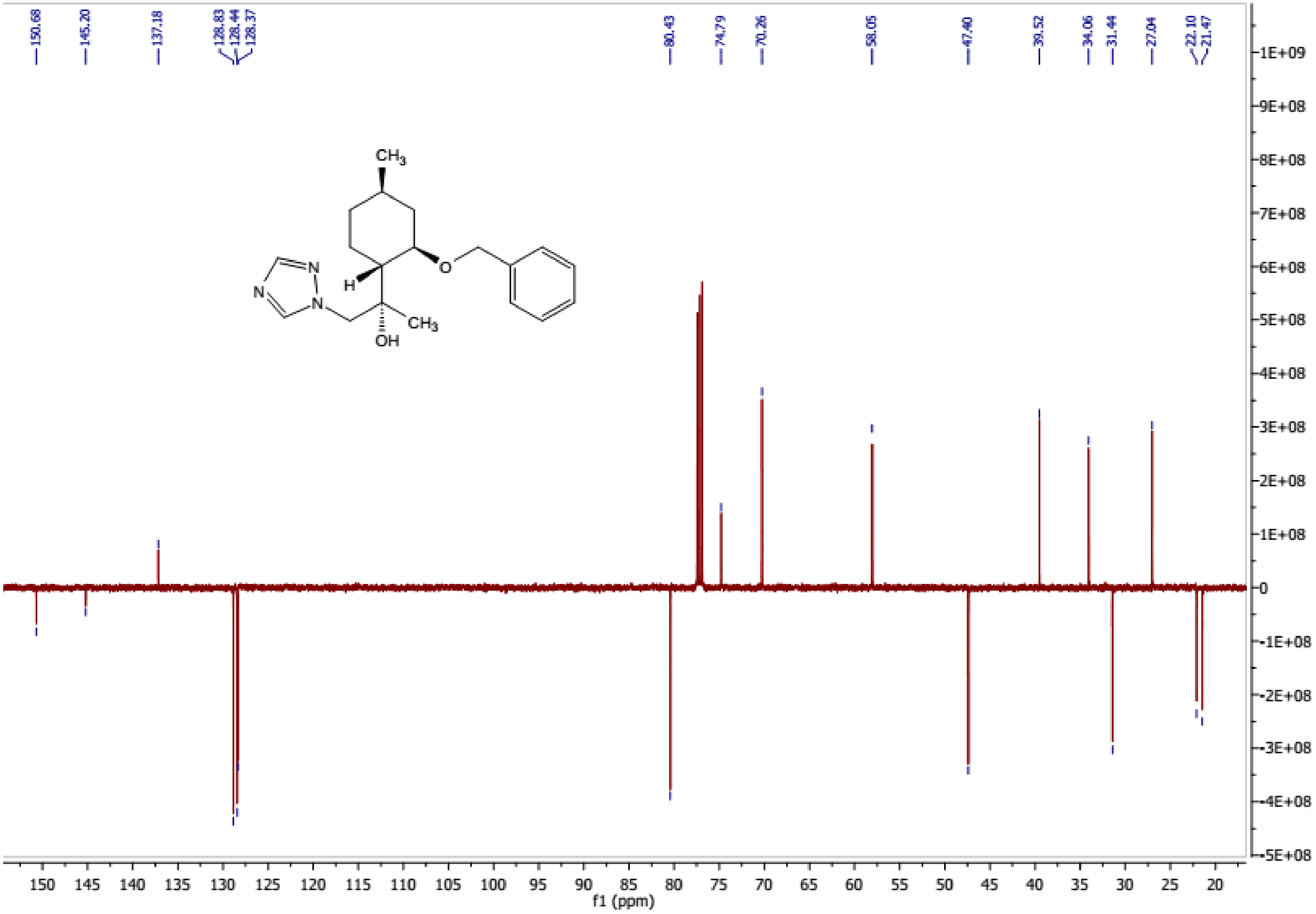

Figure S 96: ^{1}H-NMR of compound **34a**

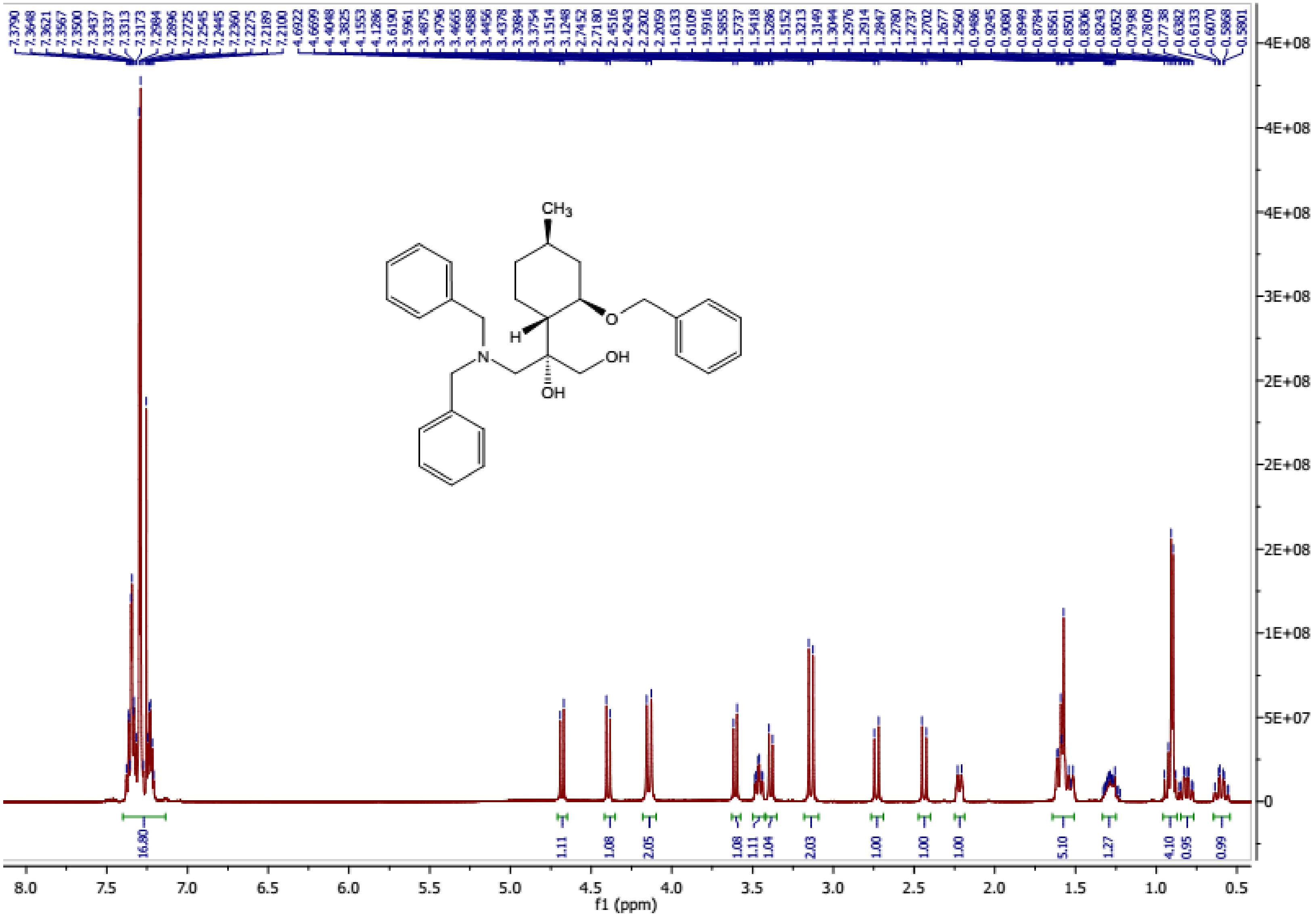

Figure S 97: ^{13}C-NMR of compound **34a**

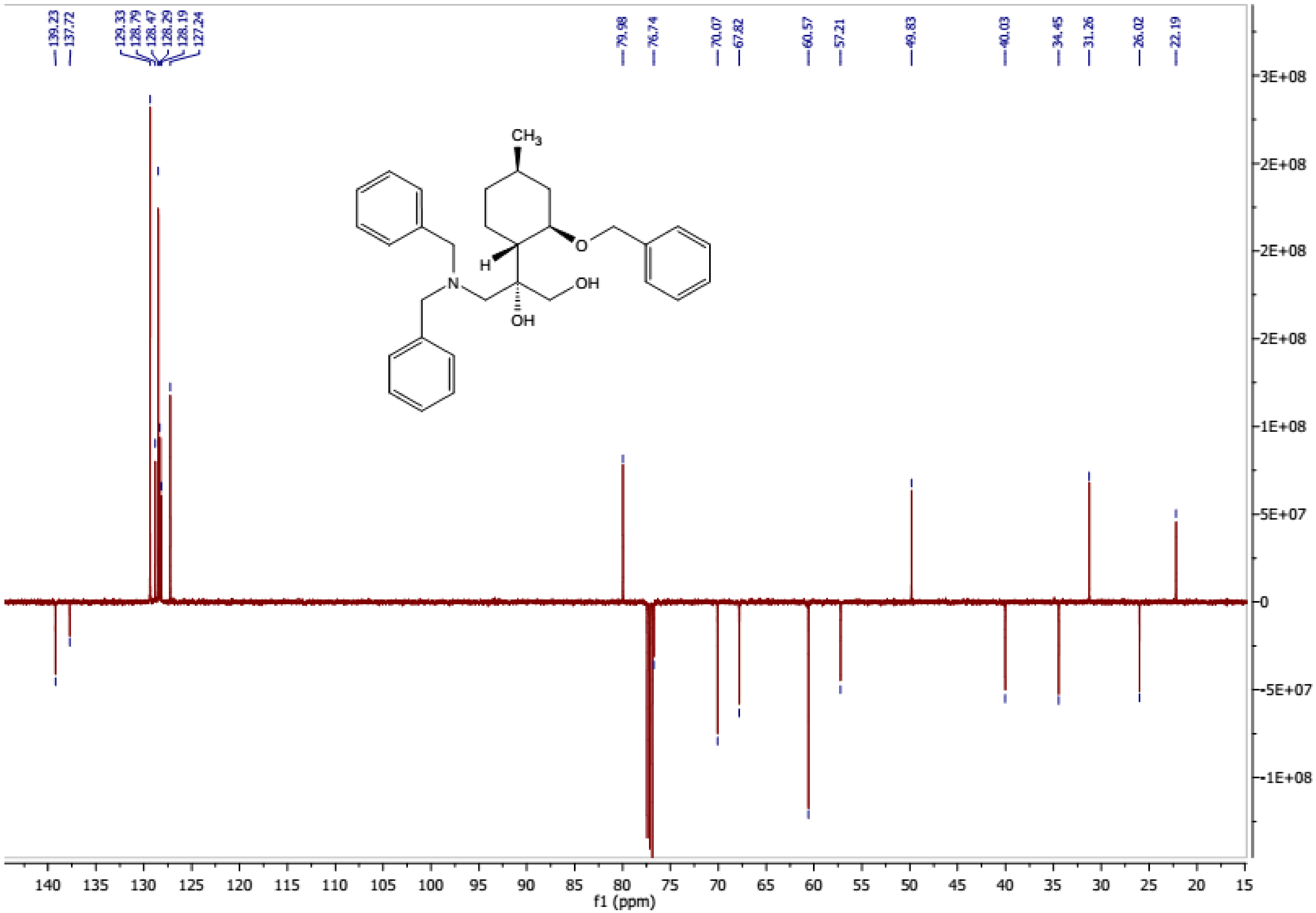

Figure S 98: ^{1}H-NMR of compound **35a**

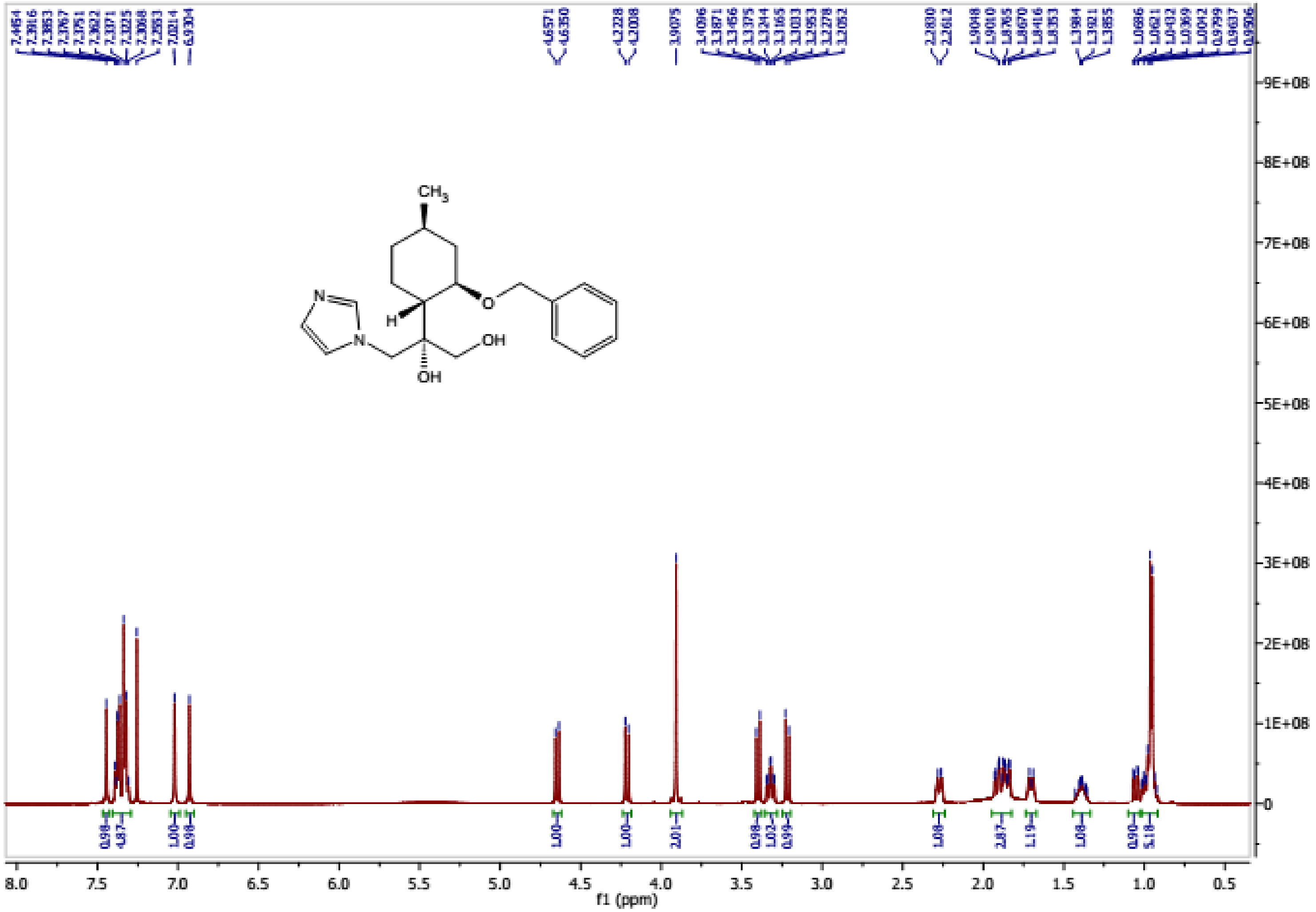

Figure S 99: ^{13}C-NMR of compound **35a**

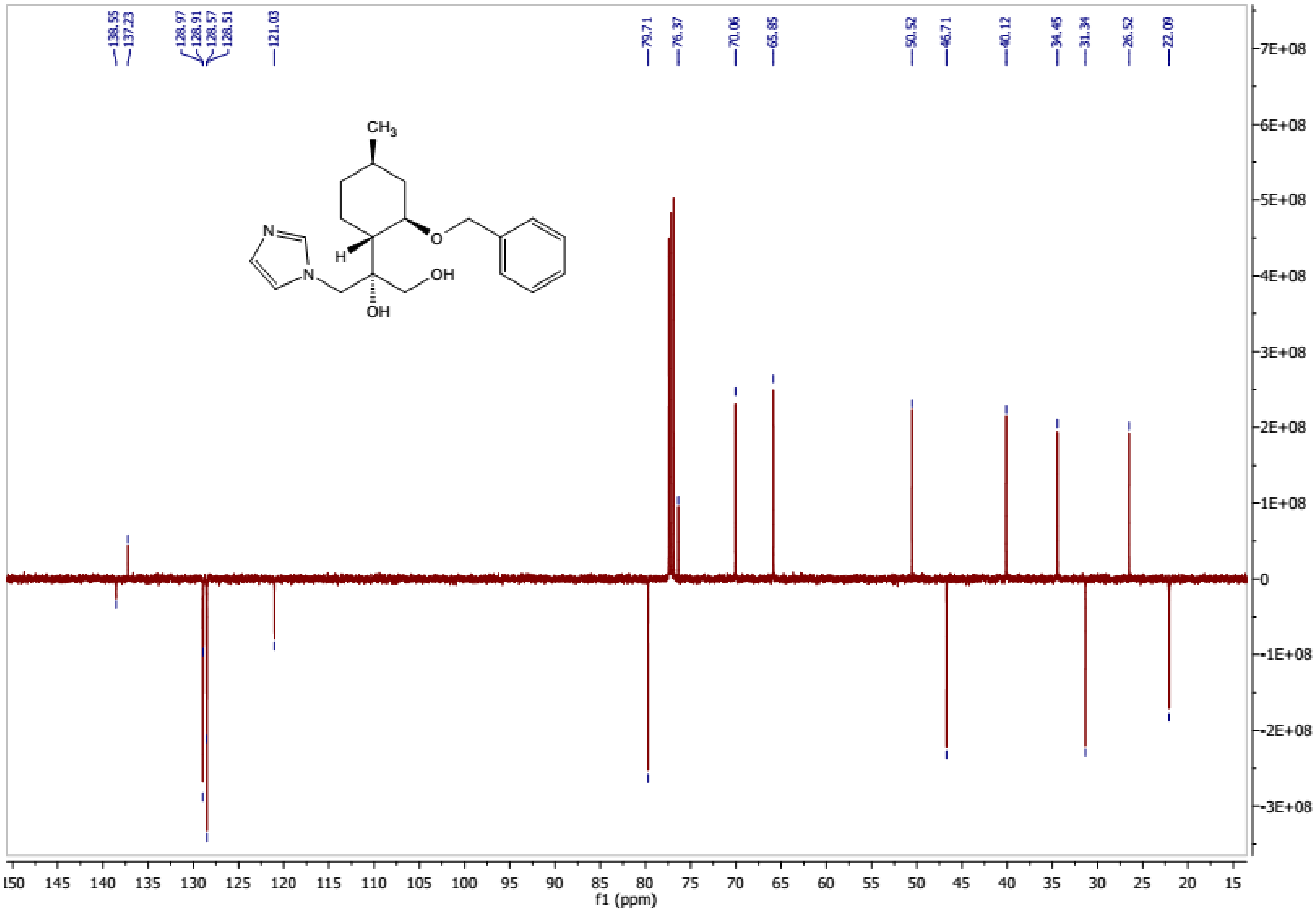

Figure S 100: ^{1}H-NMR of compound **35b**

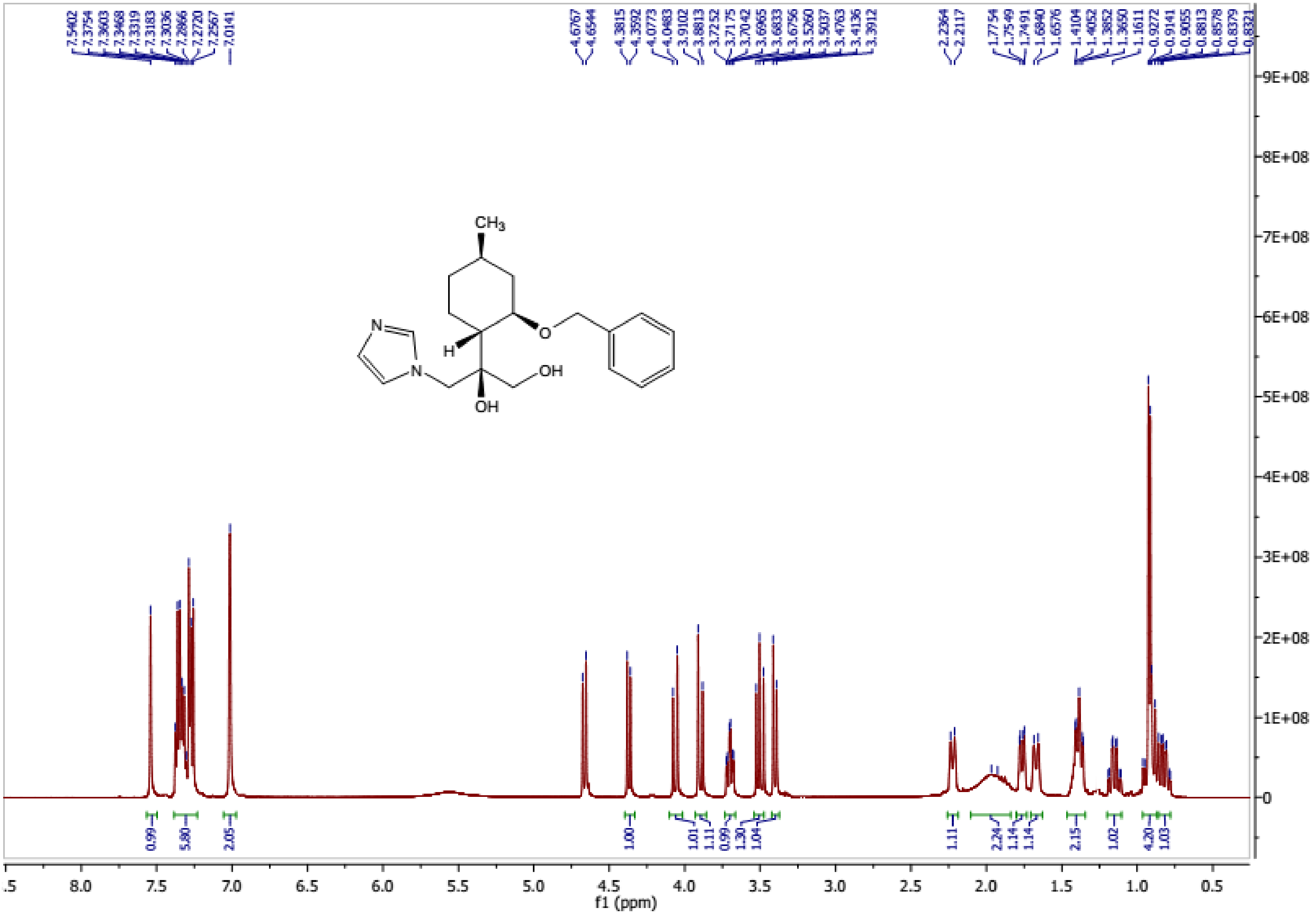

Figure S 101: ^{13}C-NMR of compound **35b**

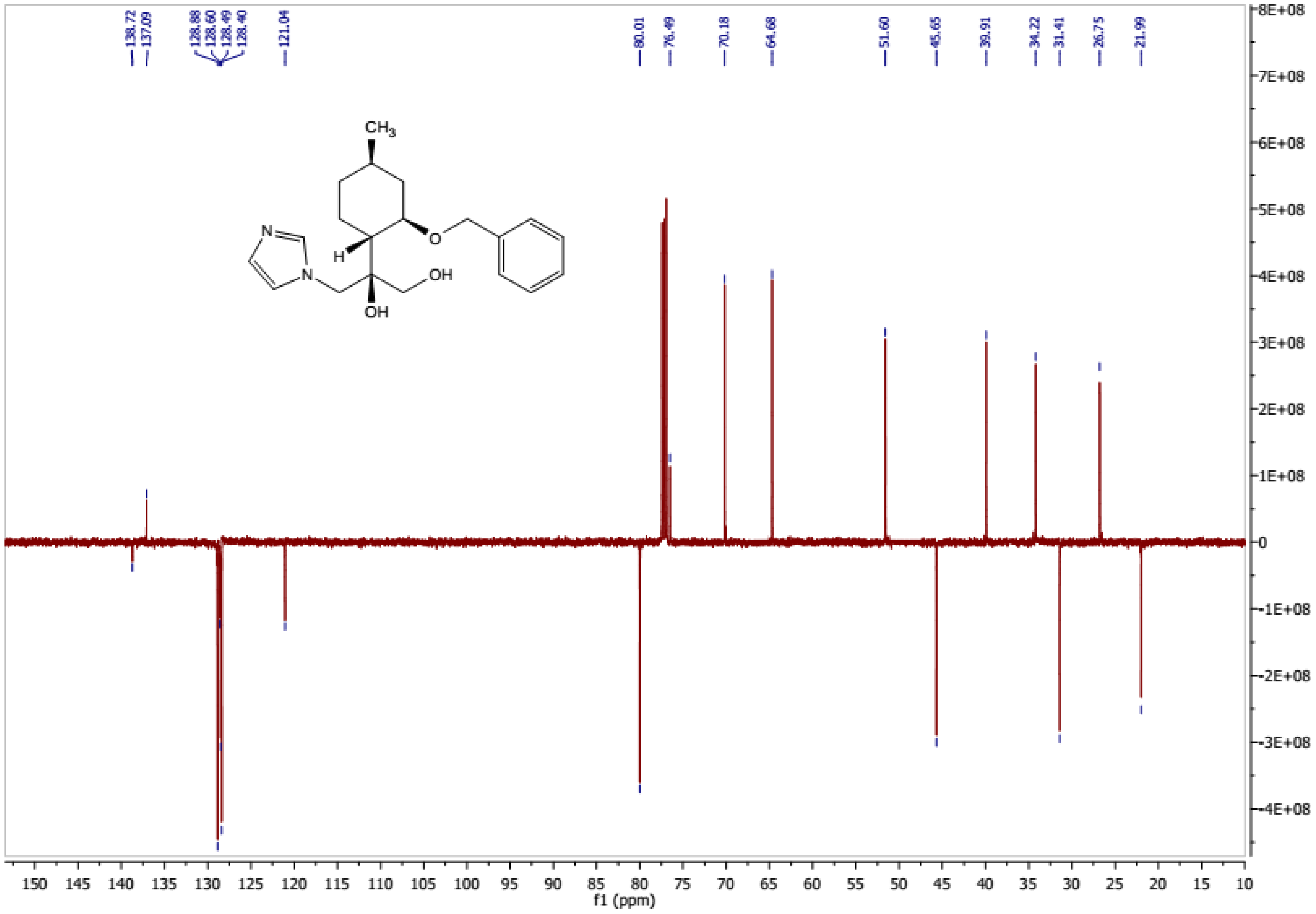

Figure S 102: ^{1}H-NMR of compound **36a**

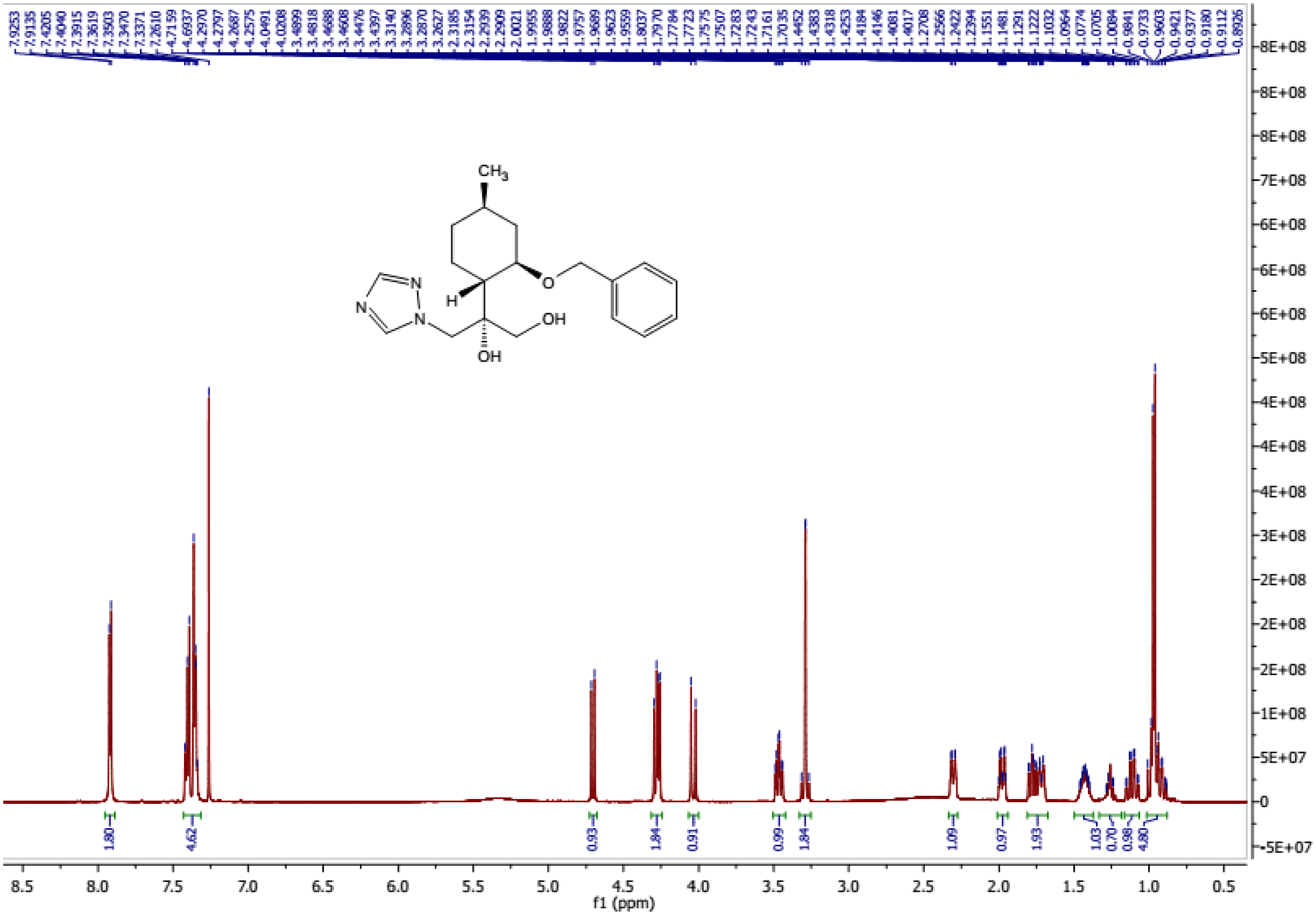

Figure S 103: ^{13}C-NMR of compound **36a**

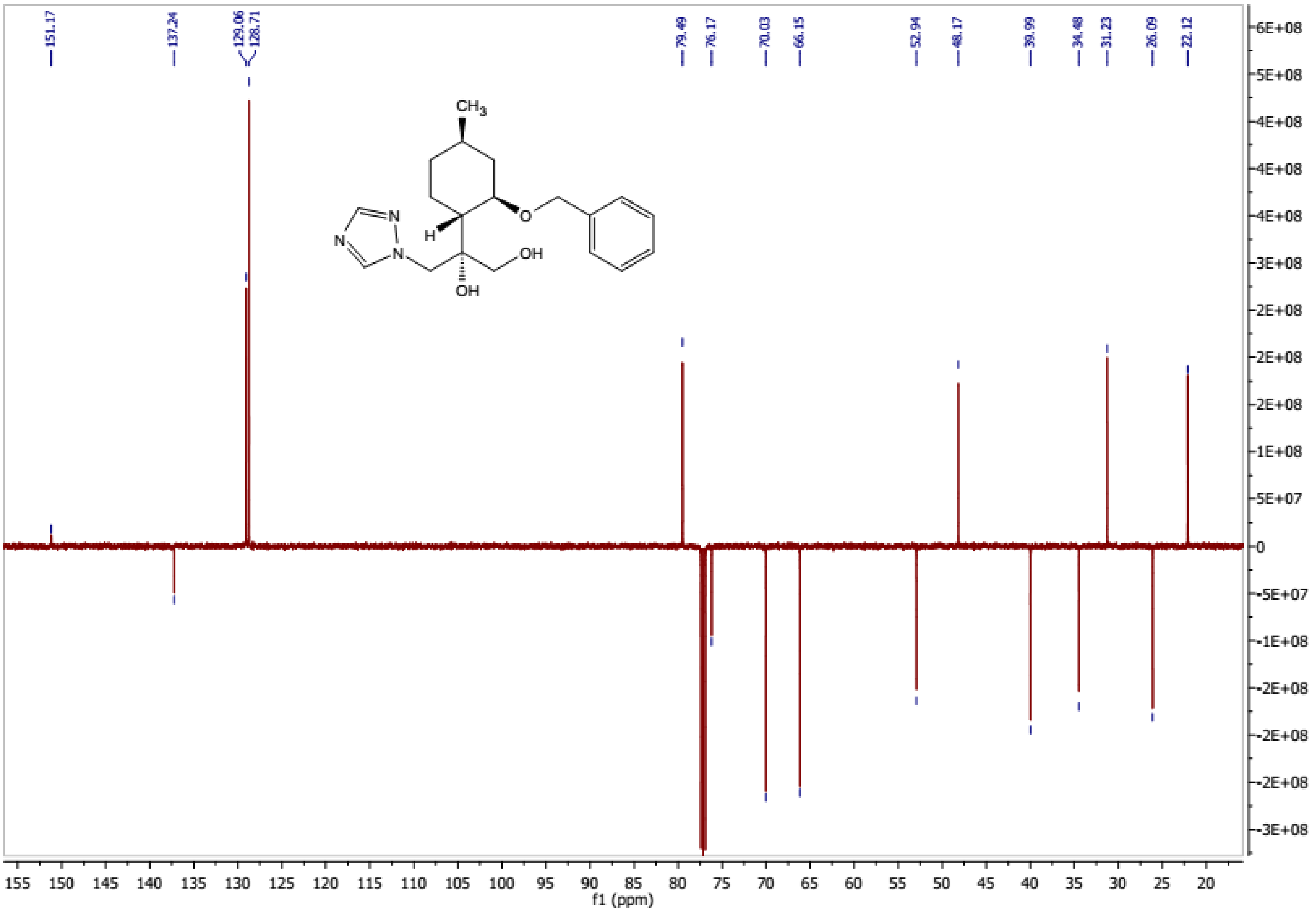

Figure S 104: ^{1}H-NMR of compound **36b**

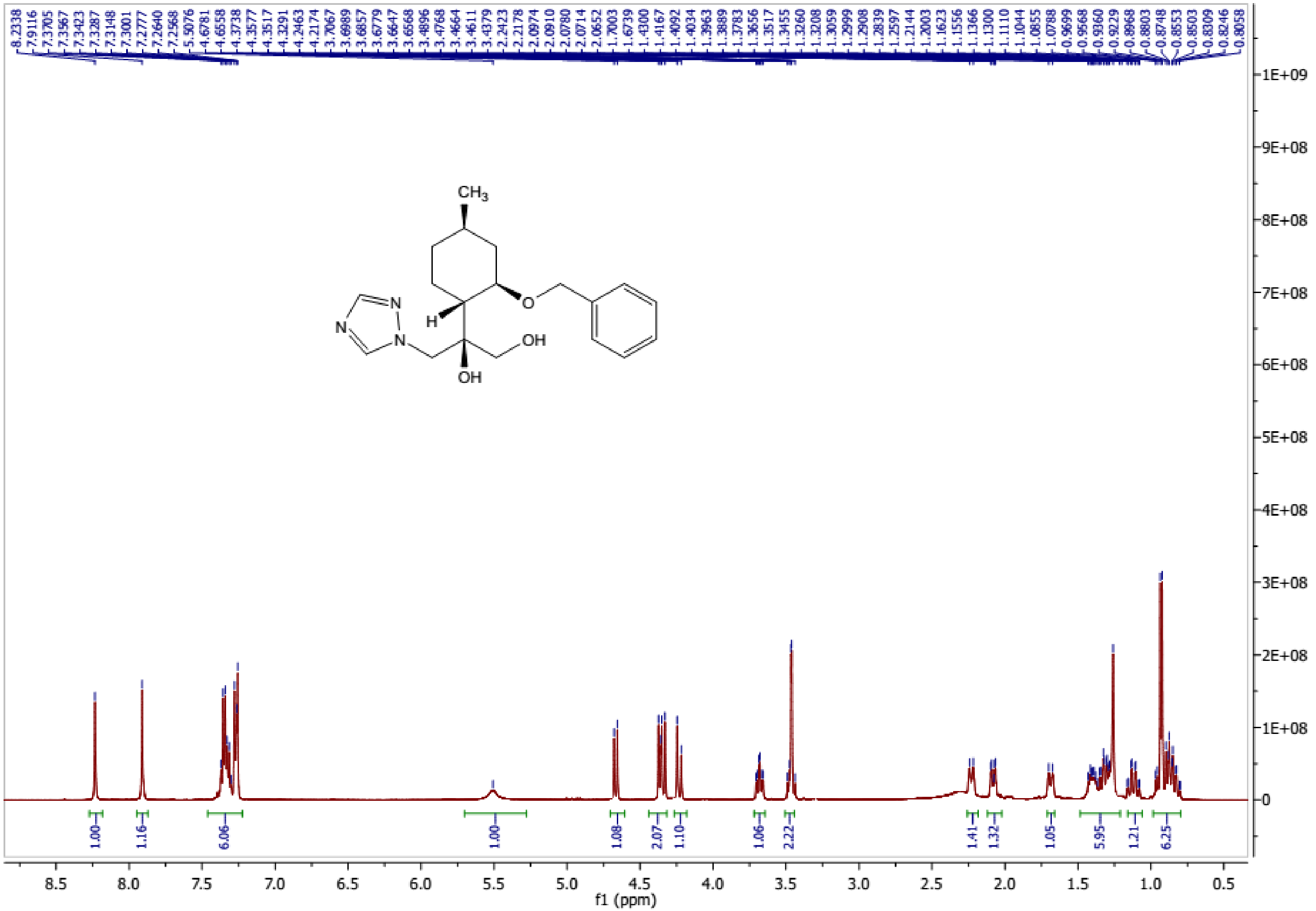

Figure S 105: ^{13}C-NMR of compound **36b**

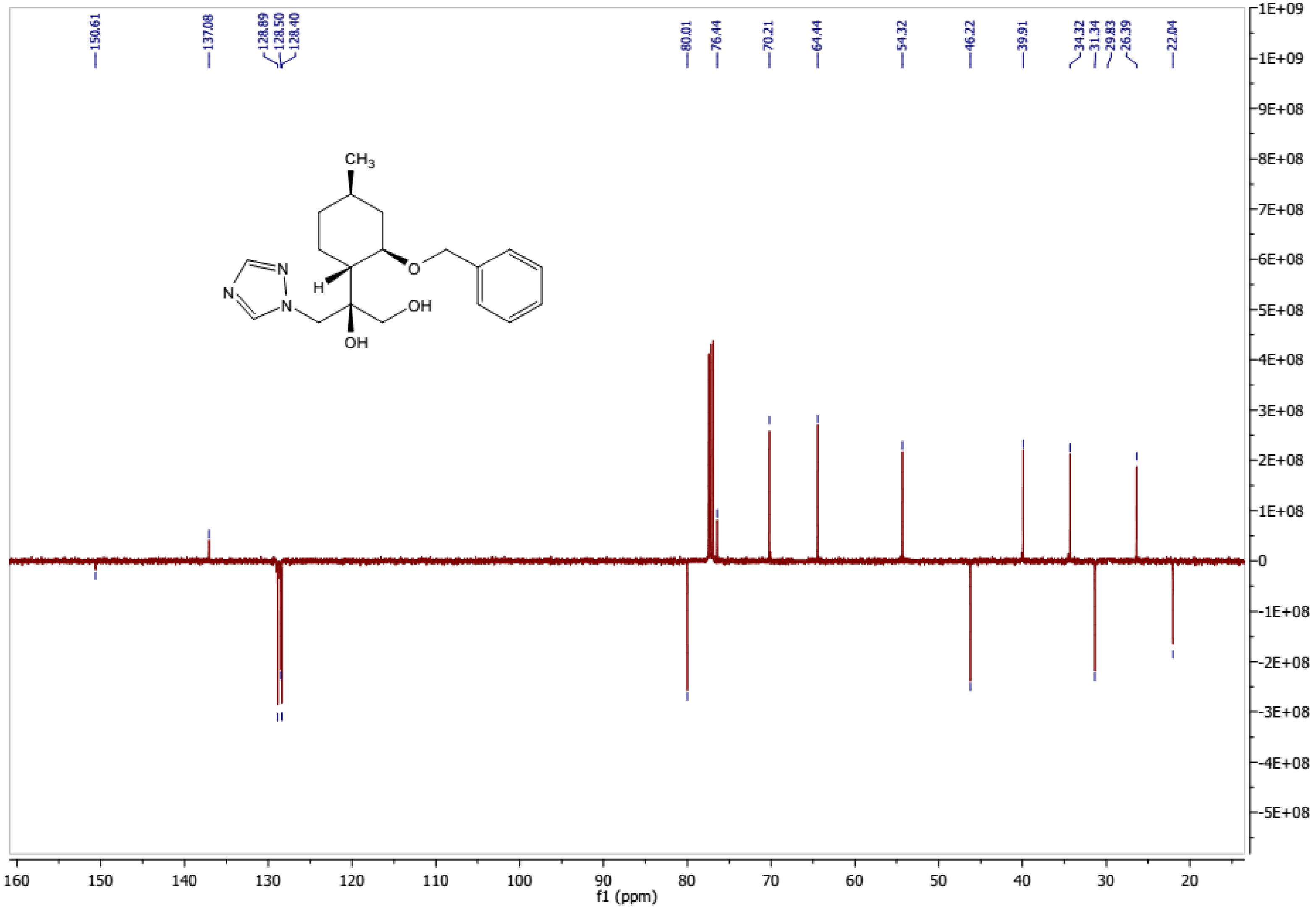

Figure S 106: ¹H-NMR of compound **38a**

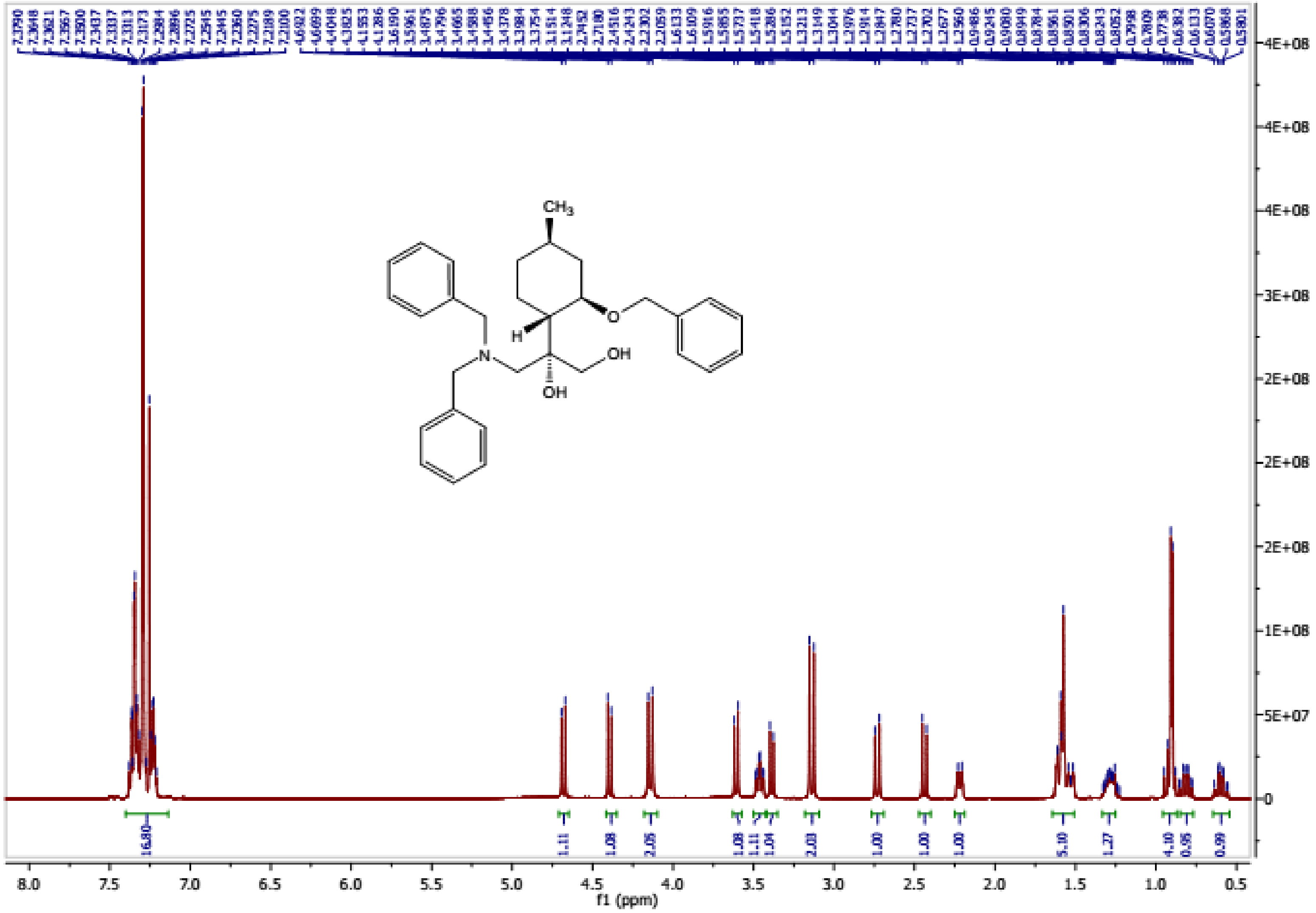

Figure S 107: ^{13}C-NMR of compound **38b**

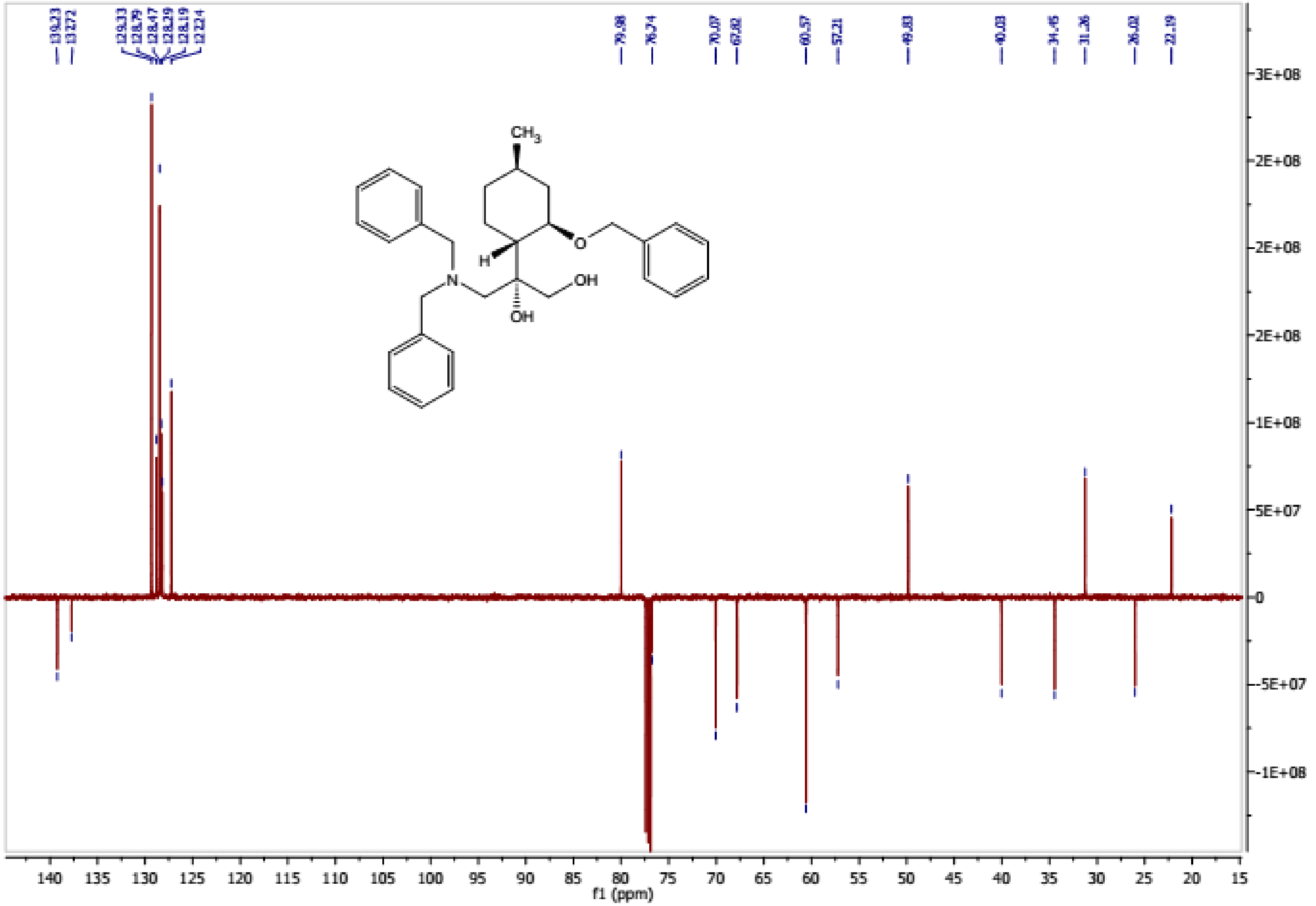

Figure S 108: ^{1}H-NMR of compound **38b**

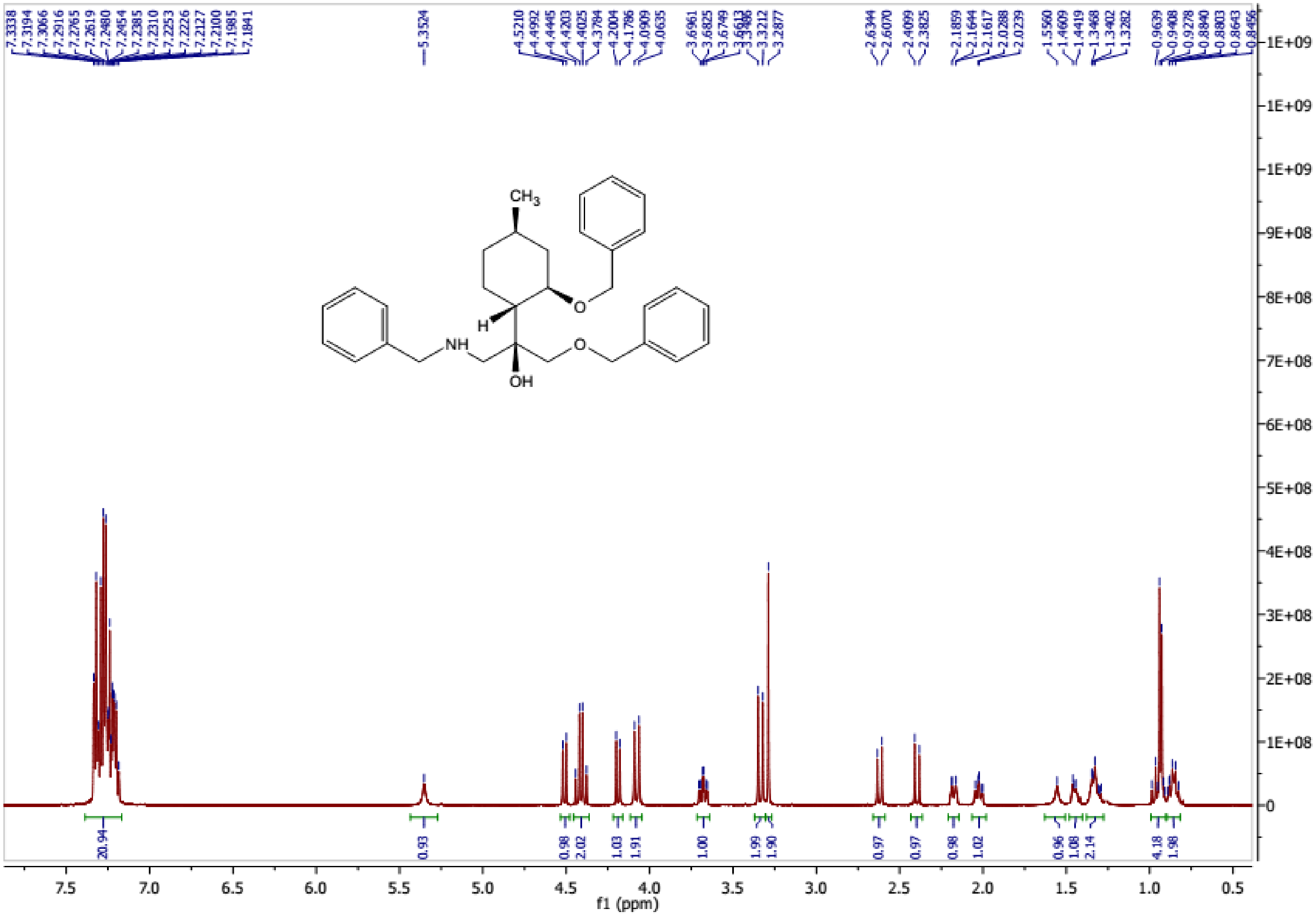

Figure S 109: ¹³C-NMR of compound **38b**

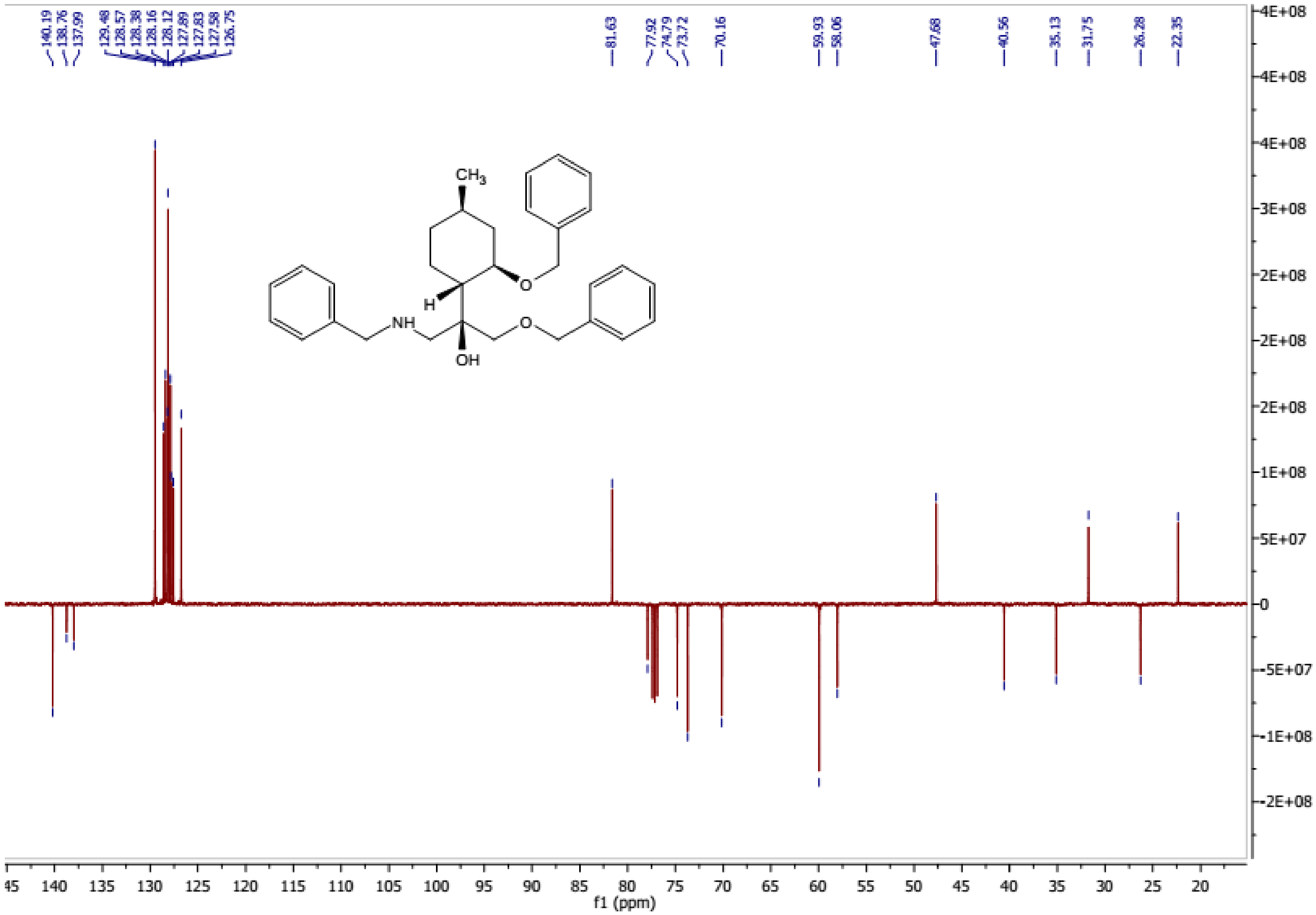

Figure S 110: ^{1}H-NMR of compound **39a**

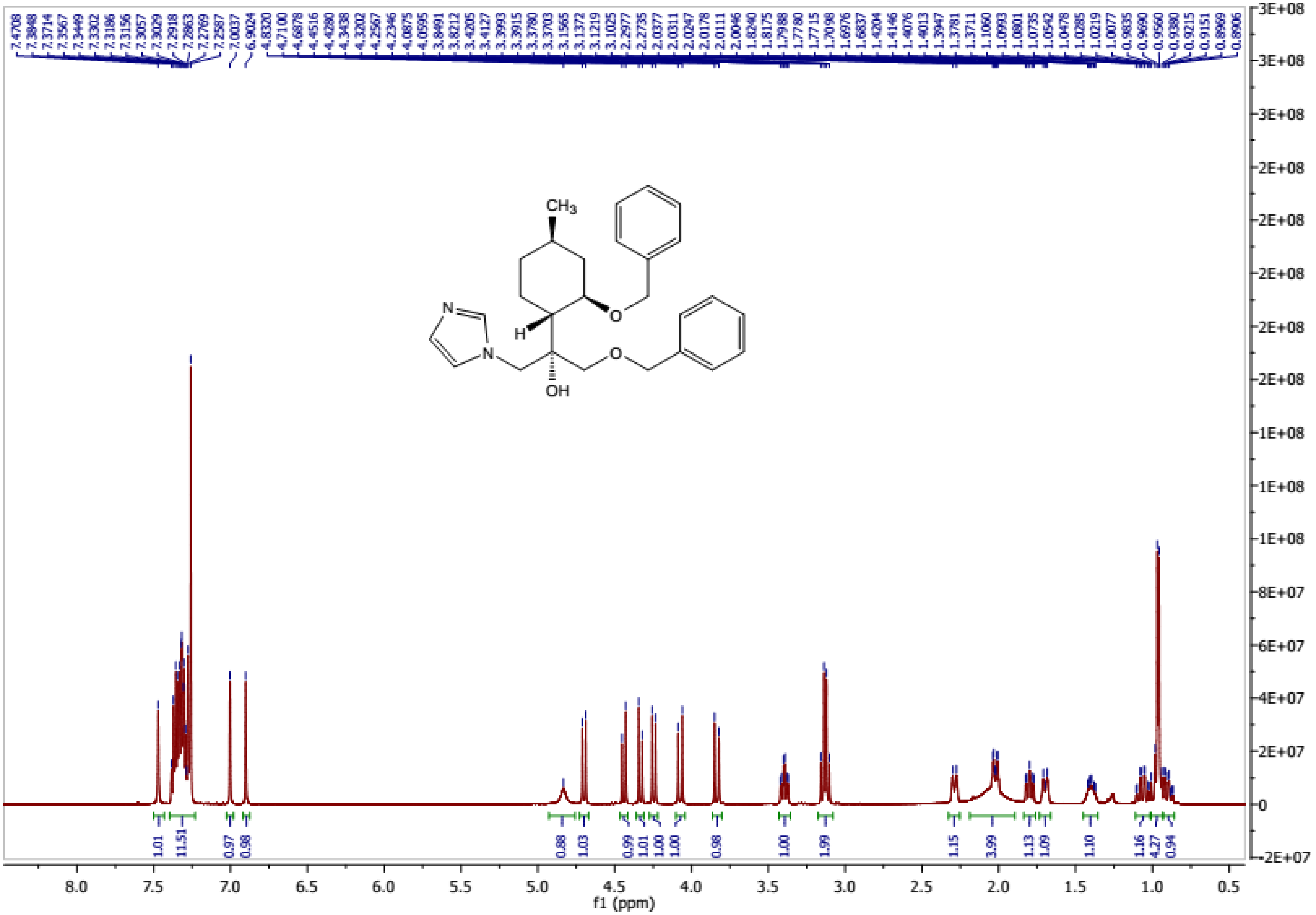

^{13}C-NMR of compound **39a**

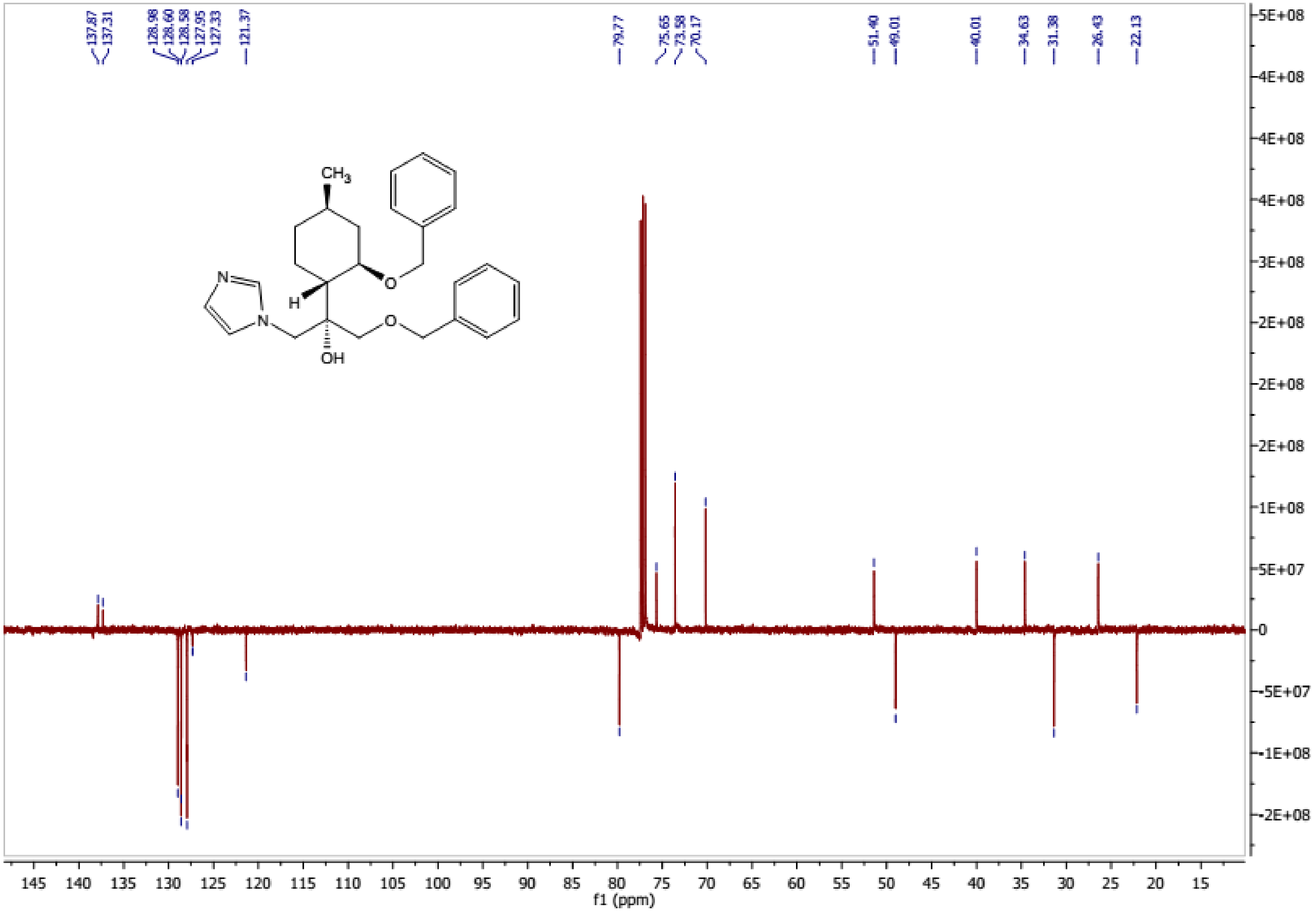

Figure S 111: ^{1}H-NMR of compound **39b**

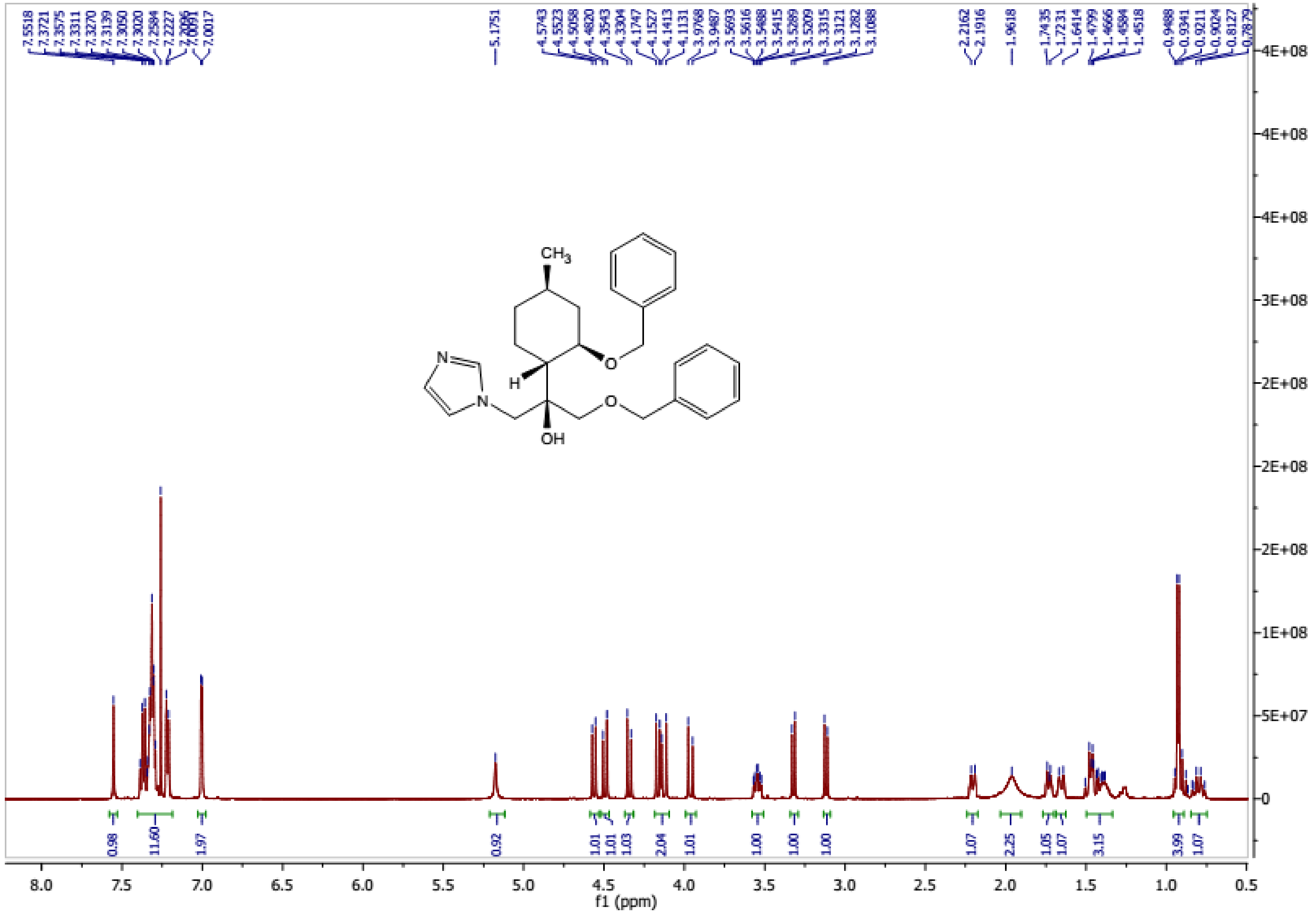

Figure S 112: ^{13}C-NMR of compound **39b**

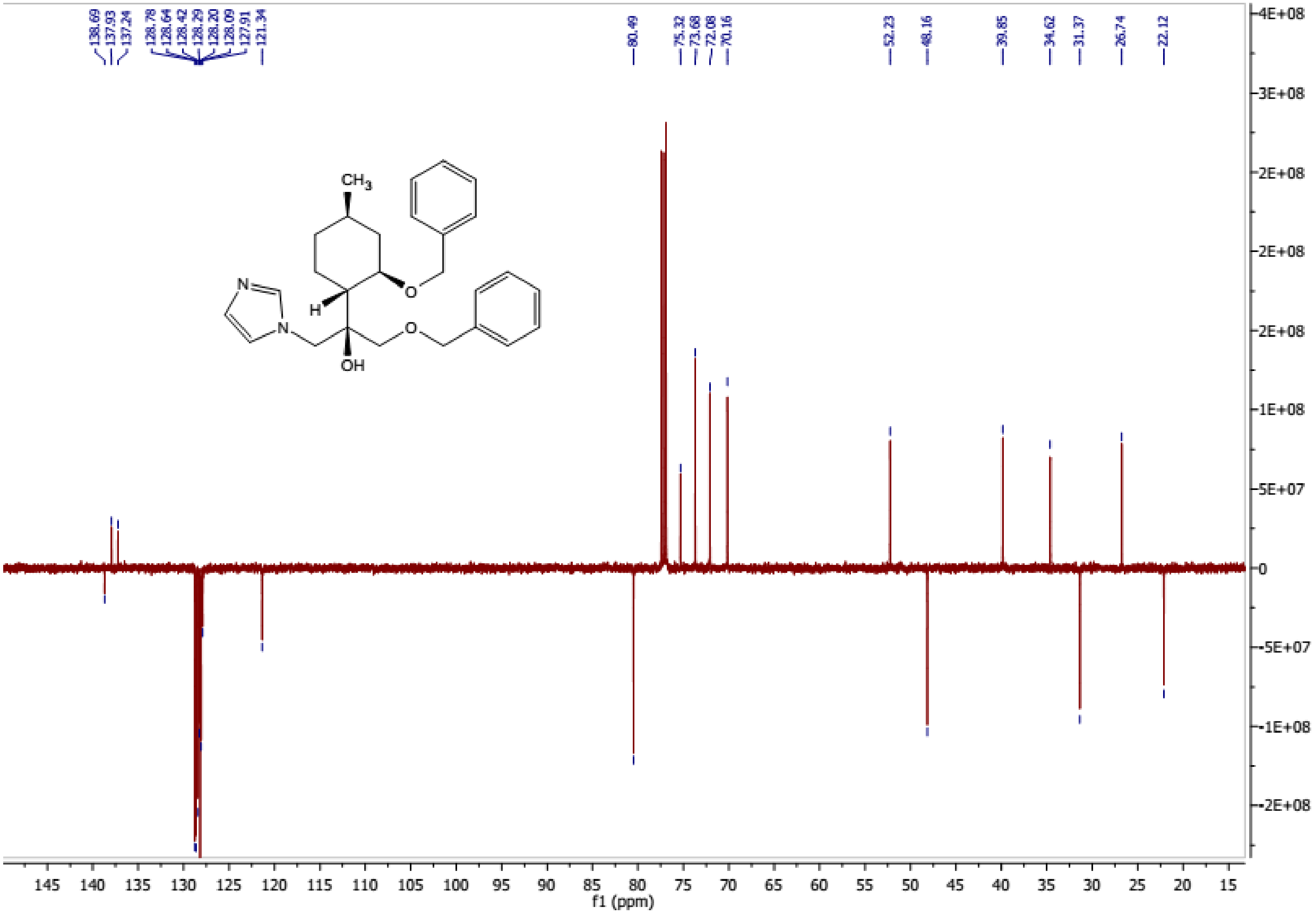

Figure S 113: ^{1}H-NMR of compound **40a**

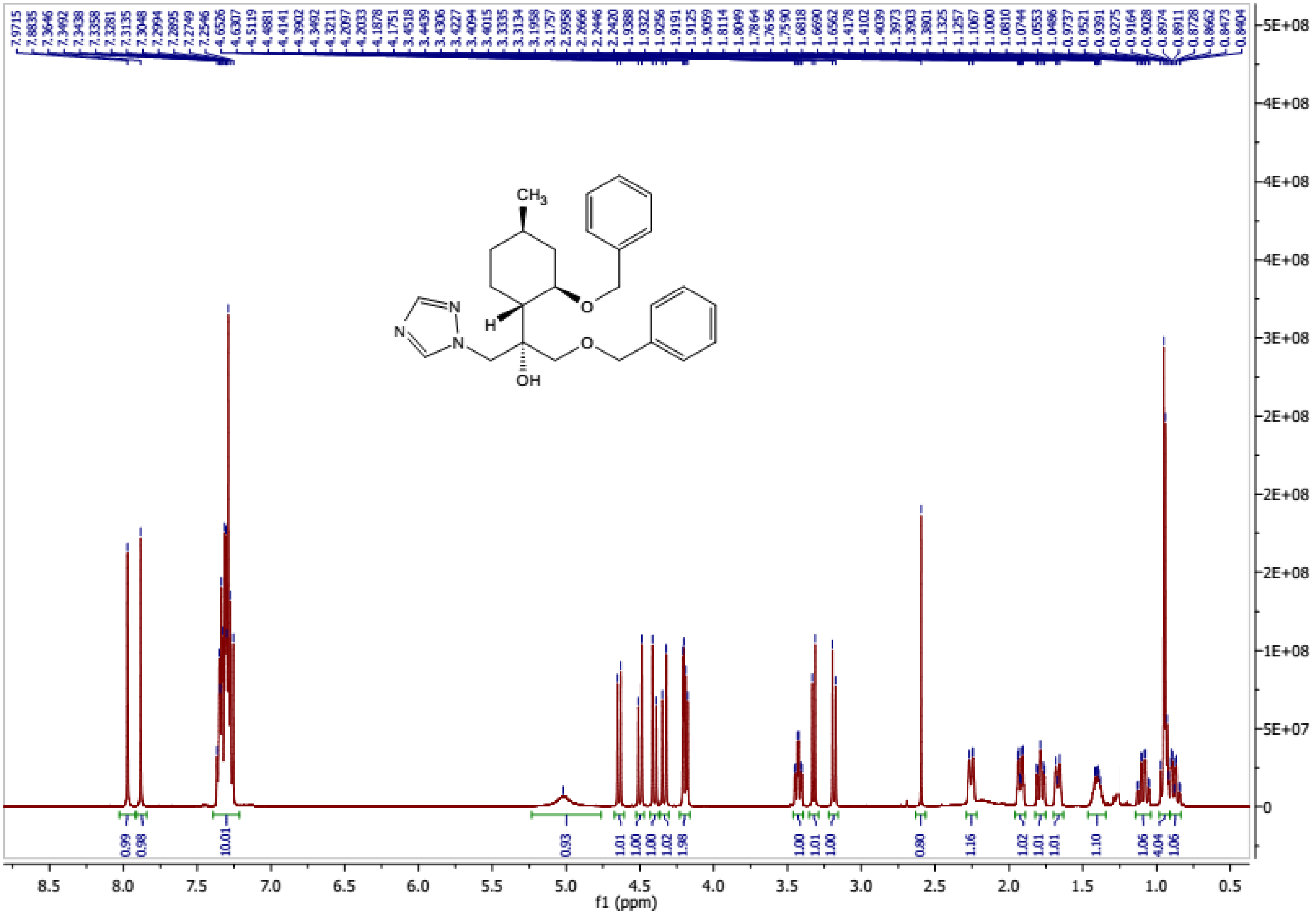

Figure S 114: ^{13}C-NMR of compound **40a**

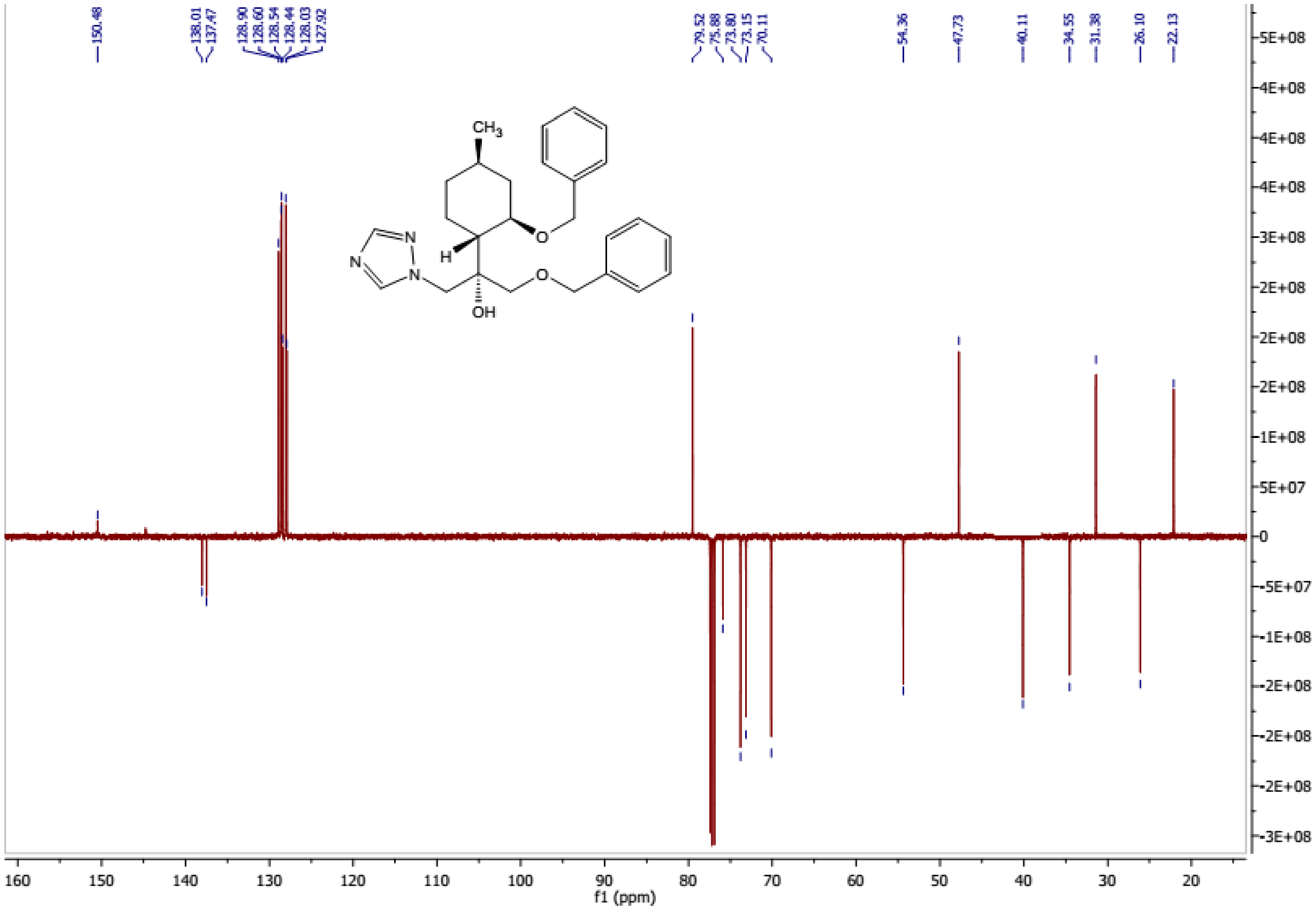

Figure S 115: ^{1}H-NMR of compound **40b**

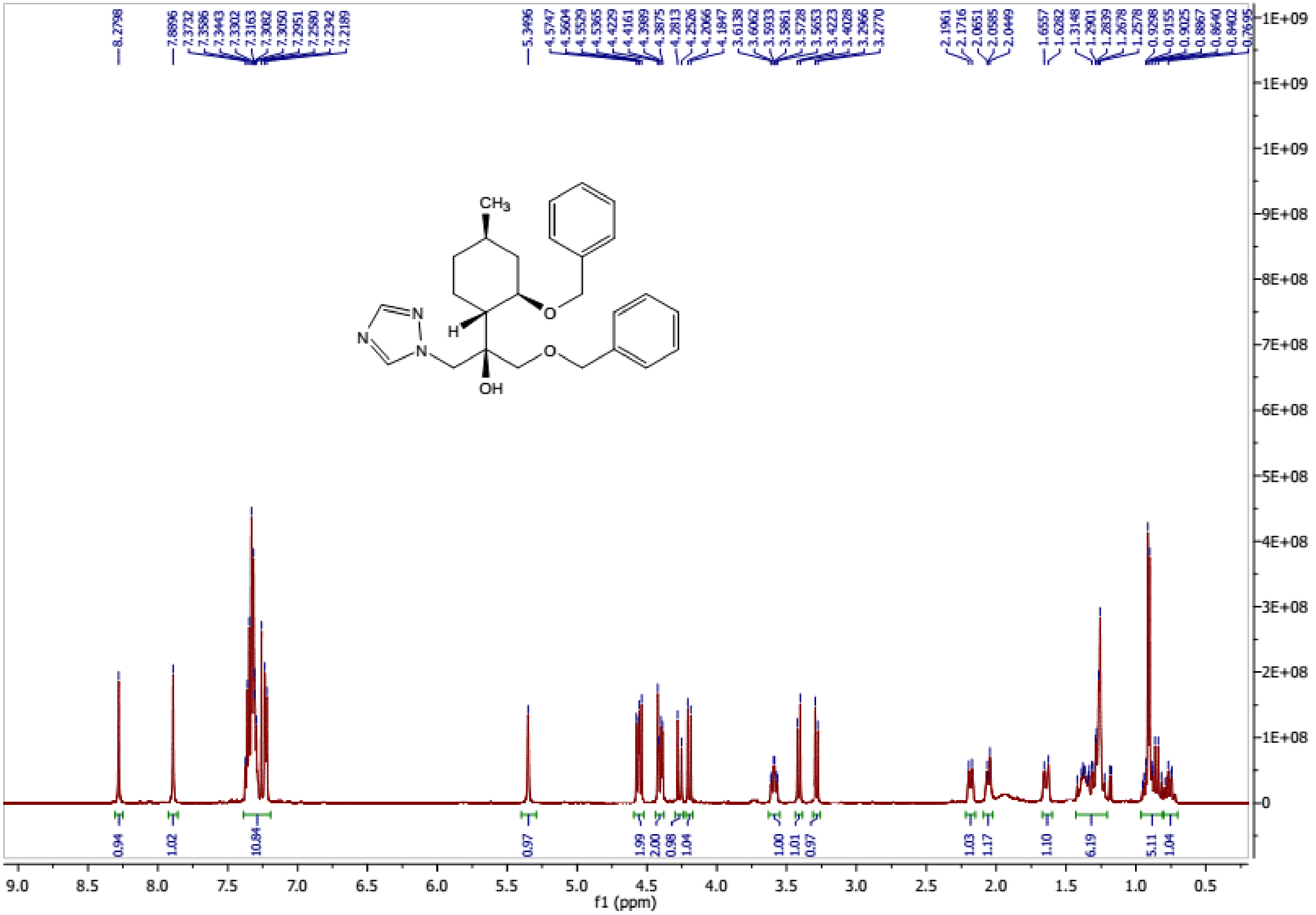

Figure S 116: ^{13}C-NMR of compound **40b**

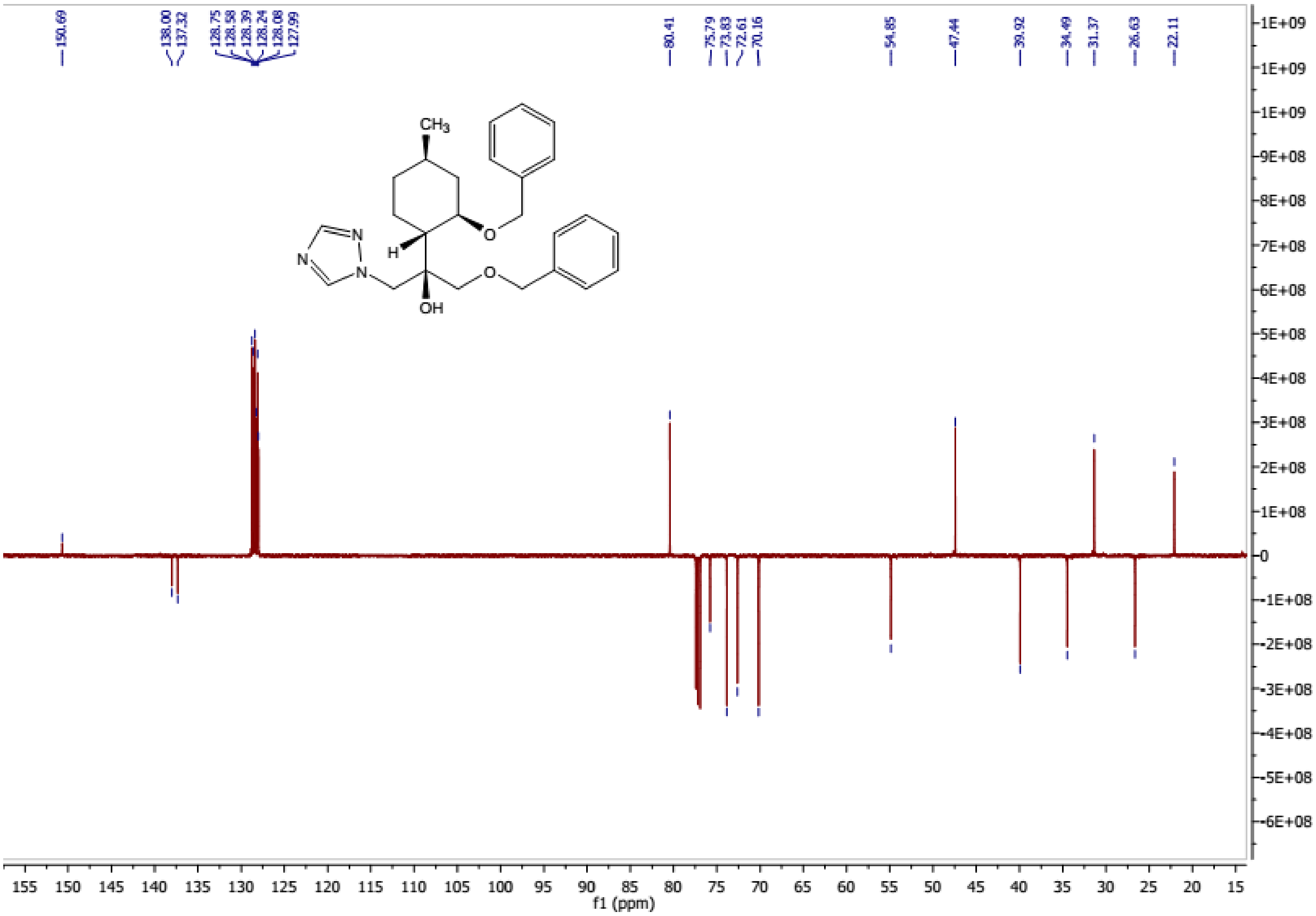

Figure S 117: ¹H-NMR of compound **41a**

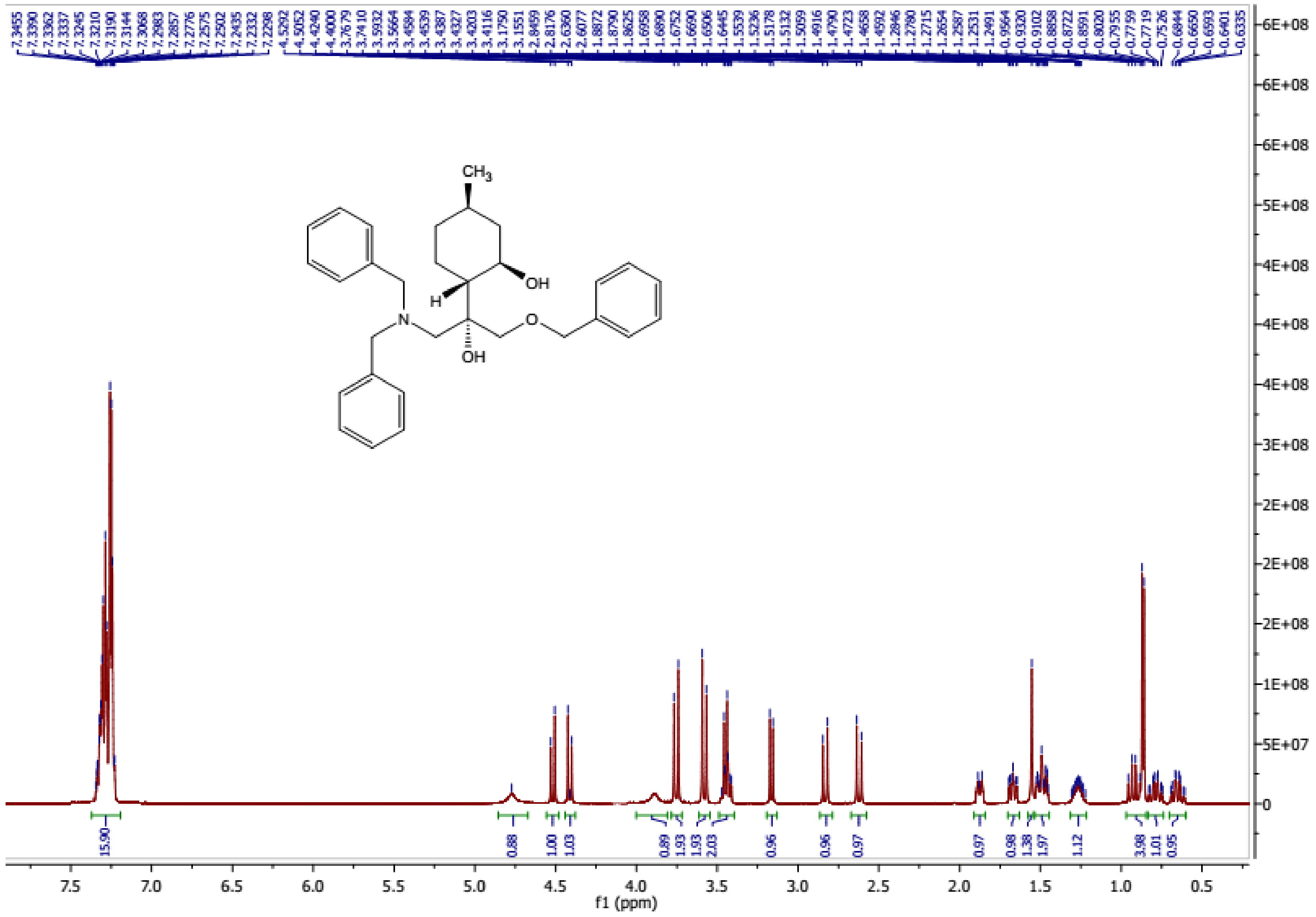

Figure S 118: ^{13}C-NMR of compound **41a**

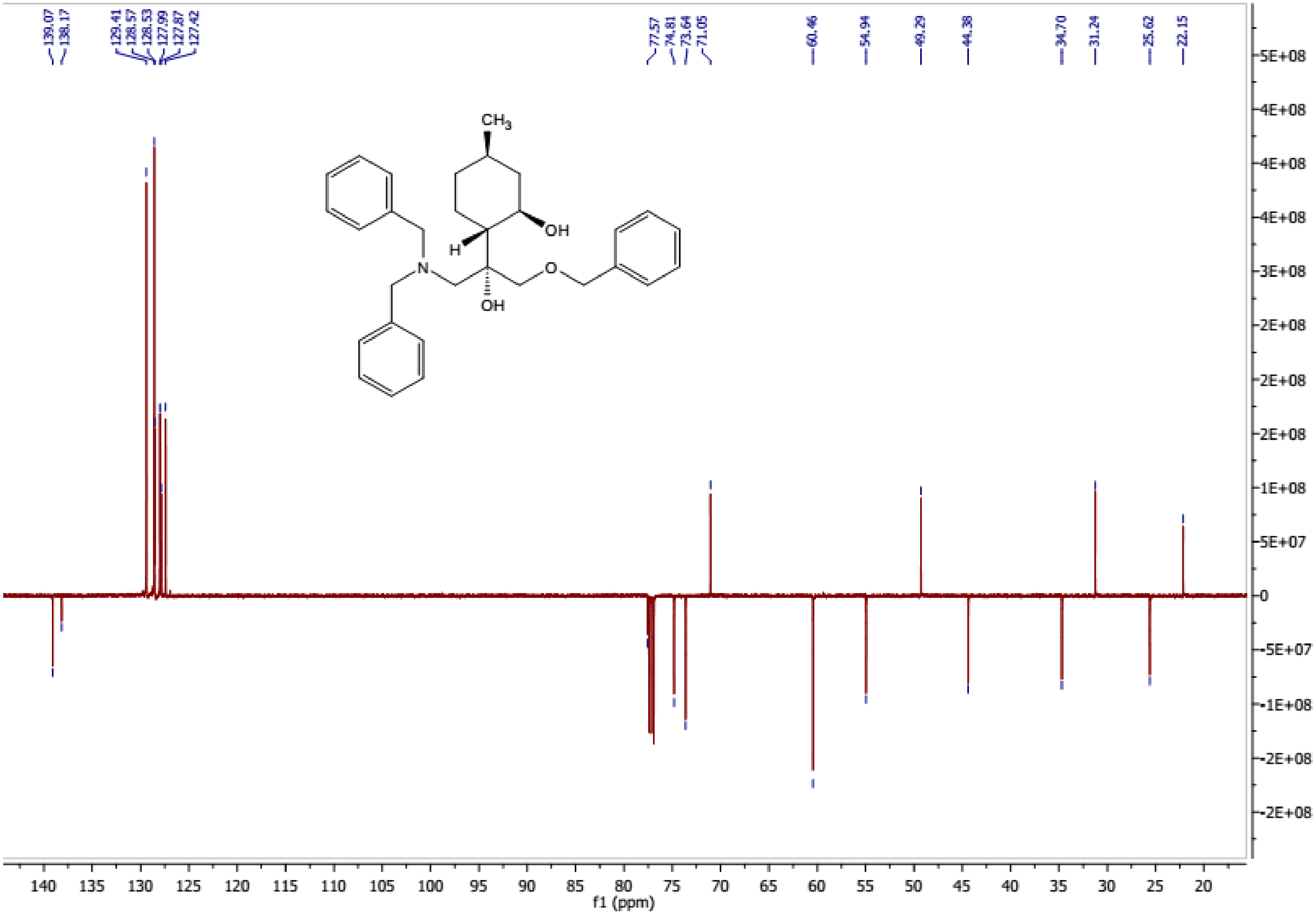

Figure S 119: ^{1}H-NMR of compound **41b**

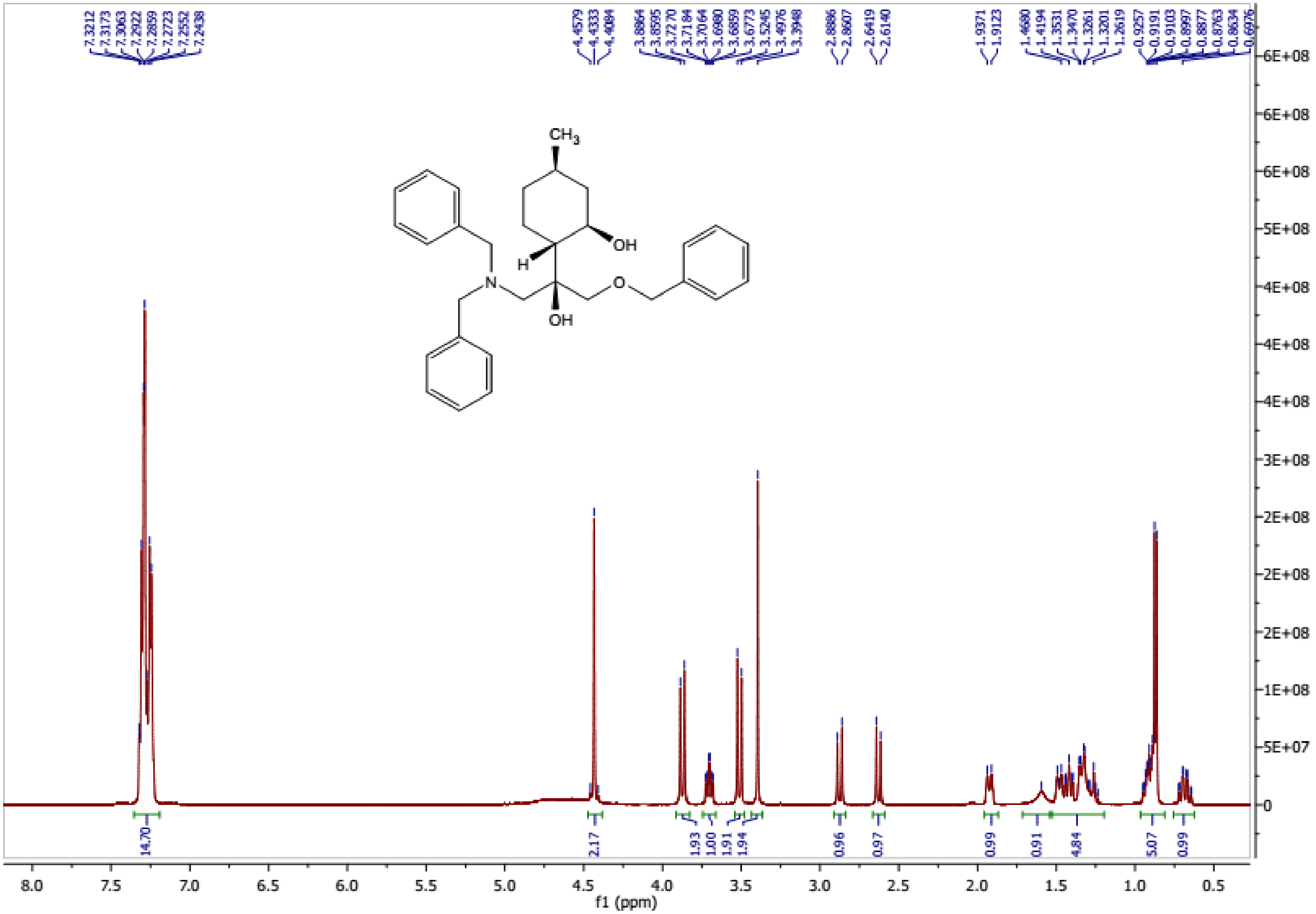

Figure S 120: ^{13}C-NMR of compound **41b**

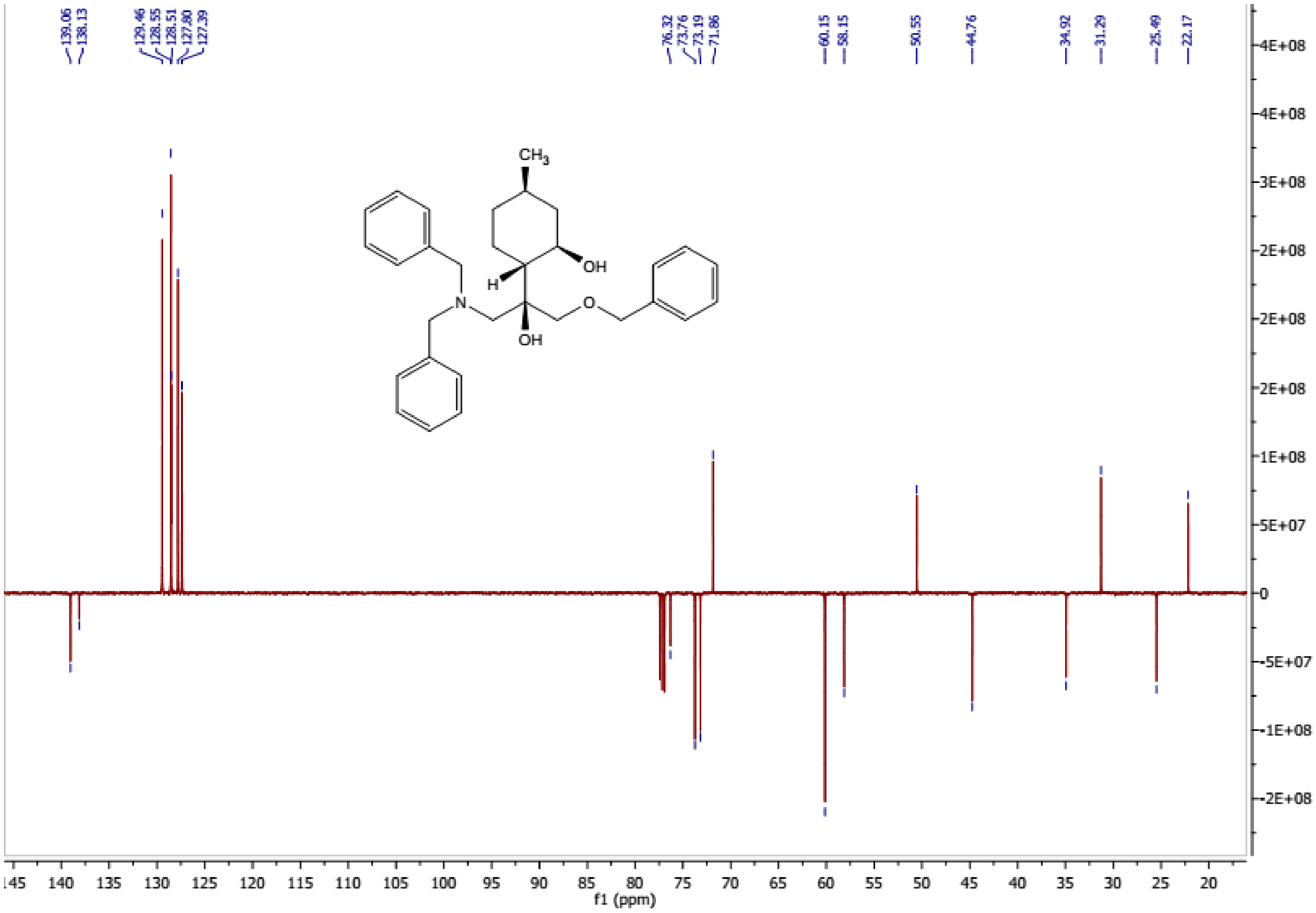

Figure S 121: ^{1}H-NMR of compound **42a**

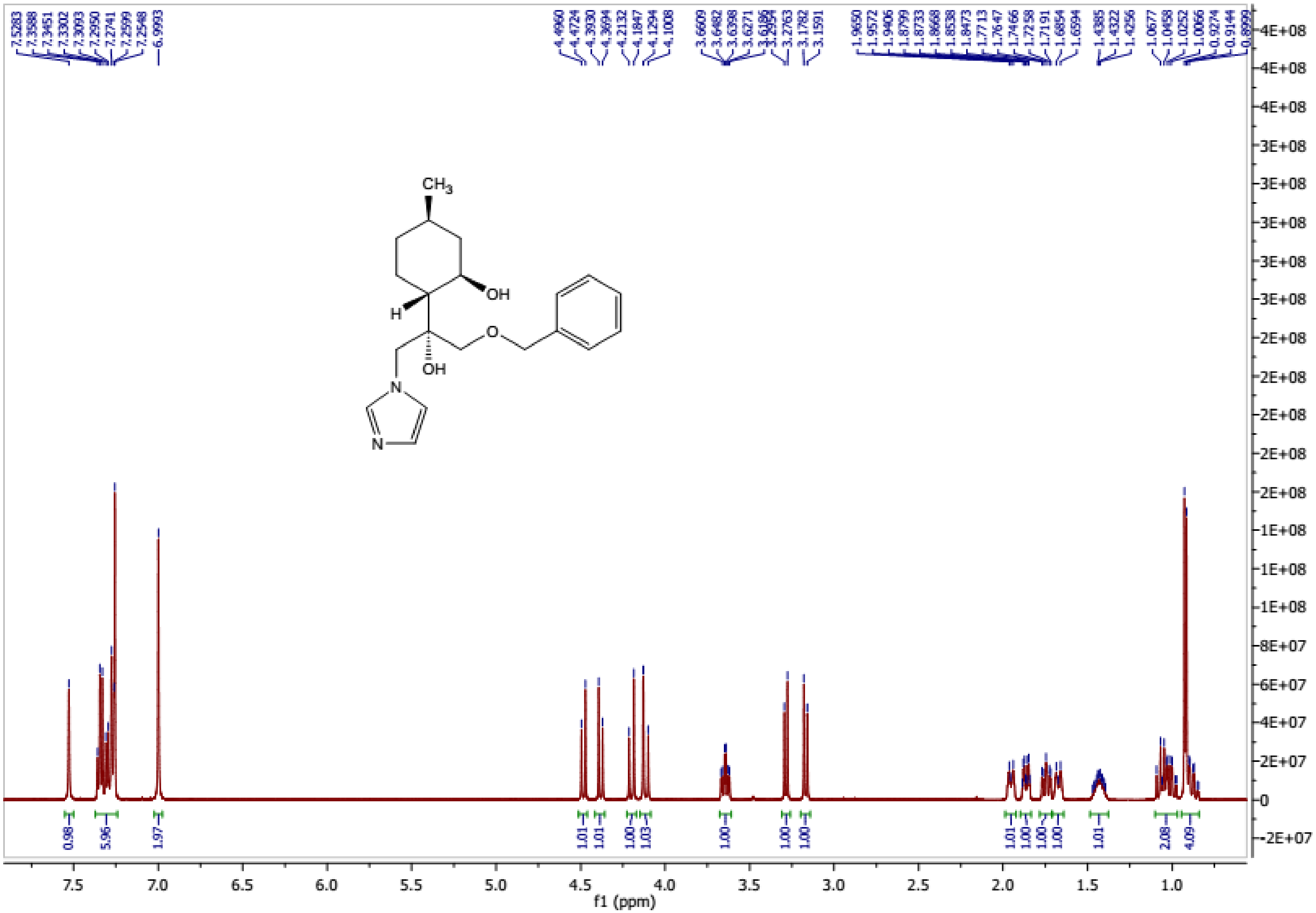

Figure S 122: 13C-NMR of compound **42a**

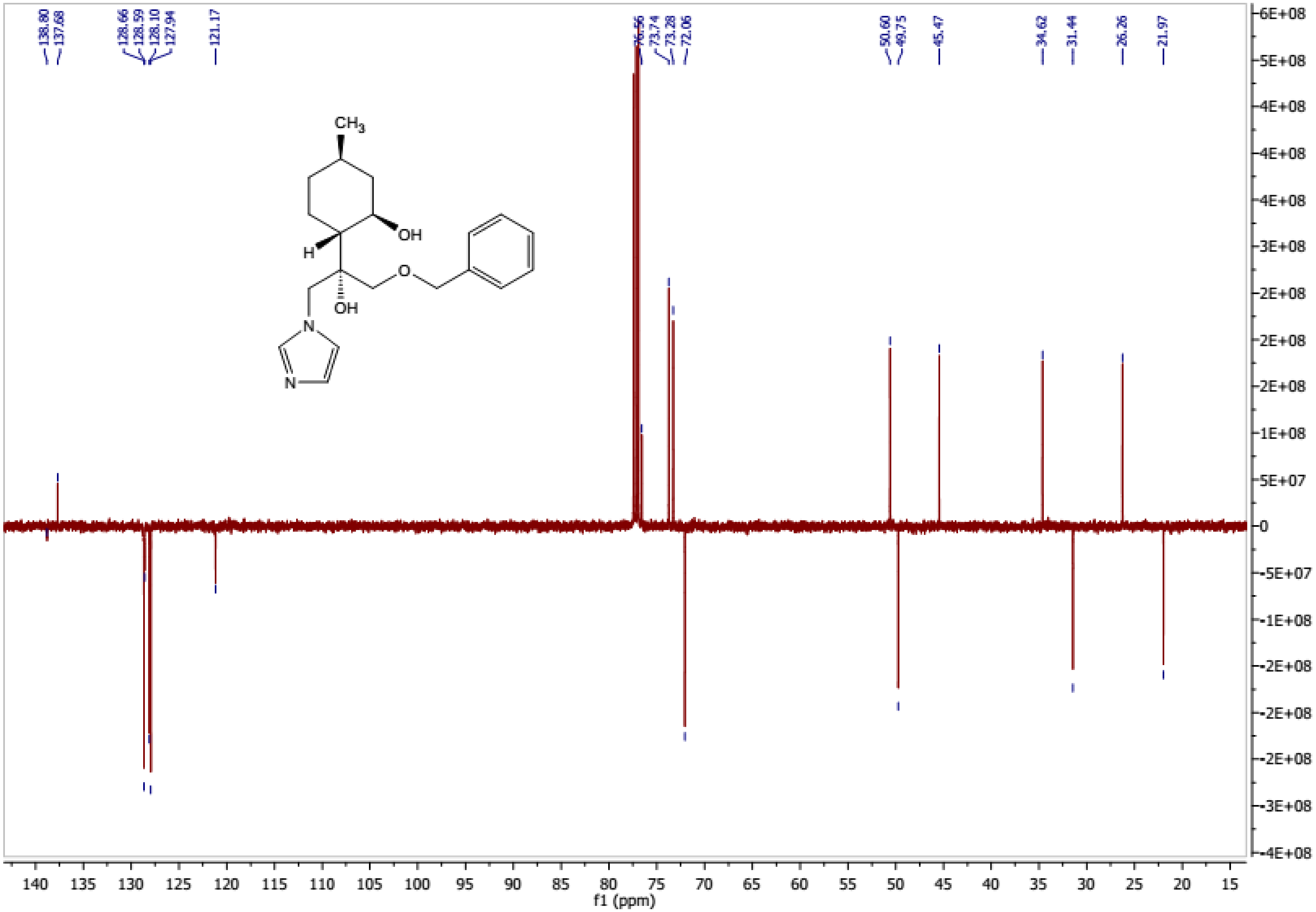

Figure S 123: ^{1}H-NMR of compound **42b**

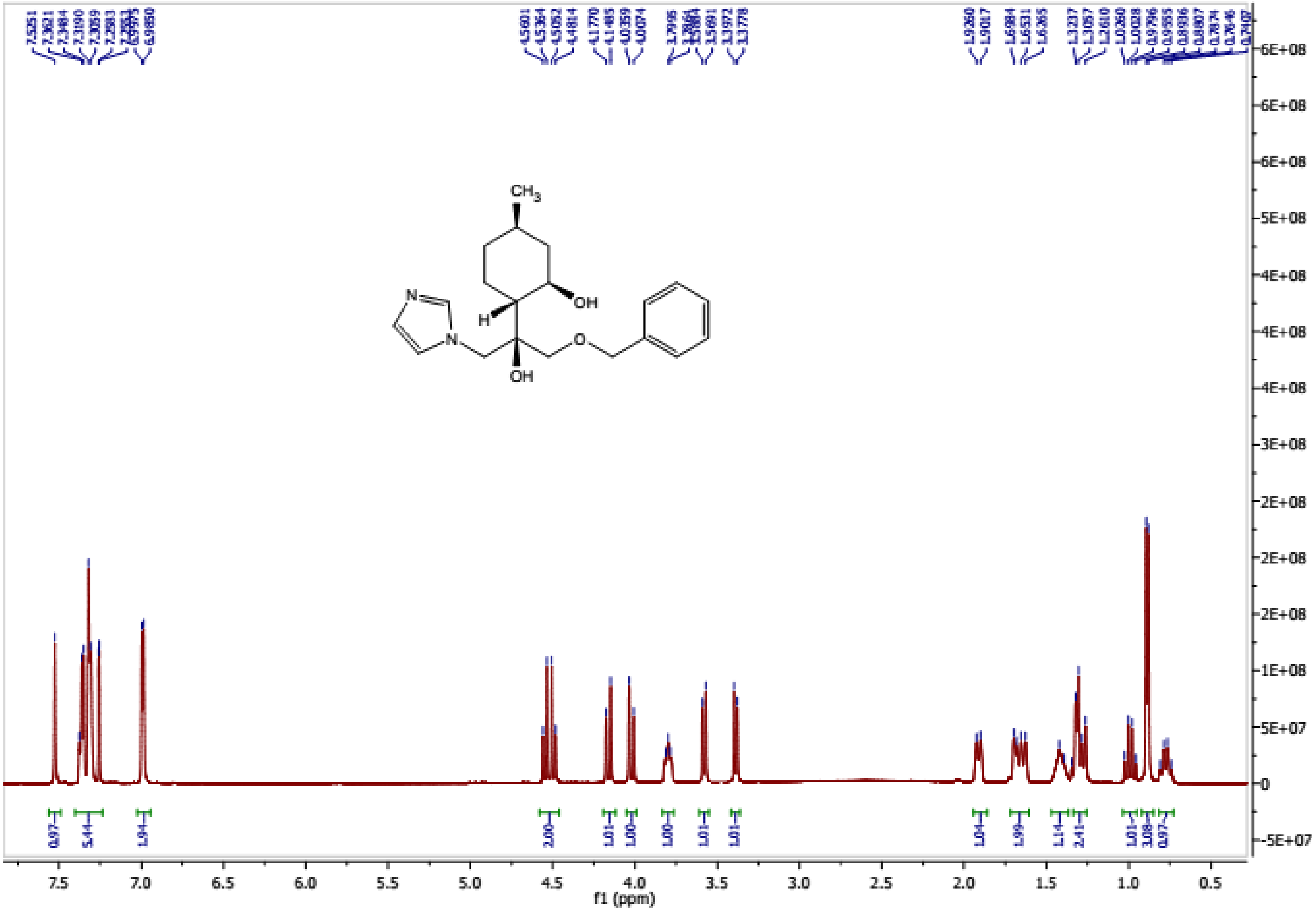

Figure S 124: ^{13}C-NMR of compound **42b**

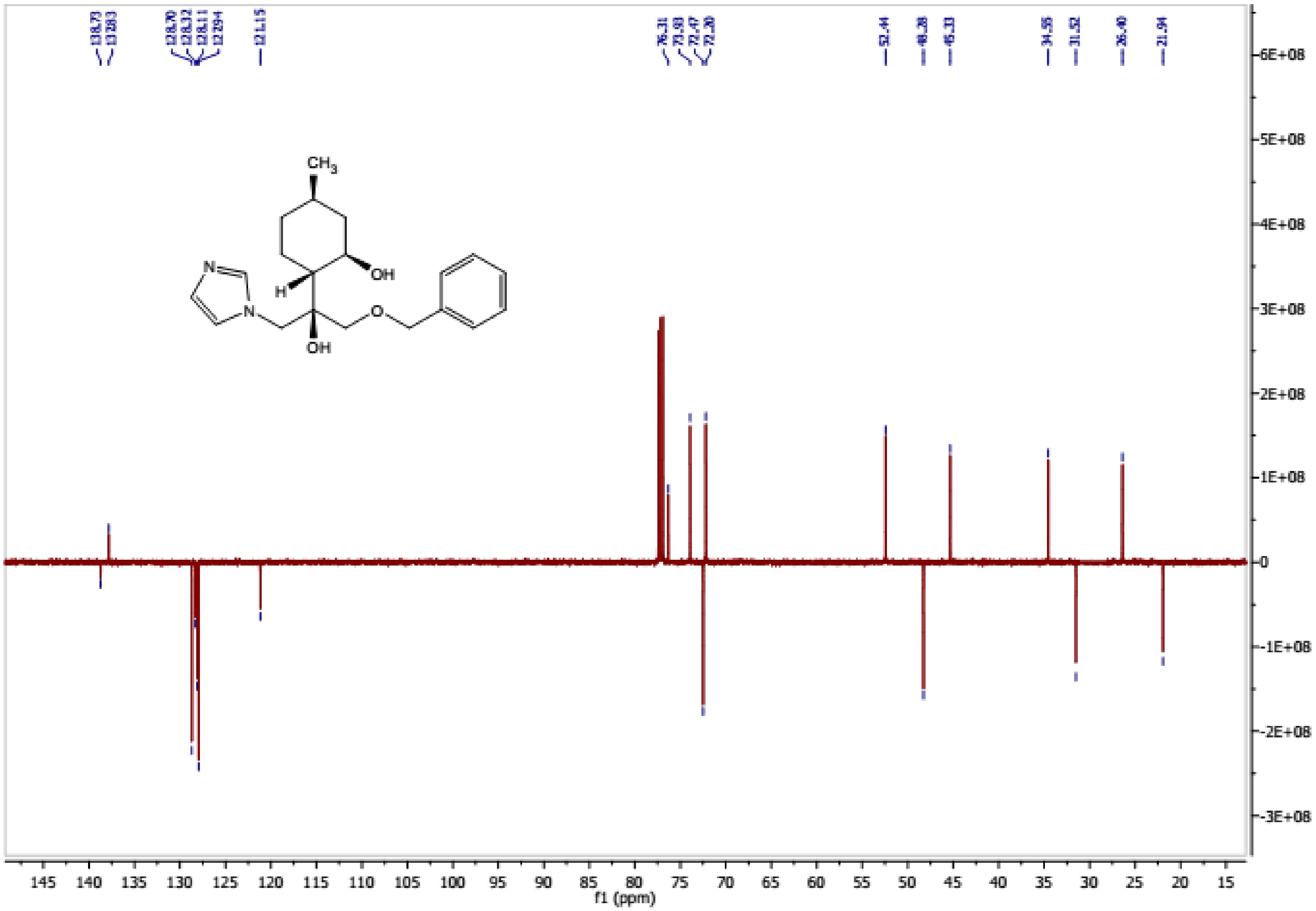

Figure S 125: ^{1}H-NMR of compound **43a**

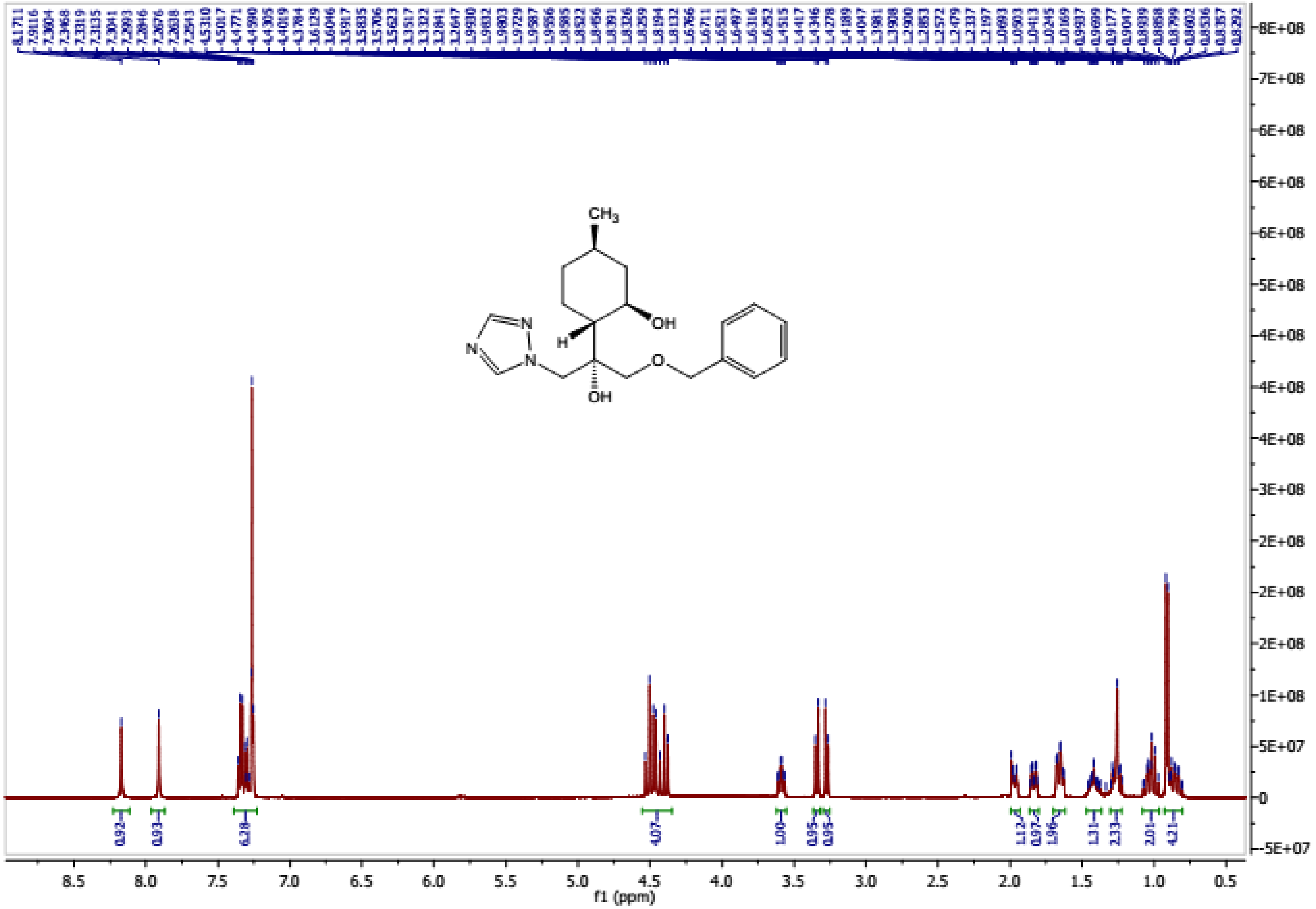

Figure S 126: ^{13}C-NMR of compound **43a**

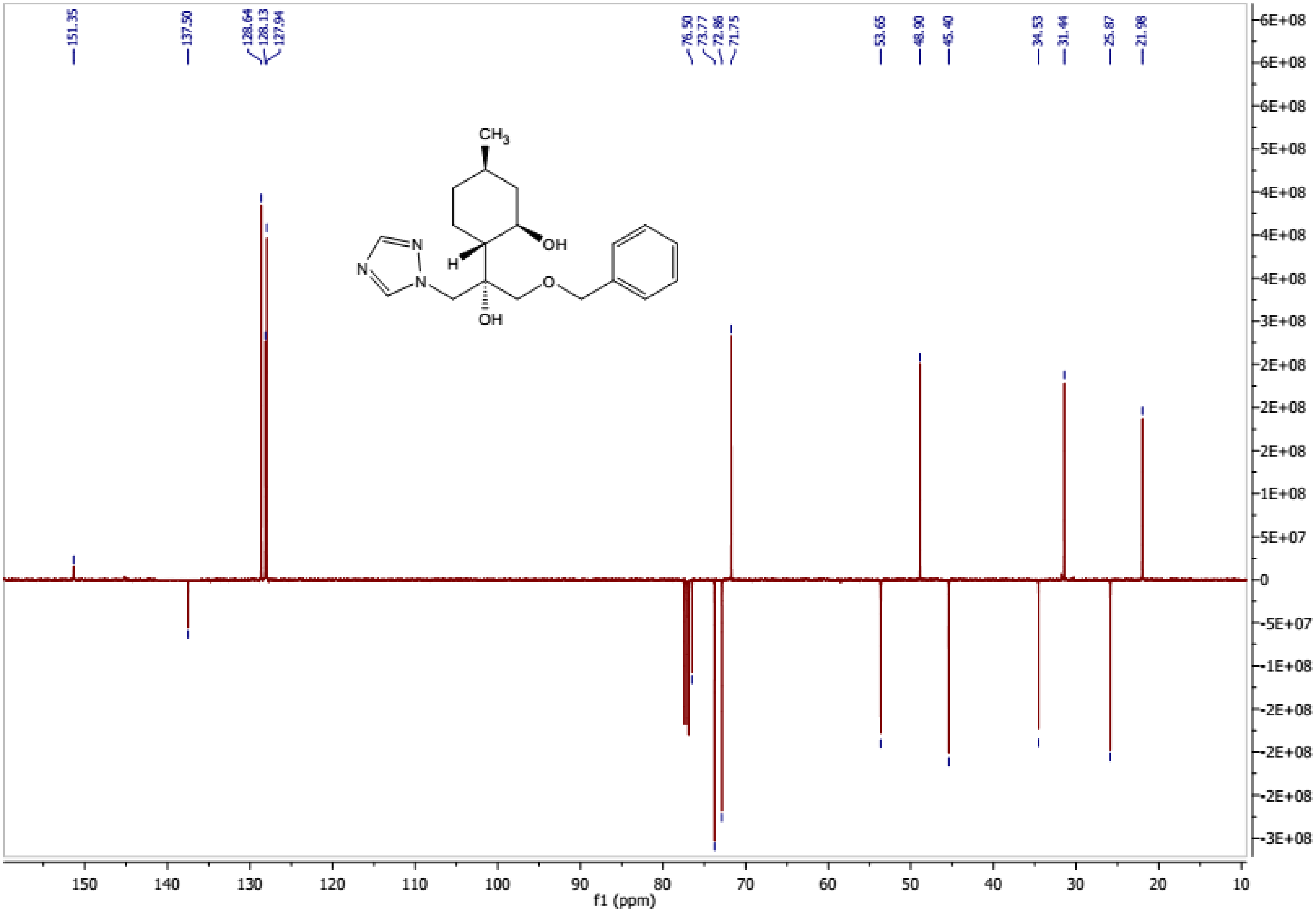

Figure S 127: ^{1}H-NMR of compound **43b**

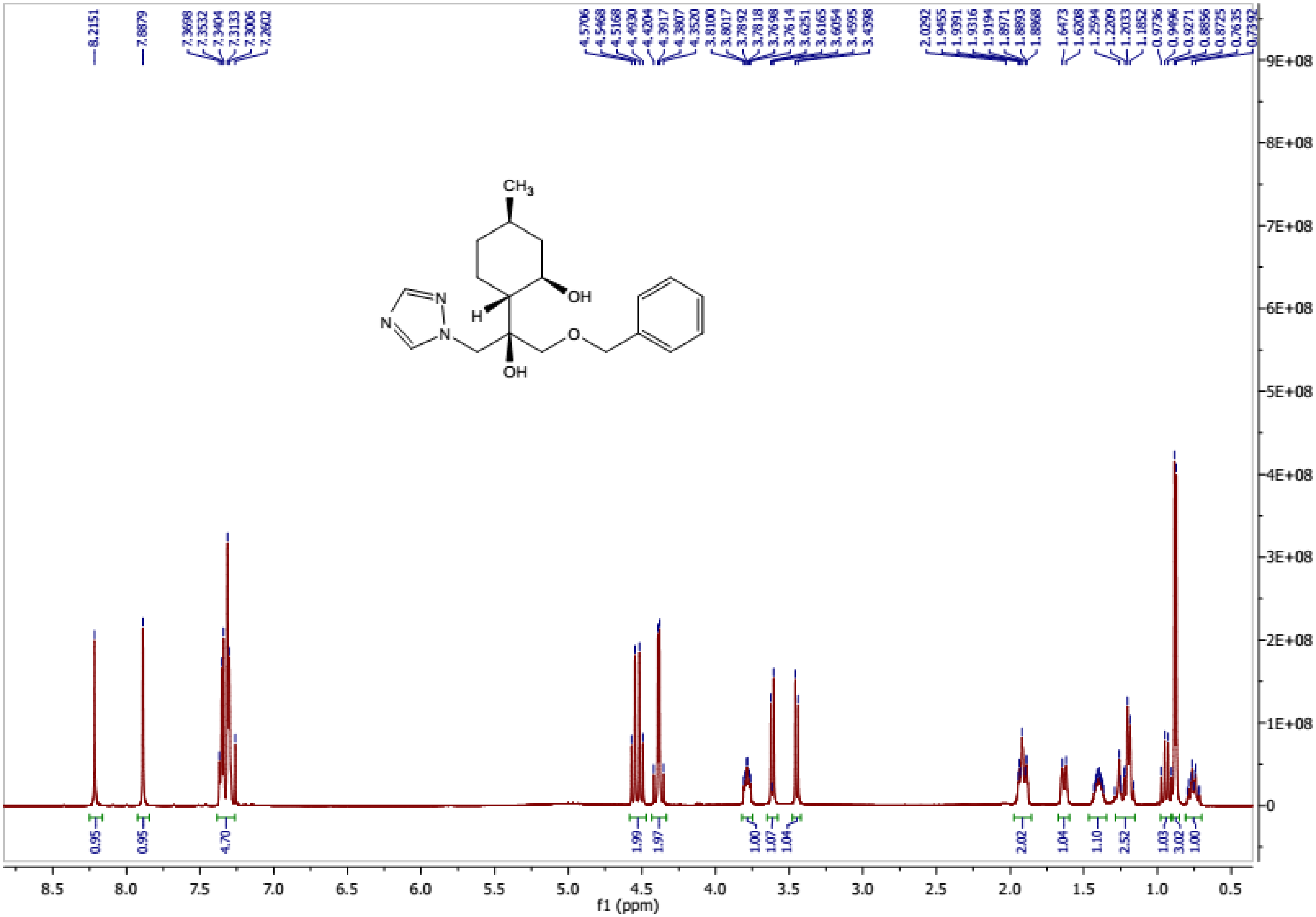

^{13}C-NMR of compound **43b**

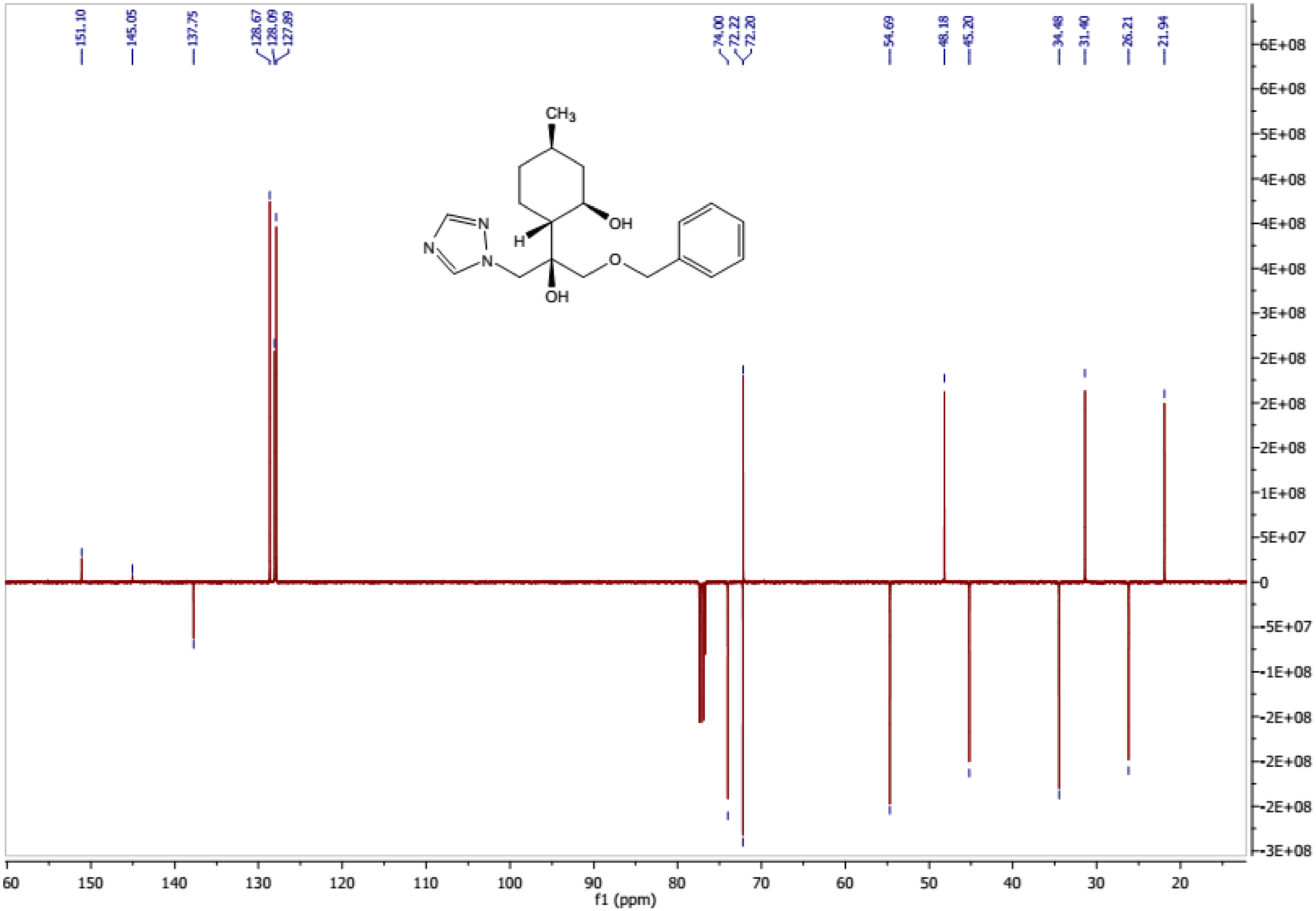

Figure S 128: ^{1}H-NMR of compound **44**

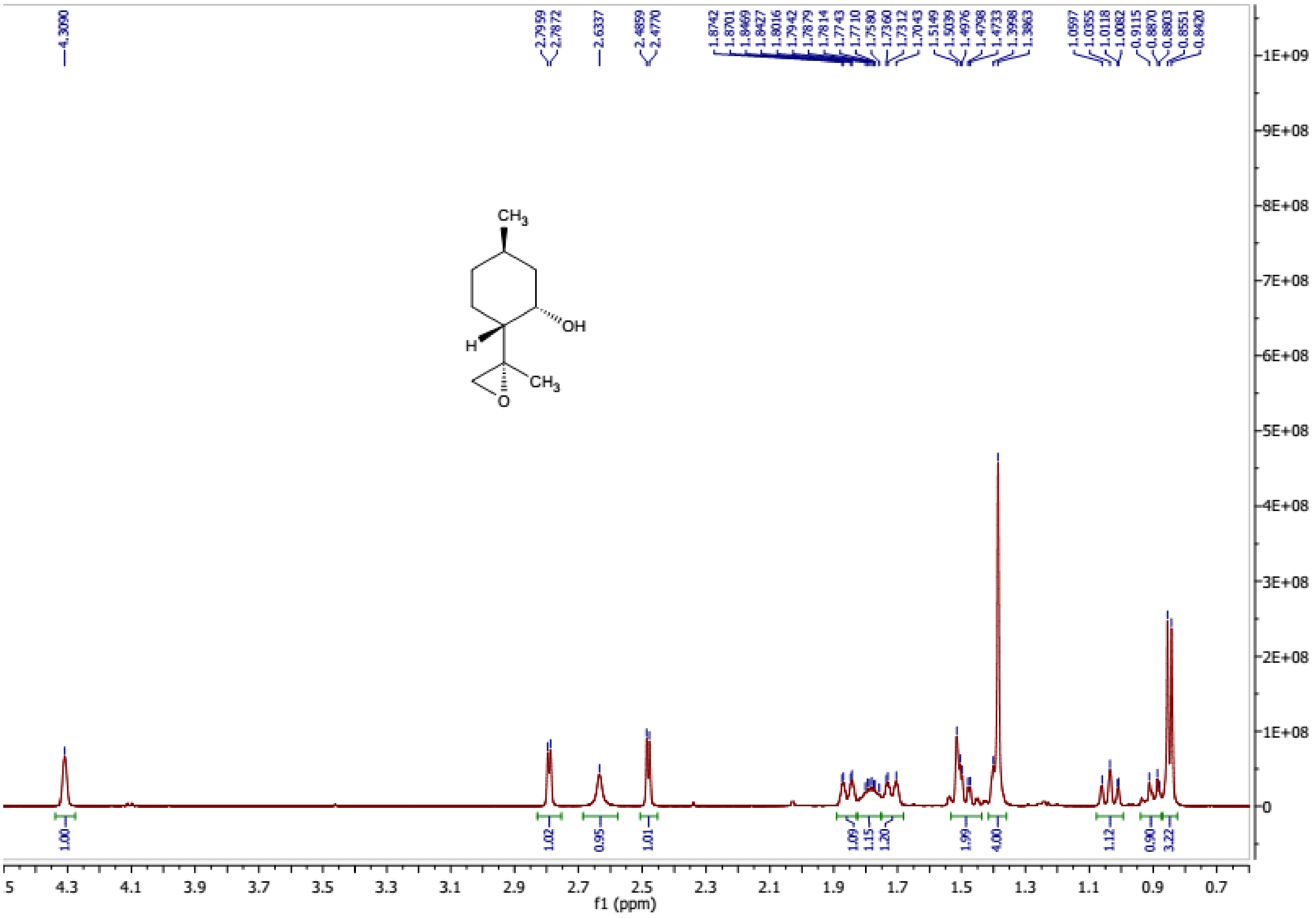

Figure S 129: ^{13}C-NMR of compound **44**

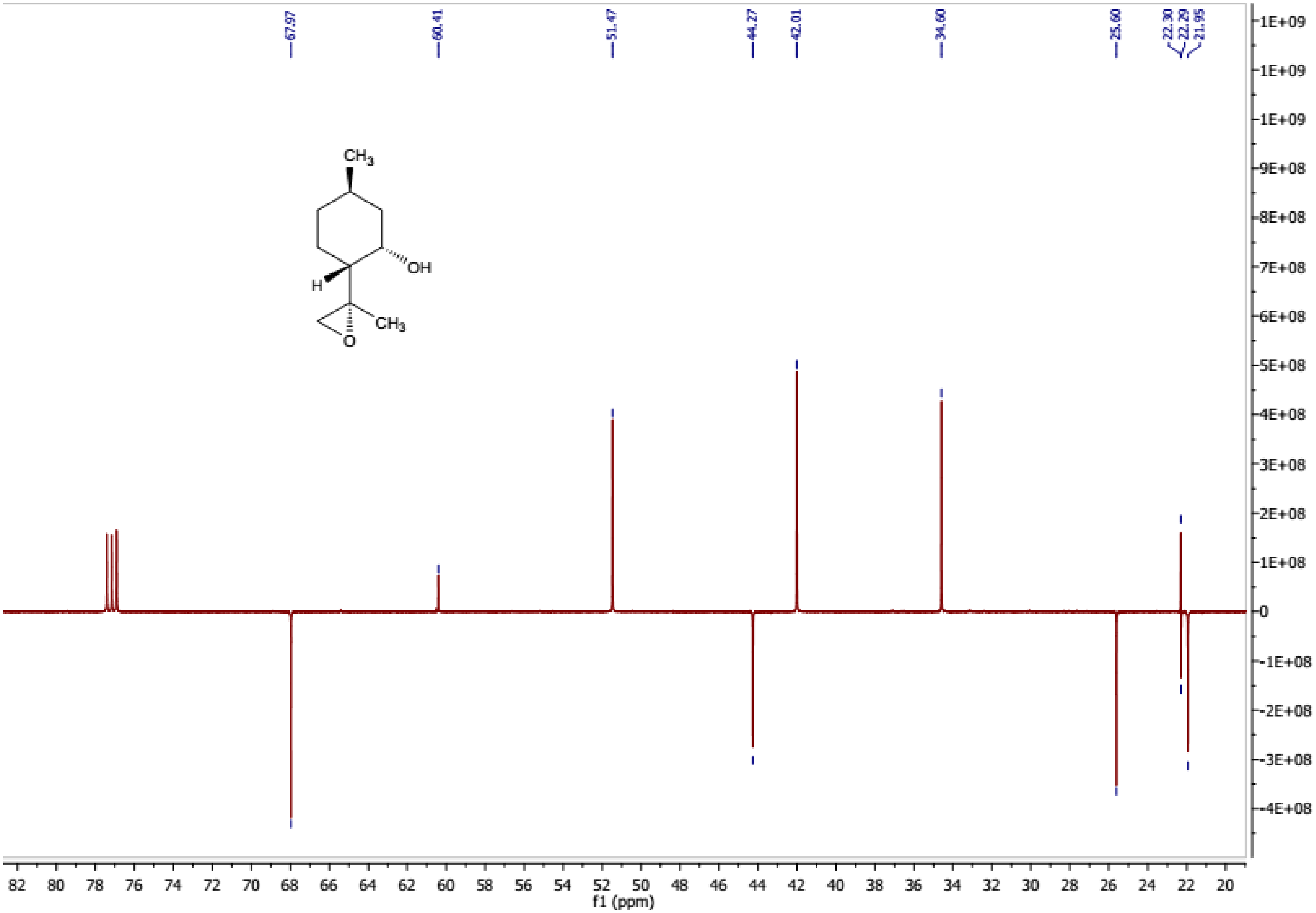

Figure S 130: ¹H-NMR of compound **45**

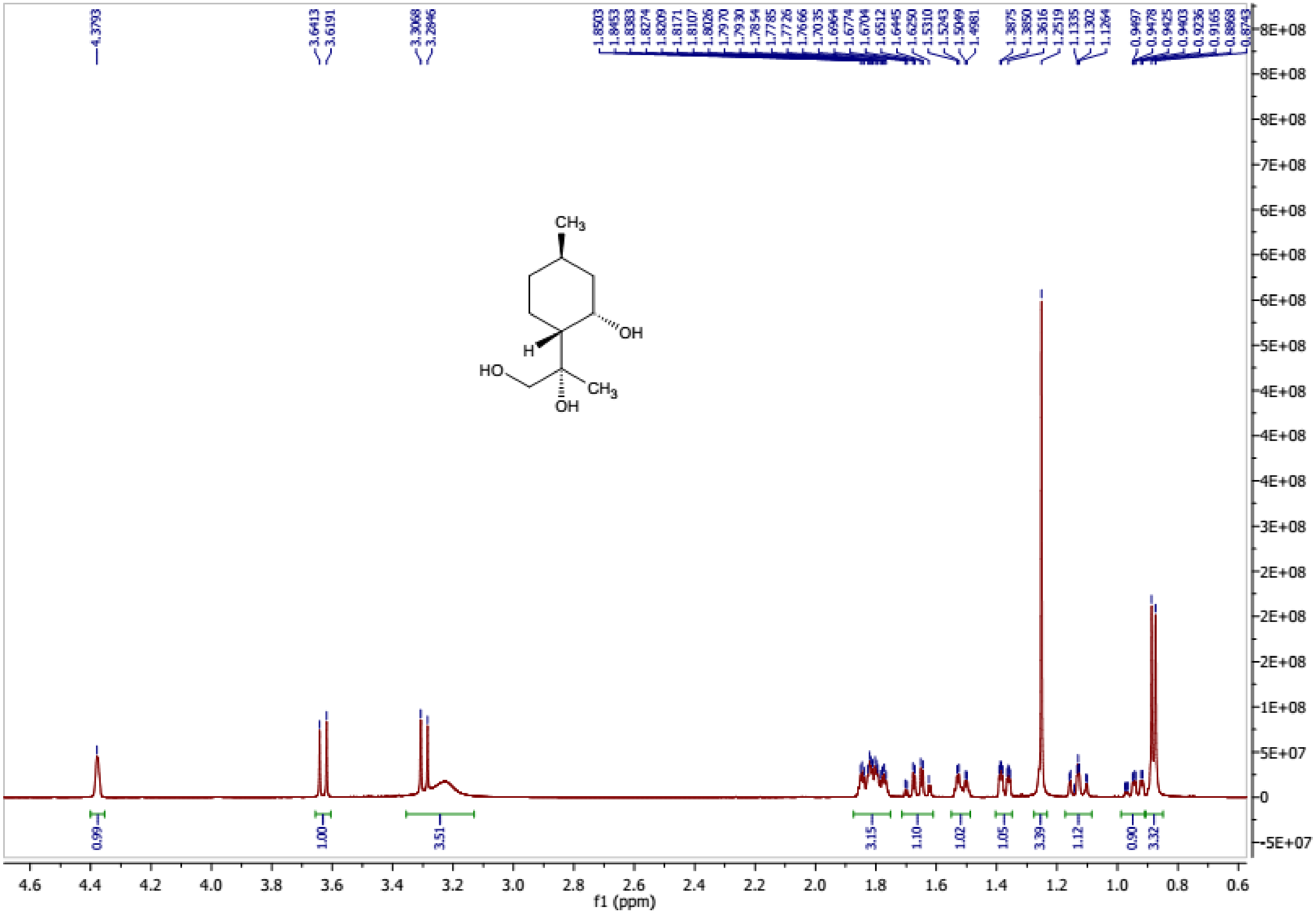

Figure S 131: ^{13}C-NMR of compound **45**

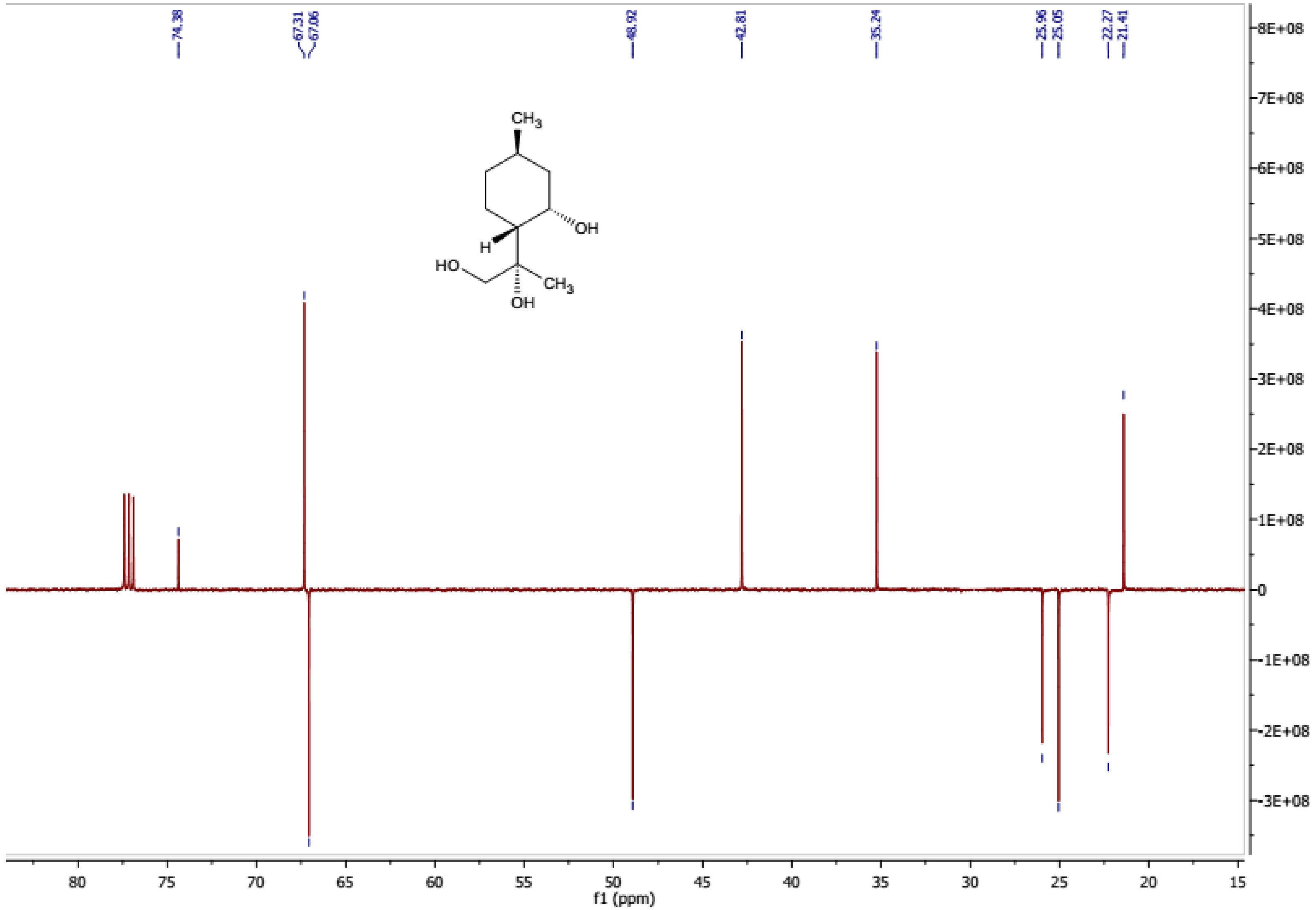

Chiral GC chromatogram of commercially available (-)-isopulegol (**1**)

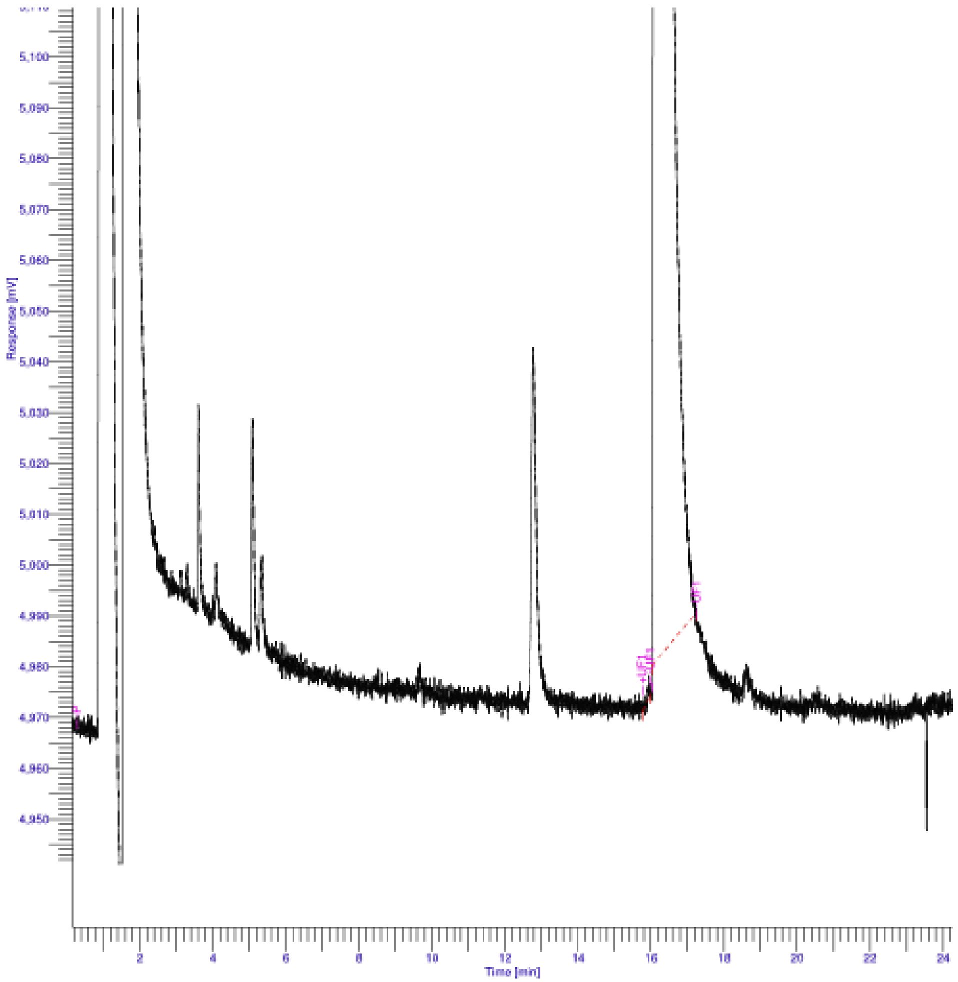

DEFAULT REPORT

Peak #	Time [min]	Area [µV·s]	Height [µV]	Area [%]	Norm. Area [%]	BL	Area/Height [s]
1	15,943	13,96	4,39	0,07	0,07	*MM	3,1823
2	16,177	19423,27	919,14	99,93	99,93	*MM	21,1319
		19437,23	923,53	100,00	100,00		

Chiral GC chromatogram of commercially available (+)-isopulegol

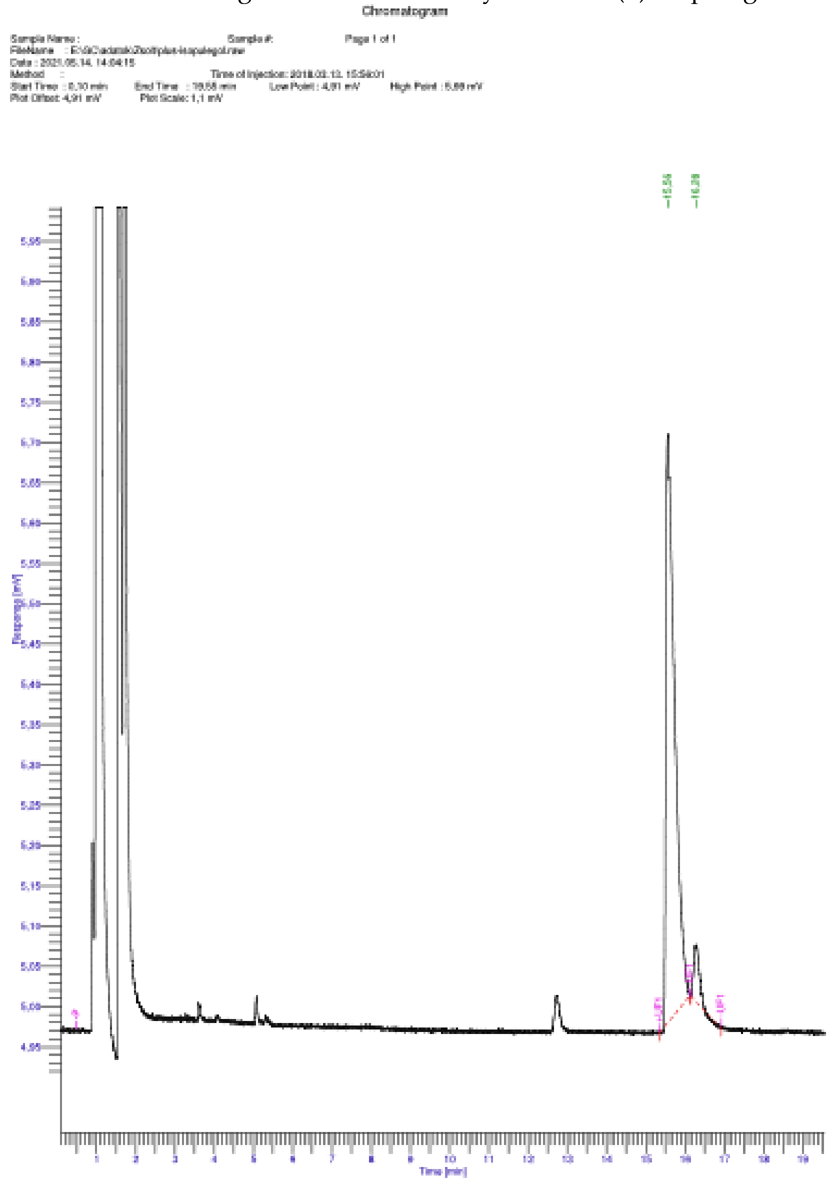

DEFAULT REPORT

Peak #	Time [min]	Area [µV·s]	Height [µV]	Area [%]	Norm. Area [%]	BL	Area/Height [s]
1	15,564	11359,73	729,80	93,88	93,88	*MM	15,5655
2	16,277	740,74	70,60	6,12	6,12	*MM	10,4920
		12100,47	800,40	100,00	100,00		
